# Biotransformation of Bisphenol by Human Cytochrome P450 2C9 Enzymes: A Density Functional Theory Study

**DOI:** 10.1021/acs.inorgchem.2c03984

**Published:** 2023-01-18

**Authors:** Artur Hermano Sampaio Dias, Rolly Yadav, Thirakorn Mokkawes, Asheesh Kumar, Munir S. Skaf, Chivukula V. Sastri, Devesh Kumar, Sam P. de Visser

**Affiliations:** § Manchester Institute of Biotechnology and Department of Chemical Engineering, The University of Manchester, 131 Princess Street, ManchesterM1 7DN, United Kingdom; ‡ Center for Computing in Engineering & Sciences, University of Campinas, Rua Josué de Castro, s/n, Campinas13083-861, Brazil; # Department of Physics, Babasaheb Bhimrao Ambedkar University, Lucknow, Uttar Pradesh (U.P.)226025, India; ⊥ Department of Chemistry, Indian Institute of Technology Guwahati, Guwahati, Assam781039, India; † Department of Physics, Siddharth University, Kapilvastu, Siddharthnagar272202, India

## Abstract

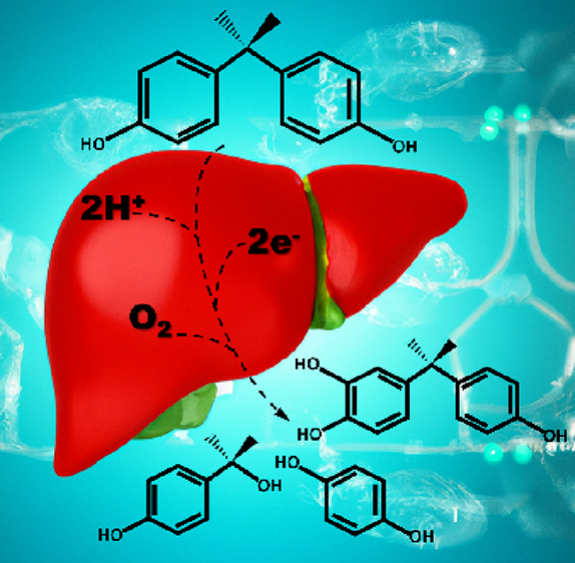

Bisphenol A (BPA, 2,2-bis-(4-hydroxyphenyl)propane) is used as a precursor in the synthesis of polycarbonate and epoxy plastics; however, its availability in the environment is causing toxicity as an endocrine-disrupting chemical. Metabolism of BPA and their analogues (substitutes) is generally performed by liver cytochrome P450 enzymes and often leads to a mixture of products, and some of those are toxic. To understand the product distributions of P450 activation of BPA, we have performed a computational study into the mechanisms and reactivities using large model structures of a human P450 isozyme (P450 2C9) with BPA bound. Density functional theory (DFT) calculations on mechanisms of BPA activation by a P450 compound I model were investigated, leading to a number of possible products. The substrate-binding pocket is tight, and as a consequence, aliphatic hydroxylation is not feasible as the methyl substituents of BPA cannot reach compound I well due to constraints of the substrate-binding pocket. Instead, we find low-energy pathways that are initiated with phenol hydrogen atom abstraction followed by OH rebound to the phenolic *ortho*- or *para*-position. The barriers of *para*-rebound are well lower in energy than those for *ortho*-rebound, and consequently, our P450 2C9 model predicts dominant hydroxycumyl alcohol products. The reactions proceed through two-state reactivity on competing doublet and quartet spin state surfaces. The calculations show fast and efficient substrate activation on a doublet spin state surface with a rate-determining electrophilic addition step, while the quartet spin state surface has multiple high-energy barriers that can also lead to various side products including C^4^-aromatic hydroxylation. This work shows that product formation is more feasible on the low spin state, while the physicochemical properties of the substrate govern barrier heights of the rate-determining step of the reaction. Finally, the importance of the second-coordination sphere is highlighted that determines the product distributions and guides the bifurcation pathways.

## Abstract 2

DFT studies on the reaction mechanism of bisphenol biodegradation by cytochrome P450 2C9 explain the formation of hydroxycumyl alcohol and 3-hydroxybisphenol product formation. The product distributions are governed by the second-coordination sphere of the substrate-binding pocket.

## Introduction

**Scheme 1:**
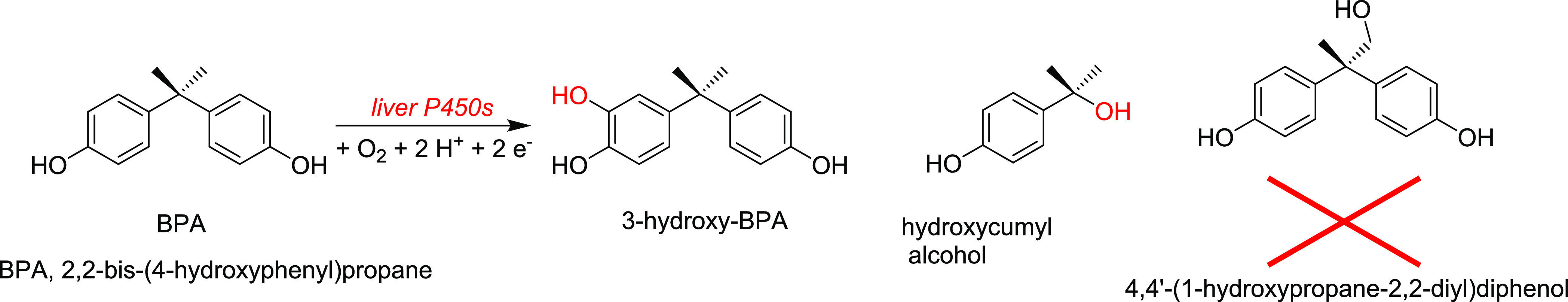
Products Obtained from BPA Metabolism by Human P450 Isozymes

Bisphenol A (BPA, 2,2-bis-(4-hydroxyphenyl)propane, Scheme 1) is used universally in industrial processes for the synthesis of polycarbonate and epoxy plastics. Due to its extensive use, BPA is ever present in the environment and humans are exposed to it through dietary and non-dietary sources.^1,2^ In particular, inside the human body, it behaves as a weak estrogen and thus is regarded as one of the endocrine-disrupting chemicals.^3−5^ Various studies carried out so far reported on the extensive occurrence of BPA in human serum, placental tissue, umbilical cord blood, urine, and breast milk, posing serious threats to human health.^1,2,6^ As a consequence, BPA has been shown to have prominent developmental, reproductive, immune, cardiovascular, and metabolic effects.^6^ Due to its toxicological nature, efforts are in progress by, e.g., food companies, to find substitutes of BPA, and currently various of its analogues are being tested.^7^ These analogues often share the basic structural features of two phenol groups separated by an alkyl chain.

The most common products from activation of BPA by biosystems are shown in Scheme 1 and result in either aromatic ring hydroxylation to give 3-hydroxy-BPA, methyl hydroxylation to give 4,4′-(1-hydroxypropane-2,2-diyl)diphenol or release of fragmentation products like hydroxycumyl alcohol. Interestingly, bacterial P450s were found to produce mostly aliphatic hydroxylation products, while plant (apple) P450s dominantly produced 3-hydroxy-BPA.^8−10^ In mammals including humans, BPA was shown to be activated by the P450 group of enzymes in the liver resulting in toxic metabolites that affected the hormone biosynthesizing P450 enzymes.^7,11−14^ In particular, the isozymes mainly responsible for BPA activation are the CYP2C subfamily in the liver.^13^ Conjugation with the enzyme and sulfate is responsible for almost 90% of activity of all BPA metabolites,^15−17^ and hence, they work as an inhibitor of P450 enzymes. Thus, in human P450s, BPA reacts through aromatic hydroxylation resulting in the formation of a catechol (3-hydroxy-BPA, Scheme 1), which is a metabolite that shows weak androgenic and antiandrogenic activities.^18,19^ Further oxidation of 3-hydroxy-BPA produces the *ortho*-quinone, i.e., BPA-3,4-quinone, which was found to form adducts with DNA and therefore is a toxic metabolite.^20^ In addition to monohydroxylated metabolites, the P450s react with BPA to form hydroxycumyl alcohol, although the product distributions vary between the different P450 isozymes. How these various products are formed through P450 activation of BPA remains under dispute; therefore, we decided to start a computational study using large enzymatic structures.

The P450s are found in nearly all life forms in nature and are involved mostly in phase I metabolism reactions.^21−26^ They carry out the oxygen insertion reactions of a variety of different substrates through the use of molecular oxygen, two reduction equivalents, and two protons on a heme active site.^27−31^ The P450s are regarded as versatile catalysts used by nature for biotransformation reactions with functions related to biosynthesis, detoxification processes, and carcinogenicity via DNA mutations.^22−33^ Details of the reaction mechanisms catalyzed by the P450s have been studied over the past few decades through a combination of experimental and computational approaches.^27,34−39^ It is generally believed that P450 catalysis proceeds through the formation of a strong oxidant in the catalytic cycle, namely, the iron(IV)-oxo cation radical species (compound I, CpdI).^36,39^ CpdI was characterized spectroscopically,^40^ while theoretical studies have focused on its electronic structure, properties, and reactivity.^41−47^ It was shown to have an unusual ground state with a quasi-degenerate pair of triradicaloid states, denoted as ^2^A_2u_ and ^4^A_2u_, with the same electronic configuration and unpaired electrons that contain an electronic triplet spin on the iron-oxo moiety (in π*_xz_ and π*_yz_ orbitals) coupled to an unpaired electron residing in a π-type orbital of the porphyrin ring with a_2u_ symmetry.^48^

Modeling and computational analysis of enzymatic reactions such as P450 oxygenation have given understanding into the electronic features that govern reactivity while also complementing experimental information.^27,49^ Density functional theory (DFT)-based calculations were employed to thoroughly understand energy profiles and the intrinsic electronic features and its contribution to selectivity and reactivity.^49−57^ In this work, we focus on realistic enzymatic structures of the liver P450 2C9 and ask ourselves what products can be expected from a reaction between P450 CpdI and BPA. We find a novel pathway starting with hydrogen atom abstraction from the phenol group followed by a bifurcation pathway of OH attack on either the *ortho*- or *para*-position with respect to the phenol group. These mechanisms are shown to be affected by the size and shape of the substrate-binding pocket and possible proton relay channels.

## Methods

### Model Setup

**Figure 1:**
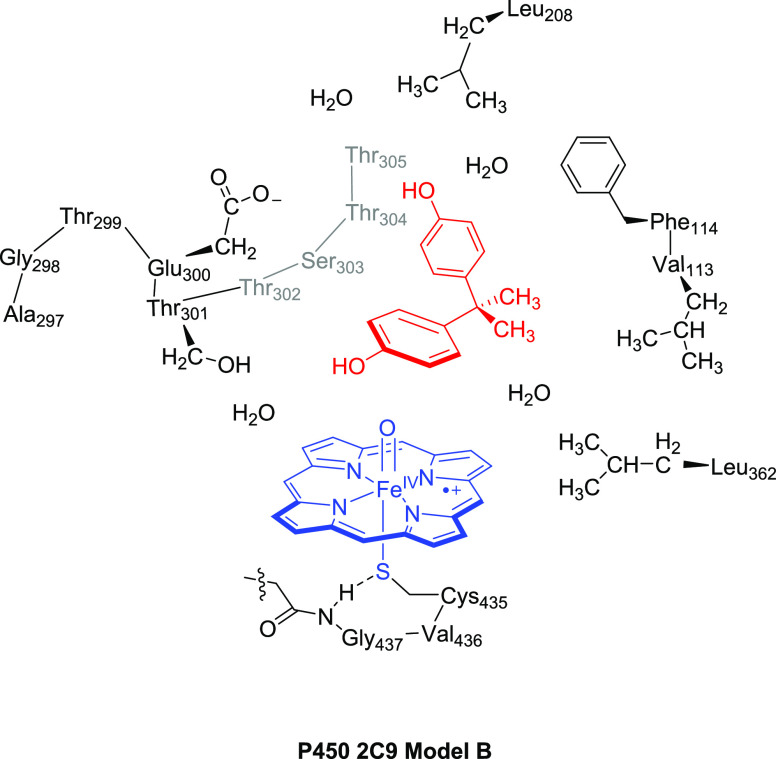
Model **B** as studied in this work.

Our models are based on typical crystal structure coordinates of P450 isozymes and contain the heme- and substrate-binding areas only. These large structural models that include the first- and second-coordination spheres of the oxidant and substrate are generally good mimics that describe enzymatic reactivity well and consider substrate and oxidant positioning. Previous studies on large active-site cluster models from our group have shown that they can reproduce experimental product distributions and rate constants well.^58,59^ As the dominant isozymes responsible for BPA activation in the liver are the CYP2C subfamily,^13^ we started the work from the P450 2C9 crystal structure coordinates as deposited in the 1OG5 Protein Data Bank (PDB) file.^60,61^ This is an enzymatic dimer with warfarin bound. We selected chain A of the PDB file and replaced warfarin with BPA manually. Hydrogen atoms were added in Chimera assuming pH 7 conditions.^62^ Based on the structure, we then created active site cluster models. Exploratory work was done with a minimal cluster model, i.e. model **A** (shown in blue and red in Figure 1), which is a truncated model that consists of the iron embedded into protoporphyrin IX with all side chains replaced by hydrogen atoms and cysteinate abbreviated by thiolate, i.e., SH^–^. Its distal site is connected to the oxo oxygen. Model **A** has overall charge 0 and was calculated in the doublet and quartet spin states. We will focus here, however, on the larger active site model (model **B**) of 222 atoms (Figure 1) that incorporates part of the protein that describes the substrate-binding pocket. In particular, model **B** contains model **A** supplemented with the protein chains Val_113_–Phe_114_ and Ala_297_–Gly_298_–Thr_299_–Glu_300_–Thr_301_–Thr_302_–Ser_303_–Thr_304_–Thr_305_, whereby the Thr_299_ and Ser_303_ residues were truncated to Gly. In addition, the side chains of Leu_208_ and Leu_362_ and three water molecules were included in the model. As hydrogen bonding interactions to the axial ligand can sometimes affect selectivities, we expanded the axial ligand description to Cys_435_–Val_436_–Gly_437_, whereby Val was truncated to Gly. Protein residues were truncated by replacing an aliphatic C–C bond by C–H. No atoms were fixed in the geometry optimizations.

### Procedures

DFT-based calculations were performed using the Gaussian09 software package.^63^ All local minima and transition state structures were optimized in the gas phase without constraints. Two spin states were taken into consideration for the study of the overall reaction mechanism, namely, the *S* = ^1^/_2_ and *S* = ^3^/_2_ spin states. The calculations were done using the unrestricted hybrid density functional UB3LYP^64,65^ with a split-valence basis set described with the LANLD2Z basis set with effective core potential on iron, and for the remaining of the atoms (C, H, N, O, and S), 6-31G* was employed, designated basis set one (BS1).^66,67^ Relaxed potential energy scans (PES) were performed along various reaction coordinates to establish potential mechanisms and to obtain starting structures for the transition state searches along a particular degree of freedom. Full geometry optimizations for the transition state structures were performed and ascertained by an analytic frequency calculation at the same level of the theory. A single imaginary frequency involving the correct vibrational distortion confirmed the structure to be a transition state, while local minima had real frequencies only.

To validate the results, single-point calculations with a larger basis set were performed. The basis set used here is referred to as basis set two (BS2), and it involves a triple-ζ quality basis set with effective core potential on iron (cc-pVTZ) and a 6-311+G* basis set on other atoms. These calculations also included a conductor-type polarized continuum model with a dielectric constant mimicking chlorobenzene (ε = 5.7),^68^ which is a value typical for enzymatic active sites.^69,70^ The methods and approaches applied in this work have been extensively tested and validated against experimental data and were shown to reproduce spectroscopic constants, and free energies of activation of oxygen atom transfer reactions have been experimentally determined well.^71−73^ Although all energies reported in this work exclude dispersion corrections, actually the effect of dispersion on the energetics and geometries was also tested using the method of Grimme, but little change in the optimized geometries was seen and the same electronic configuration was obtained (Supporting Information, Figure S3).^74^

## Results and Discussion

**Figure 2:**
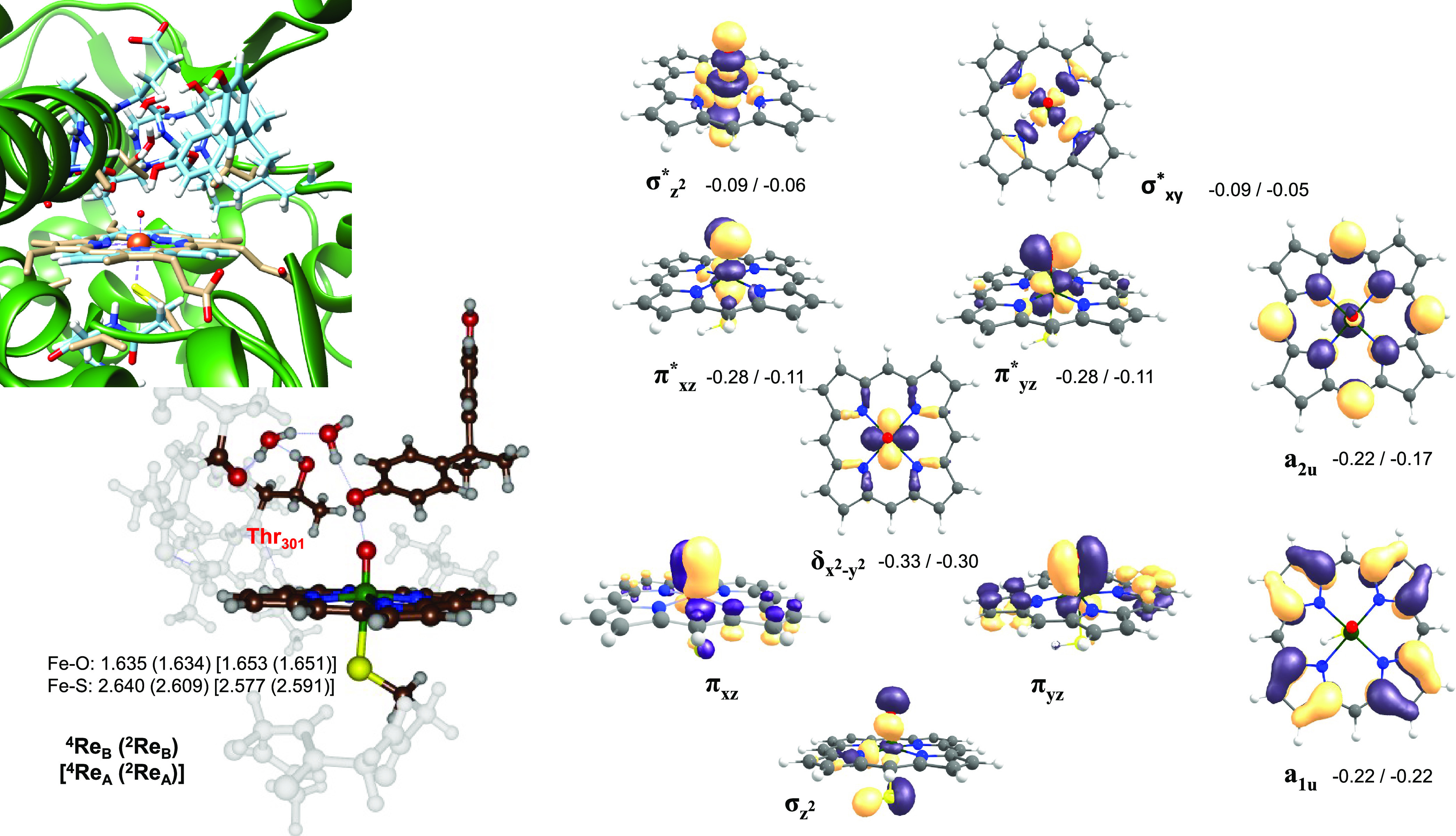
Optimized geometries of the reactant complexes in the doublet and quartet spin states for models **A** and **B**. Bond lengths are in Ångströms. Also shown are relevant molecular valence orbitals as obtained for model **A** with orbital energies of ^4^**Re**_B_. The overlay depicts the structures of ^2^**Re**_B_ with chain A of the 1OG5 PDB file.

We started our work with a detailed analysis of the reactant complexes for models **A** and **B**. The reactant complexes **Re**_A_ and **Re**_B_ represent CpdI models of an iron(IV)-oxo heme cation radical species, and their structures are shown in Figure 2. In both structures, the lowest energy doublet and quartet spin states are close in energy (within 1 kcal mol^–1^). Geometrically, the Fe–O distance is short and typically around 1.63 Å for model **A** and 1.65 Å for the model **B** structures, which matches previous calculations on P450 CpdI well.^41−47,49,50,75,76^ The Fe–S bond is rather long, and specifically with the axial protein chain included in model **B**, it is around 2.609 Å for ^2^**Re**_B_ and 2.640 Å for ^4^**Re**_B_, while for the truncated model **A** the distances are slightly shorter. Previous studies using either QM/MM or DFT cluster models gave CpdI Fe–S distances in the range 2.512–2.676 Å;^27,42,44,77−80^ hence, our calculated values fit that window nicely. The optimized geometry compares well with the crystal structure coordinates, and an overlay of the two (left-hand side of Figure 2) gives a good match. As such, no dramatic changes have occurred during the geometry optimizations. The substrate phenol group in ^4,2^**Re**_B_ undergoes hydrogen bonding interactions with two water molecules in the substrate-binding pocket that link it with the alcohol group of Thr_301_ and the carbonyl group of Ala_297_.

Figure 2 also shows the relevant valence orbitals of CpdI on the right-hand side. As discussed before,^27,34^ the valence orbitals are dominated by the 3d metal orbitals and several π* heme orbitals. The *z*-axis here is considered along the O–Fe–S axis perpendicular to the plane of the porphyrin ring, while the *x*- and *y*-axes are placed in between Fe–N bonds of porphyrin. The lowest energy orbitals are shown at the bottom of Figure 2 and represent bonding-type metal orbitals along the Fe–O bond, namely, the σ_z_2, π_xz_, and π_yz_ orbitals. The former is formed from the interactions of the 3d_z_2 orbital on iron and the 2p_z_ orbital on the oxo group, while the latter two are from mixing of the 3d_xz_ and 3d_yz_ metal orbitals with the 2p_x_ and 2p_y_ orbitals on the oxygen atom. These three molecular orbitals are doubly occupied orbitals during the entire reaction mechanism and are low in energy. The mixing also gives rise to the antibonding pair of degenerate orbitals π*_xz_ and π*_yz_. In CpdI, the π*_xz_ and π*_yz_ orbitals are singly occupied but their occupancy changes as the reaction proceeds. A low-lying doubly occupied non-bonding orbital δ_x_2_-y_2 resides in the plane of the heme and is shown in the middle image of Figure 2.

**Scheme 2:**
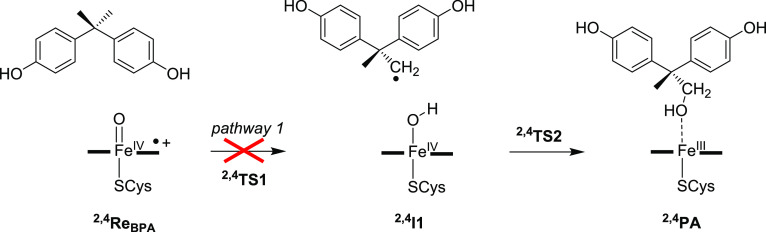
Aliphatic Hydroxylation Pathway of Methyl Activation of BPA by P450 CpdI

**Scheme 3:**
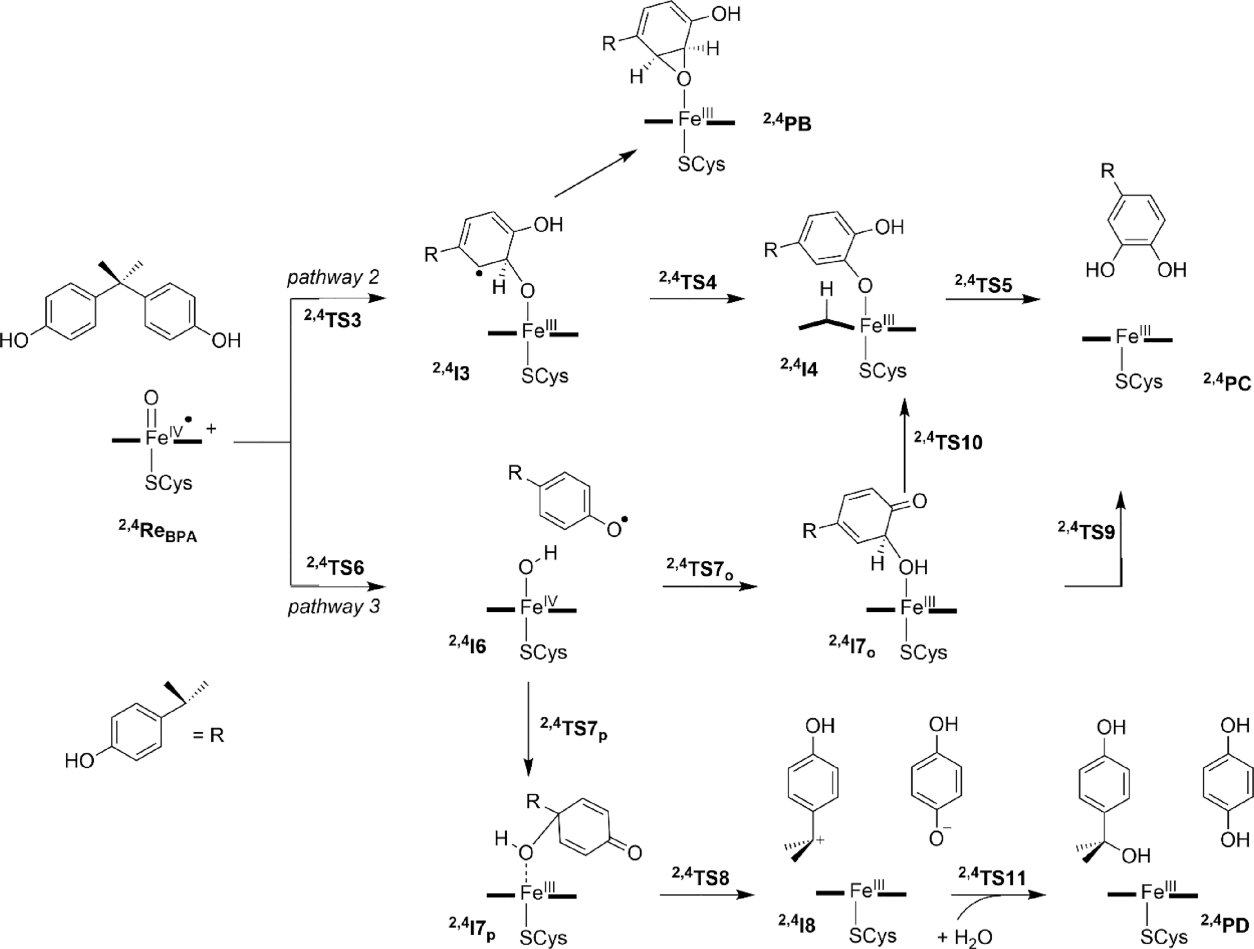
Viable Reaction Mechanisms of BPA Activation by P450 CpdI Explored in This Work

On the complete right-hand side of Figure 2 are given two porphyrin ring orbitals, which under D_4h_ symmetry have the labels a_1u_ and a_2u_.^48^ These orbitals are degenerated for an isolated porphyrin without distal and axial ligands, but the binding of axial and distal ligands leads to a mixing of metal orbitals particularly with the porphyrin a_2u_ orbital that is thereby raised in energy. To be specific, the mixing of the a_2u_ orbital on porphyrin with orbitals from an anionic axial ligand such as thiolate raises the mixed molecular orbital substantially in energy. The two unpaired electrons in π*_xz_ and π*_yz_ ferromagnetically or antiferromagnetically couple to the unpaired electron in the a_2u_ orbital to give an overall quartet or doublet spin state for the CpdI complex. Finally, there are two antibonding virtual orbitals, i.e., σ*_z_2 and σ*_xy_, that result from the mixing of metal, oxygen, and the sulfur atom of the axial thiolate ligand along the O–Fe–S bond to generate σ*_z_2 or a mixing of metal and nitrogen lone pair orbitals to give rise to σ*_xy_. Overall, CpdI in the doublet and quartet spin states has orbital occupation σ_z_2^2^ π_xz_^2^ π_yz_^2^ δ^2^ a_1u_^2^ π*_xz_^1^ π*_yz_^1^ a_2u_^1^. In the quartet spin state, the three unpaired electrons are ferromagnetically coupled, while in the doublet spin state, the two π* electrons are up-spin whereas the a_2u_ electron is down-spin. The doublet and quartet spin states are within 1 kcal mol^–1^ in energy, and local environmental perturbations and the nature of the axial ligand determine their energy splittings. Next, we investigated the competitive aliphatic and aromatic hydroxylation pathways and phenol hydrogen abstraction of BPA catalyzed by P450 CpdI following the mechanisms shown in Schemes 2 and 3. The aliphatic and aromatic hydroxylation reaction pathways have been extensively studied for P450-catalyzed reaction mechanisms for a variety of alternative substrates and generally show stepwise pathways.^27,34,54,81−90^ The aliphatic hydroxylation mechanism (Scheme 2) initially begins with the abstraction of the hydrogen atom of the substrate by CpdI. This happens via two-state reactivity (TSR) patterns on competing doublet (low-spin, LS) and quartet (high-spin, HS) spin surfaces as they are degenerate for CpdI.^27^

Generally, H abstraction results in the formation of a radical intermediate (**I1**) via transition state **TS1** and is followed by a rebound transition state (**TS2**) to produce an alcohol product (**PA**).^54,81−83,91−94^ The aliphatic hydroxylation pathway (designated pathway 1) was attempted for the BPA-bound P450 cluster model **B**; however, due to the tight substrate-binding pocket of the enzyme, the BPA substrate cannot rotate sufficiently in the substrate-binding pocket to bring the substrate methyl groups pointing toward the heme. All attempts to calculate pathway 1, therefore, failed and led to a collapse of model **B** (without geometric constraints) or high-energy pathways (with geometric constraints on atoms of the protein). Consequently, aliphatic hydroxylation of the methyl groups of BPA by P450 2C9 can be ruled out as a viable mechanism due to the shape and size of the substrate and the P450 substrate-binding pocket. This result matches experimental work on the reaction of BPA with P450 2C9 that did not detect any products originating from aliphatic hydroxylation of the methyl groups.^15−18^

As aliphatic hydroxylation of BPA by human P450 2C9 structures appears to be an unviable reaction channel, we considered two alternative pathways for oxygen atom transfer to BPA by CpdI of P450 2C9 instead, as described in Scheme 3. These mechanisms are designated pathway 2 for direct aromatic hydroxylation and pathway 3 for phenol activation followed by OH rebound to either the *ortho*- or *para*-position with respect to the phenol group. In contrast to the aliphatic hydroxylation by P450 CpdI, in the aromatic hydroxylation mechanism, an electrophilic reaction step for the C–O bond formation between substrate and oxy-ferryl cation radical species (CpdI) via transition state **TS3** leads to the intermediate **I3**.^84^ The electrophilic attack (or π attack) of CpdI on the arene ring results in the formation of a Meisenheimer complex, which is either a cation-type intermediate (**I3**_cat_) or a radical intermediate (**I3**_rad_). Previous work showed these cation- and radical-type intermediates to be within a few kilocalories per mole and their ordering and relative energies were found to be influenced by external perturbations.^95,96^ Subsequently, a proton is transferred from the *ipso*-carbon of the substrate to the nearest pyrrole nitrogen atom via a transition state **TS4** (H → N) barrier to form the protonated porphyrin intermediate **I4**. The proton shuttle to the nitrogen of the porphyrin ring happens as a result of the basic nature of nitrogen atoms of the porphyrin scaffold. From intermediate **I4**, a subsequent proton shuttle to the oxo group of the substrate then results in the hydroxylated product **PC**, via transition state **TS5** (H → O).

An alternative pathway from **I3** would lead to ring closure via transition state **TS4′** to form the epoxide-bound product **PB**. This mechanism was proposed for BPA activation by P450 CpdI using minimal cluster models, e.g., model **A**.^97^ However, for model **B**, the epoxidation reaction is strongly endothermic (see the Supporting Information for details) and encounters a high barrier. Therefore, we do not expect P450 2C9 to react with BPA to give epoxide products and, indeed, this product has never been characterized from human P450 reactivities.

Finally, CpdI can react with BPA through hydrogen atom abstraction from the phenolic O–H group via transition state **TS6** to form the iron(IV)-hydroxo with phenolate radical intermediate **I6**. It has been hypothesized based on experimental product distributions that the phenolate radical is attacked by the OH group at either the *ortho*- or *para*-position to trigger a pathway leading to 3-hydroxy-BPA and hydroxycumyl alcohol products.^14^ Indeed, DFT calculations on small cluster models of P450 CpdI with BPA showed this a feasible pathway.^98^ Furthermore, recent calculations on P450 OxyB showed that the radical obtained after phenol hydrogen atom abstraction can attack another aromatic ring and lead to ring closure to create the three-dimensional structure of the antibiotic vancomycin.^99^

We hypothesized, therefore, that a similar mechanism may apply here whereby structure **I6** reacts further, for instance through OH transfer to the radical. Two pathways were tested for the OH group transfer from **I6**, namely, attack on the *ortho*-position of the radical via transition state **TS7**_o_ to form the quinone intermediate **I7**_o_ or attack on the *para*-position via transition state **TS7**_p_ to give intermediate **I7**_p_. A C–C bond dissociation in the quinone of **I7**_p_ would then lead to deprotonated dihydroquinone and hydroxycumyl cation through heterolytic cleavage. With the assistance of a water molecule, the final products dihydroquinone and hydroxycumyl alcohol or **PD** are formed. In contrast, a proton relay from **I7**_o_ would lead to the catechol product **PC**. Alternatively, in structure **I7**_o_, the proton is shuttled to the porphyrin ring to give intermediate **I4** that relays the proton back to the phenol oxygen atom to give final products **PC**.

**Figure 3:**
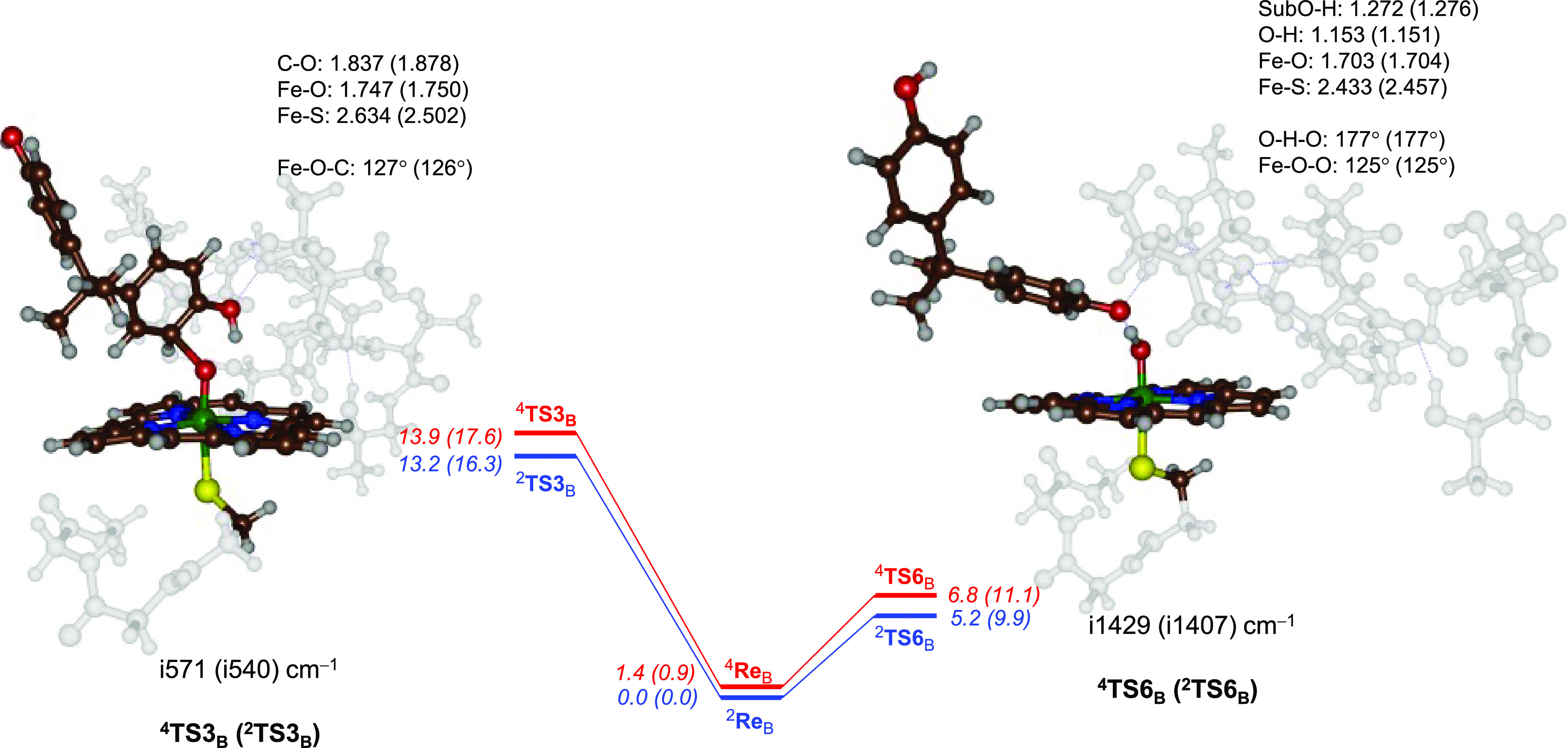
Competitive phenol hydrogen atom abstraction (via **TS6**) and aromatic electrophilic addition (via **TS3**) transition states from reactant complexes with model **B**. Geometries obtained at UB3LYP/BS1. Energies are zero-point-corrected values at UB3LYP/BS2//UB3LYP/BS1 + ZPE in kilocalories per mole with free energies obtained at 298 K in parentheses. Structures give bond lengths in Ångströms, angles in degrees, and the imaginary frequency of the transition state in per centimeter.

Next, we ran full DFT calculations on the mechanism displayed as pathway 3 in Scheme 2 for the electrophilic attack of CpdI on BPA for model **B**, and the first transition states for these pathways are shown in Figure 3. Surprisingly, the C–O activation barriers ^4,2^**TS3**_B_ are relatively high in energy (Δ*G*^‡^ = 17.6 and 16.3 kcal mol^–1^ in the quartet and doublet spin states) and, hence, the pathways may be too high for reasonable turnover numbers at room temperature. These barriers are substantially higher in energy than those previously calculated for electrophilic addition of CpdI to an aromatic substrate using either small model complexes or large enzymatic cluster models.^84−90^ Clearly, the shape of the substrate-binding pocket and the large substrate size prevent the ideal orientation for direct electrophilic addition pathways in P450 2C9. The transition state geometries of ^4,2^**TS3**_B_ are shown on the left-hand side of Figure 3. The Fe–O bond has elongated with respect to the reactants complexes to 1.75 Å in both transition states, while the aromatic C^6^ atom has approached the oxo group to 1.837/1.878 Å in ^4^**TS3**_B_/^2^**TS3**_B_, respectively. The imaginary frequency represents the C–O stretch vibration and has magnitudes of i571 and i540 cm^–1^ for the two structures. The optimized geometries and imaginary frequencies for the transition states match previous aromatic hydroxylation barriers well.^84−90^

An analysis of the group spin densities and charges shows that the transition states and their subsequent intermediates have an electronic configuration of π*_xz_^2^ π*_yz_^1^ a_2u_^1^ ϕ_Sub_^1^ with ϕ_Sub_ the π-orbital on the substrate. These systems, therefore, have an electronic configuration [Fe^III^(OSub^•^)(Por^+•^)], and an electron transfer from the substrate into the π*_xz_ orbital has taken place. Previous work with smaller models, however, led to an [Fe^IV^(OSub^•^)(Por)] configuration, where the electron transfer went into the a_2u_ orbital instead. We attempted to swap molecular orbitals to find the iron(IV) transition state and radical intermediate, but in all cases, the self-consistent field calculation converged back to the iron(III) state that is therefore the ground state. Most probably the polarity of the model stabilizes the iron(III) state below the iron(IV) state. Previous work also highlighted close energy differences (within a few kilocalories per mole) between the iron(III) and iron(IV) radical intermediates and transition states and the effect of external perturbations on their energy differences and ordering.^100^ Nevertheless, the DFT calculations on the large cluster model **B** implicate that the “normal” P450 mechanisms for aliphatic and aromatic hydroxylation do not apply for BPA activation by P450 2C9 enzymes due to the protein structure, the large shape of the substrate, and the weak phenolic O–H bond. Therefore, we attempted a number of alternative mechanisms to form the experimentally obtained products starting from an initial phenol hydrogen atom abstraction.

**Figure 4:**
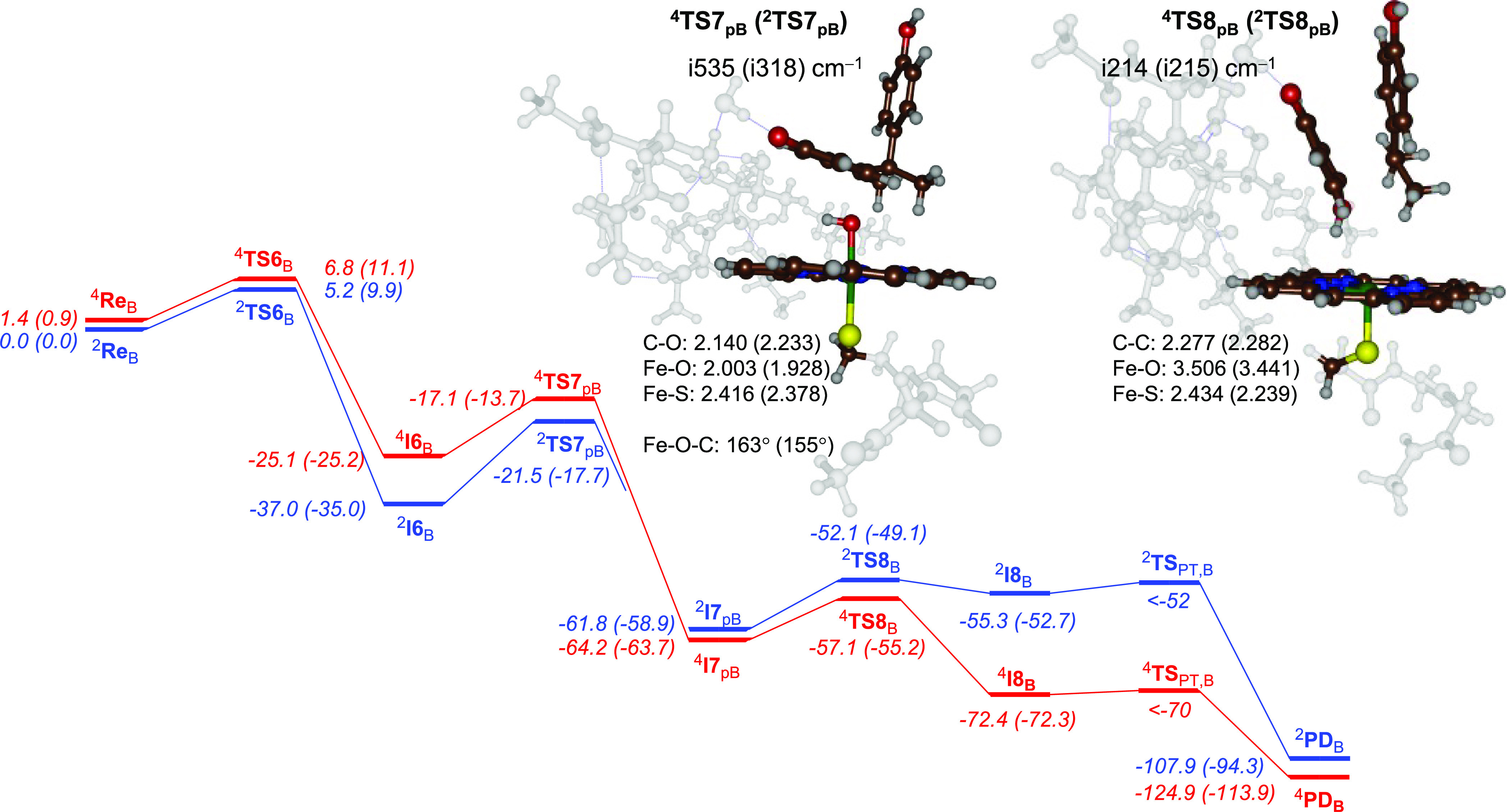
BPA activation by CpdI as described by pathway 3 for *para*-OH rebound for model **B**. Geometries obtained at UB3LYP/BS1. Energies are zero-point-corrected values at UB3LYP/BS2//UB3LYP/BS1 + ZPE in kilocalories per mole with free energies obtained at 298 K in parentheses. Structures give bond lengths in Ångströms, angles in degrees, and the imaginary frequency of the transition state in per centimeter.

Subsequently, we explored reaction mechanisms for 3-hydroxy-BPA and hydroxycumyl alcohol formation after an initial phenolic hydrogen atom abstraction step and start with the pathway leading to hydroxycumyl alcohol products shown in Figure 4. The mechanism essentially follows the pathway described at the bottom of Scheme 3, where after phenolic hydrogen atom abstraction an OH rebound takes place to the *para*-position of the phenol. The obtained product then undergoes a heterolytic C–C cleavage via **TS8** followed by a water-assisted step to form hydroxycumyl alcohol and hydroquinone. Not surprisingly, the mechanism starts with a relatively low energy barrier for substrate activation by CpdI through a phenolic hydrogen atom abstraction via ^4,2^**TS6**_B_ with values of Δ*G*^‡^ = 11.1 and 9.9 kcal mol^–1^ on the quartet and doublet spin states (Figures 3 and 4), respectively. These barriers are similar to phenol hydrogen atom abstraction calculated before for P450 reactions, such as those that take part in the biosynthesis of antibiotics and the biodegradation of lignin components.^78,99^ The optimized geometries are shown in Figure 3 as well. Typically, there are two short O–H bonds between the donor and acceptor oxygen atoms for the hydrogen transfer with the substrate O–H distance at 1.272 and 1.276 Å and the oxo-H distance at 1.153 and 1.151 Å for the quartet and doublet spin state transition state structures, respectively. These transition states have a large imaginary frequency of well over i1400 cm^–1^ for the hydrogen transfer.

The potential energy landscape for the mechanism of BPA activation by P450 CpdI via pathway 3 is shown in Figure 4. As discussed above in Figure 3, the phenolic hydrogen atom abstraction has small barriers (^2,4^**TS6**), and after the hydrogen atom abstraction, the systems collapse to a very stable radical intermediate ^2,4^**I6** that is Δ*G* = −25.2 kcal mol^–1^ more stable in the quartet spin state and Δ*G* = −35.0 kcal mol^–1^ in the doublet spin state. Both **I6** structures have an orbital occupation corresponding to π*_xz_^1^ π*_yz_^1^ a_2u_^2^ ϕ_Sub_^1^; hence, the electronic configuration of the complex is [Fe^IV^(OSub^•^)(Por)] and an electron transfer from the substrate into the a_2u_ orbital has taken place. We tried to reoptimize the quartet spin state structure starting from the doublet spin geometry for **I6**_B_; however, this did not give a lower energy structure.

Next, the OH group from the iron(IV)-hydroxo intermediate **I6** attacks the *para*-position of the aromatic ring with barriers via **TS7**_pB_ of Δ*G*^‡^ = 11.5 kcal mol^–1^ on the quartet spin state with respect to ^4^**I6** and Δ*G*^‡^ = 17.3 kcal mol^–1^ on the doublet spin state as compared to ^2^**I6**. As such, the quartet spin barriers ^4^**TS6**_B_ and ^4^**TS7**_pB_ are of similar magnitude and will determine the rate constant. In contrast, on the doublet spin state ^2^**TS7**_pB_ is well higher in energy than^2^**TS6**_B_ and consequently there is a rate-determining OH rebound step on the doublet spin state surface. However, since the phenolic hydrogen atom abstraction is a highly exothermic process, we can assume that the energy released for this step will be used to cross the **TS7_p_** barriers rapidly. Optimized geometries of the ^4,2^**TS7**_p_ transition states are shown in Figure 4. They have an imaginary frequency of i535 and i318 cm^–1^ for ^4^**TS7**_p_ and ^2^**TS7**_p_, respectively, for the C–O stretch vibration and the binding of the OH group to the *para*-position. The transition states are early with a relatively long C–O bond of 2.140 Å (^4^**TS7**_p_) and 2.233 Å (^2^**TS7**_p_), while the Fe–O bonds are 2.003 Å (^4^**TS7**_p_) and 1.928 Å (^2^**TS7**_p_).

**Figure 5:**
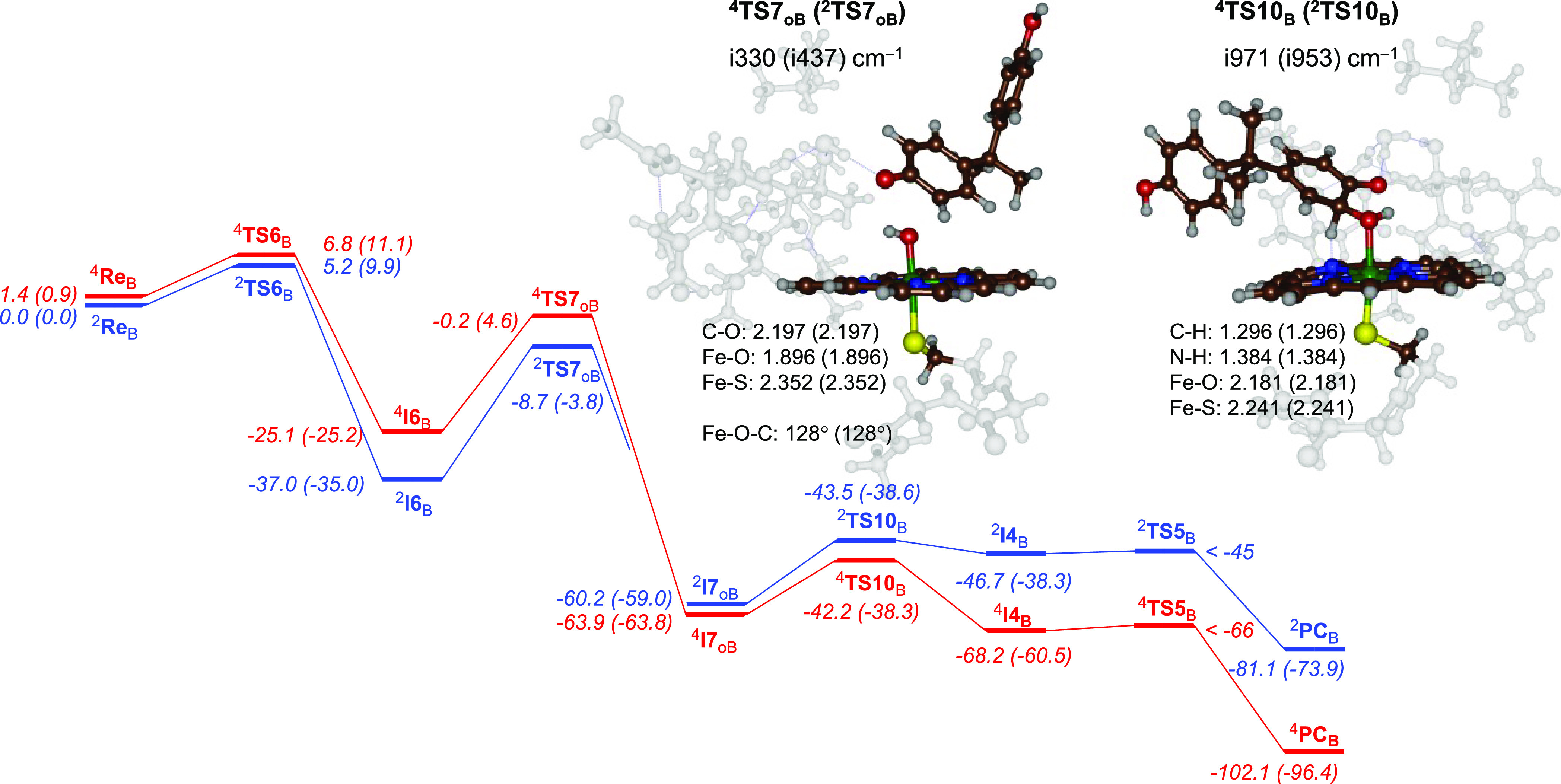
BPA activation by CpdI as described by pathway 3 for *ortho*-OH rebound for model **B**. Geometries obtained at UB3LYP/BS1. Energies are zero-point-corrected values at UB3LYP/BS2//UB3LYP/BS1 + ZPE in kilocalories per mole with free energies obtained at 298 K in parentheses. Structures give bond lengths in Ångströms, angles in degrees, and the imaginary frequency of the transition state in per centimeter.

After the C–O bond formation on the *para*-position of the substrate, the system relaxes to the highly stable ^2,4^**I7_p_** structures that are more stable than reactants by Δ*G* = −58.9 (doublet) and −63.7 (quartet) kcal mol^–1^. These structures have lost the radical character on the substrate and have configuration ^2^**I7**_p_ = π*_xz_^2^ π*_yz_^1^ a_2u_^2^ ϕ_Sub_^0^ and ^4^**I7**_p_ = π*_xz_^1^ π*_yz_^1^ σ*_z2_^1^ a_2u_^2^ ϕ_Sub_^0^. These are the iron(III) configurations that the resting state structure of the heme in P450 enzymes has, which means no further electron transfer to the heme can take place.^34,35,101^ The subsequent C–C bond cleavage of the substrate into two fragments (**TS8**_p_) has barriers of less than 10 kcal mol^–1^ on both spin state surfaces and generates an hydroxycumyl cation and deprotonated dihydroquinone. Optimized geometries of ^2,4^**TS8**_p_ are shown on the right-hand side of Figure 4. The imaginary frequency of both **TS8**_p_ structures represents the C–C stretch vibration and the splitting of the product into two fragments and has values of i215 (doublet) and i214 (quartet) cm^–1^. The C–C distance has elongated to 2.282 (doublet) and 2.277 (quartet) Å, while the oxygen atom is fully released from the iron center: Fe–O distances of 3.441 (doublet) and 3.506 (quartet) Å. After the C–C cleavage, the shallow minimum ^2,4^**I8**_p_ is reached. We then took the hydroxycumyl cation and deprotonated dihydroquinone groups from the **I8** structure and with the assistance of a water molecule investigated the pathway leading to final products. The reaction was found to be highly exothermic at Δ*G* = −41.6 kcal mol^–1^ with respect to **I8**, and as a consequence, no transition state can be characterized as the barrier was small. In principle, the OH rebound in intermediate ^4,2^**I6**_B_ can take place on the *para*-position as well as the *ortho*-position and lead to alternative products. We hypothesized that *ortho*-attack would lead to 3-hydroxy-BPA products as the aromatic pathway was found to be high in energy. Figure 5 shows the obtained energy landscape using the middle reaction pathway as described in Scheme 3 above. After phenolic hydrogen atom abstraction, the OH rebound takes place on the *ortho*-position with respect to the phenol via ^4,2^**TS7**_oB_. Free energies of activation with respect to **I6**_B_ of Δ*G* = 20.6 kcal mol^–1^ on the quartet spin state and Δ*G* = 31.2 kcal mol^–1^ on the doublet spin state are found. The ^4,2^**TS7**_oB_ transition state structures are shown in Figure 5. Both structures retain the electronic configuration of **I6**_B_, and hence, they are similar in structure. The C–O bond formation happens at a distance of 2.197 Å for both, and also the Fe–O and Fe–S distances are the same. The barrier is characterized with an imaginary frequency for the C–O stretch vibration and has a magnitude of i330 cm^–1^ for ^4^**TS7**_oB_ and i437 cm^–1^ for ^2^**TS7**_oB_. After the transition states, the structures relax to a highly stable **I7**_oB_ intermediate that is Δ*G* = −59.0 (doublet) and Δ*G* = −63.8 (quartet) kcal mol^–1^ more stable than reactants. After ^4,2^**I7**_oB_, we attempted direct proton transfer from the *ipso*-position to the phenol group either directly or via the *ortho*-phenol group; however, these pathways gave a high energy mechanism. We then attempted a proton-shuttle mechanism as proposed before for aromatic hydroxylation reactions, whereby the *ipso*-proton is initially transferred to one of the nitrogen atoms of the heme via barrier **TS10**. From the protonated heme structure **I4**, a proton relay via **TS5** gives the 3-hydroxy-BPA products. Optimized geometries of the proton-transfer barriers ^4,2^**TS10**_B_ are shown in Figure 5.

As little electronic change occurs on the doublet and quartets spin states, these proton-transfer barriers are very similar with a large imaginary frequency for proton shuttle from the *ipso*-carbon to the nitrogen atom: values of i971 cm^–1^ for ^4^**TS10**_B_ and i953 cm^–1^ for ^2^**TS10**_B_. These barriers are relatively central with long C–H distances (1.30 Å) and long N–H distances (1.38 Å). Energetically, these barriers are 20.4 kcal mol^–1^ on the doublet spin state and 25.5 kcal mol^–1^ on the quartet spin state with respect to the **I7** intermediates. The proton transfer intermediates via small and negligible barriers (via **TS5**) collapse to the 3-hydroxy-BPA product complexes with large exothermicity.

**Figure 6:**
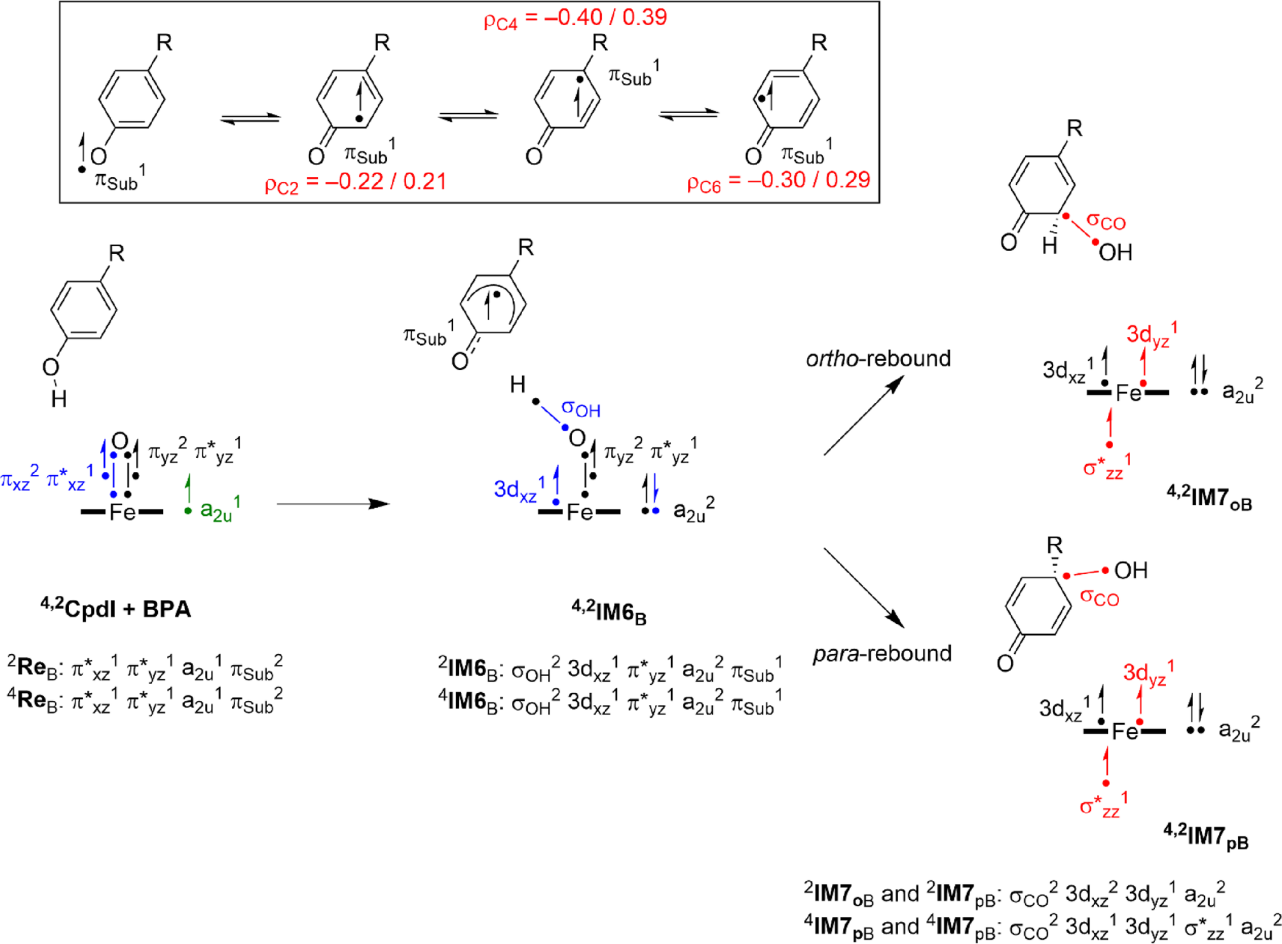
Valence bond description of the electron transfer and bond formation pathways for the reaction mechanism of BPA activation by CpdI through OH rebound to the *ortho*- or *para*-position of the phenol group. Dots represent electrons, and a line separated by two bonds is a bond orbital occupied by two electrons. The inset shows the various electromers of the phenoxy radical with spin density populations on the various atoms.

To understand the differences in product distributions for 3-hydroxy-BPA versus hydroxycumyl alcohol formation from the reaction of P450 CpdI with BPA, we analyzed the radical intermediates ^4,2^**I6**_B_ as they lead to the bifurcation channels to form the two products. In particular, from ^4,2^**I6**_B_, there is OH rebound to the *ortho*-position of substrate via ^4,2^**TS7**_oB_ for the pathway leading to 3-hydroxy-BPA as well as OH rebound to the *para*-position of the substrate via ^4,2^**TS7**_pB_ for the pathway leading to hydroxycumyl alcohol products. Thus, a comparison of the four **TS7** barrier heights from Figures 4 and 5 shows that *ortho*-OH rebound appears to have considerably higher barriers than *para*-OH rebound. Specifically, a comparison of the ^4,2^**TS7**_pB_ values in Figure 4 with those for ^4,2^**TS7**_oB_ in Figure 5 shows that the latter barriers are very high, i.e., Δ*G* = 31.2 and 20.6 kcal mol^–1^ for the doublet and quartet spin states, whereas the *para*-OH rebound barriers are Δ*G* = 17.3 and 11.5 kcal mol^–1^ for the two spin states, respectively. Consequently, for the P450 2C9 structure investigated here, we predict a dominant pathway for *para*-rebound leading to hydroxycumyl alcohol products with limited amount of 3-hydroxy-BPA forming. These relative barrier heights are not surprising, as considerably different radical characters are seen on the C_4_ and C_2_/C_6_ carbon atoms of the substrate in ^4,2^**I6**_B_, as shown in Figure 6. In particular, the *para*-carbon atom C_4_ has a spin of 0.39 in ^4^**I6**_B_ and −0.40 in ^2^**I6**_B_, whereas the spin densities on the *ortho*-carbon atoms C_2_ and C_6_ are only 0.21 and 0.29 in the quartet spin state and −0.22 and −0.30 in the doublet spin state, respectively. The higher radical character on C_4_ will push the reaction pathway to *para*-OH rebound favorably and indeed give the lowest rebound barriers. Interestingly, the radical character of an isolated BPA molecule with a hydrogen removed from the phenol group also gives a slightly larger spin on the C_4_ atom than on the C_2_/C_6_ atoms. Therefore, the protein does not change the spin distributions dramatically and keeps the preference of the C_4_ radical over the C_2_ or C_6_ radical.

To further explain the bond-formation and bond-cleavage patterns in the bifurcation pathways from ^4,2^**I6**_B_, we give a valence bond description of the bonds that are broken and formed in the process in Figure 6. As described above, CpdI has close-lying doublet and quartet spin states with orbital occupation π*_xz_^1^ π*_yz_^1^ a_2u_^1^ π_Sub,_^2^ whereby the π_Sub_ orbital represents a substrate orbital smeared over the aromatic ring. Hydrogen atom abstraction from the phenol group leads to a radical intermediate ^4,2^**I6**_B_, where the proton is abstracted by the oxo group and the electron is transferred to the heme into the a_2u_ orbital that becomes doubly occupied. This process leads to the formation of a σ_OH_ orbital for the O–H interaction and leaves a radical on the substrate in π_Sub_. This radical as shown at the top image of Figure 6 is distributed over the aromatic ring, and four electromeric states can be drawn, which will be in equilibrium. The spin density associated with single occupation of the π_Sub_ orbital gives a dominant spin on the C_4_ atom (*para* to the OH group), and consequently, this will be the dominant target place for the OH rebound. Indeed, the ^4,2^**TS7**_pB_ barriers are the lowest in energy starting from ^4,2^**I6**_B_. A comparison of the electronic states for ^4,2^**IM7**_pB_ versus ^4,2^**IM7**_oB_ local intermediates shows that they have the same electronic configuration in each spin state. Thus, the metal is in the iron(III) oxidation state with δ_x_2_-y_2^2^ 3d_xz_^2^ 3d_yz_^1^ σ*_zz_^1^ configuration in the quartet spin state. Note that OH rebound leads to cleavage of the Fe–O orbital interactions and the π*_xz_ and π*_yz_ orbitals change back to atomic iron 3d orbitals in this step. Overall, the metal-heme is in the same oxidation state with the same orbital occupation in **IM7**_oB_ as in **IM7**_pB_. Indeed, energetically, the **IM7**_oB_ and **IM7**_pB_ structures show similar driving forces and, based on the Bell–Evans–Polanyi principle, equal product distributions would be expected. In our system, however, the barriers for *ortho*-OH rebound are considerably higher than for *para*-OH rebound and, therefore, second-coordination sphere effects of the protein and substrate-binding pocket influence the bifurcation pathways and product distributions dramatically. Recent works of ours highlighted that second-coordination sphere effects in proteins indeed can influence bifurcation pathways and lead to negative catalysis, where a lesser exothermic pathway is preferred over a more exothermic reaction.^102,103^

## Conclusions

In this work, a detailed computational study is presented on the activation of BPA by the P450 isozyme 2C9. A large active site cluster model was created that includes the first- and second-coordination spheres of the heme and substrate. Our optimized structures compare well to previously reported data (computational and spectroscopic) on analogous P450 systems. We tested various pathways for substrate activation by P450 CpdI on the doublet and quartet spin states. We show that aliphatic hydroxylation of the BPA methyl groups is difficult in this particular protein due to the size and shape of the substrate-binding pocket. Much lower energy barriers were found for phenol hydrogen atom abstraction leading to a radical intermediate, where the spin is distributed over the C_2_, C_4_, C_6_, and phenolic oxygen atoms. As the spin is largest on the C_4_ carbon atom, the lowest energy rebound is on the C_4_ position and leads to dominant hydroxycumyl alcohol product complexes. Our studies show that P450 catalysis is dependent on the P450 isozyme, where the second coordination sphere determines the reactivity and product distributions.

## Supporting Information Available

Tables with energies (absolute and relative), group spin densities and charges and Cartesian coordinates of optimized geometries (PDF) XYZ coordinates of optimized structures (ZIP)
