# Supplementary material for: Biotransformation of Bisphenol by Human Cytochrome P450 2C9 Enzymes: A Density Functional Theory Study
Source: Inorg Chem. 2023 Jan 18;62(5):2244–56. doi: 10.1021/acs.inorgchem.2c03984 (PMC9923688; doi:10.1021/acs.inorgchem.2c03984)
Supplement: Supplementary file 1 — ic2c03984_si_001.pdf [file ic2c03984_si_001.pdf]

Supporting Information

Biotransformation of Bisphenol by Human Cytochrome P450 2C9 Enzymes: a Density Functional Theory Study.

Artur Hermano Sampaio Dias,[§‡] Rolly Yadav,[#] Thirakorn Mokkawes,[§] Asheesh Kumar,[#] Munir S. Skaf,[‡] Chivukula V. Sastri,*[⊥] Devesh Kumar,*[&] and Sam P. de Visser*[§]

[§] Manchester Institute of Biotechnology and Department of Chemical Engineering, The University of Manchester, 131 Princess Street, Manchester M1 7DN, United Kingdom

[‡] Center for Computing in Engineering & Sciences, University of Campinas, Rua Josué de Castro, s/n, Campinas, 13083-861, Brazil

[#] Department of Physics, Babasaheb Bhimrao Ambedkar University, Lucknow, Uttar Pradesh (U.P.) 226025, India

[⊥] Department of Chemistry, Indian Institute of Technology Guwahati, Guwahati, Assam 781039, India

[&] Department of Physics, Siddharth University, Kapilvastu, Siddharthnagar, 272202, India

Email: sam.devisser@manchester.ac.uk

Methods and approaches.

Calculations were run in Gaussian-09 as specified in the Methods section of the Main Paper with the following keywords:

UB3LYP genecp test gfinput gfprint scf(novaracc,xqc) guess=read geom=(check,newdefinition) uB3LYP genecp test gfinput gfprint scf(novaracc,xqc) guess=read geom=(check,newdefinition) scfconv=7 pop=full nosymm integral=(acc2e=12) opt(loose,modredundant) freq=noraman Temperature=298.15pop=full nosymm integral=(acc2e=12) opt(modredundant) freq=noraman Temperature=298.15

Table S1: Absolute (in au) energies and free energies of UB3LYP/BS1 optimized geometries in Gaussian-09.

	E [BS1, au]	ZPE	G [au]	E [BS2, au]
^{2}**Re**$_{BPA}$	-5938.05316	1.83661	-5936.40460	-5934.43407
^{4}**Re**$_{BPA}$	-5938.05273	1.83671	-5936.40484	-5934.43192
^{2}**TS3**$_{B}$	-5938.01409	1.83536	-5936.36188	-5934.41173
^{4}**TS3**$_{B}$	-5938.01042	1.83505	-5936.35748	-5934.41036
^{4}**I3**$_{B}$	-5938.02871	1.83618	-5936.37561	-5934.43184
^{4}**PB**	-5938.06229	1.83811	-5936.40786	-5934.49850
^{2}**TS4**$_{B}$	-5938.06926	1.83409	-5936.41072	-5934.49787
^{2}**I4**$_{B}$	-5938.12263	1.84108	-5936.45621	-5934.51298
^{4}**I4**$_{B}$	-5938.11675	1.84023	-5936.45222	-5934.54637
^{2}**PC**	-5938.14918	1.84187	-5936.48385	-5934.56859
^{4}**PC**	-5938.13383	1.83859	-5936.47411	-5934.59882
^{2}**TS6**$_{B}$	-5938.04620	1.83126	-5936.39559	-5934.42039
^{4}**TS6**$_{B}$	-5938.04583	1.83132	-5936.39574	-5934.41788
^{2}**I6**$_{B}$	-5938.05460	1.83541	-5936.40402	-5934.49183
^{4}**I6**$_{B}$	-5938.05807	1.83593	-5936.41040	-5934.47339
^{2}**TS7**$_{oB}$	-5938.04209	1.83503	-5936.38727	-5934.44631
^{4}**TS7**$_{oB}$	-5938.03893	1.83493	-5936.38441	-5934.43263
^{2}**I7**$_{oB}$	-5938.10164	1.83912	-5936.44874	-5934.53246
^{4}**I7**$_{oB}$	-5938.09890	1.83777	-5936.44902	-5934.53700
^{2}**TS10**$_{B}$	-5938.07029	1.83445	-5936.41604	-5934.50129
^{4}**TS10**$_{B}$	-5938.04507	1.83306	-5936.39374	-5934.49781
^{2}**TS7**$_{pB}$	-5938.04746	1.83423	-5936.39520	-5934.46592
^{4}**TS7**$_{pB}$	-5938.03880	1.83427	-5936.38729	-5934.45895
^{2}**I7**$_{pB}$	-5938.09635	1.83849	-5936.44130	-5934.53442
^{4}**I7**$_{pB}$	-5938.09553	1.83777	-5936.44512	-5934.53749
^{2}**TS8**$_{B}$	-5938.07680	1.83602	-5936.42397	-5934.51655
^{4}**TS8**$_{B}$	-5938.07570	1.83549	-5936.42518	-5934.52401
^{2}**I8**$_{B}$	-5938.08074	1.83564	-5936.42898	-5934.52127
^{4}**I8**$_{B}$	-5938.06646	1.83285	-5936.42154	-5934.54564

Table S2: Relative (in kcal mol^{-1}) energies and free energies of UB3LYP/BS1 optimized geometries in Gaussian-09.

	ΔE [BS1]	ΔE+ZPE	ΔG [BS1]	ΔE [BS2]	ΔE[BS2]+ZPE	ΔG [BS2]
^{2}**Re**$_{BPA}$	0.00	0.00	0.00	0.00	0.00	0.00
^{4}**Re**$_{BPA}$	0.27	0.33	-0.15	1.35	1.41	0.93
^{2}**TS3**$_{B}$	24.51	23.73	26.81	14.02	13.24	16.31
^{4}**TS3**$_{B}$	26.82	25.84	29.57	14.88	13.90	17.63
^{4}**I3**$_{B}$	15.34	15.07	18.19	1.40	1.13	4.25
^{4}**PB**	-5.73	-4.79	-2.05	-40.43	-39.49	-36.74
^{2}**TS4**$_{B}$	-10.10	-11.69	-3.84	-40.03	-41.62	-33.77
^{2}**I4**$_{B}$	-43.59	-40.79	-32.39	-49.52	-46.71	-38.31
^{4}**I4**$_{B}$	-39.91	-37.63	-29.89	-70.47	-68.20	-60.45
^{2}**PC**	-60.25	-56.95	-49.73	-84.41	-81.11	-73.89
^{4}**PC**	-50.62	-49.38	-43.62	-103.38	-102.14	-96.38
^{2}**TS6**$_{B}$	4.36	1.01	5.65	8.58	5.23	9.87
^{4}**TS6**$_{B}$	4.60	1.28	5.56	10.16	6.84	11.12
^{2}**I6**$_{B}$	-0.90	-1.66	0.36	-36.25	-37.00	-34.98
^{4}**I6**$_{B}$	-3.08	-3.51	-3.64	-24.67	-25.10	-25.23
^{2}**TS7**$_{oB}$	6.94	5.95	10.88	-7.68	-8.67	-3.75
^{4}**TS7**$_{oB}$	8.93	7.88	12.67	0.90	-0.15	4.64
^{2}**I7**$_{oB}$	-30.42	-28.85	-27.70	-61.74	-60.17	-59.02
^{4}**I7**$_{oB}$	-28.70	-27.98	-27.88	-64.59	-63.86	-63.76
^{2}**TS10**$_{B}$	-10.75	-12.11	-7.18	-42.18	-43.54	-38.61
^{4}**TS10**$_{B}$	5.08	2.85	6.81	-40.00	-42.22	-38.26
^{2}**TS7**$_{pB}$	3.58	2.09	5.90	-19.99	-21.48	-17.67
^{4}**TS7**$_{pB}$	9.01	7.54	10.86	-15.61	-17.08	-13.75
^{2}**I7**$_{pB}$	-27.10	-25.93	-23.03	-62.97	-61.79	-58.90
^{4}**I7**$_{pB}$	-26.59	-25.86	-25.43	-64.90	-64.16	-63.73
^{2}**TS8**$_{B}$	-14.83	-15.20	-12.16	-51.76	-52.13	-49.08
^{4}**TS8**$_{B}$	-14.15	-14.85	-12.92	-56.44	-57.14	-55.21
^{2}**I8**$_{B}$	-17.31	-17.92	-15.30	-54.72	-55.33	-52.71
^{4}**I8**$_{B}$	-8.34	-10.71	-10.63	-70.01	-72.37	-72.29

Table S3: Group charges (part a) and group spin densities (part b) of UB3LYP/BS1 optimized geometries in Gaussian-09.

(a) Group charges.

	Fe	O	Por	Axial	SubH	Protein	Total
^{2}**Re**$_{BPA}$	0.521	-0.501	-0.033	-0.032	-0.004	0.049	0.000
^{4}**Re**$_{BPA}$	0.513	-0.495	-0.039	-0.024	-0.003	0.049	0.000
^{2}**TS3**$_{B}$	0.357	-0.528	-0.244	0.067	0.323	0.026	0.000
^{4}**TS3**$_{B}$	0.432	-0.536	-0.275	0.084	0.271	0.024	0.000
^{4}**I3**$_{B}$	0.326	-0.607	-0.181	0.137	0.305	0.020	0.000
^{4}**PB**	0.444	-0.448	-0.384	-0.158	0.478	0.067	0.000
^{2}**TS4**$_{B}$	0.171	-0.625	-0.228	0.049	0.635	-0.002	0.000
^{2}**I4**$_{B}$	0.189	-0.687	-0.223	-0.020	0.642	0.098	0.000
^{4}**I4**$_{B}$	0.366	-0.703	-0.307	-0.118	0.663	0.099	0.000
^{2}**PC**	0.267	-0.701	-0.346	-0.013	0.691	0.103	0.000
^{4}**PC**	0.480	-0.702	-0.415	-0.146	0.683	0.100	0.000
^{2}**TS6**$_{B}$	0.395	-0.674	0.214	-0.054	0.156	-0.037	0.000
^{4}**TS6**$_{B}$	0.381	-0.669	0.200	-0.041	0.164	-0.037	0.000
^{2}**I6**$_{B}$	0.384	-0.698	-0.249	-0.011	0.542	0.032	0.000
^{4}**I6**$_{B}$	0.337	-0.717	-0.234	0.088	0.559	-0.033	0.000
^{2}**TS7**$_{oB}$	0.306	-0.646	-0.268	0.044	0.525	0.039	0.000
^{4}**TS7**$_{oB}$	0.338	-0.663	-0.224	0.045	0.467	0.038	0.000
^{2}**I7**$_{oB}$	0.178	-0.613	-0.434	0.025	0.833	0.011	0.000
^{4}**I7**$_{oB}$	0.408	-0.613	-0.425	-0.182	0.786	0.025	0.000
^{2}**TS10**$_{B}$	0.188	-0.618	-0.296	0.022	0.695	0.009	0.000
^{4}**TS10**$_{B}$	0.348	-0.619	-0.492	0.055	0.699	0.009	0.000
^{2}**TS7**$_{pB}$	0.265	-0.674	-0.164	-0.031	0.574	0.030	0.000
^{4}**TS7**$_{pB}$	0.376	-0.662	-0.281	-0.044	0.583	0.029	0.000
^{2}**I7**$_{pB}$	0.221	-0.640	-0.403	0.032	0.767	0.023	0.000
^{4}**I7**$_{pB}$	0.424	-0.642	-0.401	-0.165	0.760	0.024	0.000
^{2}**TS8**$_{B}$	0.219	-0.665	-0.354	0.027	0.798	-0.025	0.000
^{4}**TS8**$_{B}$	0.407	-0.662	-0.362	-0.151	0.792	-0.023	0.000
^{2}**I8**$_{B}$	0.231	-0.666	-0.350	0.018	0.813	-0.046	0.000
^{4}**I8**$_{B}$	0.440	-0.666	-0.487	-0.043	0.804	-0.048	0.000

(b) Group spin densities.

	Fe	O	Por	Axial	SubH	Prot	Total
^{2}**Re**$_{BPA}$	1.374	0.738	-0.461	-0.656	0.006	0.000	1.000
^{4}**Re**$_{BPA}$	1.238	0.775	0.367	0.614	0.006	0.000	3.000
^{2}**TS3**$_{B}$	1.089	0.502	-0.360	-0.558	0.329	-0.001	1.000
^{4}**TS3**$_{B}$	0.951	0.575	0.266	0.705	0.504	-0.001	3.000
^{4}**I3**$_{B}$	0.943	0.129	0.370	0.591	0.968	-0.002	3.000
^{4}**PB**	2.474	0.000	0.065	0.464	-0.003	0.000	3.000
^{2}**TS4**$_{B}$	1.076	-0.001	-0.082	0.009	-0.003	0.000	1.000
^{2}**I4**$_{B}$	1.054	-0.016	-0.068	0.039	-0.010	0.000	1.000
^{4}**I4**$_{B}$	2.810	0.038	0.068	0.090	-0.007	0.000	3.000
^{2}**PC**	1.234	0.000	-0.148	-0.085	-0.001	0.000	1.000
^{4}**PC**	3.486	0.000	-0.313	-0.169	-0.003	0.000	3.000
^{2}**TS6**$_{B}$	1.674	0.463	-0.713	-0.160	-0.262	-0.002	1.000
^{4}**TS6**$_{B}$	1.536	0.503	0.533	0.110	0.315	0.003	3.000
^{2}**I6**$_{B}$	1.860	0.272	-0.127	-0.016	-0.989	-0.001	1.000
^{4}**I6**$_{B}$	1.627	0.227	-0.075	0.240	0.982	-0.001	3.000
^{2}**TS7**$_{oB}$	1.798	0.192	-0.115	-0.118	-0.759	0.002	1.000
^{4}**TS7**$_{oB}$	2.065	0.115	-0.101	0.064	0.858	-0.002	3.000
^{2}**I7**$_{oB}$	1.114	-0.001	-0.085	-0.029	0.001	0.000	1.000
^{4}**I7**$_{oB}$	2.510	0.010	0.017	0.466	-0.003	0.000	3.000
^{2}**TS10**$_{B}$	1.077	0.000	-0.082	0.007	-0.001	0.000	1.000
^{4}**TS10**$_{B}$	3.074	0.004	0.147	-0.212	-0.013	0.000	3.000
^{2}**TS7**$_{pB}$	1.175	-0.062	-0.505	-0.217	0.610	-0.002	1.000
^{4}**TS7**$_{pB}$	2.282	-0.061	-0.067	0.239	0.609	-0.002	3.000
^{2}**I7**$_{pB}$	1.263	0.000	-0.135	-0.129	0.000	0.000	1.000
^{4}**I7**$_{pB}$	2.470	0.001	0.042	0.489	-0.002	0.000	3.000
^{2}**TS8**$_{B}$	1.257	0.000	-0.138	-0.118	-0.001	0.000	1.000
^{4}**TS8**$_{B}$	2.447	0.000	0.060	0.496	-0.004	0.000	3.000
^{2}**I8**$_{B}$	1.255	0.000	-0.137	-0.117	-0.001	0.000	1.000
^{4}**I8**$_{B}$	3.408	0.000	-0.125	-0.278	-0.004	0.000	3.000

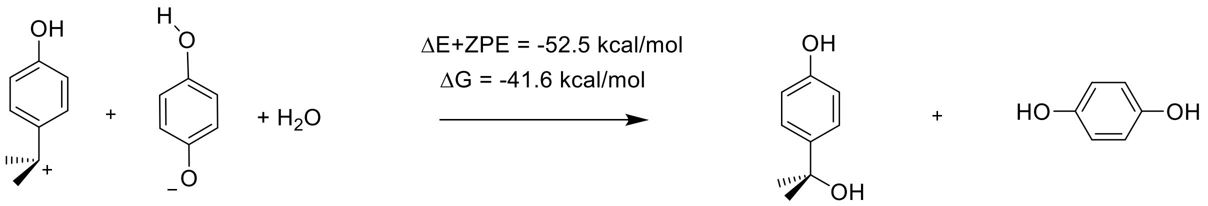

Figure S1: UB3LYP/BS2 calculated reaction energies for water addition and form hydroxycumyl alcohol products.

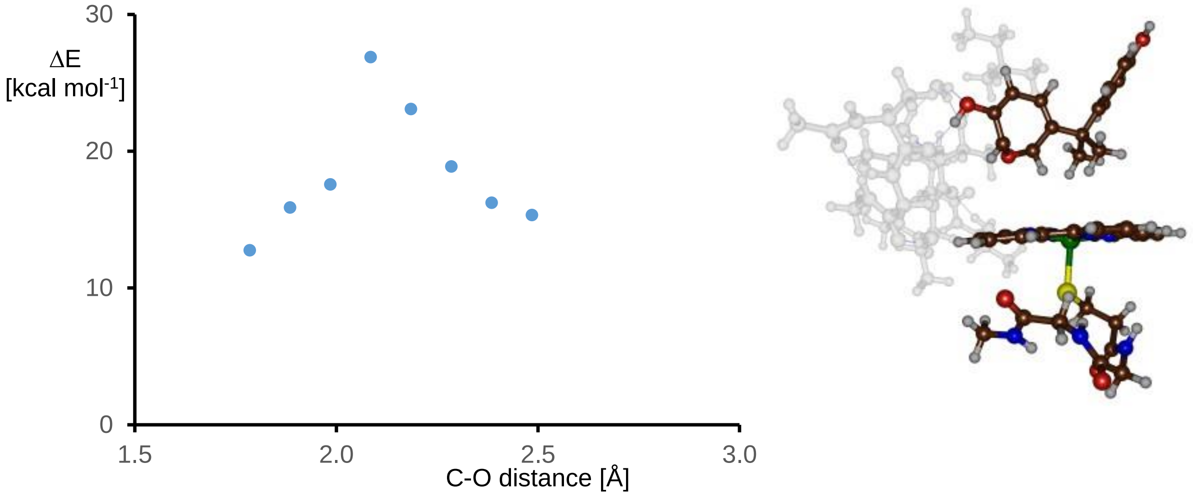

Figure S2: UB3LYP/BS1 geometry scan for epoxide formation from ^{4}I3$_B$ as calculated in Gaussian-09. Each datapoint represents a full geometry optimization with fixed C-O distance. As can be seen the maximum energy (with respect to ^{2}Re$_{BPA}$) is 27 kcal/mol, consequently this is an unviable pathway. Also shown is the optimized geometry of the epoxide product complex.

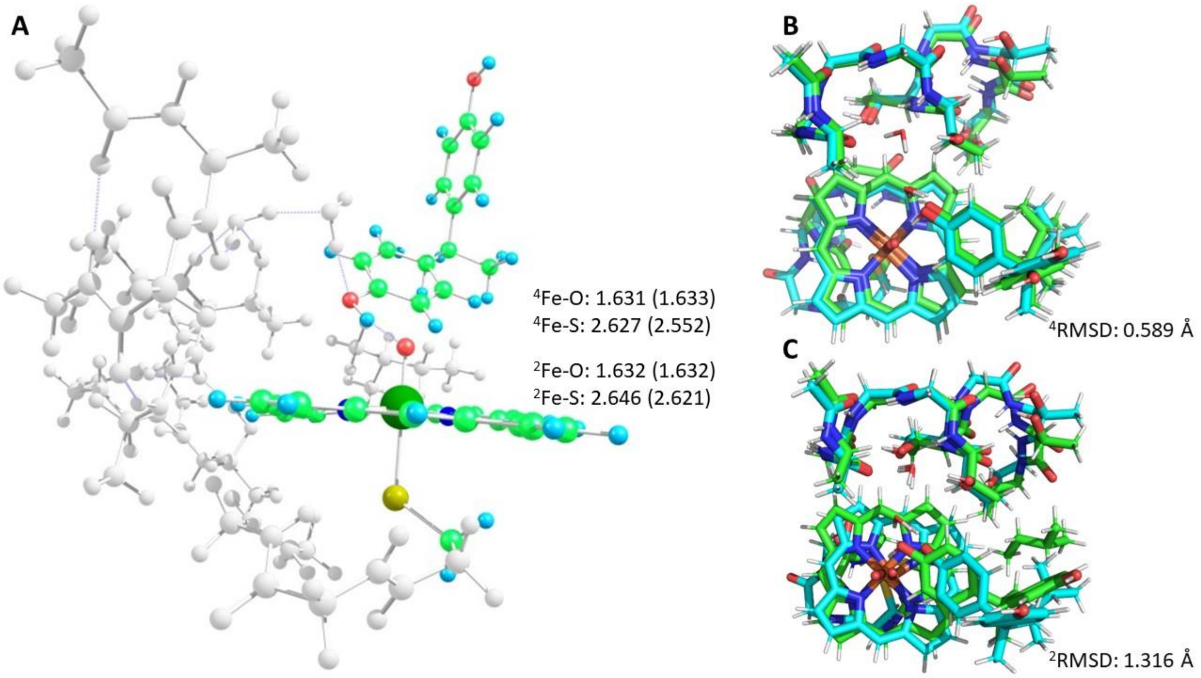

Figure S3. Optimized geometries of the reactant complexes in the doublet and quartet spin states for model B as calculated with B3LYP and B3LYP-D3. (A) Bond lengths in Ångströms are shown outside of parentheses for B3LYP models and inside parentheses for B3LYP-D3. (B) Structural alignment between quartet reactants with and without dispersion effects. Green structures obtained with B3LYP-D3, while cyan is B3LYP. (C) Same as part (B), but for doublet spin models.

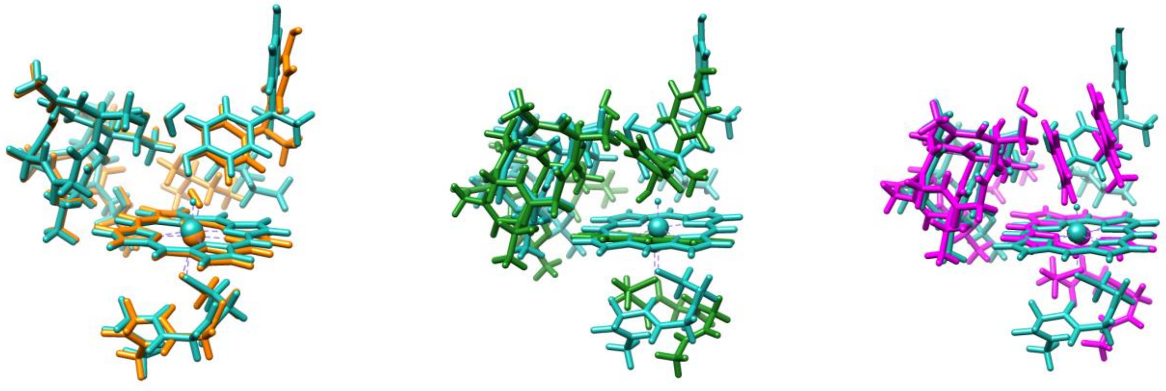

Overlay $^{2}Re_{B}$ vs $^{2}I6_{B}$ **Overlay $^{2}Re_{B}$ vs $^{2}I7p_{B}$** **Overlay $^{2}Re_{B}$ vs $^{2}I8_{B}$**

Figure S4. Overlays of optimized geometries versus the reactant structure in the doublet spin state. As can be seen little changes to the protein structure has occurred during the reaction mechanism.

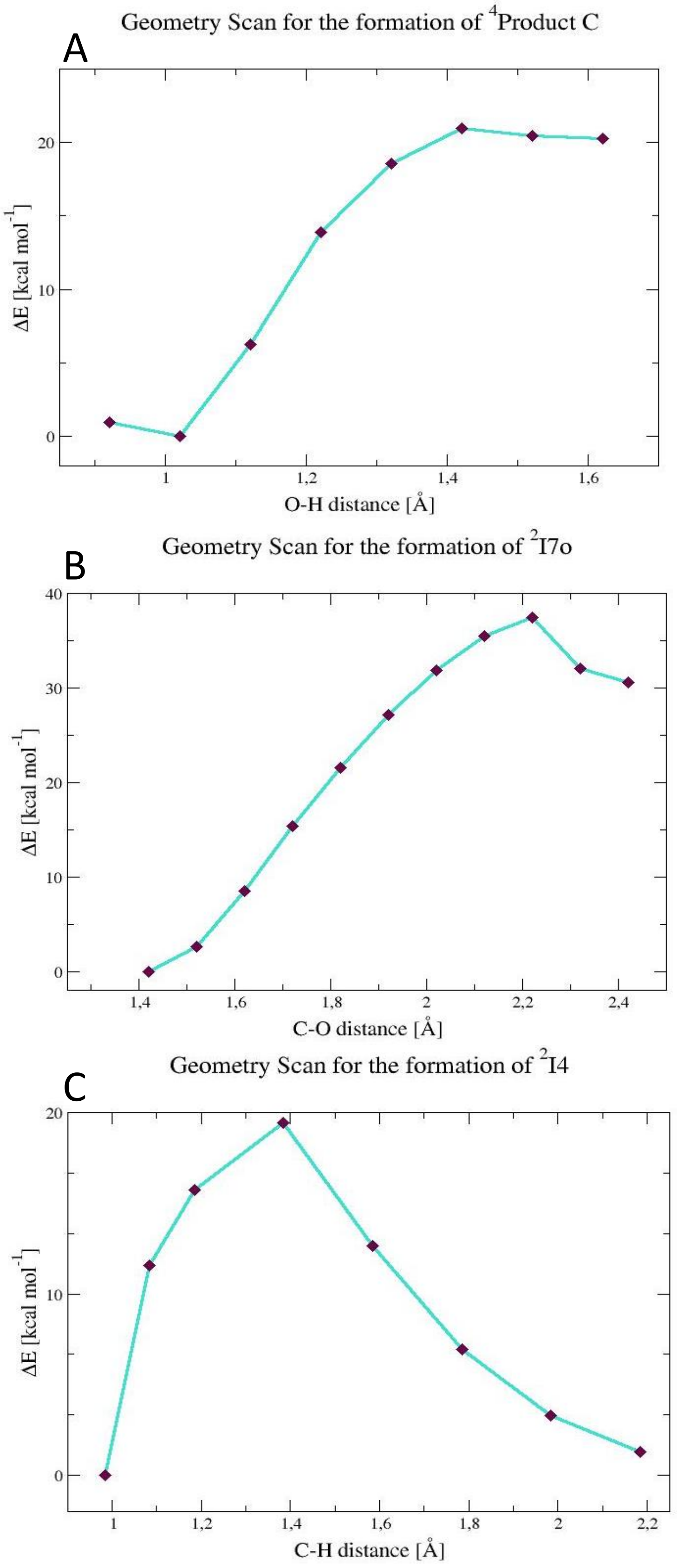

Figure S5: UB3LYP/BS1 geometry scans, with each datapoint representing a full geometry optimization with fixed reaction coordinate distances. (A) From right to left, this scan highlights the small energy barrier for the formation of ^{4}product C from ^{4}I4. (B) The formation of ^{2}I7o from ^{2}I6 is highly favourable, with ΔE of -40 kcal/mol. (C) This scan represents the formation of ^{2}I4 from ^{2}I7o intermediate.

Cartesian Coordinates of optimized geometries.

^{2}Re$_{BPA}$

C	0.572925000000	-0.822005000000	0.923705000000
C	0.257776000000	-1.058272000000	2.395316000000
O	1.073068000000	-1.573917000000	3.160257000000
C	0.895145000000	-2.140612000000	0.201180000000
H	-0.243170000000	-0.300445000000	0.416165000000
H	1.759645000000	-2.606755000000	0.689757000000
H	1.153189000000	-1.951548000000	-0.843771000000
N	-0.989414000000	-0.685158000000	2.802104000000
C	-1.425409000000	-0.835042000000	4.179339000000
C	-1.772950000000	-2.252705000000	4.667994000000
O	-2.117004000000	-2.404983000000	5.833741000000
H	-1.653458000000	-0.350996000000	2.115582000000
H	-0.632257000000	-0.482337000000	4.845395000000
N	-1.651095000000	-3.262766000000	3.763079000000
C	-1.692190000000	-4.655915000000	4.165586000000
C	-0.330701000000	-5.364588000000	4.284531000000
O	-0.307474000000	-6.575794000000	4.481125000000
H	-1.353287000000	-3.055219000000	2.815230000000
H	-2.295101000000	-5.239801000000	3.463465000000
H	-2.174859000000	-4.700744000000	5.145158000000
N	0.773230000000	-4.581643000000	4.168886000000
C	2.112695000000	-5.124851000000	4.297956000000
H	0.676229000000	-3.584825000000	4.009657000000
H	2.738754000000	-4.803108000000	3.458577000000
H	2.588284000000	-4.800829000000	5.232450000000
H	2.033631000000	-6.213098000000	4.300081000000
H	-2.299375000000	-0.199433000000	4.344394000000
H	1.459692000000	-0.176536000000	0.877492000000
S	-0.425560000000	-3.405400000000	0.307500000000
Fe	-2.187824000000	-3.423227000000	-1.666049000000
C	-1.913770000000	-0.011259000000	-1.878195000000
C	0.205361000000	-3.759640000000	-4.082752000000
C	-2.270386000000	-6.817870000000	-1.274381000000
C	-4.284914000000	-3.068073000000	1.029118000000
N	-1.049886000000	-2.145555000000	-2.730038000000
C	-1.138826000000	-0.772921000000	-2.741965000000
C	-0.283577000000	-0.223459000000	-3.763553000000
C	0.325253000000	-1.277472000000	-4.373833000000
C	-0.165885000000	-2.469255000000	-3.730087000000
N	-1.224238000000	-4.968192000000	-2.498532000000
C	-0.286294000000	-4.917747000000	-3.499816000000
C	0.140592000000	-6.247380000000	-3.859808000000
C	-0.550387000000	-7.111969000000	-3.070392000000
C	-1.404196000000	-6.303500000000	-2.229991000000
N	-3.082465000000	-4.681066000000	-0.376144000000
C	-3.049556000000	-6.052229000000	-0.415733000000
C	-3.920330000000	-6.608255000000	0.591312000000
C	-4.482759000000	-5.552651000000	1.242932000000
C	-3.958105000000	-4.356066000000	0.631998000000
N	-2.881276000000	-1.854396000000	-0.577692000000
C	-3.778740000000	-1.906001000000	0.465867000000
C	-4.139950000000	-0.574823000000	0.879028000000
C	-3.478062000000	0.288646000000	0.054079000000

C	-2.709940000000	-0.516705000000	-0.860312000000
H	-1.882906000000	1.067284000000	-1.999957000000
H	0.929184000000	-3.871526000000	-4.883544000000
H	-2.360282000000	-7.896201000000	-1.185695000000
H	-4.987649000000	-2.961155000000	1.849483000000
H	-0.460842000000	-8.191756000000	-3.051754000000
H	0.878958000000	-6.479293000000	-4.615905000000
H	-4.064323000000	-7.666351000000	0.761603000000
H	-5.175885000000	-5.563542000000	2.074620000000
H	1.039204000000	-1.268732000000	-5.187234000000
H	-0.170522000000	0.833350000000	-3.969101000000
H	-3.513735000000	1.370796000000	0.042297000000
H	-4.829645000000	-0.345135000000	1.681587000000
O	-3.437915000000	-3.393099000000	-2.714384000000
C	-3.312395000000	-2.710980000000	-6.033531000000
C	-5.239096000000	-1.514977000000	-10.355752000000
C	-3.170851000000	-2.625270000000	-8.474014000000
C	-2.993018000000	-2.036181000000	-7.216284000000
C	-3.810101000000	-4.013413000000	-6.094813000000
C	-3.993101000000	-4.632712000000	-7.338586000000
C	-3.679279000000	-3.936161000000	-8.502198000000
H	-1.739731000000	-3.697020000000	-10.596253000000
H	-1.173832000000	-2.155273000000	-11.273152000000
C	-2.413238000000	-0.442774000000	-9.590549000000
C	-1.540086000000	-2.646125000000	-10.363956000000
C	-2.789872000000	-1.929948000000	-9.797007000000
C	-3.982021000000	-1.978496000000	-10.774802000000
C	-3.874931000000	-2.422838000000	-12.100149000000
C	-4.963977000000	-2.406596000000	-12.972351000000
C	-6.201698000000	-1.934749000000	-12.532997000000
C	-6.335645000000	-1.487487000000	-11.213781000000
H	-2.928380000000	-2.795237000000	-12.476772000000
H	-4.866828000000	-2.757788000000	-13.994960000000
O	-7.240275000000	-1.936546000000	-13.425015000000
H	-7.297866000000	-1.121998000000	-10.857229000000
H	-5.368266000000	-1.177967000000	-9.330545000000
O	-4.170503000000	-4.723488000000	-4.972044000000
H	-3.180403000000	-2.228547000000	-5.069378000000
H	-4.345339000000	-5.660872000000	-7.374038000000
H	-3.836157000000	-4.425451000000	-9.459963000000
H	-3.886484000000	-4.245498000000	-4.150994000000
H	-2.603368000000	-1.026887000000	-7.134466000000
H	-8.029247000000	-1.580444000000	-12.987843000000
H	-0.735275000000	-2.617653000000	-9.621714000000
H	-3.229674000000	0.126847000000	-9.134498000000
H	-1.524110000000	-0.337568000000	-8.957648000000
H	-2.189373000000	0.015908000000	-10.559257000000
C	-12.384090000000	-9.550167000000	-2.485416000000
C	-10.950111000000	-9.078541000000	-2.612015000000
O	-10.005688000000	-9.875832000000	-2.643891000000
H	-12.677094000000	-10.035702000000	-3.422937000000
H	-12.445064000000	-10.304504000000	-1.696481000000
N	-10.749098000000	-7.731972000000	-2.674792000000
C	-9.457785000000	-7.162840000000	-3.083556000000
C	-8.339244000000	-7.440555000000	-2.070009000000
O	-7.159910000000	-7.477056000000	-2.424391000000
C	-9.598656000000	-5.655615000000	-3.334313000000

H	-11.561313000000	-7.134243000000	-2.760426000000
H	-9.124130000000	-7.650966000000	-4.005679000000
H	-10.380522000000	-5.469952000000	-4.079971000000
H	-9.853195000000	-5.117477000000	-2.413942000000
H	-8.662435000000	-5.257797000000	-3.734758000000
N	-8.687383000000	-7.628116000000	-0.771906000000
C	-7.671963000000	-7.985806000000	0.204735000000
C	-7.069100000000	-9.390296000000	0.031720000000
O	-6.006830000000	-9.658140000000	0.600216000000
H	-9.665422000000	-7.614598000000	-0.520095000000
H	-8.107356000000	-7.921665000000	1.206434000000
H	-6.837450000000	-7.282577000000	0.151449000000
N	-7.788687000000	-10.273769000000	-0.695678000000
C	-7.345679000000	-11.638279000000	-0.922675000000
C	-6.777251000000	-11.955948000000	-2.317300000000
O	-5.824534000000	-12.726528000000	-2.420455000000
H	-8.630607000000	-9.959210000000	-1.168089000000
H	-6.563550000000	-11.866152000000	-0.199538000000
N	-7.409448000000	-11.419439000000	-3.393350000000
C	-7.104424000000	-11.896280000000	-4.742319000000
C	-5.656938000000	-11.634896000000	-5.192519000000
O	-4.911107000000	-12.562929000000	-5.498333000000
H	-8.231132000000	-10.839834000000	-3.261331000000
H	-7.247575000000	-12.978456000000	-4.809163000000
N	-5.257819000000	-10.333905000000	-5.202903000000
C	-3.898311000000	-10.028341000000	-5.642158000000
C	-2.880631000000	-10.636934000000	-4.670372000000
O	-1.793806000000	-11.059609000000	-5.093023000000
C	-3.694013000000	-8.497453000000	-5.832395000000
O	-4.676590000000	-8.002498000000	-6.729736000000
C	-2.326891000000	-8.179974000000	-6.427737000000
H	-5.955683000000	-9.593322000000	-5.198781000000
H	-3.706545000000	-10.512827000000	-6.605180000000
H	-3.792129000000	-8.002665000000	-4.852526000000
H	-5.511357000000	-7.904807000000	-6.227494000000
H	-2.220203000000	-7.095299000000	-6.514797000000
H	-2.247264000000	-8.617866000000	-7.428435000000
H	-1.508800000000	-8.569024000000	-5.817759000000
N	-3.218076000000	-10.688413000000	-3.367103000000
C	-2.336313000000	-11.231781000000	-2.338359000000
C	-1.922069000000	-12.691569000000	-2.625934000000
O	-0.773565000000	-13.074150000000	-2.392442000000
C	-3.054296000000	-11.137197000000	-0.970203000000
O	-3.664748000000	-9.843232000000	-0.920485000000
C	-2.091962000000	-11.349774000000	0.195328000000
H	-4.062384000000	-10.233217000000	-3.042220000000
H	-1.398941000000	-10.665584000000	-2.314888000000
H	-3.841829000000	-11.902598000000	-0.953761000000
H	-4.409233000000	-9.868056000000	-0.286087000000
H	-2.638972000000	-11.324423000000	1.144045000000
H	-1.575547000000	-12.311518000000	0.116026000000
H	-1.338030000000	-10.555545000000	0.213068000000
N	-2.886867000000	-13.505152000000	-3.118950000000
C	-2.597043000000	-14.880913000000	-3.491338000000
C	-1.787584000000	-15.079013000000	-4.790674000000
O	-1.434246000000	-16.204615000000	-5.116202000000
H	-3.820023000000	-13.142971000000	-3.290240000000

H	-2.022846000000	-15.366105000000	-2.697225000000
N	-1.503106000000	-13.939180000000	-5.473676000000
C	-0.691490000000	-13.879092000000	-6.674163000000
C	0.600316000000	-13.054738000000	-6.443931000000
O	1.270848000000	-12.644043000000	-7.388964000000
C	-1.521169000000	-13.289177000000	-7.866954000000
O	-2.859455000000	-13.764028000000	-7.843504000000
C	-0.946748000000	-13.685321000000	-9.221179000000
H	-1.844219000000	-13.053034000000	-5.120655000000
H	-0.411699000000	-14.911862000000	-6.908362000000
H	-1.504821000000	-12.192343000000	-7.767800000000
H	-3.281031000000	-13.473907000000	-7.017102000000
H	-1.551035000000	-13.243245000000	-10.020680000000
H	-0.982774000000	-14.775647000000	-9.332943000000
H	0.086299000000	-13.345786000000	-9.308018000000
N	0.926016000000	-12.804938000000	-5.143303000000
C	2.054341000000	-11.969559000000	-4.762689000000
C	1.682974000000	-10.988931000000	-3.637577000000
O	0.654990000000	-10.082453000000	-4.043975000000
C	2.884643000000	-10.149149000000	-3.219569000000
H	0.360715000000	-13.206819000000	-4.403799000000
H	2.368187000000	-11.426741000000	-5.658477000000
H	1.330181000000	-11.574712000000	-2.774738000000
H	-0.072353000000	-10.595865000000	-4.448226000000
H	2.597286000000	-9.444843000000	-2.431991000000
H	3.693069000000	-10.782088000000	-2.837220000000
H	3.263522000000	-9.573250000000	-4.072002000000
C	2.965605000000	-2.443350000000	-8.190038000000
C	2.198156000000	-3.698107000000	-7.755438000000
C	2.031475000000	-4.776248000000	-8.845809000000
C	3.378125000000	-5.343629000000	-9.320658000000
C	1.114157000000	-5.904662000000	-8.350765000000
H	2.493629000000	-1.973196000000	-9.061959000000
H	2.702698000000	-4.150958000000	-6.887243000000
H	1.196614000000	-3.400301000000	-7.411064000000
H	1.541688000000	-4.298381000000	-9.708971000000
H	0.132471000000	-5.521159000000	-8.047399000000
H	0.952657000000	-6.658385000000	-9.129856000000
H	1.556931000000	-6.419198000000	-7.487208000000
H	4.021999000000	-4.572163000000	-9.756856000000
H	3.923654000000	-5.804213000000	-8.486138000000
H	3.228438000000	-6.117224000000	-10.082618000000
O	-7.009285000000	-7.829242000000	-5.158596000000
H	-6.874273000000	-7.735445000000	-4.188521000000
H	-7.067210000000	-6.881082000000	-5.446538000000
O	-6.861507000000	-5.140745000000	-5.501726000000
H	-5.942190000000	-5.019826000000	-5.160875000000
H	-6.822607000000	-4.762139000000	-6.394127000000
H	2.900010000000	-12.590657000000	-4.430922000000
H	4.003279000000	-2.670556000000	-8.456307000000
H	-13.091528000000	-8.743619000000	-2.272325000000
H	2.992204000000	-1.697429000000	-7.386016000000
H	-8.185575000000	-12.321722000000	-0.743305000000
H	-3.543986000000	-15.415083000000	-3.599862000000
H	-7.800196000000	-11.400770000000	-5.426625000000

$^{4}Re_{BPA}$

C	0.568778000000	-0.822069000000	0.917957000000
C	0.261954000000	-1.061884000000	2.390823000000
O	1.081042000000	-1.580626000000	3.149552000000
C	0.884112000000	-2.139135000000	0.189558000000
H	-0.249111000000	-0.297264000000	0.416762000000
H	1.750090000000	-2.608752000000	0.672112000000
H	1.135938000000	-1.947814000000	-0.856393000000
N	-0.982250000000	-0.687954000000	2.805908000000
C	-1.409744000000	-0.839164000000	4.185714000000
C	-1.756381000000	-2.256960000000	4.674623000000
O	-2.092716000000	-2.410457000000	5.842427000000
H	-1.649799000000	-0.350990000000	2.124138000000
H	-0.611870000000	-0.488552000000	4.847216000000
N	-1.642506000000	-3.265766000000	3.767282000000
C	-1.683827000000	-4.659570000000	4.167487000000
C	-0.322989000000	-5.371000000000	4.277160000000
O	-0.300870000000	-6.582522000000	4.471828000000
H	-1.350421000000	-3.056953000000	2.818039000000
H	-2.291952000000	-5.240987000000	3.467818000000
H	-2.160832000000	-4.705284000000	5.149786000000
N	0.781723000000	-4.589993000000	4.155841000000
C	2.120921000000	-5.135903000000	4.276036000000
H	0.685632000000	-3.592727000000	3.998936000000
H	2.742599000000	-4.813804000000	3.433547000000
H	2.602650000000	-4.814451000000	5.208274000000
H	2.039881000000	-6.224007000000	4.276697000000
H	-2.281683000000	-0.202455000000	4.357145000000
H	1.456673000000	-0.178435000000	0.868392000000
S	-0.439717000000	-3.400438000000	0.301121000000
Fe	-2.191883000000	-3.415181000000	-1.655465000000
C	-1.925003000000	-0.005056000000	-1.872364000000
C	0.204220000000	-3.750370000000	-4.072724000000
C	-2.272118000000	-6.812489000000	-1.269505000000
C	-4.289346000000	-3.066405000000	1.038151000000
N	-1.058218000000	-2.137170000000	-2.723741000000
C	-1.150049000000	-0.765340000000	-2.737751000000
C	-0.295576000000	-0.215220000000	-3.759663000000
C	0.317085000000	-1.268707000000	-4.367060000000
C	-0.171375000000	-2.460983000000	-3.721933000000
N	-1.224521000000	-4.962566000000	-2.491151000000
C	-0.286894000000	-4.910023000000	-3.491449000000
C	0.139875000000	-6.239251000000	-3.854118000000
C	-0.551876000000	-7.105100000000	-3.066554000000
C	-1.405348000000	-6.297333000000	-2.224753000000
N	-3.089073000000	-4.676428000000	-0.370763000000
C	-3.053063000000	-6.048067000000	-0.411868000000
C	-3.923225000000	-6.606158000000	0.594616000000
C	-4.487047000000	-5.552140000000	1.247707000000
C	-3.963788000000	-4.354249000000	0.638255000000
N	-2.889389000000	-1.851964000000	-0.572824000000
C	-3.781669000000	-1.904771000000	0.476523000000
C	-4.137871000000	-0.573996000000	0.894258000000
C	-3.478659000000	0.290455000000	0.068176000000
C	-2.716085000000	-0.512955000000	-0.852165000000
H	-1.895796000000	1.073660000000	-1.992694000000
H	0.929524000000	-3.860660000000	-4.872400000000

H	-2.361372000000	-7.890886000000	-1.181238000000
H	-4.990055000000	-2.959818000000	1.860251000000
H	-0.463241000000	-8.185003000000	-3.050081000000
H	0.877893000000	-6.469998000000	-4.610920000000
H	-4.065622000000	-7.664646000000	0.763808000000
H	-5.179885000000	-5.565053000000	2.079607000000
H	1.032106000000	-1.259356000000	-5.179531000000
H	-0.185085000000	0.841523000000	-3.966940000000
H	-3.512867000000	1.372685000000	0.059271000000
H	-4.823468000000	-0.345030000000	1.700528000000
O	-3.427119000000	-3.408934000000	-2.721145000000
C	-3.308812000000	-2.719979000000	-6.043164000000
C	-5.235240000000	-1.514809000000	-10.364301000000
C	-3.168418000000	-2.629592000000	-8.483500000000
C	-2.988987000000	-2.043278000000	-7.224704000000
C	-3.808585000000	-4.021439000000	-6.106937000000
C	-3.993218000000	-4.638043000000	-7.351721000000
C	-3.678866000000	-3.939648000000	-8.514094000000
H	-1.737057000000	-3.698799000000	-10.606626000000
H	-1.169985000000	-2.156328000000	-11.280973000000
C	-2.409059000000	-0.445701000000	-9.596123000000
C	-1.536931000000	-2.648353000000	-10.372695000000
C	-2.786548000000	-1.932304000000	-9.805201000000
C	-3.978294000000	-1.978477000000	-10.783569000000
C	-3.870925000000	-2.420721000000	-12.109595000000
C	-4.959575000000	-2.402292000000	-12.982246000000
C	-6.197170000000	-1.930318000000	-12.542662000000
C	-6.331398000000	-1.485162000000	-11.222760000000
H	-2.924467000000	-2.793160000000	-12.486407000000
H	-4.862214000000	-2.751865000000	-14.005389000000
O	-7.235348000000	-1.929930000000	-13.435136000000
H	-7.293528000000	-1.119614000000	-10.866024000000
H	-5.364634000000	-1.179393000000	-9.338599000000
O	-4.169573000000	-4.733303000000	-4.985263000000
H	-3.175356000000	-2.239555000000	-5.078208000000
H	-4.346648000000	-5.665727000000	-7.388715000000
H	-3.836705000000	-4.426818000000	-9.472777000000
H	-3.882131000000	-4.258859000000	-4.164116000000
H	-2.597679000000	-1.034787000000	-7.141032000000
H	-8.024260000000	-1.573965000000	-12.997745000000
H	-0.732472000000	-2.621410000000	-9.630017000000
H	-3.225236000000	0.123631000000	-9.139240000000
H	-1.519974000000	-0.342102000000	-8.962907000000
H	-2.184724000000	0.014502000000	-10.564000000000
C	-12.386078000000	-9.551014000000	-2.490038000000
C	-10.952185000000	-9.079151000000	-2.616706000000
O	-10.007570000000	-9.876220000000	-2.648106000000
H	-12.679022000000	-10.036692000000	-3.427507000000
H	-12.446917000000	-10.305271000000	-1.701020000000
N	-10.751471000000	-7.732545000000	-2.680206000000
C	-9.460271000000	-7.163449000000	-3.089354000000
C	-8.341710000000	-7.439598000000	-2.075383000000
O	-7.162328000000	-7.475501000000	-2.429665000000
C	-9.601514000000	-5.656560000000	-3.341896000000
H	-11.563824000000	-7.135051000000	-2.766163000000
H	-9.126343000000	-7.652523000000	-4.010873000000
H	-10.383143000000	-5.471962000000	-4.088062000000

H	-9.856530000000	-5.117426000000	-2.422239000000
H	-8.665218000000	-5.258961000000	-3.742385000000
N	-8.689967000000	-7.626312000000	-0.777197000000
C	-7.674739000000	-7.982991000000	0.200037000000
C	-7.072433000000	-9.388004000000	0.029335000000
O	-6.011193000000	-9.655877000000	0.599711000000
H	-9.668120000000	-7.613556000000	-0.525775000000
H	-8.110118000000	-7.917109000000	1.201624000000
H	-6.839961000000	-7.280148000000	0.145752000000
N	-7.791338000000	-10.271755000000	-0.698394000000
C	-7.348626000000	-11.636671000000	-0.923585000000
C	-6.778502000000	-11.955530000000	-2.317252000000
O	-5.824869000000	-12.725200000000	-2.418646000000
H	-8.632553000000	-9.957311000000	-1.172120000000
H	-6.567486000000	-11.864214000000	-0.199281000000
N	-7.410283000000	-11.420939000000	-3.394528000000
C	-7.103375000000	-11.898874000000	-4.742675000000
C	-5.655696000000	-11.636482000000	-5.191673000000
O	-4.908739000000	-12.564071000000	-5.496091000000
H	-8.232593000000	-10.841907000000	-3.263930000000
H	-7.245359000000	-12.981263000000	-4.808544000000
N	-5.257796000000	-10.335141000000	-5.202695000000
C	-3.898184000000	-10.028516000000	-5.640945000000
C	-2.880762000000	-10.635516000000	-4.667918000000
O	-1.793486000000	-11.057975000000	-5.089584000000
C	-3.695474000000	-8.497576000000	-5.832188000000
O	-4.677173000000	-8.004943000000	-6.731805000000
C	-2.327662000000	-8.178761000000	-6.425171000000
H	-5.956404000000	-9.595276000000	-5.199963000000
H	-3.705085000000	-10.513435000000	-6.603480000000
H	-3.796089000000	-8.001990000000	-4.852988000000
H	-5.512772000000	-7.907412000000	-6.230942000000
H	-2.222418000000	-7.094029000000	-6.513099000000
H	-2.245331000000	-8.617594000000	-7.425241000000
H	-1.510190000000	-8.565903000000	-5.813141000000
N	-3.218933000000	-10.686107000000	-3.364794000000
C	-2.337339000000	-11.228025000000	-2.335121000000
C	-1.921842000000	-12.687716000000	-2.621361000000
O	-0.773172000000	-13.069279000000	-2.387071000000
C	-3.056261000000	-11.133003000000	-0.967502000000
O	-3.667555000000	-9.839394000000	-0.919058000000
C	-2.094568000000	-11.344179000000	0.198808000000
H	-4.063509000000	-10.230880000000	-3.040635000000
H	-1.400425000000	-10.661073000000	-2.311484000000
H	-3.843328000000	-11.898887000000	-0.951076000000
H	-4.412517000000	-9.864399000000	-0.285243000000
H	-2.642230000000	-11.318494000000	1.147140000000
H	-1.577514000000	-12.305668000000	0.120557000000
H	-1.341136000000	-10.549480000000	0.216496000000
N	-2.885847000000	-13.502432000000	-3.114116000000
C	-2.594847000000	-14.878294000000	-3.485190000000
C	-1.784890000000	-15.076998000000	-4.784124000000
O	-1.430874000000	-16.202682000000	-5.108659000000
H	-3.819133000000	-13.141020000000	-3.286296000000
H	-2.020437000000	-15.362257000000	-2.690479000000
N	-1.500750000000	-13.937557000000	-5.467904000000
C	-0.688729000000	-13.877987000000	-6.668143000000

C	0.602506000000	-13.052679000000	-6.438097000000
O	1.273181000000	-12.642417000000	-7.383220000000
C	-1.518341000000	-13.289563000000	-7.861712000000
O	-2.856356000000	-13.765159000000	-7.838307000000
C	-0.943234000000	-13.686521000000	-9.215410000000
H	-1.842492000000	-13.051318000000	-5.115732000000
H	-0.408250000000	-14.910788000000	-6.901387000000
H	-1.502652000000	-12.192631000000	-7.763489000000
H	-3.278457000000	-13.474409000000	-7.012388000000
H	-1.547514000000	-13.245481000000	-10.015489000000
H	-0.978593000000	-14.776965000000	-9.326253000000
H	0.089649000000	-13.346473000000	-9.302189000000
N	0.927404000000	-12.801393000000	-5.137566000000
C	2.054962000000	-11.964862000000	-4.757180000000
C	1.682391000000	-10.983516000000	-3.633071000000
O	0.654022000000	-10.078091000000	-4.040690000000
C	2.883314000000	-10.142585000000	-3.215212000000
H	0.362009000000	-13.202946000000	-4.397956000000
H	2.368803000000	-11.422586000000	-5.653297000000
H	1.329635000000	-11.568834000000	-2.769894000000
H	-0.072911000000	-10.592340000000	-4.444596000000
H	2.595089000000	-9.437797000000	-2.428382000000
H	3.691990000000	-10.774652000000	-2.831941000000
H	3.262193000000	-9.567147000000	-4.067956000000
C	2.959685000000	-2.449418000000	-8.183381000000
C	2.193147000000	-3.704675000000	-7.748618000000
C	2.028665000000	-4.783765000000	-8.838393000000
C	3.376352000000	-5.350587000000	-9.310962000000
C	1.111465000000	-5.912421000000	-8.343699000000
H	2.487877000000	-1.980203000000	-9.055905000000
H	2.697356000000	-4.156420000000	-6.879658000000
H	1.191006000000	-3.407700000000	-7.405323000000
H	1.539733000000	-4.306932000000	-9.702623000000
H	0.128993000000	-5.529392000000	-8.042314000000
H	0.951851000000	-6.667087000000	-9.122271000000
H	1.553223000000	-6.425677000000	-7.478867000000
H	4.020421000000	-4.578980000000	-9.746633000000
H	3.920922000000	-5.810390000000	-8.475388000000
H	3.228267000000	-6.124697000000	-10.072714000000
O	-7.011774000000	-7.831311000000	-5.163561000000
H	-6.877213000000	-7.736263000000	-4.193543000000
H	-7.069671000000	-6.883570000000	-5.452798000000
O	-6.862860000000	-5.143260000000	-5.510711000000
H	-5.942589000000	-5.024163000000	-5.171944000000
H	-6.825032000000	-4.766002000000	-6.403722000000
H	2.900931000000	-12.585074000000	-4.424518000000
H	3.997779000000	-2.675716000000	-8.448797000000
H	-13.093645000000	-8.744553000000	-2.277039000000
H	2.984956000000	-1.703022000000	-7.379753000000
H	-8.189070000000	-12.319577000000	-0.744738000000
H	-3.541323000000	-15.413350000000	-3.593417000000
H	-7.798934000000	-11.404797000000	-5.428236000000

^{2}TS3$_B$

C	4.628598000000	-5.535557000000	-0.825800000000
C	5.661348000000	-5.827333000000	0.259044000000
O	5.374922000000	-6.158291000000	1.405600000000
C	3.226866000000	-5.281117000000	-0.270524000000
H	4.958993000000	-4.686883000000	-1.433934000000
H	2.906268000000	-6.133145000000	0.335720000000
H	2.519203000000	-5.176001000000	-1.100925000000
N	6.971105000000	-5.703767000000	-0.139874000000
C	8.055784000000	-6.224519000000	0.682060000000
C	8.434067000000	-5.391746000000	1.916562000000
O	9.183747000000	-5.866193000000	2.759046000000
H	7.155030000000	-5.513178000000	-1.115359000000
H	7.803604000000	-7.219581000000	1.061668000000
N	7.899223000000	-4.140784000000	1.978798000000
C	8.052538000000	-3.282045000000	3.134834000000
C	6.749049000000	-2.975604000000	3.890291000000
O	6.653386000000	-1.930229000000	4.536419000000
H	7.288392000000	-3.843872000000	1.229465000000
H	8.494833000000	-2.321378000000	2.857822000000
H	8.743340000000	-3.790136000000	3.815319000000
N	5.790681000000	-3.929565000000	3.821189000000
C	4.519065000000	-3.816171000000	4.515419000000
H	5.914583000000	-4.712156000000	3.187758000000
H	4.564911000000	-2.933333000000	5.154391000000
H	3.692235000000	-3.712956000000	3.804539000000
H	4.344456000000	-4.702678000000	5.135480000000
H	8.948761000000	-6.322119000000	0.057772000000
H	4.598660000000	-6.411879000000	-1.492828000000
S	3.088311000000	-3.802873000000	0.793952000000
Fe	2.979615000000	-1.642208000000	-0.463389000000
C	6.026814000000	-1.646090000000	-1.973401000000
C	1.665896000000	-3.416156000000	-3.098280000000
C	-0.118034000000	-1.496653000000	0.968052000000
C	4.388788000000	-0.487496000000	2.434435000000
N	3.706256000000	-2.406388000000	-2.186598000000
C	4.975871000000	-2.224100000000	-2.675195000000
C	5.085783000000	-2.789994000000	-3.999858000000
C	3.868509000000	-3.322157000000	-4.300119000000
C	3.012459000000	-3.063744000000	-3.166002000000
N	1.145144000000	-2.357463000000	-0.951417000000
C	0.796363000000	-3.072254000000	-2.071605000000
C	-0.618203000000	-3.368502000000	-2.053385000000
C	-1.130176000000	-2.799637000000	-0.928689000000
C	-0.021293000000	-2.167047000000	-0.244744000000
N	2.276882000000	-1.035743000000	1.315045000000
C	0.959834000000	-1.013849000000	1.707313000000
C	0.851506000000	-0.516941000000	3.058746000000
C	2.122736000000	-0.286831000000	3.495350000000
C	3.006205000000	-0.607547000000	2.402432000000
N	4.829937000000	-1.127575000000	0.106348000000
C	5.231606000000	-0.706586000000	1.347491000000
C	6.655932000000	-0.480183000000	1.365811000000
C	7.109764000000	-0.758429000000	0.108880000000
C	5.963769000000	-1.179163000000	-0.663442000000
H	6.990950000000	-1.598084000000	-2.471903000000
H	1.250246000000	-3.958907000000	-3.941891000000

H	-1.102788000000	-1.385673000000	1.413091000000
H	4.855209000000	-0.208162000000	3.373377000000
H	-2.156000000000	-2.841931000000	-0.581326000000
H	-1.143139000000	-3.937790000000	-2.809826000000
H	-0.078470000000	-0.384914000000	3.595101000000
H	2.449815000000	0.059070000000	4.467868000000
H	3.559107000000	-3.831227000000	-5.204079000000
H	5.983022000000	-2.775907000000	-4.606032000000
H	8.123920000000	-0.706869000000	-0.267669000000
H	7.209903000000	-0.164771000000	2.239955000000
O	2.601368000000	-0.169878000000	-1.331105000000
C	2.698166000000	1.564913000000	-0.617262000000
C	5.129222000000	5.929575000000	-0.350264000000
C	4.367467000000	3.183725000000	-1.436876000000
C	4.007112000000	2.143994000000	-0.592345000000
C	1.752883000000	2.116680000000	-1.543633000000
C	2.081742000000	3.190943000000	-2.357875000000
C	3.374621000000	3.716410000000	-2.296647000000
H	5.874897000000	3.582883000000	-3.706318000000
H	7.469250000000	3.577487000000	-2.921071000000
C	6.688968000000	3.272051000000	-0.343738000000
C	6.428539000000	3.246938000000	-2.823385000000
C	5.795371000000	3.762142000000	-1.507552000000
C	5.742069000000	5.301659000000	-1.446449000000
C	6.318365000000	6.133433000000	-2.416867000000
C	6.294148000000	7.523906000000	-2.304382000000
C	5.687755000000	8.123621000000	-1.199736000000
C	5.099986000000	7.316106000000	-0.219100000000
H	6.803342000000	5.702176000000	-3.285869000000
H	6.745821000000	8.154518000000	-3.063789000000
O	5.694063000000	9.490403000000	-1.131488000000
H	4.624605000000	7.773093000000	0.648213000000
H	4.661818000000	5.320672000000	0.419807000000
O	0.550746000000	1.490692000000	-1.663542000000
H	2.313650000000	1.152258000000	0.311037000000
H	1.348142000000	3.581362000000	-3.056710000000
H	3.629081000000	4.550441000000	-2.944345000000
H	0.756433000000	0.545625000000	-1.467067000000
H	4.710841000000	1.742804000000	0.126144000000
H	5.279726000000	9.766798000000	-0.299561000000
H	6.419976000000	2.152224000000	-2.823811000000
H	6.273668000000	3.539632000000	0.633496000000
H	6.825102000000	2.186062000000	-0.369680000000
H	7.676786000000	3.738030000000	-0.422773000000
C	-2.198826000000	8.325959000000	3.249246000000
C	-1.914477000000	6.957250000000	2.664555000000
O	-2.733422000000	6.035544000000	2.747852000000
H	-2.630768000000	8.207120000000	4.246236000000
H	-1.314322000000	8.966779000000	3.310031000000
N	-0.706805000000	6.791092000000	2.054382000000
C	-0.417712000000	5.632886000000	1.197747000000
C	-0.362112000000	4.305858000000	1.966667000000
O	-0.558924000000	3.241371000000	1.379480000000
C	0.892127000000	5.863250000000	0.433974000000
H	-0.128261000000	7.611003000000	1.922944000000
H	-1.238595000000	5.497608000000	0.483684000000
H	0.811758000000	6.748201000000	-0.206916000000

H	1.737002000000	5.998027000000	1.119479000000
H	1.104995000000	5.003885000000	-0.206262000000
N	-0.080295000000	4.340204000000	3.294048000000
C	-0.141782000000	3.117704000000	4.078515000000
C	-1.556362000000	2.549257000000	4.282009000000
O	-1.684860000000	1.372537000000	4.635728000000
H	0.059198000000	5.234609000000	3.742060000000
H	0.294301000000	3.308110000000	5.063890000000
H	0.444123000000	2.332087000000	3.595882000000
N	-2.587169000000	3.408029000000	4.116289000000
C	-3.966740000000	3.017368000000	4.347980000000
C	-4.881536000000	2.878119000000	3.119876000000
O	-5.815232000000	2.079640000000	3.156033000000
H	-2.401442000000	4.351294000000	3.788748000000
H	-3.964280000000	2.056489000000	4.861180000000
N	-4.669152000000	3.714244000000	2.069619000000
C	-5.694242000000	3.870868000000	1.039421000000
C	-5.923433000000	2.618476000000	0.177161000000
O	-7.005354000000	2.038276000000	0.156528000000
H	-3.923847000000	4.400619000000	2.104787000000
H	-6.660913000000	4.102466000000	1.495258000000
N	-4.855074000000	2.207769000000	-0.564844000000
C	-4.975198000000	0.979708000000	-1.343613000000
C	-5.152160000000	-0.265631000000	-0.460717000000
O	-5.776769000000	-1.243076000000	-0.899408000000
C	-3.788863000000	0.875439000000	-2.323353000000
O	-3.847230000000	1.994225000000	-3.231531000000
C	-3.784958000000	-0.386763000000	-3.172980000000
H	-3.952159000000	2.708262000000	-0.504708000000
H	-5.896467000000	1.020253000000	-1.933967000000
H	-2.853677000000	0.939494000000	-1.744701000000
H	-4.229376000000	2.747129000000	-2.747898000000
H	-2.985495000000	-0.311456000000	-3.917181000000
H	-4.737908000000	-0.503332000000	-3.696955000000
H	-3.610600000000	-1.278908000000	-2.566698000000
N	-4.637839000000	-0.221489000000	0.780654000000
C	-4.767714000000	-1.324343000000	1.727107000000
C	-6.240051000000	-1.712555000000	1.965669000000
O	-6.549173000000	-2.887455000000	2.182487000000
C	-4.090548000000	-0.920725000000	3.059892000000
O	-2.875620000000	-0.258369000000	2.698359000000
C	-3.834336000000	-2.129738000000	3.955413000000
H	-4.033298000000	0.540640000000	1.063870000000
H	-4.284860000000	-2.217472000000	1.317185000000
H	-4.751760000000	-0.206319000000	3.569751000000
H	-2.520711000000	0.212894000000	3.480215000000
H	-3.405777000000	-1.806059000000	4.910566000000
H	-4.760301000000	-2.677602000000	4.155186000000
H	-3.126043000000	-2.812368000000	3.473619000000
N	-7.149776000000	-0.710239000000	1.940941000000
C	-8.562952000000	-0.987665000000	2.138280000000
C	-9.257791000000	-1.798506000000	1.026020000000
O	-10.347974000000	-2.306704000000	1.244723000000
H	-6.856053000000	0.248138000000	1.778645000000
H	-8.716353000000	-1.555937000000	3.059604000000
N	-8.594503000000	-1.873766000000	-0.167355000000
C	-9.055010000000	-2.701876000000	-1.276727000000

C	-8.055531000000	-3.841352000000	-1.591451000000
O	-7.998022000000	-4.351324000000	-2.707762000000
C	-9.339808000000	-1.818882000000	-2.530842000000
O	-10.018358000000	-0.630408000000	-2.145898000000
C	-10.227512000000	-2.505504000000	-3.561503000000
H	-7.656907000000	-1.488303000000	-0.241312000000
H	-9.998334000000	-3.148741000000	-0.942158000000
H	-8.368158000000	-1.569503000000	-2.990007000000
H	-9.556015000000	-0.281583000000	-1.365831000000
H	-10.433020000000	-1.809231000000	-4.381644000000
H	-11.185496000000	-2.779543000000	-3.103850000000
H	-9.740803000000	-3.400024000000	-3.950370000000
N	-7.258767000000	-4.239490000000	-0.558163000000
C	-6.269559000000	-5.297106000000	-0.706809000000
C	-4.920796000000	-4.907980000000	-0.084133000000
O	-4.370747000000	-3.745516000000	-0.709475000000
C	-3.897765000000	-6.027972000000	-0.233692000000
H	-7.368855000000	-3.819338000000	0.358889000000
H	-6.159300000000	-5.480876000000	-1.778571000000
H	-5.083974000000	-4.703713000000	0.985436000000
H	-5.065401000000	-3.060706000000	-0.772765000000
H	-2.938207000000	-5.724389000000	0.197392000000
H	-4.233845000000	-6.936617000000	0.278054000000
H	-3.738056000000	-6.260548000000	-1.292886000000
C	0.290068000000	3.307961000000	-6.501545000000
C	1.003107000000	1.986086000000	-6.190068000000
C	0.066530000000	0.835231000000	-5.770314000000
C	-0.846436000000	0.376426000000	-6.917673000000
C	0.872635000000	-0.346985000000	-5.212341000000
H	-0.295816000000	3.643637000000	-5.637279000000
H	1.594107000000	1.665220000000	-7.061477000000
H	1.726288000000	2.157307000000	-5.380347000000
H	-0.573244000000	1.217182000000	-4.959737000000
H	1.470309000000	-0.053472000000	-4.341420000000
H	0.213043000000	-1.166422000000	-4.901374000000
H	1.560414000000	-0.745291000000	-5.970715000000
H	-1.471636000000	1.190683000000	-7.299562000000
H	-0.251652000000	-0.010713000000	-7.756168000000
H	-1.517630000000	-0.427131000000	-6.590433000000
O	-2.455361000000	3.723056000000	-0.617503000000
H	-1.787126000000	3.372107000000	0.008649000000
H	-2.018662000000	3.644327000000	-1.506083000000
O	-1.317503000000	3.141008000000	-3.041484000000
H	-0.735250000000	2.421145000000	-2.727076000000
H	-2.120479000000	2.692201000000	-3.388082000000
H	-6.627118000000	-6.225682000000	-0.237678000000
H	-0.391986000000	3.215666000000	-7.354584000000
H	-2.949752000000	8.824081000000	2.625693000000
H	1.012780000000	4.095658000000	-6.746833000000
H	-4.441238000000	3.749747000000	5.014360000000
H	-9.083000000000	-0.031179000000	2.238397000000
H	-5.382315000000	4.702766000000	0.401544000000

^{4}TS3$_B$

C	4.561578000000	-5.588444000000	-0.783603000000
C	5.605253000000	-5.850834000000	0.298805000000
O	5.325621000000	-6.096831000000	1.468863000000
C	3.155623000000	-5.376081000000	-0.221248000000
H	4.866112000000	-4.727866000000	-1.389102000000
H	2.847764000000	-6.244430000000	0.367716000000
H	2.447810000000	-5.264274000000	-1.052309000000
N	6.909662000000	-5.796341000000	-0.127148000000
C	7.992259000000	-6.313600000000	0.701622000000
C	8.399992000000	-5.454342000000	1.908159000000
O	9.131613000000	-5.930213000000	2.765294000000
H	7.079886000000	-5.676384000000	-1.116477000000
H	7.726958000000	-7.292716000000	1.112721000000
N	7.914267000000	-4.182150000000	1.930558000000
C	8.093074000000	-3.299621000000	3.065480000000
C	6.808224000000	-2.971644000000	3.844441000000
O	6.739209000000	-1.920779000000	4.484582000000
H	7.311891000000	-3.885502000000	1.174467000000
H	8.532966000000	-2.347717000000	2.757350000000
H	8.795437000000	-3.795431000000	3.742963000000
N	5.836438000000	-3.913765000000	3.802891000000
C	4.587059000000	-3.782574000000	4.533121000000
H	5.935760000000	-4.703990000000	3.174540000000
H	4.670416000000	-2.912182000000	5.185270000000
H	3.741927000000	-3.647665000000	3.849233000000
H	4.408622000000	-4.675820000000	5.141972000000
H	8.875994000000	-6.445766000000	0.070670000000
H	4.552405000000	-6.461001000000	-1.455423000000
S	2.983744000000	-3.904461000000	0.841611000000
Fe	2.940990000000	-1.631179000000	-0.487851000000
C	5.981261000000	-1.679671000000	-2.006618000000
C	1.618350000000	-3.487868000000	-3.046540000000
C	-0.138653000000	-1.465498000000	0.988628000000
C	4.386724000000	-0.513521000000	2.417073000000
N	3.657133000000	-2.436395000000	-2.189731000000
C	4.923605000000	-2.259420000000	-2.693672000000
C	5.021649000000	-2.850264000000	-4.008592000000
C	3.805010000000	-3.396954000000	-4.284300000000
C	2.959920000000	-3.120794000000	-3.145590000000
N	1.126578000000	-2.390671000000	-0.906095000000
C	0.766572000000	-3.136194000000	-2.009261000000
C	-0.644490000000	-3.434068000000	-1.967557000000
C	-1.150476000000	-2.831696000000	-0.856556000000
C	-0.042893000000	-2.178122000000	-0.197361000000
N	2.258345000000	-1.033848000000	1.314332000000
C	0.947929000000	-0.975079000000	1.712880000000
C	0.853731000000	-0.454471000000	3.057356000000
C	2.131819000000	-0.249079000000	3.485219000000
C	3.002417000000	-0.605877000000	2.391602000000
N	4.810924000000	-1.179428000000	0.092369000000
C	5.222038000000	-0.750569000000	1.326350000000
C	6.644876000000	-0.517944000000	1.329727000000
C	7.085815000000	-0.792610000000	0.066331000000
C	5.933435000000	-1.218327000000	-0.691943000000
H	6.939303000000	-1.629426000000	-2.516365000000
H	1.191312000000	-4.047804000000	-3.873012000000

H	-1.122356000000	-1.335640000000	1.430857000000
H	4.862482000000	-0.227841000000	3.349522000000
H	-2.175271000000	-2.863341000000	-0.504837000000
H	-1.175844000000	-4.023202000000	-2.704079000000
H	-0.071169000000	-0.294913000000	3.595275000000
H	2.471703000000	0.102491000000	4.451318000000
H	3.490053000000	-3.928477000000	-5.173310000000
H	5.912619000000	-2.845025000000	-4.624090000000
H	8.094651000000	-0.731193000000	-0.322828000000
H	7.206741000000	-0.198086000000	2.197143000000
O	2.651706000000	-0.131145000000	-1.336244000000
C	2.683860000000	1.560085000000	-0.620920000000
C	5.118417000000	5.915630000000	-0.274714000000
C	4.360988000000	3.191971000000	-1.418492000000
C	3.992256000000	2.152686000000	-0.578198000000
C	1.746833000000	2.132204000000	-1.550536000000
C	2.086805000000	3.199448000000	-2.362950000000
C	3.381394000000	3.728575000000	-2.291175000000
H	5.885282000000	3.634209000000	-3.669845000000
H	7.474419000000	3.610037000000	-2.873985000000
C	6.677567000000	3.259049000000	-0.309691000000
C	6.432122000000	3.280321000000	-2.789864000000
C	5.790184000000	3.770624000000	-1.468934000000
C	5.738562000000	5.308732000000	-1.378489000000
C	6.324862000000	6.158781000000	-2.326679000000
C	6.303896000000	7.546859000000	-2.185505000000
C	5.690986000000	8.125176000000	-1.073294000000
C	5.092738000000	7.299242000000	-0.114599000000
H	6.815240000000	5.743932000000	-3.200708000000
H	6.763475000000	8.191743000000	-2.928047000000
O	5.701180000000	9.490723000000	-0.975611000000
H	4.612298000000	7.739682000000	0.758591000000
H	4.642603000000	5.291597000000	0.477853000000
O	0.532584000000	1.514472000000	-1.671988000000
H	2.282316000000	1.179111000000	0.313212000000
H	1.359419000000	3.592072000000	-3.067394000000
H	3.642214000000	4.563599000000	-2.934669000000
H	0.722689000000	0.567526000000	-1.488587000000
H	4.689831000000	1.750782000000	0.146218000000
H	5.284536000000	9.749584000000	-0.139245000000
H	6.420983000000	2.185816000000	-2.811479000000
H	6.258822000000	3.511171000000	0.670124000000
H	6.810004000000	2.173177000000	-0.353726000000
H	7.667159000000	3.723440000000	-0.376506000000
C	-2.174415000000	8.407226000000	3.078681000000
C	-1.893628000000	7.025971000000	2.522177000000
O	-2.713792000000	6.107539000000	2.627841000000
H	-2.598072000000	8.310252000000	4.081624000000
H	-1.290072000000	9.049950000000	3.118028000000
N	-0.688129000000	6.845446000000	1.912339000000
C	-0.401901000000	5.668736000000	1.079957000000
C	-0.366083000000	4.357784000000	1.876350000000
O	-0.591315000000	3.284291000000	1.316689000000
C	0.918289000000	5.873047000000	0.326666000000
H	-0.110216000000	7.662090000000	1.759440000000
H	-1.216887000000	5.527349000000	0.360464000000
H	0.852140000000	6.745532000000	-0.332786000000

H	1.756413000000	6.015840000000	1.018655000000
H	1.133116000000	4.999845000000	-0.294007000000
N	-0.066570000000	4.414691000000	3.199375000000
C	-0.130803000000	3.207689000000	4.007098000000
C	-1.547399000000	2.650863000000	4.227444000000
O	-1.680975000000	1.480588000000	4.600737000000
H	0.095697000000	5.314958000000	3.627458000000
H	0.310540000000	3.414953000000	4.986724000000
H	0.448785000000	2.409617000000	3.537318000000
N	-2.573572000000	3.513465000000	4.054804000000
C	-3.954515000000	3.134747000000	4.296584000000
C	-4.871416000000	2.968380000000	3.073282000000
O	-5.813780000000	2.182045000000	3.134477000000
H	-2.383974000000	4.449054000000	3.707968000000
H	-3.956368000000	2.187090000000	4.833966000000
N	-4.650356000000	3.768656000000	1.996924000000
C	-5.673924000000	3.901498000000	0.961883000000
C	-5.902933000000	2.628852000000	0.129406000000
O	-6.981819000000	2.041367000000	0.130755000000
H	-3.898600000000	4.448705000000	2.010603000000
H	-6.640771000000	4.144008000000	1.411517000000
N	-4.838785000000	2.207021000000	-0.611010000000
C	-4.958875000000	0.965773000000	-1.369184000000
C	-5.138655000000	-0.263944000000	-0.464616000000
O	-5.757416000000	-1.251812000000	-0.888387000000
C	-3.770825000000	0.846280000000	-2.345503000000
O	-3.833491000000	1.944296000000	-3.277768000000
C	-3.759745000000	-0.433394000000	-3.168527000000
H	-3.939873000000	2.717739000000	-0.572595000000
H	-5.879058000000	0.996006000000	-1.962077000000
H	-2.836622000000	0.926803000000	-1.767304000000
H	-4.206768000000	2.710231000000	-2.808021000000
H	-2.959210000000	-0.369812000000	-3.912653000000
H	-4.711135000000	-0.565563000000	-3.691602000000
H	-3.582472000000	-1.311539000000	-2.542955000000
N	-4.633996000000	-0.192486000000	0.779347000000
C	-4.777184000000	-1.269358000000	1.752874000000
C	-6.252970000000	-1.647444000000	1.985618000000
O	-6.565579000000	-2.814098000000	2.240314000000
C	-4.111498000000	-0.830967000000	3.080391000000
O	-2.886223000000	-0.192683000000	2.710525000000
C	-3.876403000000	-2.012541000000	4.017315000000
H	-4.033336000000	0.577395000000	1.049773000000
H	-4.291808000000	-2.174217000000	1.372911000000
H	-4.771311000000	-0.094273000000	3.559355000000
H	-2.532296000000	0.298928000000	3.480279000000
H	-3.453807000000	-1.661307000000	4.965389000000
H	-4.809488000000	-2.544658000000	4.226017000000
H	-3.169991000000	-2.717423000000	3.565691000000
N	-7.162135000000	-0.647178000000	1.913759000000
C	-8.577246000000	-0.917912000000	2.104888000000
C	-9.258002000000	-1.774407000000	1.018911000000
O	-10.344713000000	-2.284786000000	1.249416000000
H	-6.869860000000	0.303702000000	1.709317000000
H	-8.741996000000	-1.447517000000	3.047000000000
N	-8.589215000000	-1.884589000000	-0.169434000000
C	-9.038882000000	-2.759070000000	-1.247714000000

C	-8.028543000000	-3.900954000000	-1.514360000000
O	-7.954069000000	-4.447475000000	-2.612155000000
C	-9.330695000000	-1.928614000000	-2.535117000000
O	-10.029275000000	-0.737517000000	-2.197061000000
C	-10.202877000000	-2.667011000000	-3.543119000000
H	-7.652441000000	-1.498139000000	-0.251549000000
H	-9.978884000000	-3.201202000000	-0.897734000000
H	-8.360938000000	-1.681376000000	-2.999426000000
H	-9.580125000000	-0.357341000000	-1.423945000000
H	-10.416685000000	-2.004475000000	-4.388692000000
H	-11.158017000000	-2.940335000000	-3.079131000000
H	-9.699612000000	-3.566653000000	-3.897348000000
N	-7.241728000000	-4.259358000000	-0.458994000000
C	-6.246335000000	-5.316485000000	-0.560518000000
C	-4.905923000000	-4.897470000000	0.059926000000
O	-4.356198000000	-3.756188000000	-0.603677000000
C	-3.875794000000	-6.016645000000	-0.038355000000
H	-7.364455000000	-3.809674000000	0.442409000000
H	-6.124704000000	-5.537699000000	-1.623910000000
H	-5.080126000000	-4.654185000000	1.119416000000
H	-5.051403000000	-3.074828000000	-0.694631000000
H	-2.922296000000	-5.693223000000	0.391804000000
H	-4.213373000000	-6.907922000000	0.502132000000
H	-3.703748000000	-6.286473000000	-1.086767000000
C	0.433025000000	3.269054000000	-6.510268000000
C	1.119354000000	1.940908000000	-6.167003000000
C	0.154015000000	0.799761000000	-5.787518000000
C	-0.716907000000	0.351398000000	-6.971000000000
C	0.924201000000	-0.391263000000	-5.198348000000
H	-0.189136000000	3.609620000000	-5.673699000000
H	1.744767000000	1.613554000000	-7.011666000000
H	1.807458000000	2.106394000000	-5.326387000000
H	-0.513802000000	1.188539000000	-5.003245000000
H	1.490455000000	-0.104254000000	-4.304556000000
H	0.243311000000	-1.203622000000	-4.915393000000
H	1.637366000000	-0.797260000000	-5.928716000000
H	-1.318294000000	1.172413000000	-7.375910000000
H	-0.093516000000	-0.040756000000	-7.786093000000
H	-1.408433000000	-0.445746000000	-6.671546000000
O	-2.459742000000	3.745014000000	-0.708129000000
H	-1.793530000000	3.405178000000	-0.073300000000
H	-2.019932000000	3.651964000000	-1.594154000000
O	-1.310823000000	3.122960000000	-3.114553000000
H	-0.719157000000	2.421877000000	-2.774209000000
H	-2.103353000000	2.651909000000	-3.454039000000
H	-6.603594000000	-6.229445000000	-0.061498000000
H	-0.209565000000	3.183132000000	-7.394055000000
H	-2.930965000000	8.890681000000	2.450456000000
H	1.173298000000	4.050336000000	-6.721394000000
H	-4.425233000000	3.885836000000	4.944430000000
H	-9.098420000000	0.041753000000	2.158841000000
H	-5.360443000000	4.717605000000	0.304760000000

^{4}I3$_{B}$

C	2.527332000000	-1.803046000000	-1.318399000000
C	2.467668000000	-1.118745000000	0.044785000000
O	2.351208000000	-1.722594000000	1.106990000000
C	2.163772000000	-3.287949000000	-1.264927000000
H	1.876012000000	-1.274037000000	-2.021902000000
H	2.807825000000	-3.805486000000	-0.548986000000
H	2.322802000000	-3.734893000000	-2.253535000000
N	2.554150000000	0.251695000000	0.007614000000
C	2.796760000000	1.020952000000	1.222356000000
C	1.604832000000	1.173585000000	2.179810000000
O	1.790632000000	1.601300000000	3.310522000000
H	2.744562000000	0.690857000000	-0.882881000000
H	3.594841000000	0.561999000000	1.814184000000
N	0.387396000000	0.820483000000	1.680827000000
C	-0.806374000000	0.769841000000	2.499583000000
C	-1.363982000000	-0.641296000000	2.750213000000
O	-2.565089000000	-0.785382000000	2.986305000000
H	0.339553000000	0.477568000000	0.730795000000
H	-1.613108000000	1.359291000000	2.056122000000
H	-0.546805000000	1.219198000000	3.463383000000
N	-0.455906000000	-1.645760000000	2.734549000000
C	-0.808566000000	-3.025652000000	3.023071000000
H	0.494738000000	-1.452931000000	2.437473000000
H	-0.648704000000	-3.662289000000	2.146671000000
H	-0.204587000000	-3.408595000000	3.853624000000
H	-1.862019000000	-3.048778000000	3.304574000000
H	3.128224000000	2.023220000000	0.935811000000
H	3.555160000000	-1.700306000000	-1.699779000000
S	0.440600000000	-3.627499000000	-0.767686000000
Fe	-1.206212000000	-3.234531000000	-2.585769000000
C	-0.655720000000	0.044263000000	-3.289912000000
C	1.291439000000	-4.128127000000	-4.767005000000
C	-1.871408000000	-6.542197000000	-2.009709000000
C	-3.195297000000	-2.371162000000	0.057399000000
N	0.082932000000	-2.238097000000	-3.786560000000
C	0.083420000000	-0.883591000000	-4.012664000000
C	1.029486000000	-0.553181000000	-5.053379000000
C	1.605363000000	-1.724633000000	-5.443491000000
C	0.994718000000	-2.767132000000	-4.651520000000
N	-0.407407000000	-4.980949000000	-3.214073000000
C	0.639501000000	-5.153153000000	-4.098505000000
C	0.906946000000	-6.559291000000	-4.281288000000
C	-0.004320000000	-7.242271000000	-3.535979000000
C	-0.827860000000	-6.253350000000	-2.877102000000
N	-2.375194000000	-4.242556000000	-1.300347000000
C	-2.557419000000	-5.599818000000	-1.243728000000
C	-3.467217000000	-5.941947000000	-0.174200000000
C	-3.792130000000	-4.774759000000	0.448826000000
C	-3.112156000000	-3.718239000000	-0.259795000000
N	-1.815180000000	-1.506667000000	-1.777705000000
C	-2.613044000000	-1.338188000000	-0.679220000000
C	-2.816396000000	0.062844000000	-0.415318000000
C	-2.138744000000	0.743126000000	-1.388125000000
C	-1.505606000000	-0.247724000000	-2.223536000000
H	-0.532700000000	1.091503000000	-3.551441000000
H	2.066903000000	-4.410541000000	-5.472932000000

H	-2.133673000000	-7.584597000000	-1.851917000000
H	-3.755507000000	-2.089000000000	0.942830000000
H	-0.078174000000	-8.317148000000	-3.416441000000
H	1.691305000000	-6.964859000000	-4.907406000000
H	-3.784410000000	-6.947053000000	0.068522000000
H	-4.423584000000	-4.624890000000	1.315450000000
H	2.361655000000	-1.885127000000	-6.201311000000
H	1.219661000000	0.446622000000	-5.423134000000
H	-2.063495000000	1.813809000000	-1.531887000000
H	-3.399990000000	0.448998000000	0.409760000000
O	-2.386513000000	-3.174984000000	-3.983183000000
C	-3.810606000000	-3.164004000000	-3.914294000000
C	-8.020083000000	-1.037673000000	-2.956615000000
C	-5.549843000000	-1.477050000000	-4.641604000000
C	-4.454743000000	-1.800879000000	-3.886959000000
C	-4.280491000000	-3.967222000000	-5.102540000000
C	-5.378470000000	-3.634538000000	-5.848953000000
C	-6.057328000000	-2.424084000000	-5.589600000000
H	-6.592147000000	0.030855000000	-6.730069000000
H	-6.596775000000	1.595161000000	-5.891566000000
C	-5.625254000000	0.821497000000	-3.493124000000
C	-6.135210000000	0.600917000000	-5.915617000000
C	-6.264054000000	-0.110603000000	-4.548222000000
C	-7.734871000000	-0.349303000000	-4.146816000000
C	-8.827870000000	0.113914000000	-4.892445000000
C	-10.144747000000	-0.087170000000	-4.474545000000
C	-10.400832000000	-0.760738000000	-3.280012000000
C	-9.327696000000	-1.241825000000	-2.520577000000
H	-8.665032000000	0.649991000000	-5.821396000000
H	-10.980294000000	0.283264000000	-5.060548000000
O	-11.708999000000	-0.930572000000	-2.904876000000
H	-9.514419000000	-1.767219000000	-1.584248000000
H	-7.200888000000	-1.429181000000	-2.359257000000
O	-3.525681000000	-5.071459000000	-5.366840000000
H	-4.111657000000	-3.709118000000	-2.996348000000
H	-5.687148000000	-4.278654000000	-6.668302000000
H	-6.939827000000	-2.179849000000	-6.171912000000
H	-2.662481000000	-4.879652000000	-4.933440000000
H	-4.035337000000	-1.104432000000	-3.172841000000
H	-11.729889000000	-1.302860000000	-2.009732000000
H	-5.075090000000	0.728296000000	-6.157876000000
H	-5.644407000000	0.387385000000	-2.489173000000
H	-4.582417000000	1.043700000000	-3.744840000000
H	-6.176409000000	1.767517000000	-3.458005000000
C	-11.878364000000	-8.475169000000	-3.316510000000
C	-10.397296000000	-8.155829000000	-3.351031000000
O	-9.544081000000	-9.023413000000	-3.131341000000
H	-12.125960000000	-9.083467000000	-4.193613000000
H	-12.094281000000	-9.078737000000	-2.431009000000
N	-10.055624000000	-6.865500000000	-3.620898000000
C	-8.681336000000	-6.474828000000	-3.965172000000
C	-7.691672000000	-6.676864000000	-2.811750000000
O	-6.495254000000	-6.860418000000	-3.038190000000
C	-8.657902000000	-5.016359000000	-4.438105000000
H	-10.791627000000	-6.228924000000	-3.899531000000
H	-8.307065000000	-7.127307000000	-4.762634000000
H	-9.275549000000	-4.897119000000	-5.335405000000

H	-9.023780000000	-4.331393000000	-3.664752000000
H	-7.636355000000	-4.723525000000	-4.691702000000
N	-8.164920000000	-6.629830000000	-1.539719000000
C	-7.267279000000	-6.910051000000	-0.431881000000
C	-6.766792000000	-8.362486000000	-0.360322000000
O	-5.747759000000	-8.615389000000	0.291789000000
H	-9.153920000000	-6.494454000000	-1.386806000000
H	-7.781234000000	-6.678367000000	0.506071000000
H	-6.378521000000	-6.278200000000	-0.498453000000
N	-7.525546000000	-9.297863000000	-0.973285000000
C	-7.203169000000	-10.712773000000	-0.920990000000
C	-6.654589000000	-11.367362000000	-2.200149000000
O	-5.897724000000	-12.330601000000	-2.101453000000
H	-8.320554000000	-9.004760000000	-1.533779000000
H	-6.461510000000	-10.862287000000	-0.137156000000
N	-7.103590000000	-10.903723000000	-3.396312000000
C	-6.904904000000	-11.694240000000	-4.609954000000
C	-5.438131000000	-11.829468000000	-5.051873000000
O	-4.873389000000	-12.919994000000	-5.086585000000
H	-7.776677000000	-10.146080000000	-3.430414000000
H	-7.267641000000	-12.715481000000	-4.462776000000
N	-4.817545000000	-10.670773000000	-5.414716000000
C	-3.407151000000	-10.721779000000	-5.785339000000
C	-2.501572000000	-11.118374000000	-4.608987000000
O	-1.423299000000	-11.690636000000	-4.829215000000
C	-3.001401000000	-9.389647000000	-6.448902000000
O	-3.778271000000	-9.221367000000	-7.652210000000
C	-1.537284000000	-9.309468000000	-6.855482000000
H	-5.312674000000	-9.763373000000	-5.346250000000
H	-3.259750000000	-11.522430000000	-6.517711000000
H	-3.243714000000	-8.567801000000	-5.757206000000
H	-4.654382000000	-9.616816000000	-7.500673000000
H	-1.376666000000	-8.386418000000	-7.421794000000
H	-1.264653000000	-10.157767000000	-7.489799000000
H	-0.877532000000	-9.301696000000	-5.984753000000
N	-2.950052000000	-10.846994000000	-3.371480000000
C	-2.215366000000	-11.209671000000	-2.165182000000
C	-1.897576000000	-12.716605000000	-2.109532000000
O	-0.857492000000	-13.115245000000	-1.577570000000
C	-3.048408000000	-10.785430000000	-0.930436000000
O	-3.578692000000	-9.493306000000	-1.239603000000
C	-2.208178000000	-10.763421000000	0.343531000000
H	-3.780107000000	-10.281573000000	-3.236842000000
H	-1.247361000000	-10.698167000000	-2.158253000000
H	-3.877858000000	-11.498247000000	-0.825278000000
H	-4.288053000000	-9.276248000000	-0.599455000000
H	-2.837448000000	-10.508145000000	1.203477000000
H	-1.741995000000	-11.736066000000	0.528132000000
H	-1.416892000000	-10.010525000000	0.260225000000
N	-2.810962000000	-13.557645000000	-2.648913000000
C	-2.584339000000	-14.993179000000	-2.649862000000
C	-1.422336000000	-15.495720000000	-3.528775000000
O	-0.981178000000	-16.622651000000	-3.355966000000
H	-3.659674000000	-13.195724000000	-3.073654000000
H	-2.366495000000	-15.347624000000	-1.638855000000
N	-0.972062000000	-14.628378000000	-4.486362000000
C	0.196678000000	-14.908862000000	-5.314256000000

C	1.344078000000	-13.907553000000	-5.038626000000
O	2.195069000000	-13.657424000000	-5.888588000000
C	-0.199561000000	-14.939306000000	-6.822079000000
O	-1.420255000000	-15.648650000000	-6.989271000000
C	0.828368000000	-15.640930000000	-7.701344000000
H	-1.334378000000	-13.678524000000	-4.508354000000
H	0.532224000000	-15.910682000000	-5.022255000000
H	-0.318389000000	-13.894947000000	-7.157431000000
H	-2.026186000000	-15.332537000000	-6.298793000000
H	0.454541000000	-15.681703000000	-8.730063000000
H	0.973907000000	-16.670981000000	-7.354728000000
H	1.780273000000	-15.110439000000	-7.675655000000
N	1.349352000000	-13.335943000000	-3.799740000000
C	2.365376000000	-12.376491000000	-3.393534000000
C	1.747873000000	-11.148175000000	-2.709027000000
O	0.854298000000	-10.455798000000	-3.584803000000
C	2.821598000000	-10.155069000000	-2.279982000000
H	0.650887000000	-13.605659000000	-3.114869000000
H	2.903347000000	-12.081700000000	-4.298025000000
H	1.194439000000	-11.492692000000	-1.821703000000
H	0.244325000000	-11.102088000000	-3.992417000000
H	2.363237000000	-9.279602000000	-1.808500000000
H	3.511960000000	-10.611521000000	-1.561984000000
H	3.395279000000	-9.813707000000	-3.149475000000
C	-5.168305000000	-6.091010000000	-10.997238000000
C	-3.994153000000	-6.767705000000	-11.715921000000
C	-3.808224000000	-8.267004000000	-11.404674000000
C	-5.006785000000	-9.113237000000	-11.862020000000
C	-2.510685000000	-8.790801000000	-12.039041000000
H	-5.081877000000	-6.184475000000	-9.907834000000
H	-4.108318000000	-6.644580000000	-12.803957000000
H	-3.066536000000	-6.241445000000	-11.449075000000
H	-3.714838000000	-8.377877000000	-10.313746000000
H	-1.639086000000	-8.221633000000	-11.693518000000
H	-2.341951000000	-9.845164000000	-11.789718000000
H	-2.547826000000	-8.710077000000	-13.133794000000
H	-5.937508000000	-8.809299000000	-11.370242000000
H	-5.157923000000	-9.023329000000	-12.946389000000
H	-4.845097000000	-10.174565000000	-11.637869000000
O	-6.216760000000	-8.217230000000	-5.462736000000
H	-6.135660000000	-7.730125000000	-4.615500000000
H	-5.828080000000	-7.602650000000	-6.140912000000
O	-4.876878000000	-6.686461000000	-7.283809000000
H	-4.249701000000	-6.164750000000	-6.743623000000
H	-4.359406000000	-7.440015000000	-7.641276000000
H	3.084693000000	-12.846436000000	-2.706707000000
H	-6.131539000000	-6.521540000000	-11.295085000000
H	-12.516314000000	-7.586601000000	-3.315805000000
H	-5.203652000000	-5.021124000000	-11.235750000000
H	-8.103110000000	-11.275140000000	-0.639679000000
H	-3.503761000000	-15.478502000000	-2.988601000000
H	-7.482957000000	-11.211635000000	-5.403093000000

[4]PB

C	2.648484000000	-1.093107000000	-0.103657000000
C	2.536911000000	-0.863904000000	1.399508000000
O	3.113952000000	-1.578026000000	2.210276000000
C	2.074307000000	-2.467406000000	-0.480766000000
H	2.147279000000	-0.305235000000	-0.677127000000
H	2.610093000000	-3.241600000000	0.076440000000
H	2.218058000000	-2.656649000000	-1.547694000000
N	1.722271000000	0.175583000000	1.778404000000
C	1.415174000000	0.454788000000	3.165583000000
C	-0.020416000000	0.143667000000	3.616254000000
O	-0.459641000000	0.645188000000	4.643901000000
H	1.247670000000	0.701002000000	1.058054000000
H	2.101133000000	-0.153290000000	3.763626000000
N	-0.725854000000	-0.726784000000	2.830770000000
C	-2.124462000000	-1.013982000000	3.092103000000
C	-2.492971000000	-2.498202000000	3.205441000000
O	-3.671180000000	-2.846031000000	3.107749000000
H	-0.324861000000	-1.036095000000	1.946850000000
H	-2.765760000000	-0.591846000000	2.311444000000
H	-2.377940000000	-0.521150000000	4.035916000000
N	-1.462344000000	-3.342252000000	3.448768000000
C	-1.623808000000	-4.781511000000	3.537646000000
H	-0.529025000000	-2.953588000000	3.419557000000
H	-1.243970000000	-5.281832000000	2.638892000000
H	-1.094399000000	-5.171366000000	4.413686000000
H	-2.690034000000	-4.989650000000	3.636647000000
H	1.598546000000	1.505542000000	3.411984000000
H	3.716096000000	-1.071114000000	-0.359210000000
S	0.287673000000	-2.646436000000	-0.064559000000
Fe	-1.053810000000	-2.533000000000	-2.104151000000
C	-0.955761000000	0.874953000000	-2.546373000000
C	1.425022000000	-2.887765000000	-4.452357000000
C	-1.474703000000	-5.935375000000	-2.059531000000
C	-3.826788000000	-2.171037000000	-0.116048000000
N	0.018350000000	-1.273832000000	-3.244764000000
C	-0.028017000000	0.107129000000	-3.231701000000
C	1.003220000000	0.649596000000	-4.073706000000
C	1.672065000000	-0.408944000000	-4.612678000000
C	1.050808000000	-1.599554000000	-4.100154000000
N	-0.243489000000	-4.080795000000	-3.100969000000
C	0.804585000000	-4.039908000000	-3.996154000000
C	1.181097000000	-5.371424000000	-4.390420000000
C	0.366200000000	-6.233193000000	-3.721669000000
C	-0.512774000000	-5.423946000000	-2.915082000000
N	-2.409232000000	-3.784680000000	-1.312927000000
C	-2.336418000000	-5.161888000000	-1.296295000000
C	-3.285342000000	-5.703396000000	-0.359421000000
C	-3.936876000000	-4.642342000000	0.198188000000
C	-3.395862000000	-3.455523000000	-0.406904000000
N	-2.216043000000	-0.976831000000	-1.536473000000
C	-3.289782000000	-1.020052000000	-0.672001000000
C	-3.752294000000	0.310678000000	-0.380156000000
C	-2.942296000000	1.168634000000	-1.062866000000
C	-1.982869000000	0.362288000000	-1.768410000000
H	-0.882444000000	1.954119000000	-2.637952000000
H	2.250588000000	-3.001397000000	-5.147574000000

H	-1.566756000000	-7.012716000000	-1.968297000000
H	-4.606695000000	-2.062389000000	0.629064000000
H	0.372711000000	-7.315864000000	-3.780596000000
H	1.969862000000	-5.609154000000	-5.092418000000
H	-3.397426000000	-6.758089000000	-0.148067000000
H	-4.679405000000	-4.630914000000	0.984963000000
H	2.504732000000	-0.399295000000	-5.304401000000
H	1.173083000000	1.706676000000	-4.234444000000
H	-2.977900000000	2.250281000000	-1.092629000000
H	-4.589486000000	0.542045000000	0.266056000000
O	-4.364123000000	-5.255743000000	-4.630172000000
C	-5.623196000000	-4.758556000000	-4.149831000000
C	-5.101949000000	-0.568837000000	-7.855161000000
C	-4.730596000000	-3.161987000000	-5.965565000000
C	-4.558851000000	-3.821100000000	-4.645846000000
C	-6.790115000000	-4.976305000000	-5.025251000000
C	-6.818000000000	-4.454636000000	-6.271464000000
C	-5.782517000000	-3.552738000000	-6.730063000000
H	-2.303374000000	-3.675930000000	-7.124737000000
H	-1.519283000000	-2.121121000000	-6.756259000000
C	-3.586226000000	-0.988578000000	-5.327417000000
C	-2.302200000000	-2.820667000000	-6.441680000000
C	-3.682584000000	-2.117646000000	-6.393391000000
C	-4.042825000000	-1.485843000000	-7.751616000000
C	-3.352925000000	-1.788141000000	-8.936102000000
C	-3.687272000000	-1.205198000000	-10.158050000000
C	-4.739732000000	-0.290796000000	-10.229971000000
C	-5.451016000000	0.023434000000	-9.067502000000
H	-2.528504000000	-2.491640000000	-8.921527000000
H	-3.138310000000	-1.449249000000	-11.062189000000
O	-5.028179000000	0.257622000000	-11.449337000000
H	-6.277551000000	0.731470000000	-9.107886000000
H	-5.683778000000	-0.312686000000	-6.975094000000
O	-7.851215000000	-5.708610000000	-4.543285000000
H	-5.770176000000	-4.888548000000	-3.076212000000
H	-7.667275000000	-4.655899000000	-6.918380000000
H	-5.904519000000	-3.136299000000	-7.723728000000
H	-7.616790000000	-6.151856000000	-3.696924000000
H	-3.952018000000	-3.325473000000	-3.892446000000
H	-5.773310000000	0.870548000000	-11.350610000000
H	-2.030351000000	-3.195844000000	-5.450402000000
H	-4.551649000000	-0.505913000000	-5.148582000000
H	-3.221710000000	-1.366745000000	-4.367537000000
H	-2.879589000000	-0.222758000000	-5.664779000000
C	-11.627240000000	-10.351871000000	0.095920000000
C	-10.466735000000	-9.665835000000	-0.595678000000
O	-9.433357000000	-10.264300000000	-0.893300000000
H	-12.180562000000	-10.939269000000	-0.646060000000
H	-11.239052000000	-11.048444000000	0.842533000000
N	-10.624933000000	-8.329895000000	-0.869564000000
C	-9.775914000000	-7.634773000000	-1.843015000000
C	-8.345160000000	-7.388390000000	-1.348939000000
O	-7.461996000000	-7.046659000000	-2.155845000000
C	-10.442649000000	-6.320739000000	-2.272955000000
H	-11.534039000000	-7.920771000000	-0.694622000000
H	-9.634305000000	-8.286247000000	-2.714581000000
H	-11.416487000000	-6.526004000000	-2.729817000000

H	-10.587618000000	-5.653255000000	-1.415671000000
H	-9.827201000000	-5.805604000000	-3.013776000000
N	-8.075250000000	-7.581326000000	-0.046243000000
C	-6.714489000000	-7.577925000000	0.465390000000
C	-5.959349000000	-8.915551000000	0.384777000000
O	-4.804598000000	-8.945836000000	0.819161000000
H	-8.837102000000	-7.878815000000	0.548740000000
H	-6.719527000000	-7.269740000000	1.514295000000
H	-6.131396000000	-6.843239000000	-0.094564000000
N	-6.611803000000	-9.979321000000	-0.136076000000
C	-6.030447000000	-11.315759000000	-0.109150000000
C	-5.604985000000	-11.926024000000	-1.456138000000
O	-4.566564000000	-12.586728000000	-1.517986000000
H	-7.589804000000	-9.893640000000	-0.406971000000
H	-5.143001000000	-11.286925000000	0.521726000000
N	-6.462057000000	-11.805401000000	-2.498696000000
C	-6.192859000000	-12.502195000000	-3.750688000000
C	-4.979138000000	-11.977848000000	-4.532731000000
O	-4.100886000000	-12.743381000000	-4.933755000000
H	-7.182554000000	-11.090287000000	-2.514267000000
H	-5.994713000000	-13.556477000000	-3.549096000000
N	-4.936705000000	-10.636909000000	-4.731952000000
C	-3.803397000000	-10.062986000000	-5.440910000000
C	-2.491603000000	-10.309557000000	-4.668304000000
O	-1.446634000000	-10.560115000000	-5.282562000000
C	-4.060296000000	-8.570267000000	-5.755284000000
O	-5.227539000000	-8.471392000000	-6.592041000000
C	-2.873056000000	-7.862536000000	-6.397858000000
H	-5.756206000000	-10.071020000000	-4.486569000000
H	-3.654498000000	-10.593356000000	-6.389482000000
H	-4.338480000000	-8.042915000000	-4.836720000000
H	-4.990385000000	-8.758316000000	-7.489590000000
H	-3.155285000000	-6.827194000000	-6.604383000000
H	-2.576609000000	-8.357616000000	-7.330839000000
H	-2.000179000000	-7.858015000000	-5.739450000000
N	-2.561498000000	-10.266229000000	-3.324785000000
C	-1.416145000000	-10.508133000000	-2.453300000000
C	-0.818130000000	-11.919376000000	-2.648661000000
O	0.401896000000	-12.093078000000	-2.626003000000
C	-1.877127000000	-10.309004000000	-0.988409000000
O	-2.715470000000	-9.149692000000	-0.987388000000
C	-0.694204000000	-10.154817000000	-0.036842000000
H	-3.397676000000	-9.924468000000	-2.867212000000
H	-0.606695000000	-9.811984000000	-2.697505000000
H	-2.478994000000	-11.183570000000	-0.704860000000
H	-3.306935000000	-9.179166000000	-0.209154000000
H	-1.048562000000	-10.075983000000	0.996785000000
H	-0.013449000000	-11.009388000000	-0.107057000000
H	-0.132520000000	-9.245933000000	-0.277919000000
N	-1.705093000000	-12.927030000000	-2.830541000000
C	-1.266131000000	-14.284233000000	-3.112918000000
C	-0.711857000000	-14.538959000000	-4.530659000000
O	-0.235088000000	-15.632408000000	-4.803505000000
H	-2.701885000000	-12.737584000000	-2.821697000000
H	-0.470832000000	-14.569381000000	-2.418806000000
N	-0.774454000000	-13.479291000000	-5.380578000000
C	-0.228647000000	-13.471761000000	-6.725469000000

C	0.955963000000	-12.479538000000	-6.848390000000
O	1.364861000000	-12.102983000000	-7.944689000000
C	-1.349267000000	-13.139488000000	-7.768316000000
O	-2.566418000000	-13.789592000000	-7.438117000000
C	-0.984997000000	-13.597327000000	-9.175439000000
H	-1.197724000000	-12.616641000000	-5.062180000000
H	0.144269000000	-14.485003000000	-6.910462000000
H	-1.485694000000	-12.045584000000	-7.762484000000
H	-2.861828000000	-13.489364000000	-6.561362000000
H	-1.796332000000	-13.341212000000	-9.865485000000
H	-0.862686000000	-14.686957000000	-9.187865000000
H	-0.057812000000	-13.124664000000	-9.501492000000
N	1.494127000000	-12.049322000000	-5.671685000000
C	2.560910000000	-11.062514000000	-5.607606000000
C	2.291119000000	-10.012519000000	-4.518551000000
O	1.098186000000	-9.272516000000	-4.786173000000
C	3.433947000000	-9.007362000000	-4.427635000000
H	1.142553000000	-12.425704000000	-4.798631000000
H	2.621680000000	-10.590181000000	-6.591818000000
H	2.187178000000	-10.533802000000	-3.555268000000
H	0.377209000000	-9.896783000000	-4.998167000000
H	3.217194000000	-8.256012000000	-3.661136000000
H	4.374110000000	-9.504936000000	-4.165077000000
H	3.567123000000	-8.492094000000	-5.386317000000
C	-8.224473000000	-7.691497000000	-9.843782000000
C	-7.181930000000	-6.701913000000	-10.380116000000
C	-5.763764000000	-6.861219000000	-9.795940000000
C	-5.163460000000	-8.246783000000	-10.085993000000
C	-4.834525000000	-5.756311000000	-10.320394000000
H	-8.271160000000	-7.678213000000	-8.747713000000
H	-7.125327000000	-6.786001000000	-11.475875000000
H	-7.522512000000	-5.677175000000	-10.174120000000
H	-5.838021000000	-6.746117000000	-8.703496000000
H	-5.225034000000	-4.758776000000	-10.087257000000
H	-3.834050000000	-5.833175000000	-9.877875000000
H	-4.721655000000	-5.823848000000	-11.410524000000
H	-5.766286000000	-9.059393000000	-9.661214000000
H	-5.095267000000	-8.430024000000	-11.166188000000
H	-4.143870000000	-8.327878000000	-9.685945000000
O	-7.421266000000	-9.406623000000	-3.827502000000
H	-7.187556000000	-8.620818000000	-3.301518000000
H	-7.772572000000	-9.025788000000	-4.681935000000
O	-7.976660000000	-8.101075000000	-6.097923000000
H	-8.080816000000	-7.191837000000	-5.752309000000
H	-7.036112000000	-8.150890000000	-6.375679000000
H	3.526509000000	-11.550093000000	-5.406390000000
H	-8.006728000000	-8.720293000000	-10.152836000000
H	-12.321593000000	-9.653562000000	0.572790000000
H	-9.223467000000	-7.446048000000	-10.223084000000
H	-6.755853000000	-12.002260000000	0.345188000000
H	-2.111271000000	-14.958359000000	-2.955368000000
H	-7.090345000000	-12.421348000000	-4.371076000000

^{2}TS4$_B$

C	3.747670000000	-5.702210000000	0.810200000000
C	4.927228000000	-5.747455000000	1.775088000000
O	4.778564000000	-5.706423000000	2.995024000000
C	2.815072000000	-4.534675000000	1.154203000000
H	4.075607000000	-5.649743000000	-0.233645000000
H	2.446441000000	-4.675078000000	2.174786000000
H	1.956186000000	-4.511838000000	0.481681000000
N	6.167605000000	-5.826225000000	1.204571000000
C	7.367705000000	-5.910699000000	2.018079000000
C	7.873707000000	-4.604295000000	2.652156000000
O	8.858619000000	-4.633529000000	3.379102000000
H	6.249532000000	-5.788363000000	0.199142000000
H	7.184184000000	-6.598289000000	2.848310000000
N	7.167632000000	-3.477836000000	2.357491000000
C	7.432819000000	-2.206530000000	2.997814000000
C	6.304195000000	-1.677153000000	3.894137000000
O	6.243655000000	-0.473532000000	4.144478000000
H	6.363004000000	-3.539724000000	1.742748000000
H	7.633635000000	-1.431003000000	2.253709000000
H	8.335005000000	-2.335917000000	3.603904000000
N	5.459792000000	-2.610631000000	4.402086000000
C	4.318310000000	-2.247480000000	5.221883000000
H	5.504460000000	-3.556942000000	4.040614000000
H	3.388051000000	-2.246388000000	4.639204000000
H	4.213692000000	-2.950208000000	6.054626000000
H	4.489978000000	-1.242873000000	5.610937000000
H	8.182117000000	-6.326156000000	1.418229000000
H	3.189551000000	-6.640726000000	0.936661000000
S	3.657640000000	-2.898799000000	1.152690000000
Fe	3.229419000000	-1.715685000000	-0.695653000000
C	5.966210000000	-2.756730000000	-2.390828000000
C	1.306590000000	-4.085106000000	-2.254168000000
C	0.441636000000	-0.498518000000	0.891373000000
C	5.172387000000	0.583033000000	1.033363000000
N	3.563128000000	-3.144247000000	-2.037750000000
C	4.772122000000	-3.432091000000	-2.621528000000
C	4.633016000000	-4.549688000000	-3.523423000000
C	3.325803000000	-4.932760000000	-3.479108000000
C	2.664589000000	-4.044130000000	-2.556724000000
N	1.273286000000	-2.162928000000	-0.736479000000
C	0.658994000000	-3.204077000000	-1.401465000000
C	-0.746928000000	-3.254128000000	-1.075565000000
C	-0.973978000000	-2.228322000000	-0.212518000000
C	0.285491000000	-1.556199000000	-0.004793000000
N	2.840739000000	-0.008051000000	0.477097000000
C	1.625372000000	0.142302000000	1.193789000000
C	1.842160000000	0.968765000000	2.334183000000
C	3.184955000000	1.245370000000	2.404231000000
C	3.818946000000	0.617369000000	1.295187000000
N	5.157135000000	-1.124679000000	-0.744258000000
C	5.784072000000	-0.197277000000	0.048360000000
C	7.198483000000	-0.165928000000	-0.240987000000
C	7.419235000000	-1.088087000000	-1.219178000000
C	6.144142000000	-1.698928000000	-1.509824000000
H	6.841204000000	-3.100940000000	-2.934308000000
H	0.708386000000	-4.858019000000	-2.726414000000

H	-0.436076000000	-0.219738000000	1.468468000000
H	5.819872000000	1.152523000000	1.692132000000
H	-1.903451000000	-1.945785000000	0.257144000000
H	-1.465875000000	-3.980224000000	-1.433006000000
H	1.063635000000	1.247295000000	3.034814000000
H	3.716112000000	1.778208000000	3.182076000000
H	2.836931000000	-5.728943000000	-4.025764000000
H	5.437526000000	-4.968723000000	-4.114617000000
H	8.354766000000	-1.353966000000	-1.694568000000
H	7.913488000000	0.484417000000	0.246584000000
O	2.821503000000	-0.357691000000	-2.359245000000
C	2.807201000000	1.014805000000	-1.994377000000
C	5.114360000000	4.579965000000	0.273351000000
C	3.964697000000	3.159089000000	-2.097343000000
C	4.007817000000	1.783934000000	-2.151591000000
C	1.505857000000	1.635512000000	-2.168071000000
C	1.495395000000	3.066549000000	-2.025036000000
C	2.672817000000	3.771035000000	-2.003238000000
H	4.237518000000	5.425296000000	-3.675527000000
H	6.010548000000	5.412741000000	-3.762836000000
C	6.521474000000	3.269365000000	-2.193391000000
C	5.125337000000	4.790114000000	-3.588764000000
C	5.199801000000	4.071187000000	-2.219662000000
C	5.222616000000	5.065640000000	-1.039665000000
C	5.390606000000	6.448863000000	-1.196692000000
C	5.454560000000	7.311772000000	-0.101275000000
C	5.355269000000	6.802070000000	1.194300000000
C	5.181420000000	5.425817000000	1.377772000000
H	5.478965000000	6.879036000000	-2.188715000000
H	5.589808000000	8.380369000000	-0.238126000000
O	5.422131000000	7.687008000000	2.238098000000
H	5.097006000000	5.016316000000	2.383646000000
H	4.964003000000	3.516343000000	0.437076000000
O	0.500173000000	0.904794000000	-2.415605000000
H	2.781752000000	0.716347000000	-0.579151000000
H	0.535488000000	3.572413000000	-2.039126000000
H	2.630616000000	4.856221000000	-1.936917000000
H	1.876160000000	-0.544254000000	-2.594541000000
H	4.939575000000	1.242189000000	-2.260726000000
H	5.428981000000	7.188805000000	3.070277000000
H	5.078602000000	4.044810000000	-4.389168000000
H	6.628185000000	2.686729000000	-1.273629000000
H	6.586213000000	2.581081000000	-3.044054000000
H	7.370317000000	3.958460000000	-2.254673000000
C	-3.898570000000	8.742198000000	1.658425000000
C	-3.242779000000	7.422344000000	1.307687000000
O	-3.760889000000	6.341127000000	1.608121000000
H	-4.761094000000	8.891210000000	0.998994000000
H	-4.276526000000	8.693556000000	2.682787000000
N	-2.048039000000	7.486392000000	0.656104000000
C	-1.443293000000	6.313646000000	0.011820000000
C	-1.042398000000	5.216562000000	1.006168000000
O	-0.962782000000	4.043208000000	0.642376000000
C	-0.228477000000	6.750836000000	-0.816340000000
H	-1.726980000000	8.394560000000	0.345273000000
H	-2.178908000000	5.831398000000	-0.643114000000
H	-0.533948000000	7.442257000000	-1.609191000000

H	0.528865000000	7.236959000000	-0.190767000000
H	0.225821000000	5.876753000000	-1.287151000000
N	-0.763838000000	5.570122000000	2.286631000000
C	-0.471572000000	4.529253000000	3.257464000000
C	-1.651881000000	3.590623000000	3.566285000000
O	-1.419661000000	2.472884000000	4.035747000000
H	-0.836515000000	6.538387000000	2.564146000000
H	-0.153224000000	4.999358000000	4.192912000000
H	0.342686000000	3.897453000000	2.896625000000
N	-2.890849000000	4.097992000000	3.367050000000
C	-4.099673000000	3.377735000000	3.723224000000
C	-5.011105000000	2.883773000000	2.588109000000
O	-5.741484000000	1.915851000000	2.801783000000
H	-2.984792000000	5.012027000000	2.934444000000
H	-3.812562000000	2.506072000000	4.309974000000
N	-5.039657000000	3.582427000000	1.427155000000
C	-6.110151000000	3.350110000000	0.457230000000
C	-6.088059000000	1.956657000000	-0.191998000000
O	-7.053545000000	1.200332000000	-0.105053000000
H	-4.477439000000	4.419757000000	1.320886000000
H	-7.088274000000	3.443501000000	0.936928000000
N	-4.949034000000	1.632669000000	-0.862191000000
C	-4.826397000000	0.288525000000	-1.415985000000
C	-4.740049000000	-0.777371000000	-0.312031000000
O	-5.186814000000	-1.913877000000	-0.520551000000
C	-3.653536000000	0.239025000000	-2.417305000000
O	-3.929599000000	1.154297000000	-3.492244000000
C	-3.435107000000	-1.122288000000	-3.059333000000
H	-4.160379000000	2.300904000000	-0.923872000000
H	-5.743450000000	0.044036000000	-1.961202000000
H	-2.734823000000	0.556114000000	-1.900510000000
H	-4.341596000000	1.948324000000	-3.110499000000
H	-2.637146000000	-1.035649000000	-3.805453000000
H	-4.347546000000	-1.464162000000	-3.556750000000
H	-3.136445000000	-1.871707000000	-2.323902000000
N	-4.205636000000	-0.412757000000	0.869058000000
C	-4.106574000000	-1.322045000000	2.006851000000
C	-5.483135000000	-1.887439000000	2.428635000000
O	-5.599873000000	-3.068565000000	2.758598000000
C	-3.437555000000	-0.575375000000	3.190971000000
O	-2.429467000000	0.267853000000	2.625348000000
C	-2.861592000000	-1.554961000000	4.209840000000
H	-3.763455000000	0.491857000000	0.979246000000
H	-3.513851000000	-2.200253000000	1.730116000000
H	-4.203226000000	0.053741000000	3.664345000000
H	-2.125239000000	0.919848000000	3.290145000000
H	-2.457162000000	-1.014050000000	5.072735000000
H	-3.630569000000	-2.249590000000	4.563620000000
H	-2.051814000000	-2.136817000000	3.756727000000
N	-6.516167000000	-1.010236000000	2.416864000000
C	-7.874771000000	-1.427289000000	2.726306000000
C	-8.620818000000	-2.198735000000	1.617102000000
O	-9.751834000000	-2.614834000000	1.828481000000
H	-6.352644000000	-0.037722000000	2.172962000000
H	-7.869683000000	-2.079492000000	3.604150000000
N	-7.919197000000	-2.384358000000	0.467567000000
C	-8.394329000000	-3.143981000000	-0.673775000000

C	-7.518987000000	-4.398389000000	-0.924020000000
O	-7.584753000000	-5.021247000000	-1.981384000000
C	-8.446754000000	-2.237973000000	-1.952745000000
O	-8.914821000000	-0.934037000000	-1.642921000000
C	-9.390345000000	-2.790759000000	-3.013858000000
H	-6.984287000000	-2.002807000000	0.393207000000
H	-9.409477000000	-3.471078000000	-0.424815000000
H	-7.424452000000	-2.190265000000	-2.361451000000
H	-8.305576000000	-0.510021000000	-1.015155000000
H	-9.385126000000	-2.131579000000	-3.888576000000
H	-10.412613000000	-2.819758000000	-2.618150000000
H	-9.086093000000	-3.796534000000	-3.306131000000
N	-6.672615000000	-4.746150000000	0.087382000000
C	-5.727292000000	-5.846652000000	-0.017172000000
C	-4.328482000000	-5.441929000000	0.478018000000
O	-3.766118000000	-4.399926000000	-0.318091000000
C	-3.362784000000	-6.620394000000	0.424167000000
H	-6.694415000000	-4.219005000000	0.952314000000
H	-5.691446000000	-6.139015000000	-1.069918000000
H	-4.420718000000	-5.097020000000	1.520321000000
H	-4.409725000000	-3.667489000000	-0.367314000000
H	-2.368990000000	-6.306375000000	0.760164000000
H	-3.703142000000	-7.438486000000	1.068498000000
H	-3.275363000000	-6.996564000000	-0.602000000000
C	0.671286000000	2.615231000000	-6.420213000000
C	1.343180000000	1.241089000000	-6.301579000000
C	0.364442000000	0.063449000000	-6.070719000000
C	-0.613007000000	-0.148125000000	-7.237649000000
C	1.129433000000	-1.236770000000	-5.793843000000
H	0.064181000000	2.837412000000	-5.536435000000
H	1.941819000000	1.043589000000	-7.204666000000
H	2.065437000000	1.268288000000	-5.454465000000
H	-0.220635000000	0.316892000000	-5.173305000000
H	1.766036000000	-1.142449000000	-4.918252000000
H	0.439287000000	-2.074142000000	-5.630159000000
H	1.778783000000	-1.503600000000	-6.638359000000
H	-1.229927000000	0.737449000000	-7.422734000000
H	-0.070329000000	-0.377993000000	-8.164893000000
H	-1.292602000000	-0.985179000000	-7.036229000000
O	-2.872706000000	3.510912000000	-1.329155000000
H	-2.200287000000	3.517558000000	-0.617868000000
H	-2.353324000000	3.235629000000	-2.135847000000
O	-1.496502000000	2.527410000000	-3.453991000000
H	-0.814782000000	1.902945000000	-3.093809000000
H	-2.217488000000	1.955479000000	-3.784484000000
H	-6.079781000000	-6.711897000000	0.563980000000
H	0.012670000000	2.671616000000	-7.294982000000
H	-3.229445000000	9.601607000000	1.557352000000
H	1.423148000000	3.406470000000	-6.529962000000
H	-4.723130000000	4.013032000000	4.366444000000
H	-8.462464000000	-0.538580000000	2.967842000000
H	-6.011632000000	4.115415000000	-0.318002000000

^{2}I4$_{B}$

C	3.188648000000	-3.013336000000	0.393894000000
C	4.081559000000	-1.810799000000	0.117806000000
O	4.129705000000	-0.841959000000	0.878602000000
C	1.710667000000	-2.726932000000	0.062395000000
H	3.525906000000	-3.896500000000	-0.160134000000
H	1.372480000000	-1.857845000000	0.634964000000
H	1.106902000000	-3.588409000000	0.354677000000
N	4.797915000000	-1.854109000000	-1.042462000000
C	5.674286000000	-0.767690000000	-1.445376000000
C	5.010233000000	0.575704000000	-1.793767000000
O	5.707255000000	1.577736000000	-1.892528000000
H	4.628186000000	-2.617084000000	-1.682970000000
H	6.377552000000	-0.542564000000	-0.638673000000
N	3.659216000000	0.564626000000	-1.969872000000
C	2.902723000000	1.791080000000	-2.109665000000
C	2.174253000000	2.265142000000	-0.840725000000
O	1.260340000000	3.090672000000	-0.935050000000
H	3.143314000000	-0.307651000000	-1.895045000000
H	2.153871000000	1.690132000000	-2.898915000000
H	3.604891000000	2.577790000000	-2.402088000000
N	2.628265000000	1.763659000000	0.330497000000
C	2.098708000000	2.185452000000	1.613531000000
H	3.316647000000	1.015980000000	0.328084000000
H	1.384266000000	1.456717000000	2.018430000000
H	2.917003000000	2.303535000000	2.330374000000
H	1.590130000000	3.141845000000	1.479314000000
H	6.258703000000	-1.096298000000	-2.309516000000
H	3.271442000000	-3.229752000000	1.465035000000
S	1.498382000000	-2.417374000000	-1.743021000000
Fe	-0.692067000000	-2.613181000000	-2.331468000000
C	0.294277000000	-2.759188000000	-5.585899000000
C	-0.603357000000	-5.958708000000	-2.062735000000
C	-1.871870000000	-2.360514000000	0.947510000000
C	-0.699304000000	0.863475000000	-2.509866000000
N	-0.288363000000	-4.064585000000	-3.592721000000
C	0.190540000000	-3.949071000000	-4.875587000000
C	0.597096000000	-5.243699000000	-5.367697000000
C	0.376892000000	-6.140027000000	-4.365362000000
C	-0.186285000000	-5.392731000000	-3.263534000000
N	-1.201718000000	-3.883918000000	-0.887276000000
C	-1.114878000000	-5.251943000000	-0.982360000000
C	-1.601287000000	-5.872393000000	0.228869000000
C	-1.966577000000	-4.861422000000	1.062736000000
C	-1.708545000000	-3.621736000000	0.362162000000
N	-1.402010000000	-1.007611000000	-1.056363000000
C	-1.622809000000	-1.143786000000	0.340143000000
C	-1.461637000000	0.130906000000	0.941519000000
C	-1.103847000000	1.036399000000	-0.036179000000
C	-1.049471000000	0.352605000000	-1.277238000000
N	-0.343654000000	-1.236446000000	-3.780115000000
C	-0.431663000000	0.126070000000	-3.670763000000
C	-0.115043000000	0.747068000000	-4.939772000000
C	0.165970000000	-0.258817000000	-5.811827000000
C	0.029972000000	-1.493185000000	-5.073239000000
H	0.639357000000	-2.820262000000	-6.613539000000
H	-0.539773000000	-7.038656000000	-1.955643000000

H	-2.149501000000	-2.341078000000	1.997208000000
H	-0.544035000000	1.936612000000	-2.557366000000
H	-2.375974000000	-4.926194000000	2.062868000000
H	-1.652238000000	-6.942906000000	0.374820000000
H	-1.549166000000	0.318683000000	2.004260000000
H	-0.820136000000	2.073699000000	0.084140000000
H	0.583647000000	-7.204499000000	-4.347226000000
H	1.009485000000	-5.425559000000	-6.351957000000
H	0.447434000000	-0.189385000000	-6.854729000000
H	-0.116609000000	1.814761000000	-5.118231000000
O	-2.675877000000	-2.295091000000	-2.861187000000
C	-3.823338000000	-2.993426000000	-2.769599000000
C	-7.067664000000	-6.417588000000	-0.553004000000
C	-5.818547000000	-3.831536000000	-1.605721000000
C	-4.574918000000	-3.174107000000	-1.600845000000
C	-4.347339000000	-3.497863000000	-3.980276000000
C	-5.572103000000	-4.147096000000	-4.012616000000
C	-6.302699000000	-4.311363000000	-2.830743000000
H	-8.176222000000	-2.480518000000	-1.081032000000
H	-8.052133000000	-2.509783000000	0.694563000000
C	-5.808215000000	-4.004819000000	0.956210000000
C	-7.482396000000	-2.582654000000	-0.239748000000
C	-6.680734000000	-3.905164000000	-0.322889000000
C	-7.604278000000	-5.138296000000	-0.332800000000
C	-8.979684000000	-5.065763000000	-0.070527000000
C	-9.786489000000	-6.204286000000	-0.047667000000
C	-9.232840000000	-7.463698000000	-0.290393000000
C	-7.855674000000	-7.567951000000	-0.536670000000
H	-9.451089000000	-4.105983000000	0.111984000000
H	-10.852668000000	-6.128101000000	0.145895000000
O	-10.075399000000	-8.542268000000	-0.292786000000
H	-7.397963000000	-8.537660000000	-0.718788000000
H	-6.006436000000	-6.520400000000	-0.763720000000
O	-3.607201000000	-3.301151000000	-5.121896000000
H	-2.230575000000	-1.323695000000	-1.655367000000
H	-5.951877000000	-4.521256000000	-4.958122000000
H	-7.256561000000	-4.825851000000	-2.876178000000
H	-2.832961000000	-2.776376000000	-4.824228000000
H	-4.172909000000	-2.766817000000	-0.680214000000
H	-9.644014000000	-9.305499000000	-0.730253000000
H	-6.791131000000	-1.734425000000	-0.275651000000
H	-5.122479000000	-4.856558000000	0.909511000000
H	-5.214230000000	-3.098119000000	1.115514000000
H	-6.454192000000	-4.134161000000	1.831506000000
C	-11.278414000000	-10.177166000000	-8.708431000000
C	-10.356270000000	-9.376120000000	-7.812751000000
O	-9.329530000000	-9.867591000000	-7.324223000000
H	-10.779887000000	-10.335213000000	-9.671481000000
H	-11.449649000000	-11.161110000000	-8.263738000000
N	-10.712124000000	-8.087024000000	-7.559286000000
C	-9.771702000000	-7.105377000000	-6.994981000000
C	-9.405274000000	-7.364332000000	-5.523888000000
O	-8.387648000000	-6.861788000000	-5.047971000000
C	-10.334582000000	-5.688653000000	-7.160219000000
H	-11.507734000000	-7.714630000000	-8.062221000000
H	-8.820569000000	-7.169744000000	-7.533482000000
H	-10.502405000000	-5.464368000000	-8.219158000000

H	-11.275528000000	-5.560829000000	-6.612678000000
H	-9.608299000000	-4.968918000000	-6.777576000000
N	-10.247141000000	-8.142903000000	-4.794430000000
C	-9.929980000000	-8.564422000000	-3.442892000000
C	-9.425518000000	-10.007090000000	-3.318881000000
O	-9.250690000000	-10.505068000000	-2.195409000000
H	-11.086127000000	-8.484632000000	-5.242811000000
H	-10.792628000000	-8.457757000000	-2.779785000000
H	-9.148803000000	-7.901472000000	-3.060108000000
N	-9.215294000000	-10.667265000000	-4.476196000000
C	-8.797198000000	-12.059697000000	-4.545257000000
C	-7.401023000000	-12.258513000000	-5.155307000000
O	-6.495752000000	-12.790792000000	-4.516005000000
H	-9.325443000000	-10.160863000000	-5.350935000000
H	-8.773826000000	-12.458279000000	-3.532148000000
N	-7.236629000000	-11.843645000000	-6.440142000000
C	-5.962465000000	-12.067638000000	-7.111541000000
C	-4.769615000000	-11.398163000000	-6.412436000000
O	-3.733573000000	-12.022553000000	-6.184404000000
H	-7.987651000000	-11.368294000000	-6.931926000000
H	-5.728193000000	-13.134521000000	-7.154154000000
N	-4.943806000000	-10.100879000000	-6.045905000000
C	-3.840619000000	-9.344551000000	-5.463847000000
C	-3.463399000000	-9.887844000000	-4.078188000000
O	-2.289184000000	-9.811781000000	-3.679719000000
C	-4.207850000000	-7.839135000000	-5.467160000000
O	-4.483107000000	-7.503011000000	-6.826736000000
C	-3.076724000000	-6.977302000000	-4.922426000000
H	-5.655481000000	-9.556137000000	-6.518775000000
H	-2.942014000000	-9.467226000000	-6.078423000000
H	-5.113470000000	-7.694600000000	-4.857960000000
H	-5.397332000000	-7.122324000000	-6.916149000000
H	-3.348037000000	-5.921816000000	-4.991171000000
H	-2.163197000000	-7.139210000000	-5.502928000000
H	-2.859219000000	-7.205803000000	-3.876023000000
N	-4.433292000000	-10.472203000000	-3.356056000000
C	-4.186532000000	-11.100886000000	-2.063223000000
C	-3.167693000000	-12.261928000000	-2.173548000000
O	-2.357926000000	-12.480604000000	-1.273406000000
C	-5.539777000000	-11.603217000000	-1.500217000000
O	-6.475836000000	-10.551098000000	-1.748615000000
C	-5.446086000000	-11.941283000000	-0.014995000000
H	-5.398010000000	-10.420393000000	-3.657598000000
H	-3.738509000000	-10.375753000000	-1.375353000000
H	-5.836027000000	-12.489295000000	-2.076878000000
H	-7.388768000000	-10.889257000000	-1.702334000000
H	-6.399119000000	-12.348725000000	0.340812000000
H	-4.660417000000	-12.679059000000	0.176478000000
H	-5.221603000000	-11.037938000000	0.561925000000
N	-3.245020000000	-13.013896000000	-3.303881000000
C	-2.273751000000	-14.051836000000	-3.605655000000
C	-0.952618000000	-13.585190000000	-4.256129000000
O	-0.094692000000	-14.405366000000	-4.551720000000
H	-3.895106000000	-12.750624000000	-4.036631000000
H	-2.003501000000	-14.564718000000	-2.679180000000
N	-0.833365000000	-12.237835000000	-4.413784000000
C	0.352775000000	-11.556019000000	-4.901674000000

C	0.906958000000	-10.611980000000	-3.802513000000
O	1.505741000000	-9.570412000000	-4.071264000000
C	0.052563000000	-10.771214000000	-6.217380000000
O	-0.801861000000	-11.514748000000	-7.075598000000
C	1.320787000000	-10.478472000000	-7.009987000000
H	-1.588499000000	-11.637767000000	-4.105637000000
H	1.092536000000	-12.338368000000	-5.107852000000
H	-0.425394000000	-9.821849000000	-5.930719000000
H	-1.619770000000	-11.738938000000	-6.597490000000
H	1.069785000000	-9.915993000000	-7.915289000000
H	1.793065000000	-11.419186000000	-7.316539000000
H	2.020217000000	-9.898889000000	-6.404991000000
N	0.683973000000	-11.035019000000	-2.528403000000
C	1.060633000000	-10.285739000000	-1.341795000000
C	-0.154973000000	-9.967805000000	-0.454857000000
O	-1.062960000000	-9.077570000000	-1.106452000000
C	0.276600000000	-9.307279000000	0.849507000000
H	0.128247000000	-11.870581000000	-2.397800000000
H	1.539913000000	-9.363753000000	-1.680589000000
H	-0.678264000000	-10.908319000000	-0.228819000000
H	-1.387845000000	-9.489985000000	-1.931251000000
H	-0.597078000000	-9.094729000000	1.473913000000
H	0.948575000000	-9.962639000000	1.414407000000
H	0.797894000000	-8.363023000000	0.652535000000
C	-4.231973000000	-6.552284000000	-10.897529000000
C	-3.034272000000	-7.501557000000	-11.029986000000
C	-2.936804000000	-8.594357000000	-9.945914000000
C	-4.113122000000	-9.581997000000	-10.007899000000
C	-1.601732000000	-9.347980000000	-10.049394000000
H	-4.224305000000	-6.025726000000	-9.935631000000
H	-3.053524000000	-7.983786000000	-12.019685000000
H	-2.110515000000	-6.906406000000	-11.004099000000
H	-2.979027000000	-8.099745000000	-8.965176000000
H	-0.753229000000	-8.654064000000	-9.994355000000
H	-1.485268000000	-10.082662000000	-9.243968000000
H	-1.526098000000	-9.882366000000	-11.006586000000
H	-5.073914000000	-9.085358000000	-9.828618000000
H	-4.163293000000	-10.073149000000	-10.989546000000
H	-3.990619000000	-10.368758000000	-9.252673000000
O	-6.761507000000	-6.133636000000	-7.168812000000
H	-7.189927000000	-6.088738000000	-6.291113000000
H	-6.159542000000	-5.351005000000	-7.269408000000
O	-4.541942000000	-4.633282000000	-7.459898000000
H	-4.184031000000	-4.053664000000	-6.756576000000
H	-4.097773000000	-5.492005000000	-7.325293000000
H	1.795048000000	-10.860562000000	-0.759884000000
H	-5.187600000000	-7.083739000000	-10.978149000000
H	-12.240733000000	-9.690309000000	-8.890200000000
H	-4.215151000000	-5.792142000000	-11.687812000000
H	-9.531861000000	-12.629054000000	-5.130146000000
H	-2.730574000000	-14.783363000000	-4.276593000000
H	-6.045945000000	-11.686084000000	-8.134184000000

^{4}I4$_{B}$

C	3.283310000000	-3.004225000000	0.336251000000
C	4.144547000000	-1.768242000000	0.109399000000
O	4.151266000000	-0.818352000000	0.894705000000
C	1.790213000000	-2.721623000000	0.084410000000
H	3.611426000000	-3.838999000000	-0.292966000000
H	1.451271000000	-1.932110000000	0.761264000000
H	1.215410000000	-3.626658000000	0.291842000000
N	4.889784000000	-1.766631000000	-1.034230000000
C	5.753625000000	-0.654703000000	-1.390592000000
C	5.075378000000	0.669395000000	-1.783168000000
O	5.761617000000	1.676775000000	-1.904486000000
H	4.748187000000	-2.513877000000	-1.699306000000
H	6.399926000000	-0.409737000000	-0.542910000000
N	3.725968000000	0.636002000000	-1.962428000000
C	2.951228000000	1.847540000000	-2.124337000000
C	2.227027000000	2.339594000000	-0.860279000000
O	1.319997000000	3.171335000000	-0.964649000000
H	3.217234000000	-0.237731000000	-1.855941000000
H	2.195693000000	1.715625000000	-2.902840000000
H	3.637204000000	2.638630000000	-2.441775000000
N	2.677742000000	1.846841000000	0.316181000000
C	2.149545000000	2.281701000000	1.595126000000
H	3.351053000000	1.085915000000	0.319077000000
H	1.431744000000	1.559555000000	2.005605000000
H	2.967836000000	2.402692000000	2.311634000000
H	1.645557000000	3.239257000000	1.452113000000
H	6.399247000000	-0.965549000000	-2.216721000000
H	3.413481000000	-3.296394000000	1.385186000000
S	1.510954000000	-2.198806000000	-1.661706000000
Fe	-0.725587000000	-2.607757000000	-2.394957000000
C	0.336197000000	-2.832422000000	-5.601665000000
C	-0.598688000000	-5.958959000000	-2.063102000000
C	-1.814116000000	-2.231171000000	0.917854000000
C	-0.745612000000	0.908675000000	-2.590115000000
N	-0.287637000000	-4.159146000000	-3.666107000000
C	0.213388000000	-4.047979000000	-4.928496000000
C	0.631350000000	-5.355406000000	-5.401365000000
C	0.390353000000	-6.239936000000	-4.390593000000
C	-0.192015000000	-5.469565000000	-3.305023000000
N	-1.162005000000	-3.817005000000	-0.924300000000
C	-1.073246000000	-5.192643000000	-0.994320000000
C	-1.516851000000	-5.762695000000	0.252501000000
C	-1.853404000000	-4.723880000000	1.070612000000
C	-1.633556000000	-3.499758000000	0.336342000000
N	-1.538798000000	-0.812930000000	-1.051030000000
C	-1.644275000000	-0.981739000000	0.325137000000
C	-1.377134000000	0.286923000000	0.925413000000
C	-1.056884000000	1.181856000000	-0.076556000000
C	-1.130354000000	0.488557000000	-1.322604000000
N	-0.357437000000	-1.264455000000	-3.804848000000
C	-0.447496000000	0.111682000000	-3.710921000000
C	-0.078890000000	0.689675000000	-4.981762000000
C	0.228163000000	-0.337014000000	-5.825787000000
C	0.063431000000	-1.560825000000	-5.082600000000
H	0.709027000000	-2.863364000000	-6.621272000000
H	-0.551419000000	-7.032202000000	-1.893882000000

H	-2.031895000000	-2.236488000000	1.982193000000
H	-0.553365000000	1.971599000000	-2.700389000000
H	-2.227402000000	-4.766804000000	2.085814000000
H	-1.565140000000	-6.826755000000	0.441189000000
H	-1.342169000000	0.467143000000	1.992396000000
H	-0.689149000000	2.194222000000	0.026443000000
H	0.607841000000	-7.302062000000	-4.356918000000
H	1.071891000000	-5.556384000000	-6.369895000000
H	0.546165000000	-0.281763000000	-6.859078000000
H	-0.065886000000	1.752309000000	-5.188263000000
O	-2.716048000000	-2.314085000000	-2.806030000000
C	-3.862419000000	-3.014522000000	-2.730982000000
C	-7.089909000000	-6.493811000000	-0.547856000000
C	-5.836320000000	-3.885774000000	-1.558816000000
C	-4.596231000000	-3.224001000000	-1.555807000000
C	-4.396980000000	-3.493143000000	-3.946772000000
C	-5.620015000000	-4.147048000000	-3.975948000000
C	-6.334283000000	-4.339936000000	-2.788985000000
H	-8.168458000000	-2.527689000000	-0.956165000000
H	-8.023191000000	-2.619635000000	0.815585000000
C	-5.783759000000	-4.138360000000	0.994713000000
C	-7.465479000000	-2.665047000000	-0.127570000000
C	-6.678190000000	-3.991664000000	-0.264805000000
C	-7.613930000000	-5.214955000000	-0.297597000000
C	-8.987906000000	-5.135629000000	-0.029701000000
C	-9.804819000000	-6.267162000000	-0.028755000000
C	-9.263171000000	-7.526135000000	-0.299761000000
C	-7.887847000000	-7.637220000000	-0.553174000000
H	-9.450042000000	-4.175949000000	0.175559000000
H	-10.869543000000	-6.185578000000	0.170401000000
O	-10.114725000000	-8.597085000000	-0.320749000000
H	-7.438428000000	-8.606696000000	-0.755353000000
H	-6.030319000000	-6.601919000000	-0.764222000000
O	-3.676717000000	-3.274045000000	-5.096630000000
H	-2.272562000000	-1.246254000000	-1.668778000000
H	-6.009286000000	-4.504345000000	-4.924172000000
H	-7.286777000000	-4.856842000000	-2.833616000000
H	-2.891307000000	-2.762379000000	-4.811553000000
H	-4.180328000000	-2.835848000000	-0.633298000000
H	-9.686906000000	-9.359209000000	-0.763684000000
H	-6.766752000000	-1.822356000000	-0.142741000000
H	-5.110040000000	-4.997017000000	0.912177000000
H	-5.174970000000	-3.244408000000	1.168738000000
H	-6.415458000000	-4.284420000000	1.877730000000
C	-11.286522000000	-10.183527000000	-8.755871000000
C	-10.374323000000	-9.384178000000	-7.848591000000
O	-9.347088000000	-9.873079000000	-7.358649000000
H	-10.781574000000	-10.330750000000	-9.717292000000
H	-11.454264000000	-11.172074000000	-8.320140000000
N	-10.739824000000	-8.099420000000	-7.586276000000
C	-9.808111000000	-7.116157000000	-7.010562000000
C	-9.449544000000	-7.380728000000	-5.538578000000
O	-8.440420000000	-6.870722000000	-5.052172000000
C	-10.376782000000	-5.701308000000	-7.172163000000
H	-11.535345000000	-7.728182000000	-8.090215000000
H	-8.853118000000	-7.173227000000	-7.543153000000
H	-10.537288000000	-5.471671000000	-8.231070000000

H	-11.322714000000	-5.581341000000	-6.631462000000
H	-9.657353000000	-4.980220000000	-6.779229000000
N	-10.287855000000	-8.172996000000	-4.820142000000
C	-9.973870000000	-8.602760000000	-3.470459000000
C	-9.461485000000	-10.043410000000	-3.355711000000
O	-9.290917000000	-10.549689000000	-2.235291000000
H	-11.120561000000	-8.519677000000	-5.276383000000
H	-10.839604000000	-8.505823000000	-2.809883000000
H	-9.197880000000	-7.938211000000	-3.079954000000
N	-9.239758000000	-10.692348000000	-4.517225000000
C	-8.814382000000	-12.082118000000	-4.595481000000
C	-7.414438000000	-12.270365000000	-5.200407000000
O	-6.510690000000	-12.805409000000	-4.561207000000
H	-9.347839000000	-10.179346000000	-5.388392000000
H	-8.793946000000	-12.488749000000	-3.585522000000
N	-7.244289000000	-11.842623000000	-6.480208000000
C	-5.966232000000	-12.058252000000	-7.147279000000
C	-4.778323000000	-11.392961000000	-6.435692000000
O	-3.742714000000	-12.017413000000	-6.206454000000
H	-7.994917000000	-11.367062000000	-6.972381000000
H	-5.729884000000	-13.124295000000	-7.198599000000
N	-4.957170000000	-10.098779000000	-6.060211000000
C	-3.859821000000	-9.344448000000	-5.464705000000
C	-3.489053000000	-9.900335000000	-4.082259000000
O	-2.317397000000	-9.826131000000	-3.676471000000
C	-4.233900000000	-7.840603000000	-5.454409000000
O	-4.503412000000	-7.490269000000	-6.811551000000
C	-3.110748000000	-6.978118000000	-4.894557000000
H	-5.667314000000	-9.552213000000	-6.533231000000
H	-2.957218000000	-9.457568000000	-6.075265000000
H	-5.143692000000	-7.707210000000	-4.848781000000
H	-5.422643000000	-7.122220000000	-6.903024000000
H	-3.391151000000	-5.924038000000	-4.949152000000
H	-2.194473000000	-7.123859000000	-5.474972000000
H	-2.893169000000	-7.219755000000	-3.851108000000
N	-4.462453000000	-10.492934000000	-3.371237000000
C	-4.222685000000	-11.132703000000	-2.082631000000
C	-3.202597000000	-12.292103000000	-2.197239000000
O	-2.395221000000	-12.516622000000	-1.296174000000
C	-5.578105000000	-11.643246000000	-1.532055000000
O	-6.517565000000	-10.594656000000	-1.782572000000
C	-5.493384000000	-11.988088000000	-0.047863000000
H	-5.425336000000	-10.440956000000	-3.678577000000
H	-3.779342000000	-10.413024000000	-1.386051000000
H	-5.866412000000	-12.527876000000	-2.114856000000
H	-7.429336000000	-10.936596000000	-1.740896000000
H	-6.446495000000	-12.402831000000	0.299188000000
H	-4.704908000000	-12.722449000000	0.145326000000
H	-5.278052000000	-11.086493000000	0.535245000000
N	-3.276787000000	-13.036997000000	-3.332154000000
C	-2.305007000000	-14.073758000000	-3.636493000000
C	-0.975974000000	-13.601940000000	-4.266393000000
O	-0.116168000000	-14.420093000000	-4.561787000000
H	-3.923782000000	-12.768352000000	-4.065697000000
H	-2.045611000000	-14.599378000000	-2.714044000000
N	-0.853051000000	-12.253101000000	-4.407824000000
C	0.339769000000	-11.569074000000	-4.874784000000

C	0.887825000000	-10.640252000000	-3.760069000000
O	1.513521000000	-9.610532000000	-4.013651000000
C	0.054501000000	-10.767260000000	-6.184088000000
O	-0.796932000000	-11.496279000000	-7.057853000000
C	1.330124000000	-10.472701000000	-6.964018000000
H	-1.611740000000	-11.655044000000	-4.104687000000
H	1.079282000000	-12.350946000000	-5.083468000000
H	-0.421264000000	-9.818876000000	-5.890407000000
H	-1.619183000000	-11.723921000000	-6.589005000000
H	1.088825000000	-9.898022000000	-7.864310000000
H	1.798506000000	-11.412737000000	-7.278489000000
H	2.029087000000	-9.905220000000	-6.347231000000
N	0.628612000000	-11.059695000000	-2.491872000000
C	0.999993000000	-10.320305000000	-1.297172000000
C	-0.223494000000	-9.983653000000	-0.428126000000
O	-1.106597000000	-9.076930000000	-1.090827000000
C	0.198618000000	-9.333002000000	0.884274000000
H	0.055028000000	-11.885018000000	-2.372809000000
H	1.500146000000	-9.405810000000	-1.625932000000
H	-0.765353000000	-10.915994000000	-0.211998000000
H	-1.423646000000	-9.482758000000	-1.921861000000
H	-0.682242000000	-9.101872000000	1.491945000000
H	0.845466000000	-10.002698000000	1.461485000000
H	0.744428000000	-8.400457000000	0.697741000000
C	-4.206145000000	-6.514623000000	-10.875211000000
C	-3.003960000000	-7.459039000000	-11.001097000000
C	-2.915512000000	-8.559629000000	-9.924064000000
C	-4.087625000000	-9.550743000000	-10.006980000000
C	-1.576648000000	-9.307807000000	-10.017638000000
H	-4.210725000000	-5.995532000000	-9.909265000000
H	-3.010065000000	-7.933735000000	-11.994594000000
H	-2.082510000000	-6.861181000000	-10.959904000000
H	-2.970706000000	-8.072139000000	-8.940368000000
H	-0.731375000000	-8.611333000000	-9.947590000000
H	-1.466582000000	-10.048146000000	-9.216514000000
H	-1.488151000000	-9.834585000000	-10.977927000000
H	-5.052195000000	-9.058773000000	-9.835227000000
H	-4.124655000000	-10.034698000000	-10.992755000000
H	-3.970956000000	-10.342690000000	-9.256258000000
O	-6.795960000000	-6.147944000000	-7.158624000000
H	-7.233883000000	-6.104961000000	-6.285362000000
H	-6.202154000000	-5.358817000000	-7.255772000000
O	-4.590766000000	-4.625746000000	-7.434718000000
H	-4.238957000000	-4.042746000000	-6.731699000000
H	-4.139771000000	-5.480932000000	-7.299235000000
H	1.715355000000	-10.909298000000	-0.705754000000
H	-5.159110000000	-7.048399000000	-10.970556000000
H	-12.250748000000	-9.701083000000	-8.939259000000
H	-4.182910000000	-5.748389000000	-11.659422000000
H	-9.543551000000	-12.650380000000	-5.188298000000
H	-2.756873000000	-14.795119000000	-4.321601000000
H	-6.045017000000	-11.667219000000	-8.166720000000

^{2}PC

C	3.148115000000	-2.574264000000	0.867609000000
C	3.899084000000	-1.291106000000	0.535430000000
O	3.657603000000	-0.226316000000	1.106312000000
C	1.763128000000	-2.630996000000	0.191585000000
H	3.730754000000	-3.461925000000	0.595628000000
H	1.190686000000	-1.735252000000	0.448233000000
H	1.222993000000	-3.505585000000	0.559643000000
N	4.827526000000	-1.380676000000	-0.460085000000
C	5.611453000000	-0.232218000000	-0.883750000000
C	4.850046000000	0.922475000000	-1.558173000000
O	5.405775000000	2.005144000000	-1.692005000000
H	4.923719000000	-2.255963000000	-0.955827000000
H	6.111132000000	0.215020000000	-0.019688000000
N	3.578381000000	0.658930000000	-1.969685000000
C	2.701006000000	1.706666000000	-2.450542000000
C	1.656298000000	2.213624000000	-1.440950000000
O	0.683033000000	2.856948000000	-1.843487000000
H	3.193062000000	-0.272360000000	-1.848189000000
H	2.160417000000	1.371341000000	-3.338764000000
H	3.330727000000	2.555624000000	-2.732995000000
N	1.903659000000	1.949605000000	-0.137704000000
C	1.034533000000	2.427450000000	0.922148000000
H	2.675798000000	1.342285000000	0.121008000000
H	0.275992000000	1.682137000000	1.194245000000
H	1.634211000000	2.657569000000	1.807312000000
H	0.526580000000	3.330477000000	0.577730000000
H	6.387908000000	-0.579430000000	-1.571316000000
H	3.003215000000	-2.581647000000	1.953372000000
S	1.949902000000	-2.763255000000	-1.636703000000
Fe	-0.056364000000	-3.203755000000	-2.527262000000
C	1.261318000000	-3.625005000000	-5.675584000000
C	0.183803000000	-6.560914000000	-1.993427000000
C	-1.901283000000	-2.883892000000	0.369074000000
C	-0.520515000000	0.115471000000	-3.176340000000
N	0.578961000000	-4.759148000000	-3.612833000000
C	1.161978000000	-4.741701000000	-4.862804000000
C	1.653616000000	-6.051207000000	-5.210980000000
C	1.377546000000	-6.873142000000	-4.161518000000
C	0.705276000000	-6.060849000000	-3.175336000000
N	-0.761907000000	-4.455664000000	-1.135925000000
C	-0.532480000000	-5.813933000000	-1.069760000000
C	-1.155430000000	-6.380312000000	0.100470000000
C	-1.748926000000	-5.349974000000	0.764083000000
C	-1.493811000000	-4.156838000000	-0.002361000000
N	-1.079547000000	-1.717593000000	-1.629011000000
C	-1.676016000000	-1.735711000000	-0.376475000000
C	-2.016412000000	-0.402781000000	0.036435000000
C	-1.602675000000	0.440154000000	-0.954290000000
C	-1.023973000000	-0.377331000000	-1.983295000000
N	0.241069000000	-2.022206000000	-4.124462000000
C	0.034654000000	-0.659912000000	-4.183311000000
C	0.479580000000	-0.136299000000	-5.450337000000
C	0.964543000000	-1.190441000000	-6.162569000000
C	0.826012000000	-2.355110000000	-5.326703000000
H	1.727267000000	-3.748937000000	-6.648303000000
H	0.285223000000	-7.623408000000	-1.793589000000

H	-2.416821000000	-2.776221000000	1.318528000000
H	-0.545935000000	1.191288000000	-3.314527000000
H	-2.316139000000	-5.377202000000	1.686051000000
H	-1.143044000000	-7.437510000000	0.330043000000
H	-2.502357000000	-0.154404000000	0.971629000000
H	-1.650084000000	1.519996000000	-0.998242000000
H	1.587324000000	-7.931841000000	-4.052901000000
H	2.141694000000	-6.295903000000	-6.145854000000
H	1.383255000000	-1.195758000000	-7.160863000000
H	0.411956000000	0.904072000000	-5.741907000000
O	-3.370797000000	-2.553008000000	-3.537831000000
C	-4.660135000000	-2.822241000000	-3.147899000000
C	-7.193703000000	-6.152327000000	-0.579778000000
C	-6.409990000000	-3.394394000000	-1.557361000000
C	-5.066096000000	-3.108078000000	-1.844348000000
C	-5.596254000000	-2.808213000000	-4.190054000000
C	-6.930023000000	-3.092954000000	-3.922911000000
C	-7.327249000000	-3.387411000000	-2.618917000000
H	-8.606733000000	-2.260004000000	-0.319667000000
H	-8.081679000000	-2.528844000000	1.356636000000
C	-5.760292000000	-3.836753000000	0.887760000000
C	-7.735306000000	-2.427174000000	0.321416000000
C	-6.918220000000	-3.663933000000	-0.123990000000
C	-7.759261000000	-4.956190000000	-0.110693000000
C	-9.072810000000	-5.020933000000	0.374507000000
C	-9.798692000000	-6.213426000000	0.371264000000
C	-9.219273000000	-7.389352000000	-0.113958000000
C	-7.895337000000	-7.355769000000	-0.579175000000
H	-9.558283000000	-4.129564000000	0.758292000000
H	-10.820475000000	-6.244849000000	0.738268000000
O	-9.981774000000	-8.522755000000	-0.125362000000
H	-7.407267000000	-8.263738000000	-0.924747000000
H	-6.178600000000	-6.146677000000	-0.968597000000
O	-5.183413000000	-2.511151000000	-5.470920000000
H	-2.781416000000	-2.344510000000	-2.785832000000
H	-7.646725000000	-3.074849000000	-4.739206000000
H	-8.368895000000	-3.624654000000	-2.424715000000
H	-4.237798000000	-2.276563000000	-5.410769000000
H	-4.313691000000	-3.112825000000	-1.062896000000
H	-9.530841000000	-9.223726000000	-0.641539000000
H	-7.106097000000	-1.532803000000	0.264468000000
H	-5.085343000000	-4.650866000000	0.605250000000
H	-5.169799000000	-2.917518000000	0.979263000000
H	-6.171571000000	-4.070548000000	1.875315000000
C	-11.777229000000	-10.018145000000	-8.573483000000
C	-10.744024000000	-9.340853000000	-7.698136000000
O	-9.796179000000	-9.967035000000	-7.208138000000
H	-11.311449000000	-10.270742000000	-9.532653000000
H	-12.086066000000	-10.956065000000	-8.104506000000
N	-10.913115000000	-8.010047000000	-7.462740000000
C	-9.840795000000	-7.180403000000	-6.897675000000
C	-9.481296000000	-7.550899000000	-5.451483000000
O	-8.355587000000	-7.310892000000	-5.013659000000
C	-10.223941000000	-5.697725000000	-6.985007000000
H	-11.643723000000	-7.529561000000	-7.972533000000
H	-8.916390000000	-7.346877000000	-7.462290000000
H	-10.371887000000	-5.400033000000	-8.028763000000

H	-11.137950000000	-5.483747000000	-6.419343000000
H	-9.414709000000	-5.089116000000	-6.574432000000
N	-10.442484000000	-8.120420000000	-4.678926000000
C	-10.142643000000	-8.559871000000	-3.327055000000
C	-9.449464000000	-9.926022000000	-3.226680000000
O	-9.085927000000	-10.345198000000	-2.115232000000
H	-11.350434000000	-8.299814000000	-5.084655000000
H	-11.056230000000	-8.590369000000	-2.728245000000
H	-9.472038000000	-7.832947000000	-2.861021000000
N	-9.287666000000	-10.606683000000	-4.376315000000
C	-8.604432000000	-11.886473000000	-4.464172000000
C	-7.225277000000	-11.840804000000	-5.144942000000
O	-6.272643000000	-12.446175000000	-4.654795000000
H	-9.606812000000	-10.182720000000	-5.242958000000
H	-8.459581000000	-12.271270000000	-3.455931000000
N	-7.132999000000	-11.155535000000	-6.313844000000
C	-5.921447000000	-11.254851000000	-7.122357000000
C	-4.657544000000	-10.727579000000	-6.428571000000
O	-3.627028000000	-11.398535000000	-6.406039000000
H	-7.959893000000	-10.736982000000	-6.727254000000
H	-5.708592000000	-12.295597000000	-7.382167000000
N	-4.749312000000	-9.498746000000	-5.857362000000
C	-3.577984000000	-8.900702000000	-5.216348000000
C	-3.225601000000	-9.643544000000	-3.914968000000
O	-2.041320000000	-9.758708000000	-3.563348000000
C	-3.791625000000	-7.373918000000	-5.056053000000
O	-3.912592000000	-6.809483000000	-6.358873000000
C	-2.639105000000	-6.678648000000	-4.350242000000
H	-5.562482000000	-8.925013000000	-6.055841000000
H	-2.708184000000	-9.038803000000	-5.865302000000
H	-4.724093000000	-7.209438000000	-4.489476000000
H	-4.787592000000	-7.076801000000	-6.722593000000
H	-2.832174000000	-5.602074000000	-4.318665000000
H	-1.701708000000	-6.847887000000	-4.888165000000
H	-2.515924000000	-7.039256000000	-3.326394000000
N	-4.242831000000	-10.193941000000	-3.228793000000
C	-4.045220000000	-11.014032000000	-2.040960000000
C	-3.165836000000	-12.251775000000	-2.347015000000
O	-2.316057000000	-12.644760000000	-1.546730000000
C	-5.436347000000	-11.451887000000	-1.507874000000
O	-6.312178000000	-10.337944000000	-1.699292000000
C	-5.361320000000	-11.875638000000	-0.043748000000
H	-5.204106000000	-9.984780000000	-3.468082000000
H	-3.504838000000	-10.444819000000	-1.277012000000
H	-5.785203000000	-12.288209000000	-2.127620000000
H	-7.241052000000	-10.634859000000	-1.691912000000
H	-6.330277000000	-12.260111000000	0.293844000000
H	-4.608407000000	-12.657926000000	0.100898000000
H	-5.095097000000	-11.016501000000	0.580891000000
N	-3.402972000000	-12.861746000000	-3.535976000000
C	-2.600635000000	-13.979254000000	-4.003822000000
C	-1.214584000000	-13.631101000000	-4.587215000000
O	-0.469296000000	-14.530010000000	-4.952475000000
H	-4.135035000000	-12.511417000000	-4.144898000000
H	-2.420168000000	-14.673454000000	-3.178360000000
N	-0.907888000000	-12.305277000000	-4.610743000000
C	0.385255000000	-11.767196000000	-4.993926000000

C	1.079989000000	-11.110228000000	-3.773639000000
O	1.924062000000	-10.221675000000	-3.904159000000
C	0.245296000000	-10.761998000000	-6.182398000000
O	-0.686253000000	-11.236510000000	-7.143361000000
C	1.561641000000	-10.560702000000	-6.924347000000
H	-1.586643000000	-11.636624000000	-4.268022000000
H	0.986837000000	-12.624239000000	-5.318117000000
H	-0.090690000000	-9.800306000000	-5.763615000000
H	-1.553136000000	-11.366002000000	-6.719838000000
H	1.418966000000	-9.839016000000	-7.735575000000
H	1.882947000000	-11.509648000000	-7.369805000000
H	2.333735000000	-10.199281000000	-6.243680000000
N	0.700202000000	-11.595584000000	-2.561289000000
C	1.212917000000	-11.093055000000	-1.297251000000
C	0.082047000000	-10.632455000000	-0.364052000000
O	-0.654370000000	-9.547733000000	-0.931388000000
C	0.633840000000	-10.154739000000	0.974180000000
H	-0.036243000000	-12.289945000000	-2.527149000000
H	1.882393000000	-10.261297000000	-1.530552000000
H	-0.599509000000	-11.478228000000	-0.197278000000
H	-1.039534000000	-9.824758000000	-1.785865000000
H	-0.183724000000	-9.838227000000	1.629947000000
H	1.185994000000	-10.956163000000	1.477282000000
H	1.309792000000	-9.303261000000	0.832288000000
C	-4.328950000000	-6.222909000000	-10.708461000000
C	-3.003256000000	-6.989064000000	-10.801936000000
C	-2.783098000000	-8.070614000000	-9.725124000000
C	-3.840782000000	-9.185069000000	-9.792364000000
C	-1.372701000000	-8.669866000000	-9.840422000000
H	-4.441297000000	-5.719219000000	-9.740779000000
H	-2.921610000000	-7.459086000000	-11.794266000000
H	-2.174880000000	-6.269043000000	-10.741360000000
H	-2.869389000000	-7.589232000000	-8.739619000000
H	-0.605248000000	-7.889647000000	-9.758805000000
H	-1.184363000000	-9.415699000000	-9.059351000000
H	-1.237500000000	-9.164225000000	-10.812388000000
H	-4.848562000000	-8.803619000000	-9.585162000000
H	-3.855656000000	-9.652816000000	-10.786536000000
H	-3.613896000000	-9.972843000000	-9.063748000000
O	-6.560928000000	-6.997375000000	-7.110091000000
H	-7.076765000000	-6.949552000000	-6.276706000000
H	-6.318971000000	-6.054810000000	-7.317835000000
O	-5.295043000000	-4.609034000000	-7.403310000000
H	-5.419309000000	-3.831002000000	-6.823195000000
H	-4.527761000000	-5.075208000000	-7.014369000000
H	1.800721000000	-11.875591000000	-0.796368000000
H	-5.192806000000	-6.885261000000	-10.841326000000
H	-12.658874000000	-9.399881000000	-8.764666000000
H	-4.387711000000	-5.452817000000	-11.487333000000
H	-9.236854000000	-12.594487000000	-5.015064000000
H	-3.161859000000	-14.511109000000	-4.775661000000
H	-6.086622000000	-10.687375000000	-8.043135000000

^{4}PC

C	3.320179000000	-2.371120000000	1.021569000000
C	3.977751000000	-1.035650000000	0.698393000000
O	3.626223000000	0.014474000000	1.239432000000
C	1.961278000000	-2.530072000000	0.309137000000
H	3.973811000000	-3.212396000000	0.764523000000
H	1.314111000000	-1.687354000000	0.569698000000
H	1.485237000000	-3.451653000000	0.652892000000
N	4.952266000000	-1.065049000000	-0.255618000000
C	5.639974000000	0.139842000000	-0.687332000000
C	4.808630000000	1.191326000000	-1.443730000000
O	5.278862000000	2.309138000000	-1.615171000000
H	5.119908000000	-1.932464000000	-0.746262000000
H	6.045073000000	0.663447000000	0.183203000000
N	3.575362000000	0.806239000000	-1.874636000000
C	2.630960000000	1.758975000000	-2.420552000000
C	1.546082000000	2.251475000000	-1.446824000000
O	0.547714000000	2.827448000000	-1.889632000000
H	3.258494000000	-0.148104000000	-1.726310000000
H	2.122191000000	1.332957000000	-3.288886000000
H	3.198829000000	2.633454000000	-2.751840000000
N	1.785291000000	2.052269000000	-0.130977000000
C	0.879211000000	2.527684000000	0.898230000000
H	2.580672000000	1.491958000000	0.161759000000
H	0.162814000000	1.752688000000	1.199672000000
H	1.455104000000	2.834014000000	1.776173000000
H	0.323784000000	3.382534000000	0.507309000000
H	6.483995000000	-0.150319000000	-1.319405000000
H	3.150699000000	-2.388125000000	2.103940000000
S	2.183387000000	-2.607727000000	-1.519247000000
Fe	0.124436000000	-3.190986000000	-2.468460000000
C	1.353594000000	-3.645250000000	-5.644985000000
C	0.165585000000	-6.622511000000	-2.002022000000
C	-1.804722000000	-2.885679000000	0.388148000000
C	-0.610087000000	0.101038000000	-3.269090000000
N	0.643121000000	-4.839262000000	-3.619294000000
C	1.242067000000	-4.793688000000	-4.854614000000
C	1.709402000000	-6.114771000000	-5.218303000000
C	1.384207000000	-6.954001000000	-4.192681000000
C	0.714559000000	-6.142531000000	-3.195892000000
N	-0.706557000000	-4.522757000000	-1.070469000000
C	-0.518444000000	-5.878395000000	-1.030986000000
C	-1.136010000000	-6.429116000000	0.158588000000
C	-1.678525000000	-5.379003000000	0.840258000000
C	-1.408817000000	-4.187821000000	0.060883000000
N	-1.121210000000	-1.695029000000	-1.658494000000
C	-1.644683000000	-1.723749000000	-0.380365000000
C	-1.994429000000	-0.386615000000	0.028607000000
C	-1.654358000000	0.451320000000	-0.998568000000
C	-1.102741000000	-0.369941000000	-2.045509000000
N	0.270795000000	-1.993934000000	-4.188669000000
C	0.006416000000	-0.650995000000	-4.274859000000
C	0.480115000000	-0.140603000000	-5.544757000000
C	1.031885000000	-1.195240000000	-6.211771000000
C	0.901394000000	-2.351577000000	-5.349685000000
H	1.843835000000	-3.773250000000	-6.606659000000
H	0.234656000000	-7.691760000000	-1.817540000000

H	-2.293197000000	-2.758380000000	1.350907000000
H	-0.670885000000	1.174583000000	-3.420938000000
H	-2.227165000000	-5.399830000000	1.773732000000
H	-1.156836000000	-7.484738000000	0.394723000000
H	-2.431712000000	-0.124173000000	0.984001000000
H	-1.723511000000	1.529811000000	-1.044163000000
H	1.570304000000	-8.019511000000	-4.108975000000
H	2.211874000000	-6.362389000000	-6.144932000000
H	1.484324000000	-1.199696000000	-7.195500000000
H	0.390676000000	0.886918000000	-5.874106000000
O	-3.280909000000	-2.681224000000	-3.568636000000
C	-4.573223000000	-2.904298000000	-3.163229000000
C	-7.151325000000	-6.176949000000	-0.582721000000
C	-6.322898000000	-3.429999000000	-1.555595000000
C	-4.973421000000	-3.188282000000	-1.857121000000
C	-5.522066000000	-2.848758000000	-4.192748000000
C	-6.861413000000	-3.090413000000	-3.911934000000
C	-7.252394000000	-3.382804000000	-2.605527000000
H	-8.482409000000	-2.254711000000	-0.289158000000
H	-7.949308000000	-2.549063000000	1.380064000000
C	-5.659573000000	-3.899238000000	0.881008000000
C	-7.609406000000	-2.446620000000	0.342761000000
C	-6.823005000000	-3.697083000000	-0.118877000000
C	-7.690516000000	-4.971680000000	-0.106203000000
C	-9.003281000000	-5.011908000000	0.383690000000
C	-9.753324000000	-6.189510000000	0.378092000000
C	-9.199559000000	-7.374802000000	-0.114157000000
C	-7.877055000000	-7.365861000000	-0.584339000000
H	-9.469267000000	-4.112593000000	0.773106000000
H	-10.774150000000	-6.201716000000	0.748895000000
O	-9.984369000000	-8.493033000000	-0.127520000000
H	-7.408231000000	-8.281811000000	-0.935131000000
H	-6.137885000000	-6.190287000000	-0.975637000000
O	-5.113034000000	-2.553143000000	-5.475117000000
H	-2.680643000000	-2.459693000000	-2.826606000000
H	-7.587169000000	-3.040238000000	-4.718858000000
H	-8.299172000000	-3.586300000000	-2.400639000000
H	-4.159970000000	-2.348146000000	-5.419218000000
H	-4.212429000000	-3.227445000000	-1.085338000000
H	-9.546530000000	-9.201903000000	-0.644045000000
H	-6.961291000000	-1.565738000000	0.287529000000
H	-5.005278000000	-4.727635000000	0.591577000000
H	-5.047868000000	-2.993491000000	0.968098000000
H	-6.066542000000	-4.125712000000	1.872075000000
C	-11.820287000000	-9.974060000000	-8.570114000000
C	-10.777773000000	-9.306877000000	-7.698041000000
O	-9.834668000000	-9.942212000000	-7.210707000000
H	-11.359696000000	-10.232291000000	-9.530285000000
H	-12.137679000000	-10.908321000000	-8.099536000000
N	-10.932923000000	-7.974437000000	-7.462409000000
C	-9.850563000000	-7.155690000000	-6.900565000000
C	-9.488466000000	-7.532596000000	-5.456723000000
O	-8.357543000000	-7.307936000000	-5.024203000000
C	-10.220630000000	-5.669391000000	-6.983120000000
H	-11.660125000000	-7.486683000000	-7.970169000000
H	-8.930193000000	-7.329471000000	-7.469549000000
H	-10.370957000000	-5.368283000000	-8.025566000000

H	-11.129889000000	-5.448273000000	-6.412587000000
H	-9.403951000000	-5.068893000000	-6.575337000000
N	-10.453487000000	-8.089755000000	-4.679900000000
C	-10.153954000000	-8.532747000000	-3.329104000000
C	-9.477421000000	-9.907338000000	-3.230719000000
O	-9.118160000000	-10.331635000000	-2.119841000000
H	-11.365460000000	-8.257405000000	-5.081620000000
H	-11.065658000000	-8.551857000000	-2.726982000000
H	-9.472710000000	-7.814179000000	-2.865492000000
N	-9.324230000000	-10.588674000000	-4.381184000000
C	-8.655093000000	-11.875740000000	-4.471145000000
C	-7.276804000000	-11.844049000000	-5.154654000000
O	-6.328943000000	-12.458419000000	-4.666362000000
H	-9.639542000000	-10.160198000000	-5.247023000000
H	-8.512654000000	-12.262911000000	-3.463479000000
N	-7.180716000000	-11.160064000000	-6.323951000000
C	-5.971845000000	-11.269723000000	-7.135086000000
C	-4.702078000000	-10.752580000000	-6.444501000000
O	-3.677086000000	-11.431958000000	-6.424148000000
H	-8.004637000000	-10.733839000000	-6.735435000000
H	-5.768166000000	-12.312300000000	-7.394893000000
N	-4.782169000000	-9.522547000000	-5.873947000000
C	-3.604719000000	-8.934461000000	-5.235165000000
C	-3.256178000000	-9.680596000000	-3.934760000000
O	-2.071952000000	-9.806374000000	-3.586279000000
C	-3.804706000000	-7.405768000000	-5.075090000000
O	-3.919222000000	-6.840766000000	-6.378194000000
C	-2.647141000000	-6.720648000000	-4.367299000000
H	-5.590646000000	-8.941779000000	-6.071050000000
H	-2.737184000000	-9.080024000000	-5.885514000000
H	-4.736233000000	-7.232688000000	-4.509562000000
H	-4.796881000000	-7.099476000000	-6.741926000000
H	-2.828775000000	-5.642073000000	-4.336947000000
H	-1.709822000000	-6.900803000000	-4.901922000000
H	-2.531683000000	-7.081380000000	-3.342434000000
N	-4.276445000000	-10.222102000000	-3.246202000000
C	-4.083441000000	-11.044268000000	-2.058945000000
C	-3.214532000000	-12.288789000000	-2.366949000000
O	-2.363201000000	-12.686213000000	-1.570568000000
C	-5.476922000000	-11.471847000000	-1.523776000000
O	-6.344389000000	-10.351284000000	-1.713419000000
C	-5.402585000000	-11.896608000000	-0.059900000000
H	-5.236408000000	-10.004463000000	-3.483357000000
H	-3.537681000000	-10.479538000000	-1.295493000000
H	-5.833262000000	-12.305236000000	-2.143153000000
H	-7.275771000000	-10.640279000000	-1.702472000000
H	-6.373985000000	-12.273328000000	0.279376000000
H	-4.655835000000	-12.685082000000	0.083175000000
H	-5.128252000000	-11.039871000000	0.564523000000
N	-3.462839000000	-12.899408000000	-3.553340000000
C	-2.672658000000	-14.025288000000	-4.021922000000
C	-1.285055000000	-13.691553000000	-4.609777000000
O	-0.548822000000	-14.598385000000	-4.973807000000
H	-4.195341000000	-12.544346000000	-4.159081000000
H	-2.496444000000	-14.720002000000	-3.195962000000
N	-0.966689000000	-12.368623000000	-4.639226000000
C	0.330195000000	-11.844128000000	-5.028235000000

C	1.032611000000	-11.185492000000	-3.813412000000
O	1.881656000000	-10.302766000000	-3.951171000000
C	0.197172000000	-10.845629000000	-6.223058000000
O	-0.739868000000	-11.318336000000	-7.179384000000
C	1.514273000000	-10.660881000000	-6.967970000000
H	-1.638426000000	-11.692543000000	-4.297308000000
H	0.923811000000	-12.708503000000	-5.347689000000
H	-0.129709000000	-9.878205000000	-5.810171000000
H	-1.608388000000	-11.432218000000	-6.754726000000
H	1.377229000000	-9.942775000000	-7.783309000000
H	1.826131000000	-11.615496000000	-7.407983000000
H	2.290632000000	-10.302480000000	-6.290548000000
N	0.654843000000	-11.663103000000	-2.597340000000
C	1.176227000000	-11.157529000000	-1.338068000000
C	0.053118000000	-10.685661000000	-0.401097000000
O	-0.680613000000	-9.599381000000	-0.969390000000
C	0.615316000000	-10.205486000000	0.931984000000
H	-0.085809000000	-12.352672000000	-2.556887000000
H	1.849654000000	-10.331027000000	-1.578654000000
H	-0.632632000000	-11.526513000000	-0.226688000000
H	-1.074671000000	-9.880113000000	-1.818734000000
H	-0.196689000000	-9.883233000000	1.591875000000
H	1.167107000000	-11.007484000000	1.434513000000
H	1.294159000000	-9.357526000000	0.783074000000
C	-4.330392000000	-6.245354000000	-10.729138000000
C	-3.014626000000	-7.028779000000	-10.820786000000
C	-2.811106000000	-8.114377000000	-9.744873000000
C	-3.884482000000	-9.213565000000	-9.814897000000
C	-1.409094000000	-8.733193000000	-9.859470000000
H	-4.438639000000	-5.742673000000	-9.760465000000
H	-2.937047000000	-7.498747000000	-11.813476000000
H	-2.177052000000	-6.319661000000	-10.757772000000
H	-2.891951000000	-7.632893000000	-8.758913000000
H	-0.630875000000	-7.964123000000	-9.774071000000
H	-1.232314000000	-9.484338000000	-9.080795000000
H	-1.279176000000	-9.226180000000	-10.832861000000
H	-4.886826000000	-8.818172000000	-9.607595000000
H	-3.905334000000	-9.679288000000	-10.809914000000
H	-3.669309000000	-10.005850000000	-9.087611000000
O	-6.569739000000	-7.003836000000	-7.127111000000
H	-7.082899000000	-6.953019000000	-6.292228000000
H	-6.320118000000	-6.063060000000	-7.334269000000
O	-5.283614000000	-4.627389000000	-7.422025000000
H	-5.394909000000	-3.850080000000	-6.838164000000
H	-4.519516000000	-5.102724000000	-7.038101000000
H	1.761826000000	-11.941032000000	-0.836155000000
H	-5.202530000000	-6.895907000000	-10.865897000000
H	-12.695913000000	-9.346754000000	-8.759449000000
H	-4.376792000000	-5.472716000000	-11.506279000000
H	-9.296059000000	-12.576514000000	-5.021452000000
H	-3.241355000000	-14.552871000000	-4.791221000000
H	-6.134388000000	-10.701321000000	-8.055753000000

^{2}TS6$_{B}$

C	2.223391000000	-6.323758000000	-1.549603000000
C	3.586112000000	-7.002587000000	-1.560127000000
O	4.016264000000	-7.615911000000	-0.581659000000
C	2.226252000000	-5.056800000000	-0.673703000000
H	1.883836000000	-6.079227000000	-2.562216000000
H	2.578653000000	-5.316814000000	0.328569000000
H	1.204242000000	-4.679171000000	-0.595188000000
N	4.319482000000	-6.860413000000	-2.701203000000
C	5.624706000000	-7.479709000000	-2.858540000000
C	6.763846000000	-6.948027000000	-1.973091000000
O	7.810698000000	-7.579358000000	-1.908931000000
H	3.951876000000	-6.284413000000	-3.445030000000
H	5.557280000000	-8.548236000000	-2.633873000000
N	6.531045000000	-5.785587000000	-1.300268000000
C	7.453333000000	-5.295280000000	-0.297115000000
C	7.021241000000	-5.520335000000	1.161044000000
O	7.550886000000	-4.861689000000	2.062568000000
H	5.639381000000	-5.310082000000	-1.402693000000
H	7.624123000000	-4.223031000000	-0.422141000000
H	8.405212000000	-5.812890000000	-0.449874000000
N	6.083488000000	-6.472928000000	1.366197000000
C	5.621608000000	-6.835603000000	2.693067000000
H	5.630812000000	-6.916313000000	0.571383000000
H	5.572726000000	-7.924939000000	2.787462000000
H	6.329770000000	-6.437961000000	3.422102000000
H	4.624795000000	-6.424097000000	2.899054000000
H	5.931089000000	-7.381938000000	-3.903756000000
H	1.515994000000	-7.041780000000	-1.118565000000
S	3.307087000000	-3.759373000000	-1.406207000000
Fe	2.879671000000	-1.636026000000	-0.246103000000
C	5.454445000000	-0.210061000000	-1.973511000000
C	0.772608000000	-1.032603000000	-2.862603000000
C	0.267880000000	-2.855622000000	1.583869000000
C	5.088993000000	-2.834178000000	2.068956000000
N	3.075141000000	-0.789704000000	-2.054277000000
C	4.202034000000	-0.202626000000	-2.572993000000
C	3.916918000000	0.368033000000	-3.864571000000
C	2.609685000000	0.098144000000	-4.133113000000
C	2.089719000000	-0.613595000000	-2.993546000000
N	0.922560000000	-1.926729000000	-0.586450000000
C	0.229475000000	-1.627708000000	-1.730568000000
C	-1.168498000000	-1.941529000000	-1.565962000000
C	-1.327085000000	-2.387847000000	-0.288776000000
C	-0.012611000000	-2.384880000000	0.306901000000
N	2.711569000000	-2.656105000000	1.476317000000
C	1.546577000000	-3.013189000000	2.109753000000
C	1.840890000000	-3.642763000000	3.370422000000
C	3.200762000000	-3.674730000000	3.484916000000
C	3.731041000000	-3.044082000000	2.304767000000
N	4.881257000000	-1.557464000000	-0.015435000000
C	5.612285000000	-2.124309000000	1.000600000000
C	7.014837000000	-1.826310000000	0.832869000000
C	7.113345000000	-1.042450000000	-0.276804000000
C	5.776375000000	-0.893258000000	-0.799885000000
H	6.260044000000	0.298700000000	-2.492415000000
H	0.101202000000	-0.834070000000	-3.691797000000

H	-0.574166000000	-3.158265000000	2.199332000000
H	5.799087000000	-3.256468000000	2.771903000000
H	-2.236069000000	-2.678153000000	0.225396000000
H	-1.920549000000	-1.814244000000	-2.333185000000
H	1.095495000000	-4.030362000000	4.053141000000
H	3.802852000000	-4.078026000000	4.288600000000
H	2.031893000000	0.358140000000	-5.010340000000
H	4.638560000000	0.890944000000	-4.478333000000
H	8.000742000000	-0.615760000000	-0.726104000000
H	7.793341000000	-2.210600000000	1.477884000000
O	2.664423000000	-0.138599000000	0.537797000000
C	5.036398000000	2.218573000000	0.365559000000
C	6.959148000000	6.716329000000	0.323838000000
C	6.571068000000	3.875347000000	-0.567611000000
C	6.272017000000	2.862819000000	0.356097000000
C	4.024121000000	2.573982000000	-0.561742000000
C	4.332940000000	3.582740000000	-1.512297000000
C	5.564143000000	4.214397000000	-1.500384000000
H	8.109800000000	4.308894000000	-2.827727000000
H	9.671209000000	4.470390000000	-1.996586000000
C	8.842715000000	4.239626000000	0.566806000000
C	8.659034000000	4.058184000000	-1.914529000000
C	7.939400000000	4.576058000000	-0.644311000000
C	7.737015000000	6.104541000000	-0.672302000000
C	8.328886000000	6.943899000000	-1.627063000000
C	8.159949000000	8.327972000000	-1.594332000000
C	7.385167000000	8.912869000000	-0.590541000000
C	6.781875000000	8.096425000000	0.373054000000
H	8.937861000000	6.525287000000	-2.421277000000
H	8.622397000000	8.965920000000	-2.340997000000
O	7.249981000000	10.273018000000	-0.601726000000
H	6.170819000000	8.539856000000	1.157864000000
H	6.470360000000	6.099637000000	1.073640000000
O	2.819180000000	2.024465000000	-0.550077000000
H	4.827116000000	1.437442000000	1.090236000000
H	3.574894000000	3.851548000000	-2.242677000000
H	5.760213000000	5.001484000000	-2.223891000000
H	2.745393000000	0.871737000000	-0.007245000000
H	7.011789000000	2.559411000000	1.089497000000
H	6.690103000000	10.541848000000	0.143213000000
H	8.747838000000	2.967611000000	-1.865824000000
H	8.374684000000	4.520460000000	1.515840000000
H	9.085026000000	3.171214000000	0.604967000000
H	9.784157000000	4.792219000000	0.484605000000
C	-0.724945000000	4.635141000000	6.416456000000
C	-0.719637000000	3.640794000000	5.272629000000
O	-1.758727000000	3.093766000000	4.887563000000
H	0.275078000000	4.888171000000	6.780337000000
H	-1.219199000000	5.553249000000	6.080000000000
N	0.488259000000	3.368423000000	4.705247000000
C	0.618267000000	2.643470000000	3.433679000000
C	0.038563000000	1.227185000000	3.498390000000
O	-0.440143000000	0.689011000000	2.500057000000
C	2.093547000000	2.593734000000	3.017946000000
H	1.280690000000	3.932005000000	4.985941000000
H	0.041295000000	3.156991000000	2.658713000000
H	2.460680000000	3.601876000000	2.801110000000

H	2.708794000000	2.142159000000	3.805179000000
H	2.204310000000	1.996318000000	2.111408000000
N	0.100722000000	0.570820000000	4.685295000000
C	-0.436012000000	-0.774330000000	4.790594000000
C	-1.953333000000	-0.874466000000	4.561946000000
O	-2.436053000000	-1.942130000000	4.174279000000
H	0.511880000000	1.030903000000	5.484548000000
H	-0.214687000000	-1.158510000000	5.791372000000
H	0.039516000000	-1.430560000000	4.060139000000
N	-2.675182000000	0.220781000000	4.895841000000
C	-4.124830000000	0.244948000000	4.847469000000
C	-4.798787000000	1.074620000000	3.743242000000
O	-5.942732000000	0.771819000000	3.405411000000
H	-2.182043000000	1.075673000000	5.134687000000
H	-4.473555000000	-0.781209000000	4.734651000000
N	-4.138754000000	2.147236000000	3.245857000000
C	-4.830988000000	3.111444000000	2.390975000000
C	-5.296319000000	2.529505000000	1.045017000000
O	-6.487922000000	2.508206000000	0.741793000000
H	-3.221443000000	2.392091000000	3.600903000000
H	-5.730986000000	3.485142000000	2.886632000000
N	-4.319115000000	2.021049000000	0.250982000000
C	-4.697489000000	1.405099000000	-1.016049000000
C	-5.626296000000	0.210828000000	-0.758289000000
O	-6.586564000000	-0.021683000000	-1.504293000000
C	-3.448014000000	0.946159000000	-1.819455000000
O	-2.534507000000	2.015106000000	-1.997141000000
C	-3.836854000000	0.429899000000	-3.202412000000
H	-3.340011000000	2.244504000000	0.436925000000
H	-5.269982000000	2.112398000000	-1.624777000000
H	-2.963725000000	0.139002000000	-1.248960000000
H	-2.012485000000	2.108029000000	-1.169568000000
H	-2.934096000000	0.106156000000	-3.731096000000
H	-4.296275000000	1.234624000000	-3.786104000000
H	-4.542864000000	-0.403915000000	-3.157431000000
N	-5.307373000000	-0.574358000000	0.291519000000
C	-5.993957000000	-1.822812000000	0.598859000000
C	-7.511643000000	-1.626149000000	0.816275000000
O	-8.319115000000	-2.456671000000	0.395930000000
C	-5.349305000000	-2.435029000000	1.870943000000
O	-3.933736000000	-2.237955000000	1.766457000000
C	-5.700724000000	-3.912485000000	2.022048000000
H	-4.454070000000	-0.404162000000	0.810638000000
H	-5.906484000000	-2.513152000000	-0.248184000000
H	-5.723216000000	-1.868545000000	2.734932000000
H	-3.536241000000	-2.247475000000	2.660492000000
H	-5.302809000000	-4.304186000000	2.964911000000
H	-6.784817000000	-4.064110000000	2.009269000000
H	-5.263495000000	-4.485798000000	1.197594000000
N	-7.875755000000	-0.518494000000	1.506861000000
C	-9.273191000000	-0.191659000000	1.737880000000
C	-10.040614000000	0.401345000000	0.537605000000
O	-11.237595000000	0.636780000000	0.643855000000
H	-7.167621000000	0.122189000000	1.853869000000
H	-9.818085000000	-1.091965000000	2.035217000000
N	-9.300919000000	0.584847000000	-0.586163000000
C	-9.830984000000	1.063024000000	-1.848205000000

C	-9.700740000000	-0.012114000000	-2.956983000000
O	-9.808687000000	0.278873000000	-4.146634000000
C	-9.125357000000	2.397155000000	-2.276164000000
O	-8.899494000000	3.238632000000	-1.155959000000
C	-9.970568000000	3.198963000000	-3.258414000000
H	-8.309928000000	0.372910000000	-0.567068000000
H	-10.894634000000	1.262113000000	-1.677864000000
H	-8.168833000000	2.127017000000	-2.750218000000
H	-8.229470000000	2.830858000000	-0.581340000000
H	-9.433946000000	4.108440000000	-3.549542000000
H	-10.908848000000	3.499260000000	-2.776418000000
H	-10.192525000000	2.600662000000	-4.143218000000
N	-9.447475000000	-1.280690000000	-2.526439000000
C	-9.194482000000	-2.389494000000	-3.434193000000
C	-8.007297000000	-3.243271000000	-2.958299000000
O	-6.796915000000	-2.493991000000	-2.915436000000
C	-7.770974000000	-4.426052000000	-3.891769000000
H	-9.404375000000	-1.461001000000	-1.530236000000
H	-8.989487000000	-1.958917000000	-4.418168000000
H	-8.241327000000	-3.620607000000	-1.949657000000
H	-6.966122000000	-1.659855000000	-2.437947000000
H	-6.912086000000	-5.010338000000	-3.545535000000
H	-8.646858000000	-5.083856000000	-3.926048000000
H	-7.555549000000	-4.073791000000	-4.907257000000
C	1.474480000000	6.494395000000	-2.865852000000
C	1.029126000000	5.454242000000	-3.901401000000
C	-0.056935000000	4.474664000000	-3.414765000000
C	-1.396488000000	5.168159000000	-3.118340000000
C	-0.260554000000	3.331662000000	-4.420326000000
H	1.871234000000	6.010666000000	-1.961243000000
H	0.664791000000	5.969527000000	-4.802152000000
H	1.907874000000	4.874744000000	-4.220405000000
H	0.301059000000	4.017842000000	-2.477268000000
H	0.680071000000	2.796867000000	-4.606477000000
H	-0.997669000000	2.617485000000	-4.039410000000
H	-0.622419000000	3.718085000000	-5.382496000000
H	-1.300760000000	5.949104000000	-2.354085000000
H	-1.793567000000	5.642989000000	-4.025475000000
H	-2.134212000000	4.438385000000	-2.768565000000
O	-1.425355000000	2.394392000000	0.519892000000
H	-1.063560000000	1.668166000000	1.072239000000
H	-0.648541000000	2.985394000000	0.346685000000
O	0.878080000000	3.818082000000	0.177757000000
H	1.544364000000	3.162888000000	-0.155073000000
H	0.834163000000	4.505758000000	-0.504070000000
H	-10.087117000000	-3.026963000000	-3.523583000000
H	0.651486000000	7.150018000000	-2.560778000000
H	-1.319052000000	4.230145000000	7.240015000000
H	2.272449000000	7.132962000000	-3.261907000000
H	-4.513750000000	0.619815000000	5.803215000000
H	-9.328563000000	0.526082000000	2.560002000000
H	-4.141664000000	3.943852000000	2.220249000000

^{4}TS6$_{B}$

C	2.221696000000	-6.308767000000	-1.547413000000
C	3.584456000000	-6.987139000000	-1.568351000000
O	4.017524000000	-7.609454000000	-0.596812000000
C	2.230446000000	-5.041646000000	-0.672143000000
H	1.874278000000	-6.064312000000	-2.557332000000
H	2.593837000000	-5.297991000000	0.327295000000
H	1.207958000000	-4.667509000000	-0.581301000000
N	4.315254000000	-6.833507000000	-2.709592000000
C	5.619735000000	-7.452177000000	-2.876067000000
C	6.759671000000	-6.933097000000	-1.984148000000
O	7.803276000000	-7.569829000000	-1.922027000000
H	3.945481000000	-6.250958000000	-3.447278000000
H	5.551794000000	-8.523548000000	-2.665623000000
N	6.531308000000	-5.773850000000	-1.304141000000
C	7.453602000000	-5.293958000000	-0.295791000000
C	7.019353000000	-5.528841000000	1.160328000000
O	7.549112000000	-4.877712000000	2.067088000000
H	5.642404000000	-5.293839000000	-1.407154000000
H	7.627578000000	-4.221271000000	-0.412308000000
H	8.404153000000	-5.813131000000	-0.451480000000
N	6.079771000000	-6.481269000000	1.357717000000
C	5.615605000000	-6.851668000000	2.681697000000
H	5.627654000000	-6.919296000000	0.559610000000
H	5.557272000000	-7.941316000000	2.765971000000
H	6.327449000000	-6.466791000000	3.413995000000
H	4.622538000000	-6.433377000000	2.892298000000
H	5.925345000000	-7.340499000000	-3.920143000000
H	1.518465000000	-7.027470000000	-1.110841000000
S	3.296808000000	-3.737875000000	-1.414159000000
Fe	2.890797000000	-1.648474000000	-0.236111000000
C	5.477386000000	-0.230748000000	-1.958601000000
C	0.792295000000	-1.021264000000	-2.860337000000
C	0.266041000000	-2.858667000000	1.578279000000
C	5.085841000000	-2.845750000000	2.087529000000
N	3.096176000000	-0.801291000000	-2.050305000000
C	4.229478000000	-0.223698000000	-2.566475000000
C	3.952676000000	0.345469000000	-3.860904000000
C	2.643382000000	0.087994000000	-4.131054000000
C	2.114629000000	-0.616999000000	-2.990834000000
N	0.932212000000	-1.924889000000	-0.585900000000
C	0.244267000000	-1.617516000000	-1.732485000000
C	-1.155655000000	-1.927541000000	-1.573783000000
C	-1.320192000000	-2.381140000000	-0.300300000000
C	-0.007714000000	-2.384603000000	0.300807000000
N	2.710683000000	-2.656462000000	1.485454000000
C	1.541895000000	-3.016476000000	2.110831000000
C	1.829505000000	-3.652863000000	3.369418000000
C	3.188998000000	-3.688746000000	3.489661000000
C	3.726082000000	-3.052970000000	2.315618000000
N	4.890806000000	-1.560416000000	0.008975000000
C	5.616081000000	-2.129725000000	1.026097000000
C	7.020070000000	-1.833794000000	0.865255000000
C	7.124657000000	-1.051743000000	-0.245380000000
C	5.790122000000	-0.902423000000	-0.775086000000
H	6.288227000000	0.270577000000	-2.476697000000
H	0.123062000000	-0.813690000000	-3.689049000000

H	-0.579022000000	-3.163733000000	2.188330000000
H	5.791146000000	-3.274461000000	2.791412000000
H	-2.231650000000	-2.672274000000	0.208979000000
H	-1.904382000000	-1.794070000000	-2.343210000000
H	1.080353000000	-4.042373000000	4.046937000000
H	3.786392000000	-4.097719000000	4.293998000000
H	2.069632000000	0.351811000000	-5.009821000000
H	4.680261000000	0.860528000000	-4.474343000000
H	8.014893000000	-0.627997000000	-0.691880000000
H	7.795472000000	-2.218908000000	1.513455000000
O	2.676489000000	-0.139799000000	0.523225000000
C	5.037470000000	2.218307000000	0.356951000000
C	6.948088000000	6.720580000000	0.334848000000
C	6.571120000000	3.881185000000	-0.566720000000
C	6.271269000000	2.866028000000	0.353799000000
C	4.028911000000	2.572432000000	-0.574901000000
C	4.337767000000	3.584947000000	-1.521318000000
C	5.566938000000	4.219950000000	-1.502847000000
H	8.115998000000	4.325362000000	-2.820172000000
H	9.674049000000	4.489384000000	-1.983226000000
C	8.837787000000	4.248670000000	0.576649000000
C	8.662899000000	4.073807000000	-1.905821000000
C	7.937637000000	4.586074000000	-0.636507000000
C	7.730555000000	6.113970000000	-0.660862000000
C	8.322253000000	6.957761000000	-1.611820000000
C	8.148819000000	8.341173000000	-1.575801000000
C	7.369511000000	8.920917000000	-0.572498000000
C	6.766254000000	8.099962000000	0.387295000000
H	8.934680000000	6.543266000000	-2.405525000000
H	8.611227000000	8.982599000000	-2.319499000000
O	7.230092000000	10.280596000000	-0.580430000000
H	6.151729000000	8.539324000000	1.171677000000
H	6.459352000000	6.100342000000	1.081766000000
O	2.826019000000	2.018187000000	-0.571149000000
H	4.826544000000	1.436173000000	1.079954000000
H	3.582052000000	3.852661000000	-2.254467000000
H	5.763961000000	5.009258000000	-2.223625000000
H	2.753028000000	0.871133000000	-0.025884000000
H	7.008771000000	2.563623000000	1.089846000000
H	6.667368000000	10.545791000000	0.163671000000
H	8.754890000000	2.983396000000	-1.859898000000
H	8.366049000000	4.525810000000	1.524934000000
H	9.082686000000	3.180776000000	0.612712000000
H	9.778080000000	4.803852000000	0.498948000000
C	-0.715027000000	4.635542000000	6.407274000000
C	-0.712756000000	3.641314000000	5.263339000000
O	-1.752984000000	3.095196000000	4.880168000000
H	0.285926000000	4.888039000000	6.768963000000
H	-1.209612000000	5.553914000000	6.072036000000
N	0.493857000000	3.367959000000	4.693585000000
C	0.620601000000	2.642744000000	3.421910000000
C	0.040720000000	1.226559000000	3.487886000000
O	-0.439175000000	0.688466000000	2.490051000000
C	2.094863000000	2.592437000000	3.002558000000
H	1.287371000000	3.930711000000	4.972862000000
H	0.041635000000	3.156138000000	2.648316000000
H	2.461527000000	3.600134000000	2.782924000000

H	2.712058000000	2.142273000000	3.789077000000
H	2.203436000000	1.993018000000	2.097064000000
N	0.104185000000	0.570475000000	4.674780000000
C	-0.433081000000	-0.774447000000	4.781233000000
C	-1.950990000000	-0.873680000000	4.556332000000
O	-2.435424000000	-1.941142000000	4.170354000000
H	0.516302000000	1.030739000000	5.473436000000
H	-0.209419000000	-1.158927000000	5.781369000000
H	0.040253000000	-1.430756000000	4.049456000000
N	-2.671382000000	0.222235000000	4.891304000000
C	-4.121118000000	0.247119000000	4.846016000000
C	-4.796798000000	1.076490000000	3.742650000000
O	-5.941366000000	0.773880000000	3.406852000000
H	-2.177221000000	1.076885000000	5.128814000000
H	-4.470541000000	-0.778935000000	4.734403000000
N	-4.137301000000	2.148765000000	3.243728000000
C	-4.830711000000	3.112790000000	2.389610000000
C	-5.298529000000	2.530329000000	1.044748000000
O	-6.490662000000	2.508826000000	0.743771000000
H	-3.219413000000	2.393600000000	3.597264000000
H	-5.729750000000	3.486993000000	2.886627000000
N	-4.322792000000	2.021701000000	0.248977000000
C	-4.703449000000	1.405520000000	-1.017296000000
C	-5.631750000000	0.211273000000	-0.757657000000
O	-6.594109000000	-0.020746000000	-1.501070000000
C	-3.455386000000	0.946442000000	-1.822722000000
O	-2.542225000000	2.015505000000	-2.001923000000
C	-3.846557000000	0.430088000000	-3.204969000000
H	-3.343460000000	2.245439000000	0.432940000000
H	-5.276985000000	2.112739000000	-1.625125000000
H	-2.970052000000	0.139387000000	-1.252976000000
H	-2.018837000000	2.108413000000	-1.175281000000
H	-2.944769000000	0.106261000000	-3.735234000000
H	-4.307026000000	1.234765000000	-3.787908000000
H	-4.552413000000	-0.403778000000	-3.158665000000
N	-5.309892000000	-0.574404000000	0.290904000000
C	-5.994914000000	-1.823482000000	0.599068000000
C	-7.512099000000	-1.627871000000	0.820767000000
O	-8.320249000000	-2.458535000000	0.402020000000
C	-5.346258000000	-2.436157000000	1.868912000000
O	-3.931131000000	-2.237903000000	1.760846000000
C	-5.696083000000	-3.914007000000	2.019842000000
H	-4.454732000000	-0.404912000000	0.807225000000
H	-5.909330000000	-2.513150000000	-0.248716000000
H	-5.718342000000	-1.870661000000	2.734369000000
H	-3.531895000000	-2.246958000000	2.654092000000
H	-5.295399000000	-4.306069000000	2.961378000000
H	-6.780083000000	-4.066524000000	2.009728000000
H	-5.260472000000	-4.486294000000	1.193822000000
N	-7.874965000000	-0.520899000000	1.513132000000
C	-9.271913000000	-0.194977000000	1.748250000000
C	-10.042818000000	0.399145000000	0.550776000000
O	-11.239583000000	0.634070000000	0.660639000000
H	-7.166312000000	0.119970000000	1.858734000000
H	-9.815601000000	-1.095900000000	2.045922000000
N	-9.306364000000	0.584135000000	-0.574872000000
C	-9.840310000000	1.063373000000	-1.834890000000

C	-9.713186000000	-0.010790000000	-2.944999000000
O	-9.824863000000	0.281131000000	-4.134077000000
C	-9.136322000000	2.398068000000	-2.263685000000
O	-8.907952000000	3.238780000000	-1.143429000000
C	-9.984389000000	3.200279000000	-3.243154000000
H	-8.315240000000	0.372521000000	-0.558876000000
H	-10.903480000000	1.262074000000	-1.661143000000
H	-8.180902000000	2.128657000000	-2.740374000000
H	-8.235745000000	2.831192000000	-0.571237000000
H	-9.448850000000	4.110166000000	-3.535000000000
H	-10.921499000000	3.499891000000	-2.758457000000
H	-10.208487000000	2.602552000000	-4.127802000000
N	-9.458228000000	-1.279644000000	-2.516273000000
C	-9.207676000000	-2.387650000000	-3.425677000000
C	-8.018745000000	-3.241396000000	-2.954098000000
O	-6.808513000000	-2.491743000000	-2.914407000000
C	-7.784860000000	-4.423353000000	-3.889227000000
H	-9.412145000000	-1.460776000000	-1.520354000000
H	-9.005823000000	-1.956231000000	-4.409931000000
H	-8.249553000000	-3.619618000000	-1.945040000000
H	-6.976449000000	-1.658082000000	-2.435669000000
H	-6.924670000000	-5.007560000000	-3.546108000000
H	-8.660583000000	-5.081487000000	-3.921334000000
H	-7.572692000000	-4.070198000000	-4.905089000000
C	1.474458000000	6.482339000000	-2.902932000000
C	1.028106000000	5.434687000000	-3.930434000000
C	-0.059503000000	4.460556000000	-3.436531000000
C	-1.398032000000	5.158247000000	-3.145302000000
C	-0.264657000000	3.310106000000	-4.433242000000
H	1.869595000000	6.005288000000	-1.994059000000
H	0.664769000000	5.943380000000	-4.835319000000
H	1.906094000000	4.851407000000	-4.244613000000
H	0.297724000000	4.010227000000	-2.495567000000
H	0.675217000000	2.772528000000	-4.615158000000
H	-1.002832000000	2.599963000000	-4.046884000000
H	-0.625879000000	3.689530000000	-5.398428000000
H	-1.301049000000	5.944850000000	-2.387034000000
H	-1.794489000000	5.626740000000	-4.055993000000
H	-2.136765000000	4.432214000000	-2.789929000000
O	-1.426939000000	2.395951000000	0.513220000000
H	-1.065002000000	1.668923000000	1.064459000000
H	-0.649001000000	2.983817000000	0.335393000000
O	0.880175000000	3.812831000000	0.156475000000
H	1.544378000000	3.156800000000	-0.177311000000
H	0.833836000000	4.498607000000	-0.527136000000
H	-10.100384000000	-3.025328000000	-3.512827000000
H	0.652316000000	7.141703000000	-2.603742000000
H	-1.307486000000	4.230742000000	7.232119000000
H	2.273755000000	7.116394000000	-3.303527000000
H	-4.507855000000	0.622714000000	5.802363000000
H	-9.325431000000	0.521728000000	2.571400000000
H	-4.141429000000	3.944912000000	2.217299000000

^{2}I6$_{B}$

C	0.628758000000	-0.927879000000	0.516926000000
C	0.362887000000	-0.950912000000	2.016510000000
O	1.161191000000	-1.444575000000	2.814636000000
C	0.704419000000	-2.349845000000	-0.060778000000
H	-0.127231000000	-0.344422000000	-0.014575000000
H	1.506006000000	-2.898591000000	0.445367000000
H	0.938912000000	-2.315755000000	-1.126987000000
N	-0.825724000000	-0.414929000000	2.417339000000
C	-1.217723000000	-0.368226000000	3.814389000000
C	-1.623276000000	-1.693345000000	4.484400000000
O	-1.876263000000	-1.695367000000	5.683728000000
H	-1.490444000000	-0.115727000000	1.716376000000
H	-0.385279000000	0.017080000000	4.410596000000
N	-1.658701000000	-2.796768000000	3.689507000000
C	-1.790751000000	-4.131032000000	4.240700000000
C	-0.486622000000	-4.935635000000	4.390353000000
O	-0.551852000000	-6.110844000000	4.740626000000
H	-1.448266000000	-2.711835000000	2.699490000000
H	-2.465041000000	-4.730837000000	3.621989000000
H	-2.233634000000	-4.037126000000	5.235481000000
N	0.668086000000	-4.272455000000	4.127351000000
C	1.963780000000	-4.912034000000	4.254485000000
H	0.643394000000	-3.298911000000	3.842498000000
H	2.549205000000	-4.780205000000	3.337726000000
H	2.534159000000	-4.496287000000	5.094931000000
H	1.795592000000	-5.975631000000	4.431071000000
H	-2.051683000000	0.330783000000	3.919537000000
H	1.599904000000	-0.437783000000	0.367694000000
S	-0.799807000000	-3.371506000000	0.215200000000
Fe	-2.514139000000	-2.999978000000	-1.380045000000
C	-2.426771000000	0.427879000000	-1.336059000000
C	-0.245501000000	-3.028896000000	-3.944994000000
C	-2.634046000000	-6.428473000000	-1.456186000000
C	-4.563667000000	-2.980757000000	1.361059000000
N	-1.470860000000	-1.588875000000	-2.373781000000
C	-1.622205000000	-0.226419000000	-2.258303000000
C	-0.805352000000	0.442273000000	-3.238581000000
C	-0.170278000000	-0.528201000000	-3.954976000000
C	-0.608357000000	-1.793800000000	-3.423929000000
N	-1.683763000000	-4.431817000000	-2.531026000000
C	-0.748924000000	-4.249760000000	-3.520825000000
C	-0.335227000000	-5.525864000000	-4.050366000000
C	-1.012879000000	-6.483128000000	-3.360878000000
C	-1.844164000000	-5.793114000000	-2.401722000000
N	-3.404073000000	-4.416464000000	-0.264363000000
C	-3.343574000000	-5.774040000000	-0.457199000000
C	-4.119810000000	-6.449660000000	0.553539000000
C	-4.650543000000	-5.481064000000	1.352521000000
C	-4.207056000000	-4.211752000000	0.830578000000
N	-3.336140000000	-1.558503000000	-0.218079000000
C	-4.166004000000	-1.750703000000	0.855925000000
C	-4.571145000000	-0.477696000000	1.398265000000
C	-3.984857000000	0.483844000000	0.628462000000
C	-3.210714000000	-0.203669000000	-0.378300000000
H	-2.433325000000	1.513155000000	-1.356657000000
H	0.475500000000	-3.039976000000	-4.756015000000

H	-2.687774000000	-7.513063000000	-1.473725000000
H	-5.207177000000	-2.979621000000	2.234724000000
H	-0.922313000000	-7.556739000000	-3.470982000000
H	0.393235000000	-5.662479000000	-4.839238000000
H	-4.227261000000	-7.523970000000	0.629242000000
H	-5.279780000000	-5.596311000000	2.226258000000
H	0.527584000000	-0.414394000000	-4.774951000000
H	-0.736308000000	1.517047000000	-3.349878000000
H	-4.063398000000	1.560276000000	0.717391000000
H	-5.226442000000	-0.352656000000	2.250933000000
O	-3.920536000000	-2.814974000000	-2.464172000000
C	-3.402585000000	-2.669188000000	-5.788976000000
C	-4.684397000000	-1.118603000000	-10.193697000000
C	-3.105565000000	-2.722120000000	-8.208402000000
C	-2.865145000000	-2.137270000000	-6.942026000000
C	-4.244562000000	-3.844491000000	-5.841671000000
C	-4.488314000000	-4.435876000000	-7.141498000000
C	-3.933916000000	-3.881882000000	-8.266870000000
H	-1.918440000000	-4.218581000000	-10.201303000000
H	-0.943069000000	-2.906195000000	-10.892148000000
C	-1.719754000000	-0.864379000000	-9.306131000000
C	-1.454794000000	-3.251168000000	-9.987172000000
C	-2.483236000000	-2.194437000000	-9.507173000000
C	-3.611774000000	-1.960114000000	-10.531511000000
C	-3.616398000000	-2.524931000000	-11.815389000000
C	-4.637013000000	-2.260452000000	-12.726953000000
C	-5.691529000000	-1.415877000000	-12.370062000000
C	-5.711695000000	-0.844464000000	-11.092371000000
H	-2.813783000000	-3.184451000000	-12.126668000000
H	-4.629243000000	-2.701736000000	-13.718532000000
O	-6.665138000000	-1.189692000000	-13.299734000000
H	-6.529042000000	-0.186971000000	-10.800869000000
H	-4.725180000000	-0.668481000000	-9.204860000000
O	-4.759158000000	-4.329344000000	-4.785610000000
H	-3.233998000000	-2.219159000000	-4.815794000000
H	-5.061827000000	-5.358491000000	-7.182921000000
H	-4.124827000000	-4.337143000000	-9.234346000000
H	-4.054959000000	-3.551730000000	-3.099405000000
H	-2.242573000000	-1.252757000000	-6.864668000000
H	-7.327173000000	-0.585790000000	-12.928317000000
H	-0.695740000000	-3.409245000000	-9.214175000000
H	-2.364320000000	-0.072522000000	-8.911546000000
H	-0.868397000000	-0.989209000000	-8.627190000000
H	-1.330652000000	-0.523601000000	-10.270436000000
C	-12.379456000000	-9.993285000000	-1.811465000000
C	-10.999462000000	-9.427080000000	-2.076399000000
O	-10.013663000000	-10.159694000000	-2.214294000000
H	-12.721409000000	-10.517673000000	-2.710468000000
H	-12.314588000000	-10.733744000000	-1.009521000000
N	-10.893803000000	-8.069373000000	-2.138840000000
C	-9.681908000000	-7.414257000000	-2.646750000000
C	-8.457571000000	-7.672499000000	-1.758287000000
O	-7.320253000000	-7.670926000000	-2.232733000000
C	-9.921261000000	-5.907508000000	-2.809113000000
H	-11.746164000000	-7.523789000000	-2.136739000000
H	-9.417133000000	-7.844485000000	-3.618686000000
H	-10.775482000000	-5.731107000000	-3.473411000000

H	-10.119339000000	-5.426721000000	-1.844376000000
H	-9.043655000000	-5.441758000000	-3.265839000000
N	-8.665963000000	-7.881585000000	-0.434940000000
C	-7.541910000000	-8.184250000000	0.436838000000
C	-6.852339000000	-9.529506000000	0.156067000000
O	-5.706427000000	-9.714291000000	0.575154000000
H	-9.612255000000	-7.889717000000	-0.082169000000
H	-7.893369000000	-8.187260000000	1.473025000000
H	-6.773562000000	-7.413504000000	0.341313000000
N	-7.586694000000	-10.465072000000	-0.487514000000
C	-7.059380000000	-11.779873000000	-0.806948000000
C	-6.614041000000	-12.017674000000	-2.260835000000
O	-5.674758000000	-12.774103000000	-2.489811000000
H	-8.502379000000	-10.214940000000	-0.846617000000
H	-6.192898000000	-11.963656000000	-0.172510000000
N	-7.340873000000	-11.425657000000	-3.248161000000
C	-7.161323000000	-11.839031000000	-4.639010000000
C	-5.775905000000	-11.505749000000	-5.221696000000
O	-4.996794000000	-12.390841000000	-5.567782000000
H	-8.160576000000	-10.877632000000	-3.013075000000
H	-7.272599000000	-12.922929000000	-4.734466000000
N	-5.464695000000	-10.182823000000	-5.304778000000
C	-4.182830000000	-9.786585000000	-5.885685000000
C	-3.020778000000	-10.351146000000	-5.057429000000
O	-1.948278000000	-10.660542000000	-5.600616000000
C	-4.087009000000	-8.239067000000	-6.020356000000
O	-5.191986000000	-7.749729000000	-6.771091000000
C	-2.815105000000	-7.815396000000	-6.745622000000
H	-6.204366000000	-9.489373000000	-5.220824000000
H	-4.073901000000	-10.224316000000	-6.883744000000
H	-4.096526000000	-7.801480000000	-5.008774000000
H	-5.976708000000	-7.764211000000	-6.185043000000
H	-2.781496000000	-6.724274000000	-6.805950000000
H	-2.813780000000	-8.218615000000	-7.763977000000
H	-1.914795000000	-8.165826000000	-6.237749000000
N	-3.231077000000	-10.471316000000	-3.734314000000
C	-2.228675000000	-10.969730000000	-2.802392000000
C	-1.702215000000	-12.362394000000	-3.199895000000
O	-0.528927000000	-12.674507000000	-2.974584000000
C	-2.855083000000	-11.002059000000	-1.385260000000
O	-3.585793000000	-9.778811000000	-1.252812000000
C	-1.799003000000	-11.150150000000	-0.294034000000
H	-4.076932000000	-10.099574000000	-3.318064000000
H	-1.356873000000	-10.307019000000	-2.807926000000
H	-3.558407000000	-11.845480000000	-1.350669000000
H	-4.231619000000	-9.865689000000	-0.523000000000
H	-2.280275000000	-11.211511000000	0.688222000000
H	-1.195754000000	-12.050820000000	-0.444120000000
H	-1.131589000000	-10.281781000000	-0.294404000000
N	-2.585098000000	-13.208720000000	-3.779436000000
C	-2.169405000000	-14.531247000000	-4.213428000000
C	-1.186699000000	-14.581308000000	-5.399990000000
O	-0.585788000000	-15.618340000000	-5.642322000000
H	-3.533933000000	-12.906668000000	-3.981842000000
H	-1.674893000000	-15.062322000000	-3.395856000000
N	-1.067482000000	-13.430611000000	-6.129347000000
C	-0.084890000000	-13.270730000000	-7.194430000000

C	0.973927000000	-12.200369000000	-6.834717000000
O	1.596151000000	-11.599443000000	-7.711223000000
C	-0.795161000000	-12.955008000000	-8.547324000000
O	-1.947235000000	-13.774672000000	-8.694500000000
C	0.082064000000	-13.225515000000	-9.763508000000
H	-1.548495000000	-12.589272000000	-5.819821000000
H	0.418882000000	-14.240315000000	-7.281915000000
H	-1.086653000000	-11.891434000000	-8.531610000000
H	-2.421121000000	-13.747712000000	-7.846638000000
H	-0.496070000000	-13.038658000000	-10.674931000000
H	0.392764000000	-14.277041000000	-9.771289000000
H	0.964840000000	-12.585902000000	-9.749696000000
N	1.172255000000	-11.984936000000	-5.505143000000
C	2.123163000000	-11.005052000000	-5.000690000000
C	1.487499000000	-10.070785000000	-3.958831000000
O	0.387917000000	-9.338791000000	-4.508739000000
C	2.494933000000	-9.050983000000	-3.441593000000
H	0.650139000000	-12.526793000000	-4.823655000000
H	2.480258000000	-10.433792000000	-5.860065000000
H	1.129974000000	-10.687391000000	-3.120341000000
H	-0.220773000000	-9.964268000000	-4.949891000000
H	2.017625000000	-8.381111000000	-2.719188000000
H	3.336872000000	-9.548170000000	-2.947412000000
H	2.882977000000	-8.442989000000	-4.267146000000
C	3.642464000000	-5.392892000000	-9.019746000000
C	2.430908000000	-6.039120000000	-8.336937000000
C	2.242298000000	-7.541700000000	-8.627227000000
C	3.422202000000	-8.389937000000	-8.126050000000
C	0.923141000000	-8.053401000000	-8.028839000000
H	3.600044000000	-5.527992000000	-10.108141000000
H	2.506598000000	-5.897018000000	-7.247412000000
H	1.520426000000	-5.506249000000	-8.650356000000
H	2.182968000000	-7.660590000000	-9.721202000000
H	0.068688000000	-7.465102000000	-8.389219000000
H	0.756157000000	-9.102747000000	-8.292798000000
H	0.933570000000	-8.000067000000	-6.932222000000
H	4.356818000000	-8.147383000000	-8.644864000000
H	3.586029000000	-8.224006000000	-7.051773000000
H	3.218876000000	-9.456619000000	-8.267206000000
O	-7.378249000000	-7.858622000000	-4.985072000000
H	-7.184938000000	-7.817067000000	-4.020029000000
H	-7.511279000000	-6.902143000000	-5.205209000000
O	-7.383125000000	-5.153673000000	-5.146288000000
H	-6.438205000000	-4.951910000000	-4.936537000000
H	-7.577516000000	-4.627502000000	-5.936144000000
H	2.984972000000	-11.514182000000	-4.545384000000
H	4.586107000000	-5.825796000000	-8.670267000000
H	-13.119786000000	-9.233756000000	-1.544176000000
H	3.682328000000	-4.314882000000	-8.820784000000
H	-7.817972000000	-12.536379000000	-0.569171000000
H	-3.066120000000	-15.092984000000	-4.488827000000
H	-7.940484000000	-11.343905000000	-5.226976000000

^{4}I6$_{B}$

C	0.917658000000	-3.950530000000	-0.346408000000
C	1.698732000000	-2.848116000000	-1.050407000000
O	2.466274000000	-2.102520000000	-0.441129000000
C	0.051494000000	-3.383905000000	0.788756000000
H	0.303224000000	-4.521002000000	-1.047778000000
H	0.698493000000	-2.869899000000	1.506763000000
H	-0.476263000000	-4.187661000000	1.305276000000
N	1.470573000000	-2.718337000000	-2.389071000000
C	2.182912000000	-1.744119000000	-3.198887000000
C	1.779988000000	-0.267088000000	-3.043129000000
O	2.406159000000	0.585879000000	-3.659389000000
H	0.792785000000	-3.322191000000	-2.834515000000
H	3.249841000000	-1.783765000000	-2.959269000000
N	0.743423000000	0.000011000000	-2.200451000000
C	0.428018000000	1.350843000000	-1.778448000000
C	0.866638000000	1.733029000000	-0.353664000000
O	0.442371000000	2.775142000000	0.139079000000
H	0.282952000000	-0.766240000000	-1.722504000000
H	-0.649466000000	1.532091000000	-1.835764000000
H	0.924678000000	2.031881000000	-2.474972000000
N	1.727033000000	0.882913000000	0.262070000000
C	2.237439000000	1.140274000000	1.595395000000
H	2.014577000000	0.027030000000	-0.200645000000
H	2.043998000000	0.285933000000	2.253905000000
H	3.317913000000	1.329812000000	1.577925000000
H	1.727194000000	2.023305000000	1.983321000000
H	2.072569000000	-2.014756000000	-4.252207000000
H	1.650367000000	-4.641429000000	0.091226000000
S	-1.148747000000	-2.100849000000	0.239071000000
Fe	-3.259457000000	-2.915567000000	-0.285510000000
C	-2.688630000000	-1.256515000000	-3.249735000000
C	-2.068920000000	-5.804923000000	-1.699148000000
C	-3.624916000000	-4.512238000000	2.707801000000
C	-4.491519000000	-0.039598000000	1.084345000000
N	-2.584146000000	-3.442590000000	-2.135659000000
C	-2.402449000000	-2.615224000000	-3.217073000000
C	-1.839839000000	-3.362207000000	-4.316620000000
C	-1.678467000000	-4.649522000000	-3.884018000000
C	-2.139892000000	-4.682298000000	-2.515876000000
N	-2.846830000000	-4.753700000000	0.388140000000
C	-2.396063000000	-5.824128000000	-0.352353000000
C	-2.308566000000	-6.996214000000	0.481515000000
C	-2.727496000000	-6.629876000000	1.724267000000
C	-3.082211000000	-5.234202000000	1.652586000000
N	-3.971570000000	-2.400068000000	1.515544000000
C	-4.046994000000	-3.194687000000	2.629403000000
C	-4.617286000000	-2.452091000000	3.729752000000
C	-4.873092000000	-1.199543000000	3.267214000000
C	-4.454050000000	-1.170384000000	1.884668000000
N	-3.501370000000	-1.018514000000	-0.939332000000
C	-4.033122000000	0.024951000000	-0.225457000000
C	-4.057564000000	1.215158000000	-1.038452000000
C	-3.541812000000	0.875010000000	-2.252950000000
C	-3.205492000000	-0.525992000000	-2.188083000000
H	-2.489900000000	-0.723480000000	-4.173858000000
H	-1.715271000000	-6.721924000000	-2.158641000000

H	-3.742621000000	-5.024211000000	3.657462000000
H	-4.883697000000	0.872563000000	1.522153000000
H	-2.805948000000	-7.238003000000	2.616489000000
H	-1.973246000000	-7.966816000000	0.139659000000
H	-4.783046000000	-2.853457000000	4.721371000000
H	-5.290181000000	-0.354516000000	3.799891000000
H	-1.274936000000	-5.510092000000	-4.404635000000
H	-1.594998000000	-2.941131000000	-5.284219000000
H	-3.394763000000	1.502416000000	-3.122870000000
H	-4.412466000000	2.181162000000	-0.703065000000
O	-4.918064000000	-3.370426000000	-0.771013000000
C	-6.574257000000	-1.497933000000	-3.144808000000
C	-10.136629000000	0.100315000000	-6.087508000000
C	-7.311543000000	-0.204753000000	-5.076272000000
C	-7.096481000000	-0.341275000000	-3.683414000000
C	-6.229024000000	-2.615752000000	-3.994728000000
C	-6.458700000000	-2.471165000000	-5.416560000000
C	-6.985128000000	-1.309616000000	-5.918825000000
H	-6.267772000000	1.047779000000	-7.295399000000
H	-6.926867000000	2.660798000000	-6.955568000000
C	-8.281283000000	2.136451000000	-4.677909000000
C	-6.634356000000	1.707295000000	-6.502947000000
C	-7.822799000000	1.089590000000	-5.720395000000
C	-9.016986000000	0.749469000000	-6.633351000000
C	-9.064961000000	1.080279000000	-7.995732000000
C	-10.179140000000	0.788354000000	-8.780007000000
C	-11.284883000000	0.147866000000	-8.213384000000
C	-11.257412000000	-0.198512000000	-6.857064000000
H	-8.226397000000	1.579534000000	-8.468721000000
H	-10.207005000000	1.052155000000	-9.832384000000
O	-12.349010000000	-0.112607000000	-9.025086000000
H	-12.111215000000	-0.699819000000	-6.404489000000
H	-10.136599000000	-0.182071000000	-5.037563000000
O	-5.741521000000	-3.683104000000	-3.505258000000
H	-6.387452000000	-1.604026000000	-2.080177000000
H	-6.193569000000	-3.307345000000	-6.057350000000
H	-7.164880000000	-1.220405000000	-6.986157000000
H	-4.979232000000	-3.541002000000	-1.736357000000
H	-7.331872000000	0.482663000000	-3.019509000000
H	-13.047090000000	-0.548046000000	-8.511171000000
H	-5.802783000000	1.903752000000	-5.818543000000
H	-9.083520000000	1.758567000000	-4.036149000000
H	-7.450989000000	2.460159000000	-4.040078000000
H	-8.664433000000	3.018561000000	-5.199623000000
C	-10.251199000000	-8.418805000000	-3.526347000000
C	-8.837024000000	-8.168756000000	-3.043304000000
O	-7.876904000000	-8.821572000000	-3.466421000000
H	-10.326806000000	-8.094812000000	-4.570230000000
H	-10.453167000000	-9.493142000000	-3.504433000000
N	-8.670069000000	-7.194565000000	-2.104831000000
C	-7.345100000000	-6.649563000000	-1.772993000000
C	-6.394204000000	-7.707877000000	-1.194592000000
O	-5.186198000000	-7.655216000000	-1.423277000000
C	-7.501510000000	-5.473207000000	-0.801395000000
H	-9.467572000000	-6.615096000000	-1.875566000000
H	-6.858238000000	-6.303040000000	-2.690523000000
H	-8.133543000000	-4.697859000000	-1.250081000000

H	-7.957069000000	-5.797509000000	0.141540000000
H	-6.530505000000	-5.016427000000	-0.588452000000
N	-6.938461000000	-8.653991000000	-0.385363000000
C	-6.147216000000	-9.742385000000	0.163842000000
C	-6.115755000000	-11.041008000000	-0.659602000000
O	-5.577126000000	-12.039418000000	-0.178619000000
H	-7.941887000000	-8.656589000000	-0.261189000000
H	-6.510170000000	-10.001650000000	1.162137000000
H	-5.117436000000	-9.390795000000	0.263882000000
N	-6.712086000000	-10.998745000000	-1.872648000000
C	-6.781787000000	-12.157227000000	-2.747317000000
C	-5.897200000000	-12.100139000000	-4.003658000000
O	-5.232449000000	-13.081798000000	-4.331519000000
H	-7.093411000000	-10.120031000000	-2.208668000000
H	-6.470079000000	-13.029862000000	-2.174814000000
N	-5.943930000000	-10.964596000000	-4.745459000000
C	-5.268311000000	-10.905492000000	-6.038214000000
C	-3.740547000000	-11.067503000000	-5.957731000000
O	-3.155236000000	-11.889903000000	-6.659558000000
H	-6.512609000000	-10.178696000000	-4.450816000000
H	-5.621893000000	-11.706321000000	-6.692994000000
N	-3.110123000000	-10.258940000000	-5.070022000000
C	-1.659612000000	-10.341462000000	-4.923760000000
C	-1.254774000000	-11.755628000000	-4.474779000000
O	-0.291541000000	-12.335620000000	-4.990217000000
C	-1.135931000000	-9.280277000000	-3.919192000000
O	-1.500890000000	-7.962718000000	-4.322563000000
C	0.384507000000	-9.318905000000	-3.800871000000
H	-3.629585000000	-9.488270000000	-4.646138000000
H	-1.173106000000	-10.181912000000	-5.891358000000
H	-1.582347000000	-9.498464000000	-2.934014000000
H	-2.473816000000	-7.851070000000	-4.199823000000
H	0.710726000000	-8.558520000000	-3.084537000000
H	0.843217000000	-9.096496000000	-4.770058000000
H	0.749373000000	-10.294227000000	-3.467612000000
N	-2.000442000000	-12.291814000000	-3.488918000000
C	-1.703785000000	-13.570540000000	-2.855413000000
C	-1.713010000000	-14.737706000000	-3.866118000000
O	-0.895234000000	-15.655391000000	-3.779619000000
C	-2.759477000000	-13.804028000000	-1.742556000000
O	-2.942821000000	-12.549839000000	-1.081737000000
C	-2.316937000000	-14.894112000000	-0.769980000000
H	-2.689243000000	-11.726734000000	-3.006403000000
H	-0.693632000000	-13.550773000000	-2.430980000000
H	-3.699661000000	-14.095550000000	-2.232892000000
H	-3.815785000000	-12.551445000000	-0.644147000000
H	-3.108717000000	-15.094677000000	-0.039444000000
H	-2.082405000000	-15.824897000000	-1.296617000000
H	-1.422703000000	-14.567617000000	-0.228795000000
N	-2.674483000000	-14.691106000000	-4.821249000000
C	-2.753248000000	-15.696209000000	-5.867767000000
C	-1.699181000000	-15.598284000000	-6.990451000000
O	-1.650302000000	-16.472818000000	-7.846435000000
H	-3.338428000000	-13.923349000000	-4.836604000000
H	-2.641870000000	-16.693291000000	-5.432713000000
N	-0.865344000000	-14.528818000000	-6.915292000000
C	0.240940000000	-14.291361000000	-7.823605000000

C	1.600858000000	-14.347204000000	-7.082540000000
O	2.624637000000	-13.889867000000	-7.587291000000
C	0.056838000000	-12.926768000000	-8.572093000000
O	-1.296887000000	-12.734383000000	-8.950548000000
C	0.878996000000	-12.858139000000	-9.853529000000
H	-0.996411000000	-13.848075000000	-6.175950000000
H	0.212227000000	-15.103868000000	-8.557799000000
H	0.378980000000	-12.128083000000	-7.884911000000
H	-1.833778000000	-12.614253000000	-8.148810000000
H	0.728502000000	-11.885516000000	-10.334726000000
H	0.540447000000	-13.635245000000	-10.549577000000
H	1.937314000000	-13.004433000000	-9.634379000000
N	1.580213000000	-14.922499000000	-5.847145000000
C	2.752050000000	-14.988807000000	-4.987352000000
C	2.399975000000	-14.639376000000	-3.531713000000
O	1.907495000000	-13.308846000000	-3.409261000000
C	3.624342000000	-14.749691000000	-2.629126000000
H	0.714036000000	-15.319365000000	-5.501697000000
H	3.484227000000	-14.281888000000	-5.386850000000
H	1.631656000000	-15.350386000000	-3.187044000000
H	1.218822000000	-13.167962000000	-4.085969000000
H	3.357769000000	-14.478379000000	-1.602452000000
H	4.022377000000	-15.770793000000	-2.626497000000
H	4.411007000000	-14.065770000000	-2.968241000000
C	-3.242625000000	-4.448711000000	-9.526075000000
C	-1.918779000000	-5.194494000000	-9.320748000000
C	-1.795944000000	-5.946730000000	-7.980945000000
C	-2.851535000000	-7.052689000000	-7.822374000000
C	-0.383896000000	-6.525471000000	-7.805274000000
H	-3.428383000000	-3.738403000000	-8.709021000000
H	-1.771909000000	-5.911264000000	-10.141851000000
H	-1.090204000000	-4.476044000000	-9.395059000000
H	-1.956353000000	-5.207301000000	-7.175700000000
H	0.382487000000	-5.743238000000	-7.871741000000
H	-0.283811000000	-7.023862000000	-6.834439000000
H	-0.168193000000	-7.267634000000	-8.584584000000
H	-3.871725000000	-6.653468000000	-7.817242000000
H	-2.773629000000	-7.781052000000	-8.640228000000
H	-2.698292000000	-7.594057000000	-6.882049000000
O	-4.240475000000	-7.823518000000	-4.038888000000
H	-4.524647000000	-7.665893000000	-3.113935000000
H	-4.504809000000	-7.021124000000	-4.559783000000
O	-4.741231000000	-5.482656000000	-5.344723000000
H	-5.108136000000	-4.873217000000	-4.660907000000
H	-3.828936000000	-5.172650000000	-5.470576000000
H	3.199143000000	-15.993937000000	-5.021637000000
H	-4.096025000000	-5.134191000000	-9.568265000000
H	-11.011986000000	-7.895861000000	-2.939737000000
H	-3.231447000000	-3.879423000000	-10.462771000000
H	-7.821703000000	-12.305321000000	-3.067681000000
H	-3.742131000000	-15.636546000000	-6.329168000000
H	-5.510706000000	-9.940211000000	-6.492858000000

^{2}TS7$_{oB}$

C	6.776193000000	-1.137504000000	-3.189139000000
C	7.714055000000	-2.153426000000	-2.551591000000
O	7.822183000000	-3.304556000000	-2.974714000000
C	5.319608000000	-1.626863000000	-3.154823000000
H	6.864265000000	-0.157547000000	-2.711894000000
H	5.254746000000	-2.565184000000	-3.716484000000
H	4.663048000000	-0.897493000000	-3.634059000000
N	8.413916000000	-1.710441000000	-1.465823000000
C	9.351778000000	-2.561559000000	-0.756606000000
C	8.770395000000	-3.652409000000	0.160617000000
O	9.541412000000	-4.402825000000	0.747622000000
H	8.203645000000	-0.792883000000	-1.097684000000
H	9.970491000000	-3.091882000000	-1.486614000000
N	7.414322000000	-3.725874000000	0.238187000000
C	6.743176000000	-4.845573000000	0.867811000000
C	6.127994000000	-5.882988000000	-0.087624000000
O	5.399161000000	-6.758148000000	0.373179000000
H	6.841879000000	-3.062173000000	-0.275171000000
H	5.937861000000	-4.491906000000	1.518353000000
H	7.479607000000	-5.361341000000	1.489786000000
N	6.458364000000	-5.766533000000	-1.399173000000
C	5.951833000000	-6.682641000000	-2.403060000000
H	7.042212000000	-4.996513000000	-1.707435000000
H	5.410236000000	-6.140131000000	-3.186555000000
H	6.767286000000	-7.248661000000	-2.870152000000
H	5.272560000000	-7.377324000000	-1.906462000000
H	10.017297000000	-1.935870000000	-0.155671000000
H	7.082986000000	-1.025238000000	-4.237814000000
S	4.685613000000	-2.037950000000	-1.479344000000
Fe	3.556363000000	-0.258189000000	-0.436719000000
C	6.475221000000	0.224517000000	1.279841000000
C	4.472896000000	2.146980000000	-2.693879000000
C	0.770022000000	-0.931535000000	-2.260732000000
C	2.512976000000	-2.461879000000	1.981361000000
N	5.166494000000	0.940814000000	-0.675467000000
C	6.282424000000	0.984003000000	0.126690000000
C	7.216350000000	1.963229000000	-0.374709000000
C	6.642832000000	2.522341000000	-1.481745000000
C	5.366275000000	1.876323000000	-1.657135000000
N	2.795852000000	0.451574000000	-2.145330000000
C	3.278443000000	1.477874000000	-2.920588000000
C	2.384042000000	1.735124000000	-4.028117000000
C	1.361562000000	0.844777000000	-3.924117000000
C	1.628482000000	0.054872000000	-2.739829000000
N	1.947458000000	-1.437522000000	-0.169996000000
C	0.906437000000	-1.600187000000	-1.047349000000
C	-0.062585000000	-2.532772000000	-0.511954000000
C	0.403643000000	-2.922407000000	0.705159000000
C	1.669724000000	-2.254599000000	0.898286000000
N	4.340647000000	-0.988922000000	1.285814000000
C	3.773795000000	-1.890348000000	2.145578000000
C	4.667582000000	-2.173704000000	3.242908000000
C	5.789412000000	-1.430324000000	3.034754000000
C	5.574659000000	-0.687740000000	1.815445000000
H	7.406152000000	0.373040000000	1.820149000000
H	4.749007000000	2.926830000000	-3.397688000000

H	-0.103025000000	-1.148163000000	-2.871155000000
H	2.174543000000	-3.151124000000	2.748686000000
H	0.476439000000	0.725527000000	-4.537799000000
H	2.535849000000	2.501936000000	-4.777296000000
H	-0.977232000000	-2.848009000000	-0.997108000000
H	-0.059712000000	-3.588160000000	1.420954000000
H	7.040906000000	3.294930000000	-2.127415000000
H	8.171611000000	2.198784000000	0.078263000000
H	6.684962000000	-1.378369000000	3.640578000000
H	4.452962000000	-2.857014000000	4.054421000000
O	2.595488000000	1.096692000000	0.477874000000
C	3.329902000000	2.427330000000	2.064473000000
C	3.764155000000	7.231594000000	2.726654000000
C	3.029887000000	4.712442000000	1.288938000000
C	3.821088000000	3.583373000000	1.432355000000
C	2.002615000000	2.430795000000	2.700273000000
C	1.203634000000	3.630897000000	2.533799000000
C	1.701406000000	4.703279000000	1.856296000000
H	1.588988000000	6.107839000000	-0.604061000000
H	2.980909000000	6.863208000000	-1.404773000000
C	4.978699000000	5.930053000000	0.156421000000
C	2.665059000000	6.018230000000	-0.782684000000
C	3.480117000000	5.970061000000	0.535934000000
C	3.240277000000	7.207046000000	1.424171000000
C	2.549613000000	8.346853000000	0.987385000000
C	2.389557000000	9.464132000000	1.805704000000
C	2.922666000000	9.466335000000	3.097128000000
C	3.613177000000	8.339214000000	3.556848000000
H	2.121230000000	8.378941000000	-0.008596000000
H	1.851887000000	10.340278000000	1.457176000000
O	2.737555000000	10.585197000000	3.858940000000
H	4.028803000000	8.326180000000	4.563105000000
H	4.296448000000	6.363321000000	3.106966000000
O	1.595512000000	1.418539000000	3.324127000000
H	3.995696000000	1.617161000000	2.329660000000
H	0.212180000000	3.642741000000	2.974804000000
H	1.094482000000	5.597953000000	1.752155000000
H	2.060416000000	0.601769000000	1.124469000000
H	4.828899000000	3.559607000000	1.035810000000
H	3.149757000000	10.450042000000	4.726502000000
H	2.834044000000	5.098107000000	-1.350998000000
H	5.621110000000	5.834016000000	1.037817000000
H	5.199283000000	5.098970000000	-0.522327000000
H	5.248171000000	6.862335000000	-0.350103000000
C	-5.936271000000	-3.637536000000	7.315108000000
C	-4.953086000000	-3.378740000000	6.191709000000
O	-5.333146000000	-3.168580000000	5.034562000000
H	-6.455429000000	-2.701007000000	7.547708000000
H	-6.690516000000	-4.351696000000	6.974582000000
N	-3.629348000000	-3.402322000000	6.518530000000
C	-2.595237000000	-2.872916000000	5.619970000000
C	-2.322231000000	-3.755226000000	4.393011000000
O	-1.708057000000	-3.288904000000	3.431613000000
C	-1.298233000000	-2.620204000000	6.397185000000
H	-3.386788000000	-3.486361000000	7.497460000000
H	-2.950801000000	-1.929778000000	5.187160000000
H	-1.461627000000	-1.880331000000	7.187752000000

H	-0.912783000000	-3.543398000000	6.846311000000
H	-0.540647000000	-2.226528000000	5.716031000000
N	-2.782319000000	-5.031604000000	4.402586000000
C	-2.752464000000	-5.836332000000	3.193018000000
C	-3.809339000000	-5.468606000000	2.135881000000
O	-3.694105000000	-5.921817000000	0.995417000000
H	-3.279811000000	-5.357794000000	5.219068000000
H	-2.891849000000	-6.888421000000	3.458431000000
H	-1.778037000000	-5.740726000000	2.711094000000
N	-4.843412000000	-4.701351000000	2.553919000000
C	-5.956072000000	-4.367915000000	1.682700000000
C	-6.116859000000	-2.892910000000	1.284399000000
O	-6.539167000000	-2.614839000000	0.160888000000
H	-4.858242000000	-4.355172000000	3.508819000000
H	-5.844604000000	-4.941527000000	0.763578000000
N	-5.869909000000	-1.946110000000	2.223384000000
C	-6.305332000000	-0.569142000000	2.002531000000
C	-5.593022000000	0.148676000000	0.845632000000
O	-6.229795000000	0.654298000000	-0.077684000000
H	-5.552072000000	-2.208776000000	3.150105000000
H	-7.371600000000	-0.539818000000	1.762668000000
N	-4.235225000000	0.203101000000	0.927031000000
C	-3.486671000000	0.806452000000	-0.170287000000
C	-3.478370000000	-0.073414000000	-1.432302000000
O	-3.353340000000	0.463200000000	-2.543681000000
C	-2.095687000000	1.235464000000	0.340065000000
O	-2.297520000000	2.204650000000	1.390625000000
C	-1.208335000000	1.887855000000	-0.707064000000
H	-3.731796000000	-0.222654000000	1.724163000000
H	-4.014227000000	1.712140000000	-0.488262000000
H	-1.591660000000	0.355733000000	0.767125000000
H	-3.161565000000	2.024953000000	1.800868000000
H	-0.261007000000	2.184035000000	-0.245772000000
H	-1.696535000000	2.774800000000	-1.122088000000
H	-0.987403000000	1.198587000000	-1.523846000000
N	-3.694740000000	-1.392728000000	-1.288068000000
C	-3.858427000000	-2.285412000000	-2.433295000000
C	-5.059453000000	-1.865662000000	-3.313077000000
O	-4.988106000000	-1.912127000000	-4.542958000000
C	-4.031583000000	-3.736693000000	-1.922135000000
O	-3.116497000000	-3.884351000000	-0.838505000000
C	-3.777659000000	-4.753652000000	-3.032295000000
H	-3.674801000000	-1.824940000000	-0.372980000000
H	-2.980390000000	-2.216720000000	-3.083706000000
H	-5.059340000000	-3.842240000000	-1.547188000000
H	-3.302129000000	-4.722456000000	-0.367299000000
H	-3.963654000000	-5.768621000000	-2.663897000000
H	-4.429563000000	-4.572358000000	-3.893210000000
H	-2.736309000000	-4.694190000000	-3.366358000000
N	-6.167257000000	-1.448260000000	-2.652960000000
C	-7.328371000000	-0.936563000000	-3.362934000000
C	-7.199712000000	0.492052000000	-3.933243000000
O	-8.116366000000	0.967322000000	-4.588376000000
H	-6.199059000000	-1.480461000000	-1.638184000000
H	-7.560026000000	-1.585235000000	-4.212433000000
N	-6.021019000000	1.122155000000	-3.668742000000
C	-5.631515000000	2.405820000000	-4.225184000000

C	-4.475160000000	2.260551000000	-5.249671000000
O	-3.847375000000	3.241230000000	-5.641993000000
C	-5.225278000000	3.392332000000	-3.078060000000
O	-6.088643000000	3.262932000000	-1.952877000000
C	-5.298603000000	4.851623000000	-3.509188000000
H	-5.307639000000	0.629566000000	-3.146583000000
H	-6.511786000000	2.797339000000	-4.745606000000
H	-4.191548000000	3.145809000000	-2.788525000000
H	-6.155068000000	2.326766000000	-1.696070000000
H	-4.997596000000	5.496604000000	-2.676592000000
H	-6.330486000000	5.105517000000	-3.778917000000
H	-4.646421000000	5.026274000000	-4.365626000000
N	-4.195067000000	0.991534000000	-5.660889000000
C	-3.087887000000	0.676827000000	-6.551081000000
C	-2.269853000000	-0.519488000000	-6.037372000000
O	-1.660072000000	-0.236071000000	-4.773844000000
C	-1.153473000000	-0.883793000000	-7.008782000000
H	-4.773011000000	0.222521000000	-5.342167000000
H	-2.462616000000	1.571265000000	-6.615341000000
H	-2.947739000000	-1.377844000000	-5.923948000000
H	-2.348906000000	0.081278000000	-4.157146000000
H	-0.559886000000	-1.711714000000	-6.608034000000
H	-1.563804000000	-1.190440000000	-7.977195000000
H	-0.485375000000	-0.029405000000	-7.170241000000
C	-3.942442000000	4.402917000000	4.521519000000
C	-4.558300000000	5.260658000000	3.407961000000
C	-5.053801000000	4.483289000000	2.172350000000
C	-6.135950000000	3.448045000000	2.520383000000
C	-5.565111000000	5.439216000000	1.083775000000
H	-3.128971000000	3.764586000000	4.151776000000
H	-5.395536000000	5.843541000000	3.821084000000
H	-3.814375000000	5.997172000000	3.073432000000
H	-4.186115000000	3.948990000000	1.755810000000
H	-4.803818000000	6.185913000000	0.825602000000
H	-5.829762000000	4.893702000000	0.171009000000
H	-6.456989000000	5.979586000000	1.428556000000
H	-5.772643000000	2.692995000000	3.229839000000
H	-7.006546000000	3.937055000000	2.980850000000
H	-6.484664000000	2.931399000000	1.620206000000
O	-2.778052000000	-0.678591000000	3.204802000000
H	-2.237238000000	-1.485071000000	3.089448000000
H	-2.145298000000	0.029244000000	3.510120000000
O	-1.167365000000	1.428453000000	3.793654000000
H	-0.186012000000	1.350467000000	3.770561000000
H	-1.394610000000	1.841431000000	2.933040000000
H	-3.460416000000	0.450377000000	-7.561034000000
H	-4.685569000000	3.747337000000	4.990460000000
H	-5.466248000000	-4.012719000000	8.228804000000
H	-3.524975000000	5.035353000000	5.314075000000
H	-6.896632000000	-4.675662000000	2.159682000000
H	-8.183142000000	-0.949577000000	-2.682855000000
H	-6.131078000000	-0.018064000000	2.931060000000

^{4}TS7$_{oB}$

C	6.776193000000	-1.137504000000	-3.189139000000
C	7.714055000000	-2.153426000000	-2.551591000000
O	7.822183000000	-3.304556000000	-2.974714000000
C	5.319608000000	-1.626863000000	-3.154823000000
H	6.864265000000	-0.157547000000	-2.711894000000
H	5.254746000000	-2.565184000000	-3.716484000000
H	4.663048000000	-0.897493000000	-3.634059000000
N	8.413916000000	-1.710441000000	-1.465823000000
C	9.351778000000	-2.561559000000	-0.756606000000
C	8.770395000000	-3.652409000000	0.160617000000
O	9.541412000000	-4.402825000000	0.747622000000
H	8.203645000000	-0.792883000000	-1.097684000000
H	9.970491000000	-3.091882000000	-1.486614000000
N	7.414322000000	-3.725874000000	0.238187000000
C	6.743176000000	-4.845573000000	0.867811000000
C	6.127994000000	-5.882988000000	-0.087624000000
O	5.399161000000	-6.758148000000	0.373179000000
H	6.841879000000	-3.062173000000	-0.275171000000
H	5.937861000000	-4.491906000000	1.518353000000
H	7.479607000000	-5.361341000000	1.489786000000
N	6.458364000000	-5.766533000000	-1.399173000000
C	5.951833000000	-6.682641000000	-2.403060000000
H	7.042212000000	-4.996513000000	-1.707435000000
H	5.410236000000	-6.140131000000	-3.186555000000
H	6.767286000000	-7.248661000000	-2.870152000000
H	5.272560000000	-7.377324000000	-1.906462000000
H	10.017297000000	-1.935870000000	-0.155671000000
H	7.082986000000	-1.025238000000	-4.237814000000
S	4.685613000000	-2.037950000000	-1.479344000000
Fe	3.556363000000	-0.258189000000	-0.436719000000
C	6.475221000000	0.224517000000	1.279841000000
C	4.472896000000	2.146980000000	-2.693879000000
C	0.770022000000	-0.931535000000	-2.260732000000
C	2.512976000000	-2.461879000000	1.981361000000
N	5.166494000000	0.940814000000	-0.675467000000
C	6.282424000000	0.984003000000	0.126690000000
C	7.216350000000	1.963229000000	-0.374709000000
C	6.642832000000	2.522341000000	-1.481745000000
C	5.366275000000	1.876323000000	-1.657135000000
N	2.795852000000	0.451574000000	-2.145330000000
C	3.278443000000	1.477874000000	-2.920588000000
C	2.384042000000	1.735124000000	-4.028117000000
C	1.361562000000	0.844777000000	-3.924117000000
C	1.628482000000	0.054872000000	-2.739829000000
N	1.947458000000	-1.437522000000	-0.169996000000
C	0.906437000000	-1.600187000000	-1.047349000000
C	-0.062585000000	-2.532772000000	-0.511954000000
C	0.403643000000	-2.922407000000	0.705159000000
C	1.669724000000	-2.254599000000	0.898286000000
N	4.340647000000	-0.988922000000	1.285814000000
C	3.773795000000	-1.890348000000	2.145578000000
C	4.667582000000	-2.173704000000	3.242908000000
C	5.789412000000	-1.430324000000	3.034754000000
C	5.574659000000	-0.687740000000	1.815445000000
H	7.406152000000	0.373040000000	1.820149000000
H	4.749007000000	2.926830000000	-3.397688000000

H	-0.103025000000	-1.148163000000	-2.871155000000
H	2.174543000000	-3.151124000000	2.748686000000
H	0.476439000000	0.725527000000	-4.537799000000
H	2.535849000000	2.501936000000	-4.777296000000
H	-0.977232000000	-2.848009000000	-0.997108000000
H	-0.059712000000	-3.588160000000	1.420954000000
H	7.040906000000	3.294930000000	-2.127415000000
H	8.171611000000	2.198784000000	0.078263000000
H	6.684962000000	-1.378369000000	3.640578000000
H	4.452962000000	-2.857014000000	4.054421000000
O	2.595488000000	1.096692000000	0.477874000000
C	3.329902000000	2.427330000000	2.064473000000
C	3.764155000000	7.231594000000	2.726654000000
C	3.029887000000	4.712442000000	1.288938000000
C	3.821088000000	3.583373000000	1.432355000000
C	2.002615000000	2.430795000000	2.700273000000
C	1.203634000000	3.630897000000	2.533799000000
C	1.701406000000	4.703279000000	1.856296000000
H	1.588988000000	6.107839000000	-0.604061000000
H	2.980909000000	6.863208000000	-1.404773000000
C	4.978699000000	5.930053000000	0.156421000000
C	2.665059000000	6.018230000000	-0.782684000000
C	3.480117000000	5.970061000000	0.535934000000
C	3.240277000000	7.207046000000	1.424171000000
C	2.549613000000	8.346853000000	0.987385000000
C	2.389557000000	9.464132000000	1.805704000000
C	2.922666000000	9.466335000000	3.097128000000
C	3.613177000000	8.339214000000	3.556848000000
H	2.121230000000	8.378941000000	-0.008596000000
H	1.851887000000	10.340278000000	1.457176000000
O	2.737555000000	10.585197000000	3.858940000000
H	4.028803000000	8.326180000000	4.563105000000
H	4.296448000000	6.363321000000	3.106966000000
O	1.595512000000	1.418539000000	3.324127000000
H	3.995696000000	1.617161000000	2.329660000000
H	0.212180000000	3.642741000000	2.974804000000
H	1.094482000000	5.597953000000	1.752155000000
H	2.060416000000	0.601769000000	1.124469000000
H	4.828899000000	3.559607000000	1.035810000000
H	3.149757000000	10.450042000000	4.726502000000
H	2.834044000000	5.098107000000	-1.350998000000
H	5.621110000000	5.834016000000	1.037817000000
H	5.199283000000	5.098970000000	-0.522327000000
H	5.248171000000	6.862335000000	-0.350103000000
C	-5.936271000000	-3.637536000000	7.315108000000
C	-4.953086000000	-3.378740000000	6.191709000000
O	-5.333146000000	-3.168580000000	5.034562000000
H	-6.455429000000	-2.701007000000	7.547708000000
H	-6.690516000000	-4.351696000000	6.974582000000
N	-3.629348000000	-3.402322000000	6.518530000000
C	-2.595237000000	-2.872916000000	5.619970000000
C	-2.322231000000	-3.755226000000	4.393011000000
O	-1.708057000000	-3.288904000000	3.431613000000
C	-1.298233000000	-2.620204000000	6.397185000000
H	-3.386788000000	-3.486361000000	7.497460000000
H	-2.950801000000	-1.929778000000	5.187160000000
H	-1.461627000000	-1.880331000000	7.187752000000

H	-0.912783000000	-3.543398000000	6.846311000000
H	-0.540647000000	-2.226528000000	5.716031000000
N	-2.782319000000	-5.031604000000	4.402586000000
C	-2.752464000000	-5.836332000000	3.193018000000
C	-3.809339000000	-5.468606000000	2.135881000000
O	-3.694105000000	-5.921817000000	0.995417000000
H	-3.279811000000	-5.357794000000	5.219068000000
H	-2.891849000000	-6.888421000000	3.458431000000
H	-1.778037000000	-5.740726000000	2.711094000000
N	-4.843412000000	-4.701351000000	2.553919000000
C	-5.956072000000	-4.367915000000	1.682700000000
C	-6.116859000000	-2.892910000000	1.284399000000
O	-6.539167000000	-2.614839000000	0.160888000000
H	-4.858242000000	-4.355172000000	3.508819000000
H	-5.844604000000	-4.941527000000	0.763578000000
N	-5.869909000000	-1.946110000000	2.223384000000
C	-6.305332000000	-0.569142000000	2.002531000000
C	-5.593022000000	0.148676000000	0.845632000000
O	-6.229795000000	0.654298000000	-0.077684000000
H	-5.552072000000	-2.208776000000	3.150105000000
H	-7.371600000000	-0.539818000000	1.762668000000
N	-4.235225000000	0.203101000000	0.927031000000
C	-3.486671000000	0.806452000000	-0.170287000000
C	-3.478370000000	-0.073414000000	-1.432302000000
O	-3.353340000000	0.463200000000	-2.543681000000
C	-2.095687000000	1.235464000000	0.340065000000
O	-2.297520000000	2.204650000000	1.390625000000
C	-1.208335000000	1.887855000000	-0.707064000000
H	-3.731796000000	-0.222654000000	1.724163000000
H	-4.014227000000	1.712140000000	-0.488262000000
H	-1.591660000000	0.355733000000	0.767125000000
H	-3.161565000000	2.024953000000	1.800868000000
H	-0.261007000000	2.184035000000	-0.245772000000
H	-1.696535000000	2.774800000000	-1.122088000000
H	-0.987403000000	1.198587000000	-1.523846000000
N	-3.694740000000	-1.392728000000	-1.288068000000
C	-3.858427000000	-2.285412000000	-2.433295000000
C	-5.059453000000	-1.865662000000	-3.313077000000
O	-4.988106000000	-1.912127000000	-4.542958000000
C	-4.031583000000	-3.736693000000	-1.922135000000
O	-3.116497000000	-3.884351000000	-0.838505000000
C	-3.777659000000	-4.753652000000	-3.032295000000
H	-3.674801000000	-1.824940000000	-0.372980000000
H	-2.980390000000	-2.216720000000	-3.083706000000
H	-5.059340000000	-3.842240000000	-1.547188000000
H	-3.302129000000	-4.722456000000	-0.367299000000
H	-3.963654000000	-5.768621000000	-2.663897000000
H	-4.429563000000	-4.572358000000	-3.893210000000
H	-2.736309000000	-4.694190000000	-3.366358000000
N	-6.167257000000	-1.448260000000	-2.652960000000
C	-7.328371000000	-0.936563000000	-3.362934000000
C	-7.199712000000	0.492052000000	-3.933243000000
O	-8.116366000000	0.967322000000	-4.588376000000
H	-6.199059000000	-1.480461000000	-1.638184000000
H	-7.560026000000	-1.585235000000	-4.212433000000
N	-6.021019000000	1.122155000000	-3.668742000000
C	-5.631515000000	2.405820000000	-4.225184000000

C	-4.475160000000	2.260551000000	-5.249671000000
O	-3.847375000000	3.241230000000	-5.641993000000
C	-5.225278000000	3.392332000000	-3.078060000000
O	-6.088643000000	3.262932000000	-1.952877000000
C	-5.298603000000	4.851623000000	-3.509188000000
H	-5.307639000000	0.629566000000	-3.146583000000
H	-6.511786000000	2.797339000000	-4.745606000000
H	-4.191548000000	3.145809000000	-2.788525000000
H	-6.155068000000	2.326766000000	-1.696070000000
H	-4.997596000000	5.496604000000	-2.676592000000
H	-6.330486000000	5.105517000000	-3.778917000000
H	-4.646421000000	5.026274000000	-4.365626000000
N	-4.195067000000	0.991534000000	-5.660889000000
C	-3.087887000000	0.676827000000	-6.551081000000
C	-2.269853000000	-0.519488000000	-6.037372000000
O	-1.660072000000	-0.236071000000	-4.773844000000
C	-1.153473000000	-0.883793000000	-7.008782000000
H	-4.773011000000	0.222521000000	-5.342167000000
H	-2.462616000000	1.571265000000	-6.615341000000
H	-2.947739000000	-1.377844000000	-5.923948000000
H	-2.348906000000	0.081278000000	-4.157146000000
H	-0.559886000000	-1.711714000000	-6.608034000000
H	-1.563804000000	-1.190440000000	-7.977195000000
H	-0.485375000000	-0.029405000000	-7.170241000000
C	-3.942442000000	4.402917000000	4.521519000000
C	-4.558300000000	5.260658000000	3.407961000000
C	-5.053801000000	4.483289000000	2.172350000000
C	-6.135950000000	3.448045000000	2.520383000000
C	-5.565111000000	5.439216000000	1.083775000000
H	-3.128971000000	3.764586000000	4.151776000000
H	-5.395536000000	5.843541000000	3.821084000000
H	-3.814375000000	5.997172000000	3.073432000000
H	-4.186115000000	3.948990000000	1.755810000000
H	-4.803818000000	6.185913000000	0.825602000000
H	-5.829762000000	4.893702000000	0.171009000000
H	-6.456989000000	5.979586000000	1.428556000000
H	-5.772643000000	2.692995000000	3.229839000000
H	-7.006546000000	3.937055000000	2.980850000000
H	-6.484664000000	2.931399000000	1.620206000000
O	-2.778052000000	-0.678591000000	3.204802000000
H	-2.237238000000	-1.485071000000	3.089448000000
H	-2.145298000000	0.029244000000	3.510120000000
O	-1.167365000000	1.428453000000	3.793654000000
H	-0.186012000000	1.350467000000	3.770561000000
H	-1.394610000000	1.841431000000	2.933040000000
H	-3.460416000000	0.450377000000	-7.561034000000
H	-4.685569000000	3.747337000000	4.990460000000
H	-5.466248000000	-4.012719000000	8.228804000000
H	-3.524975000000	5.035353000000	5.314075000000
H	-6.896632000000	-4.675662000000	2.159682000000
H	-8.183142000000	-0.949577000000	-2.682855000000
H	-6.131078000000	-0.018064000000	2.931060000000

^{2}I7$_{oB}$

C	2.465984000000	-2.688155000000	-0.744140000000
C	2.384569000000	-1.550678000000	0.264744000000
O	2.589470000000	-1.721681000000	1.467050000000
C	1.448728000000	-3.793546000000	-0.423318000000
H	2.331666000000	-2.325753000000	-1.766355000000
H	1.669771000000	-4.201390000000	0.568668000000
H	1.527879000000	-4.606292000000	-1.148582000000
N	2.050800000000	-0.329452000000	-0.246820000000
C	1.946791000000	0.850646000000	0.591714000000
C	0.718672000000	0.964300000000	1.511941000000
O	0.640603000000	1.916204000000	2.280025000000
H	1.770242000000	-0.271869000000	-1.216345000000
H	2.814630000000	0.892780000000	1.256300000000
N	-0.205302000000	-0.030074000000	1.421871000000
C	-1.285032000000	-0.161745000000	2.379070000000
C	-1.119318000000	-1.274970000000	3.428221000000
O	-2.074998000000	-1.567487000000	4.142898000000
H	-0.074039000000	-0.781458000000	0.751430000000
H	-2.233011000000	-0.346528000000	1.865155000000
H	-1.367384000000	0.789748000000	2.911344000000
N	0.106627000000	-1.852678000000	3.505619000000
C	0.399313000000	-2.909440000000	4.454814000000
H	0.838570000000	-1.575044000000	2.860339000000
H	0.739475000000	-3.814136000000	3.937560000000
H	1.176615000000	-2.599766000000	5.164171000000
H	-0.519096000000	-3.128118000000	5.001897000000
H	1.975798000000	1.740670000000	-0.042982000000
H	3.476337000000	-3.112728000000	-0.671726000000
S	-0.297700000000	-3.224473000000	-0.313067000000
Fe	-1.345375000000	-3.320664000000	-2.294201000000
C	-0.544779000000	-0.082711000000	-2.995096000000
C	1.286749000000	-4.401765000000	-4.216594000000
C	-2.177445000000	-6.560569000000	-1.607820000000
C	-4.177968000000	-2.256458000000	-0.639891000000
N	0.087015000000	-2.429855000000	-3.378081000000
C	0.272089000000	-1.075841000000	-3.526919000000
C	1.427193000000	-0.819164000000	-4.354959000000
C	1.929131000000	-2.033326000000	-4.717333000000
C	1.088179000000	-3.031023000000	-4.103974000000
N	-0.605761000000	-5.112416000000	-2.826622000000
C	0.503487000000	-5.370000000000	-3.604045000000
C	0.752092000000	-6.789420000000	-3.669912000000
C	-0.207267000000	-7.389532000000	-2.914523000000
C	-1.054744000000	-6.341987000000	-2.396584000000
N	-2.911732000000	-4.221837000000	-1.384956000000
C	-3.030803000000	-5.569250000000	-1.140205000000
C	-4.182169000000	-5.829004000000	-0.307324000000
C	-4.753094000000	-4.622160000000	-0.042443000000
C	-3.950016000000	-3.624733000000	-0.708111000000
N	-2.220397000000	-1.533848000000	-1.934771000000
C	-3.360754000000	-1.285096000000	-1.206686000000
C	-3.576270000000	0.136155000000	-1.085022000000
C	-2.544176000000	0.747364000000	-1.732514000000
C	-1.701494000000	-0.300847000000	-2.255500000000
H	-0.270409000000	0.948952000000	-3.196890000000
H	2.127584000000	-4.741153000000	-4.813454000000

H	-2.421576000000	-7.586022000000	-1.345019000000
H	-5.035934000000	-1.917967000000	-0.067203000000
H	-0.315464000000	-8.448916000000	-2.731792000000
H	1.551607000000	-7.272067000000	-4.215994000000
H	-4.484641000000	-6.813381000000	0.027895000000
H	-5.622233000000	-4.402554000000	0.565042000000
H	2.785588000000	-2.249110000000	-5.343409000000
H	1.783553000000	0.165790000000	-4.630446000000
H	-2.352476000000	1.807270000000	-1.842795000000
H	-4.402289000000	0.592414000000	-0.554153000000
O	-2.469657000000	-3.516224000000	-4.258374000000
C	-3.798019000000	-3.052696000000	-4.453168000000
C	-6.747849000000	0.698166000000	-4.464178000000
C	-4.851141000000	-1.230444000000	-5.767246000000
C	-3.838552000000	-1.651863000000	-4.981315000000
C	-4.608305000000	-4.069124000000	-5.245578000000
C	-5.752170000000	-3.551533000000	-5.973503000000
C	-5.842747000000	-2.216560000000	-6.207890000000
H	-5.605854000000	-0.421559000000	-8.313591000000
H	-4.937798000000	1.215918000000	-8.217841000000
C	-3.941598000000	1.155847000000	-5.698500000000
C	-4.859178000000	0.202122000000	-7.810821000000
C	-5.022642000000	0.211637000000	-6.271656000000
C	-6.410164000000	0.719349000000	-5.827297000000
C	-7.363924000000	1.235201000000	-6.717093000000
C	-8.596319000000	1.714834000000	-6.274524000000
C	-8.906722000000	1.689780000000	-4.913010000000
C	-7.973101000000	1.175557000000	-4.005899000000
H	-7.154621000000	1.275536000000	-7.780714000000
H	-9.324722000000	2.114472000000	-6.973096000000
O	-10.126727000000	2.171119000000	-4.529650000000
H	-8.204066000000	1.148458000000	-2.942120000000
H	-6.040584000000	0.300240000000	-3.740839000000
O	-4.278000000000	-5.260172000000	-5.194533000000
H	-4.314881000000	-3.024947000000	-3.471020000000
H	-6.444778000000	-4.262667000000	-6.413553000000
H	-6.680681000000	-1.848787000000	-6.794860000000
H	-2.499618000000	-4.491963000000	-4.347260000000
H	-3.060814000000	-0.990412000000	-4.618656000000
H	-10.196087000000	2.126057000000	-3.563202000000
H	-3.870043000000	-0.187790000000	-8.071588000000
H	-3.957584000000	1.186191000000	-4.604897000000
H	-2.938491000000	0.851185000000	-6.018446000000
H	-4.117179000000	2.172700000000	-6.063817000000
C	-12.072284000000	-10.649090000000	-2.439486000000
C	-10.724929000000	-9.968146000000	-2.565007000000
O	-9.667164000000	-10.595795000000	-2.446114000000
H	-12.234784000000	-11.270163000000	-3.327538000000
H	-12.057266000000	-11.317031000000	-1.574373000000
N	-10.736196000000	-8.625230000000	-2.802952000000
C	-9.529098000000	-7.905264000000	-3.225645000000
C	-8.453518000000	-7.823681000000	-2.134274000000
O	-7.278023000000	-7.617705000000	-2.438990000000
C	-9.895702000000	-6.498946000000	-3.714751000000
H	-11.626969000000	-8.186441000000	-2.998575000000
H	-9.043932000000	-8.456211000000	-4.040304000000
H	-10.571210000000	-6.554834000000	-4.575197000000

H	-10.374500000000	-5.909996000000	-2.923717000000
H	-8.987718000000	-5.979866000000	-4.030175000000
N	-8.827129000000	-7.974409000000	-0.839106000000
C	-7.803821000000	-8.044201000000	0.191255000000
C	-6.914629000000	-9.299572000000	0.133748000000
O	-5.819785000000	-9.280048000000	0.700627000000
H	-9.797114000000	-8.153851000000	-0.622292000000
H	-8.286454000000	-8.009937000000	1.172538000000
H	-7.134513000000	-7.185523000000	0.109926000000
N	-7.436519000000	-10.383070000000	-0.488994000000
C	-6.752991000000	-11.663251000000	-0.525308000000
C	-6.207440000000	-12.148066000000	-1.877855000000
O	-5.246635000000	-12.917850000000	-1.884993000000
H	-8.333991000000	-10.303546000000	-0.957472000000
H	-5.911061000000	-11.615044000000	0.164351000000
N	-6.855672000000	-11.776087000000	-3.009048000000
C	-6.575546000000	-12.457859000000	-4.272378000000
C	-5.156873000000	-12.224523000000	-4.819481000000
O	-4.387021000000	-13.162236000000	-5.015317000000
H	-7.683937000000	-11.193836000000	-2.955496000000
H	-6.678911000000	-13.539845000000	-4.153909000000
N	-4.830274000000	-10.927599000000	-5.074470000000
C	-3.470381000000	-10.608500000000	-5.496675000000
C	-2.432823000000	-10.865372000000	-4.388307000000
O	-1.280925000000	-11.197325000000	-4.700475000000
C	-3.438345000000	-9.177392000000	-6.068826000000
O	-4.293595000000	-9.134215000000	-7.232041000000
C	-2.059708000000	-8.704920000000	-6.504417000000
H	-5.516409000000	-10.174436000000	-4.894759000000
H	-3.178882000000	-11.298503000000	-6.296131000000
H	-3.844813000000	-8.488305000000	-5.312884000000
H	-4.989303000000	-9.804462000000	-7.116135000000
H	-2.159551000000	-7.738336000000	-7.008462000000
H	-1.604987000000	-9.416440000000	-7.199595000000
H	-1.392751000000	-8.578123000000	-5.647754000000
N	-2.841564000000	-10.778920000000	-3.109489000000
C	-1.967458000000	-11.104298000000	-1.986309000000
C	-1.432695000000	-12.553749000000	-2.072284000000
O	-0.263242000000	-12.810061000000	-1.781111000000
C	-2.745378000000	-10.878382000000	-0.665686000000
O	-3.522529000000	-9.694173000000	-0.857690000000
C	-1.800494000000	-10.760039000000	0.527040000000
H	-3.743569000000	-10.382299000000	-2.874489000000
H	-1.075668000000	-10.469902000000	-2.011279000000
H	-3.420613000000	-11.733554000000	-0.525098000000
H	-4.201509000000	-9.626240000000	-0.155719000000
H	-2.372249000000	-10.669034000000	1.457194000000
H	-1.150404000000	-11.637881000000	0.603170000000
H	-1.170121000000	-9.870502000000	0.422928000000
N	-2.320543000000	-13.493864000000	-2.477899000000
C	-1.930999000000	-14.881348000000	-2.670039000000
C	-1.167958000000	-15.202395000000	-3.972731000000
O	-0.798815000000	-16.348352000000	-4.189222000000
H	-3.285046000000	-13.237144000000	-2.666230000000
H	-1.282764000000	-15.198191000000	-1.848161000000
N	-0.935273000000	-14.138472000000	-4.789496000000
C	-0.124031000000	-14.188281000000	-5.993237000000

C	1.203981000000	-13.406329000000	-5.823031000000
O	1.920518000000	-13.155420000000	-6.789307000000
C	-0.931004000000	-13.630613000000	-7.215511000000
O	-2.288466000000	-14.048475000000	-7.180184000000
C	-0.371393000000	-14.107533000000	-8.550189000000
H	-1.262259000000	-13.222521000000	-4.507965000000
H	0.111832000000	-15.243613000000	-6.164418000000
H	-0.872995000000	-12.530994000000	-7.167041000000
H	-2.669435000000	-13.851066000000	-6.307405000000
H	-0.970302000000	-13.690419000000	-9.367095000000
H	-0.435640000000	-15.200590000000	-8.607595000000
H	0.669118000000	-13.799728000000	-8.659182000000
N	1.500903000000	-12.998949000000	-4.556345000000
C	2.654522000000	-12.169105000000	-4.244732000000
C	2.277555000000	-11.016187000000	-3.299528000000
O	1.316888000000	-10.143216000000	-3.891735000000
C	3.497059000000	-10.170963000000	-2.949639000000
H	0.905020000000	-13.284981000000	-3.788693000000
H	3.034811000000	-11.780128000000	-5.192867000000
H	1.860344000000	-11.450121000000	-2.377544000000
H	0.557952000000	-10.675235000000	-4.196750000000
H	3.204968000000	-9.342545000000	-2.296083000000
H	4.255275000000	-10.769433000000	-2.432545000000
H	3.944060000000	-9.750293000000	-3.858171000000
C	-5.469313000000	-5.323276000000	-9.953879000000
C	-4.101330000000	-5.755180000000	-10.497190000000
C	-3.920589000000	-7.274885000000	-10.690201000000
C	-4.903873000000	-7.856605000000	-11.717838000000
C	-2.472834000000	-7.594562000000	-11.092858000000
H	-5.695242000000	-5.814513000000	-8.999351000000
H	-3.912398000000	-5.251248000000	-11.457387000000
H	-3.319512000000	-5.398755000000	-9.811176000000
H	-4.113120000000	-7.763325000000	-9.722954000000
H	-1.758906000000	-7.219826000000	-10.349273000000
H	-2.315840000000	-8.675041000000	-11.191458000000
H	-2.222365000000	-7.132432000000	-12.057246000000
H	-5.947781000000	-7.714672000000	-11.416955000000
H	-4.770976000000	-7.380991000000	-12.699179000000
H	-4.741640000000	-8.933237000000	-11.847742000000
O	-6.804015000000	-8.909865000000	-4.857175000000
H	-6.780553000000	-8.437138000000	-3.999250000000
H	-6.614184000000	-8.196200000000	-5.524274000000
O	-5.985121000000	-6.999365000000	-6.615984000000
H	-5.388819000000	-6.416683000000	-6.095340000000
H	-5.368558000000	-7.606932000000	-7.081730000000
H	3.449224000000	-12.772584000000	-3.781608000000
H	-6.279645000000	-5.562240000000	-10.652087000000
H	-12.907624000000	-9.949270000000	-2.344440000000
H	-5.494985000000	-4.239533000000	-9.784620000000
H	-7.431067000000	-12.447127000000	-0.162603000000
H	-2.830984000000	-15.500131000000	-2.649972000000
H	-7.309712000000	-12.099544000000	-4.999378000000

$^{4}\mathbf{I7}_{OB}$

C	2.257291000000	-2.993253000000	0.123738000000
C	2.169794000000	-1.656036000000	0.846267000000
O	2.303142000000	-1.559793000000	2.067352000000
C	1.150950000000	-3.952844000000	0.590441000000
H	2.211630000000	-2.864910000000	-0.962111000000
H	1.273976000000	-4.144611000000	1.660874000000
H	1.240023000000	-4.906377000000	0.062511000000
N	1.911443000000	-0.570806000000	0.059510000000
C	1.784929000000	0.765489000000	0.610406000000
C	0.499915000000	1.098676000000	1.390322000000
O	0.386266000000	2.212601000000	1.890632000000
H	1.704464000000	-0.713489000000	-0.919563000000
H	2.605348000000	0.941254000000	1.312732000000
N	-0.427661000000	0.109710000000	1.495483000000
C	-1.547484000000	0.208331000000	2.409941000000
C	-1.400315000000	-0.550083000000	3.741676000000
O	-2.363981000000	-0.607994000000	4.501903000000
H	-0.268954000000	-0.787544000000	1.043307000000
H	-2.460813000000	-0.160863000000	1.933462000000
H	-1.690491000000	1.265571000000	2.647549000000
N	-0.184991000000	-1.098345000000	3.994236000000
C	0.084410000000	-1.838535000000	5.211620000000
H	0.554444000000	-1.037228000000	3.301908000000
H	0.400456000000	-2.863195000000	4.983015000000
H	0.872278000000	-1.354677000000	5.802032000000
H	-0.836880000000	-1.864900000000	5.795847000000
H	1.884276000000	1.492451000000	-0.200139000000
H	3.233732000000	-3.432706000000	0.368264000000
S	-0.556969000000	-3.290959000000	0.387995000000
Fe	-1.389303000000	-3.556756000000	-1.908157000000
C	-2.085908000000	-0.197329000000	-1.921262000000
C	1.446890000000	-2.967676000000	-3.747994000000
C	-0.802683000000	-6.929770000000	-2.094225000000
C	-4.513720000000	-4.190080000000	-0.629233000000
N	-0.520516000000	-1.919170000000	-2.714927000000
C	-0.919134000000	-0.610351000000	-2.549554000000
C	0.047288000000	0.281431000000	-3.134755000000
C	1.034531000000	-0.499166000000	-3.666225000000
C	0.674927000000	-1.867241000000	-3.394857000000
N	0.037935000000	-4.698255000000	-2.715555000000
C	1.138978000000	-4.282454000000	-3.435307000000
C	1.933271000000	-5.416640000000	-3.831230000000
C	1.310472000000	-6.529415000000	-3.352089000000
C	0.128022000000	-6.072121000000	-2.666793000000
N	-2.474064000000	-5.223744000000	-1.526967000000
C	-2.023390000000	-6.526532000000	-1.572345000000
C	-3.019335000000	-7.421466000000	-1.035130000000
C	-4.078037000000	-6.649744000000	-0.661869000000
C	-3.723227000000	-5.282593000000	-0.946258000000
N	-2.985212000000	-2.429317000000	-1.411710000000
C	-4.157454000000	-2.863025000000	-0.831338000000
C	-4.971624000000	-1.739346000000	-0.455300000000
C	-4.281816000000	-0.614989000000	-0.802917000000
C	-3.048591000000	-1.050327000000	-1.401368000000
H	-2.265138000000	0.870123000000	-1.842897000000
H	2.360551000000	-2.784382000000	-4.304787000000

H	-0.561573000000	-7.988395000000	-2.102102000000
H	-5.473855000000	-4.385272000000	-0.162763000000
H	1.566517000000	-7.575953000000	-3.467712000000
H	2.842284000000	-5.353954000000	-4.416166000000
H	-2.920574000000	-8.496351000000	-0.945130000000
H	-5.022025000000	-6.968973000000	-0.238591000000
H	1.925409000000	-0.189022000000	-4.197961000000
H	-0.034901000000	1.361116000000	-3.141888000000
H	-4.571610000000	0.419926000000	-0.672924000000
H	-5.940289000000	-1.813396000000	0.022069000000
O	-2.628680000000	-3.984143000000	-4.343660000000
C	-3.523470000000	-3.010061000000	-4.839933000000
C	-4.134075000000	1.159501000000	-7.112047000000
C	-3.694631000000	-1.784778000000	-6.997271000000
C	-2.931235000000	-2.256881000000	-5.990222000000
C	-4.902430000000	-3.608017000000	-5.090083000000
C	-5.707740000000	-2.978741000000	-6.120607000000
C	-5.119749000000	-2.132976000000	-7.006539000000
H	-4.321550000000	-2.075392000000	-9.685475000000
H	-2.874612000000	-1.274233000000	-10.320822000000
C	-1.678335000000	-0.574466000000	-7.993467000000
C	-3.286133000000	-1.803214000000	-9.454584000000
C	-3.168447000000	-0.944540000000	-8.171813000000
C	-3.990405000000	0.358025000000	-8.255894000000
C	-4.584824000000	0.817763000000	-9.440513000000
C	-5.285493000000	2.022154000000	-9.489616000000
C	-5.410490000000	2.804849000000	-8.339185000000
C	-4.829907000000	2.365520000000	-7.144068000000
H	-4.506412000000	0.236688000000	-10.353182000000
H	-5.740393000000	2.367548000000	-10.412649000000
O	-6.108097000000	3.975003000000	-8.439317000000
H	-4.924434000000	2.964014000000	-6.239381000000
H	-3.696925000000	0.834440000000	-6.171348000000
O	-5.264702000000	-4.558003000000	-4.385811000000
H	-3.716383000000	-2.248019000000	-4.051902000000
H	-6.744892000000	-3.283888000000	-6.221960000000
H	-5.728651000000	-1.704791000000	-7.798924000000
H	-3.167494000000	-4.607793000000	-3.816934000000
H	-1.868227000000	-2.055106000000	-5.914937000000
H	-6.107819000000	4.419674000000	-7.577268000000
H	-2.717252000000	-2.730105000000	-9.329205000000
H	-1.506502000000	0.001030000000	-7.078277000000
H	-1.045131000000	-1.468664000000	-7.963935000000
H	-1.352537000000	0.040056000000	-8.838584000000
C	-12.128233000000	-11.280134000000	-1.924077000000
C	-10.781090000000	-10.589857000000	-1.857508000000
O	-9.729412000000	-11.198731000000	-2.081511000000
H	-12.361431000000	-11.491354000000	-2.973729000000
H	-12.065734000000	-12.240306000000	-1.405406000000
N	-10.787359000000	-9.266391000000	-1.525933000000
C	-9.610889000000	-8.409696000000	-1.722337000000
C	-8.471535000000	-8.677857000000	-0.728227000000
O	-7.337688000000	-8.259750000000	-0.971432000000
C	-10.019156000000	-6.932616000000	-1.680737000000
H	-11.684427000000	-8.803693000000	-1.452097000000
H	-9.164733000000	-8.638489000000	-2.697792000000
H	-10.728455000000	-6.706432000000	-2.483823000000

H	-10.473819000000	-6.668383000000	-0.718530000000
H	-9.133112000000	-6.310378000000	-1.824741000000
N	-8.751841000000	-9.383179000000	0.395720000000
C	-7.678958000000	-9.853234000000	1.255199000000
C	-6.869016000000	-11.040530000000	0.705109000000
O	-5.799606000000	-11.330595000000	1.245517000000
H	-9.698315000000	-9.706972000000	0.537160000000
H	-8.096837000000	-10.148483000000	2.222101000000
H	-6.968115000000	-9.044034000000	1.431545000000
N	-7.419774000000	-11.732759000000	-0.319490000000
C	-6.800317000000	-12.931011000000	-0.856968000000
C	-6.259540000000	-12.862895000000	-2.293410000000
O	-5.259014000000	-13.517742000000	-2.588252000000
H	-8.300933000000	-11.425038000000	-0.720545000000
H	-5.965156000000	-13.194852000000	-0.209471000000
N	-6.961568000000	-12.154047000000	-3.212493000000
C	-6.678874000000	-12.323823000000	-4.636538000000
C	-5.301324000000	-11.805279000000	-5.083589000000
O	-4.487018000000	-12.546007000000	-5.629361000000
H	-7.794897000000	-11.642050000000	-2.944391000000
H	-6.702230000000	-13.383556000000	-4.903941000000
N	-5.066905000000	-10.483456000000	-4.850860000000
C	-3.750410000000	-9.928269000000	-5.150043000000
C	-2.657548000000	-10.440303000000	-4.192067000000
O	-1.482776000000	-10.496839000000	-4.584286000000
C	-3.872110000000	-8.393228000000	-5.250312000000
O	-4.801005000000	-8.096961000000	-6.318496000000
C	-2.570435000000	-7.672208000000	-5.561301000000
H	-5.768779000000	-9.907188000000	-4.355222000000
H	-3.431966000000	-10.302737000000	-6.129801000000
H	-4.297034000000	-8.011752000000	-4.310148000000
H	-5.362522000000	-8.881069000000	-6.447322000000
H	-2.765006000000	-6.599997000000	-5.664485000000
H	-2.137624000000	-8.043920000000	-6.494711000000
H	-1.840600000000	-7.809388000000	-4.759874000000
N	-3.036109000000	-10.892957000000	-2.982424000000
C	-2.095323000000	-11.536562000000	-2.068008000000
C	-1.488738000000	-12.818803000000	-2.686352000000
O	-0.300533000000	-13.100861000000	-2.514928000000
C	-2.821290000000	-11.856132000000	-0.739113000000
O	-3.662737000000	-10.736149000000	-0.468247000000
C	-1.828665000000	-12.116008000000	0.391321000000
H	-3.967270000000	-10.714358000000	-2.627344000000
H	-1.245035000000	-10.871389000000	-1.884685000000
H	-3.444217000000	-12.747394000000	-0.900434000000
H	-4.263217000000	-10.961644000000	0.271571000000
H	-2.362194000000	-12.406255000000	1.303172000000
H	-1.129787000000	-12.916550000000	0.127655000000
H	-1.253744000000	-11.208008000000	0.602164000000
N	-2.330729000000	-13.586381000000	-3.418962000000
C	-1.864867000000	-14.762152000000	-4.135235000000
C	-1.084256000000	-14.494450000000	-5.439256000000
O	-0.623611000000	-15.432535000000	-6.073923000000
H	-3.315462000000	-13.346736000000	-3.484019000000
H	-1.201246000000	-15.349918000000	-3.494747000000
N	-0.949356000000	-13.181298000000	-5.780034000000
C	-0.124567000000	-12.696914000000	-6.873476000000

C	1.149428000000	-11.977573000000	-6.358493000000
O	1.853263000000	-11.313910000000	-7.115803000000
C	-0.953988000000	-11.734010000000	-7.789585000000
O	-2.292976000000	-12.187347000000	-7.951015000000
C	-0.366930000000	-11.611142000000	-9.190309000000
H	-1.329451000000	-12.474053000000	-5.163749000000
H	0.180072000000	-13.578702000000	-7.446465000000
H	-0.951606000000	-10.745337000000	-7.304222000000
H	-2.643617000000	-12.478012000000	-7.091461000000
H	-0.986379000000	-10.933594000000	-9.788049000000
H	-0.368666000000	-12.591917000000	-9.680268000000
H	0.654374000000	-11.231260000000	-9.142193000000
N	1.410763000000	-12.104889000000	-5.026217000000
C	2.506736000000	-11.412223000000	-4.366795000000
C	2.052661000000	-10.783606000000	-3.039291000000
O	1.036367000000	-9.797173000000	-3.247531000000
C	3.212518000000	-10.100262000000	-2.324908000000
H	0.834589000000	-12.714510000000	-4.457999000000
H	2.862988000000	-10.642924000000	-5.057178000000
H	1.649908000000	-11.580676000000	-2.398648000000
H	0.325331000000	-10.184699000000	-3.794302000000
H	2.860906000000	-9.624041000000	-1.404037000000
H	3.989368000000	-10.826819000000	-2.062635000000
H	3.661532000000	-9.328316000000	-2.961153000000
C	-7.794742000000	-5.519753000000	-8.808288000000
C	-6.519927000000	-5.405187000000	-9.654389000000
C	-5.658058000000	-6.682284000000	-9.722598000000
C	-6.401129000000	-7.856393000000	-10.379272000000
C	-4.341179000000	-6.401767000000	-10.462714000000
H	-7.570248000000	-5.820074000000	-7.777273000000
H	-6.784862000000	-5.101505000000	-10.678854000000
H	-5.899409000000	-4.593085000000	-9.248018000000
H	-5.404196000000	-6.974156000000	-8.692633000000
H	-3.778991000000	-5.590905000000	-9.982967000000
H	-3.697694000000	-7.289017000000	-10.480804000000
H	-4.529942000000	-6.106458000000	-11.503672000000
H	-7.311996000000	-8.126360000000	-9.833066000000
H	-6.691751000000	-7.608389000000	-11.409260000000
H	-5.764325000000	-8.748013000000	-10.421641000000
O	-7.048867000000	-8.798024000000	-3.725838000000
H	-6.911248000000	-8.511814000000	-2.800542000000
H	-7.085544000000	-7.955993000000	-4.258105000000
O	-6.927695000000	-6.595826000000	-5.306650000000
H	-6.537276000000	-5.805849000000	-4.876014000000
H	-6.168465000000	-6.967428000000	-5.809074000000
H	3.339739000000	-12.103750000000	-4.171927000000
H	-8.494713000000	-6.253399000000	-9.224078000000
H	-12.943280000000	-10.688534000000	-1.497161000000
H	-8.323009000000	-4.558667000000	-8.769115000000
H	-7.522820000000	-13.757930000000	-0.828079000000
H	-2.730642000000	-15.379569000000	-4.384996000000
H	-7.462514000000	-11.794727000000	-5.186350000000

^{2}TS10$_{B}$

C	-3.848136000000	-5.527839000000	-0.222900000000
C	-5.237972000000	-5.713405000000	-0.817080000000
O	-5.411625000000	-6.001825000000	-2.001340000000
C	-3.082062000000	-4.411256000000	-0.947692000000
H	-3.893980000000	-5.328626000000	0.851269000000
H	-2.992122000000	-4.675194000000	-2.006287000000
H	-2.075636000000	-4.309146000000	-0.536672000000
N	-6.281824000000	-5.514983000000	0.040393000000
C	-7.662558000000	-5.666885000000	-0.383428000000
C	-8.250721000000	-4.569558000000	-1.288543000000
O	-9.385057000000	-4.711043000000	-1.729385000000
H	-6.085797000000	-5.185214000000	0.975277000000
H	-7.764749000000	-6.598341000000	-0.947934000000
N	-7.448567000000	-3.503024000000	-1.554737000000
C	-7.794904000000	-2.512365000000	-2.553767000000
C	-6.996800000000	-2.581078000000	-3.866859000000
O	-7.045916000000	-1.632981000000	-4.647381000000
H	-6.518793000000	-3.459449000000	-1.149415000000
H	-7.667035000000	-1.501298000000	-2.156436000000
H	-8.852961000000	-2.650623000000	-2.793887000000
N	-6.301917000000	-3.725838000000	-4.087228000000
C	-5.523654000000	-3.933873000000	-5.293474000000
H	-6.278182000000	-4.446498000000	-3.373702000000
H	-4.481956000000	-4.168696000000	-5.046981000000
H	-5.933781000000	-4.755740000000	-5.893436000000
H	-5.561296000000	-3.012657000000	-5.877052000000
H	-8.297681000000	-5.748687000000	0.502859000000
H	-3.305576000000	-6.472270000000	-0.363842000000
S	-3.936337000000	-2.781494000000	-0.941083000000
Fe	-3.370726000000	-1.503361000000	0.810628000000
C	-6.092515000000	-2.417790000000	2.608523000000
C	-1.457379000000	-3.834813000000	2.443442000000
C	-0.619358000000	-0.467580000000	-0.941606000000
C	-5.288534000000	0.830358000000	-0.898947000000
N	-3.697918000000	-2.851538000000	2.241371000000
C	-4.902085000000	-3.096939000000	2.854322000000
C	-4.762191000000	-4.171036000000	3.808018000000
C	-3.458468000000	-4.569111000000	3.766314000000
C	-2.802106000000	-3.738672000000	2.788659000000
N	-1.432098000000	-2.015955000000	0.802554000000
C	-0.822056000000	-3.034020000000	1.506223000000
C	0.564603000000	-3.155894000000	1.121556000000
C	0.784939000000	-2.194988000000	0.184621000000
C	-0.459237000000	-1.490508000000	-0.008605000000
N	-2.973283000000	0.124174000000	-0.446201000000
C	-1.792197000000	0.214476000000	-1.203933000000
C	-2.002712000000	1.062879000000	-2.336387000000
C	-3.325365000000	1.416671000000	-2.343159000000
C	-3.942384000000	0.798980000000	-1.211071000000
N	-5.281305000000	-0.855669000000	0.896261000000
C	-5.900991000000	0.077309000000	0.104651000000
C	-7.307819000000	0.152215000000	0.424450000000
C	-7.532186000000	-0.751117000000	1.418037000000
C	-6.266167000000	-1.388019000000	1.694593000000
H	-6.964197000000	-2.726065000000	3.178464000000
H	-0.862375000000	-4.594115000000	2.941103000000

H	0.240241000000	-0.241657000000	-1.566912000000
H	-5.932992000000	1.443301000000	-1.521746000000
H	1.700275000000	-1.973217000000	-0.342370000000
H	1.274714000000	-3.884958000000	1.490955000000
H	-1.236867000000	1.301719000000	-3.064898000000
H	-3.855536000000	2.005563000000	-3.080937000000
H	-2.971590000000	-5.344374000000	4.343957000000
H	-5.561396000000	-4.549945000000	4.432739000000
H	-8.464655000000	-0.988009000000	1.914152000000
H	-8.016713000000	0.811650000000	-0.059820000000
O	-2.858382000000	-0.093759000000	2.393985000000
C	-2.800866000000	1.253044000000	1.916487000000
C	-5.116519000000	4.878049000000	-0.167390000000
C	-3.892761000000	3.440458000000	2.148189000000
C	-3.976352000000	2.078084000000	2.169455000000
C	-1.471636000000	1.856438000000	2.118602000000
C	-1.422118000000	3.295779000000	1.991283000000
C	-2.577243000000	4.024124000000	2.017178000000
H	-4.013404000000	5.723430000000	3.727901000000
H	-5.778112000000	5.764951000000	3.900156000000
C	-6.433322000000	3.624704000000	2.381255000000
C	-4.922409000000	5.113939000000	3.688749000000
C	-5.090546000000	4.389616000000	2.331054000000
C	-5.141074000000	5.375801000000	1.145224000000
C	-5.253915000000	6.765023000000	1.300317000000
C	-5.346443000000	7.622378000000	0.203198000000
C	-5.331972000000	7.101505000000	-1.091947000000
C	-5.212747000000	5.719165000000	-1.273404000000
H	-5.276234000000	7.204127000000	2.292051000000
H	-5.439089000000	8.695658000000	0.338271000000
O	-5.424607000000	7.981871000000	-2.136487000000
H	-5.193354000000	5.301306000000	-2.279116000000
H	-5.010463000000	3.808906000000	-0.330228000000
O	-0.488209000000	1.118496000000	2.373845000000
H	-2.834424000000	0.948041000000	0.657699000000
H	-0.448951000000	3.775770000000	2.011077000000
H	-2.509977000000	5.109292000000	1.977110000000
H	-1.920516000000	-0.302461000000	2.619086000000
H	-4.918342000000	1.559779000000	2.300579000000
H	-5.486771000000	7.480909000000	-2.964697000000
H	-4.860389000000	4.372981000000	4.492161000000
H	-6.605831000000	3.038130000000	1.473943000000
H	-6.470250000000	2.945000000000	3.240371000000
H	-7.258180000000	4.337631000000	2.481699000000
C	3.955841000000	8.424864000000	-2.574591000000
C	3.273389000000	7.154418000000	-2.110532000000
O	3.739237000000	6.038536000000	-2.365762000000
H	4.866188000000	8.569821000000	-1.982133000000
H	4.262168000000	8.306708000000	-3.617195000000
N	2.113281000000	7.303088000000	-1.410734000000
C	1.498734000000	6.194541000000	-0.669865000000
C	0.999235000000	5.061900000000	-1.575030000000
O	0.870414000000	3.920892000000	-1.130374000000
C	0.349471000000	6.723731000000	0.197312000000
H	1.838678000000	8.239872000000	-1.143592000000
H	2.250639000000	5.716992000000	-0.030147000000
H	0.725041000000	7.432418000000	0.943294000000

H	-0.416320000000	7.218625000000	-0.411047000000
H	-0.119617000000	5.890651000000	0.725411000000
N	0.691993000000	5.346981000000	-2.865466000000
C	0.319149000000	4.261095000000	-3.755852000000
C	1.440156000000	3.239150000000	-4.019107000000
O	1.137325000000	2.096340000000	-4.372463000000
H	0.806697000000	6.289602000000	-3.209056000000
H	0.007027000000	4.684336000000	-4.715513000000
H	-0.520511000000	3.702648000000	-3.337266000000
N	2.709206000000	3.700416000000	-3.915497000000
C	3.864994000000	2.895310000000	-4.267757000000
C	4.827515000000	2.469664000000	-3.147492000000
O	5.534009000000	1.477834000000	-3.329055000000
H	2.863636000000	4.644586000000	-3.574467000000
H	3.506913000000	1.987037000000	-4.750568000000
N	4.924115000000	3.248351000000	-2.042366000000
C	6.043999000000	3.075072000000	-1.117116000000
C	6.035497000000	1.741290000000	-0.352575000000
O	6.976489000000	0.955209000000	-0.433602000000
H	4.374060000000	4.096828000000	-1.966352000000
H	6.994243000000	3.107316000000	-1.656871000000
N	4.933536000000	1.504847000000	0.413055000000
C	4.808755000000	0.206863000000	1.066155000000
C	4.607261000000	-0.932583000000	0.053835000000
O	5.008475000000	-2.072349000000	0.325811000000
C	3.710671000000	0.264854000000	2.148141000000
O	4.064907000000	1.273757000000	3.113845000000
C	3.530552000000	-1.034525000000	2.918655000000
H	4.150344000000	2.182178000000	0.431093000000
H	5.757786000000	-0.029727000000	1.557878000000
H	2.760870000000	0.550629000000	1.670809000000
H	4.541535000000	1.982130000000	2.647313000000
H	2.806736000000	-0.872485000000	3.723549000000
H	4.478212000000	-1.354321000000	3.361805000000
H	3.157503000000	-1.834146000000	2.275775000000
N	4.028244000000	-0.630355000000	-1.123224000000
C	3.831470000000	-1.618805000000	-2.179302000000
C	5.159885000000	-2.275192000000	-2.622177000000
O	5.211322000000	-3.482046000000	-2.862565000000
C	3.125439000000	-0.937734000000	-3.380906000000
O	2.192680000000	-0.004097000000	-2.827964000000
C	2.446059000000	-1.966671000000	-4.280353000000
H	3.622718000000	0.283772000000	-1.283676000000
H	3.221508000000	-2.446663000000	-1.802822000000
H	3.887853000000	-0.386376000000	-3.947612000000
H	1.848292000000	0.583324000000	-3.532195000000
H	2.016020000000	-1.478663000000	-5.162157000000
H	3.160341000000	-2.725404000000	-4.616974000000
H	1.639012000000	-2.468231000000	-3.735594000000
N	6.228353000000	-1.447636000000	-2.727642000000
C	7.549540000000	-1.950986000000	-3.069391000000
C	8.326201000000	-2.653492000000	-1.935370000000
O	9.432422000000	-3.123135000000	-2.163514000000
H	6.118323000000	-0.451624000000	-2.560136000000
H	7.466919000000	-2.678174000000	-3.881978000000
N	7.679052000000	-2.717624000000	-0.740420000000
C	8.184470000000	-3.406402000000	0.433297000000

C	7.297250000000	-4.620412000000	0.810985000000
O	7.415573000000	-5.179709000000	1.898705000000
C	8.302569000000	-2.413677000000	1.641287000000
O	8.784053000000	-1.144618000000	1.224737000000
C	9.272320000000	-2.909654000000	2.706874000000
H	6.755473000000	-2.311200000000	-0.658615000000
H	9.182649000000	-3.771438000000	0.169705000000
H	7.296513000000	-2.313985000000	2.080491000000
H	8.189769000000	-0.776580000000	0.549238000000
H	9.315387000000	-2.185813000000	3.527787000000
H	10.277910000000	-2.995577000000	2.278259000000
H	8.955970000000	-3.882037000000	3.085929000000
N	6.378874000000	-5.003296000000	-0.121726000000
C	5.410978000000	-6.064069000000	0.111584000000
C	3.987824000000	-5.630633000000	-0.279824000000
O	3.532866000000	-4.535472000000	0.512825000000
C	2.992848000000	-6.769673000000	-0.087107000000
H	6.362162000000	-4.535300000000	-1.019868000000
H	5.457055000000	-6.309621000000	1.175708000000
H	3.999352000000	-5.336400000000	-1.341774000000
H	4.185908000000	-3.814062000000	0.442508000000
H	1.985355000000	-6.438656000000	-0.360017000000
H	3.254553000000	-7.631186000000	-0.711212000000
H	2.976941000000	-7.089240000000	0.961483000000
C	0.993186000000	2.953136000000	6.703284000000
C	1.566647000000	1.655253000000	7.286444000000
C	3.068327000000	1.424264000000	7.019849000000
C	3.952791000000	2.490197000000	7.685160000000
C	3.485836000000	0.018669000000	7.478213000000
H	1.143088000000	3.002564000000	5.617975000000
H	1.392448000000	1.629294000000	8.373118000000
H	1.006414000000	0.805376000000	6.870936000000
H	3.231738000000	1.482597000000	5.932884000000
H	2.891300000000	-0.756903000000	6.979861000000
H	4.541956000000	-0.176271000000	7.257377000000
H	3.345136000000	-0.099955000000	8.561091000000
H	3.724694000000	3.498895000000	7.323731000000
H	3.816078000000	2.486543000000	8.775192000000
H	5.013798000000	2.299443000000	7.484149000000
O	2.904426000000	3.446635000000	0.730744000000
H	2.183188000000	3.415083000000	0.069982000000
H	2.437823000000	3.275967000000	1.595290000000
O	1.685689000000	2.744420000000	3.052741000000
H	0.964861000000	2.099999000000	2.850324000000
H	2.437992000000	2.204875000000	3.377059000000
H	5.685131000000	-6.964140000000	-0.458495000000
H	1.458122000000	3.840622000000	7.148241000000
H	3.330064000000	9.316784000000	-2.478007000000
H	-0.084773000000	3.020615000000	6.894391000000
H	4.473039000000	3.436443000000	-5.005004000000
H	8.158633000000	-1.115720000000	-3.422605000000
H	6.002177000000	3.904433000000	-0.405356000000

^{4}TS10$_{B}$

C	-3.848136000000	-5.527839000000	-0.222900000000
C	-5.237972000000	-5.713405000000	-0.817080000000
O	-5.411625000000	-6.001825000000	-2.001340000000
C	-3.082062000000	-4.411256000000	-0.947692000000
H	-3.893980000000	-5.328626000000	0.851269000000
H	-2.992122000000	-4.675194000000	-2.006287000000
H	-2.075636000000	-4.309146000000	-0.536672000000
N	-6.281824000000	-5.514983000000	0.040393000000
C	-7.662558000000	-5.666885000000	-0.383428000000
C	-8.250721000000	-4.569558000000	-1.288543000000
O	-9.385057000000	-4.711043000000	-1.729385000000
H	-6.085797000000	-5.185214000000	0.975277000000
H	-7.764749000000	-6.598341000000	-0.947934000000
N	-7.448567000000	-3.503024000000	-1.554737000000
C	-7.794904000000	-2.512365000000	-2.553767000000
C	-6.996800000000	-2.581078000000	-3.866859000000
O	-7.045916000000	-1.632981000000	-4.647381000000
H	-6.518793000000	-3.459449000000	-1.149415000000
H	-7.667035000000	-1.501298000000	-2.156436000000
H	-8.852961000000	-2.650623000000	-2.793887000000
N	-6.301917000000	-3.725838000000	-4.087228000000
C	-5.523654000000	-3.933873000000	-5.293474000000
H	-6.278182000000	-4.446498000000	-3.373702000000
H	-4.481956000000	-4.168696000000	-5.046981000000
H	-5.933781000000	-4.755740000000	-5.893436000000
H	-5.561296000000	-3.012657000000	-5.877052000000
H	-8.297681000000	-5.748687000000	0.502859000000
H	-3.305576000000	-6.472270000000	-0.363842000000
S	-3.936337000000	-2.781494000000	-0.941083000000
Fe	-3.370726000000	-1.503361000000	0.810628000000
C	-6.092515000000	-2.417790000000	2.608523000000
C	-1.457379000000	-3.834813000000	2.443442000000
C	-0.619358000000	-0.467580000000	-0.941606000000
C	-5.288534000000	0.830358000000	-0.898947000000
N	-3.697918000000	-2.851538000000	2.241371000000
C	-4.902085000000	-3.096939000000	2.854322000000
C	-4.762191000000	-4.171036000000	3.808018000000
C	-3.458468000000	-4.569111000000	3.766314000000
C	-2.802106000000	-3.738672000000	2.788659000000
N	-1.432098000000	-2.015955000000	0.802554000000
C	-0.822056000000	-3.034020000000	1.506223000000
C	0.564603000000	-3.155894000000	1.121556000000
C	0.784939000000	-2.194988000000	0.184621000000
C	-0.459237000000	-1.490508000000	-0.008605000000
N	-2.973283000000	0.124174000000	-0.446201000000
C	-1.792197000000	0.214476000000	-1.203933000000
C	-2.002712000000	1.062879000000	-2.336387000000
C	-3.325365000000	1.416671000000	-2.343159000000
C	-3.942384000000	0.798980000000	-1.211071000000
N	-5.281305000000	-0.855669000000	0.896261000000
C	-5.900991000000	0.077309000000	0.104651000000
C	-7.307819000000	0.152215000000	0.424450000000
C	-7.532186000000	-0.751117000000	1.418037000000
C	-6.266167000000	-1.388019000000	1.694593000000
H	-6.964197000000	-2.726065000000	3.178464000000
H	-0.862375000000	-4.594115000000	2.941103000000

H	0.240241000000	-0.241657000000	-1.566912000000
H	-5.932992000000	1.443301000000	-1.521746000000
H	1.700275000000	-1.973217000000	-0.342370000000
H	1.274714000000	-3.884958000000	1.490955000000
H	-1.236867000000	1.301719000000	-3.064898000000
H	-3.855536000000	2.005563000000	-3.080937000000
H	-2.971590000000	-5.344374000000	4.343957000000
H	-5.561396000000	-4.549945000000	4.432739000000
H	-8.464655000000	-0.988009000000	1.914152000000
H	-8.016713000000	0.811650000000	-0.059820000000
O	-2.858382000000	-0.093759000000	2.393985000000
C	-2.800866000000	1.253044000000	1.916487000000
C	-5.116519000000	4.878049000000	-0.167390000000
C	-3.892761000000	3.440458000000	2.148189000000
C	-3.976352000000	2.078084000000	2.169455000000
C	-1.471636000000	1.856438000000	2.118602000000
C	-1.422118000000	3.295779000000	1.991283000000
C	-2.577243000000	4.024124000000	2.017178000000
H	-4.013404000000	5.723430000000	3.727901000000
H	-5.778112000000	5.764951000000	3.900156000000
C	-6.433322000000	3.624704000000	2.381255000000
C	-4.922409000000	5.113939000000	3.688749000000
C	-5.090546000000	4.389616000000	2.331054000000
C	-5.141074000000	5.375801000000	1.145224000000
C	-5.253915000000	6.765023000000	1.300317000000
C	-5.346443000000	7.622378000000	0.203198000000
C	-5.331972000000	7.101505000000	-1.091947000000
C	-5.212747000000	5.719165000000	-1.273404000000
H	-5.276234000000	7.204127000000	2.292051000000
H	-5.439089000000	8.695658000000	0.338271000000
O	-5.424607000000	7.981871000000	-2.136487000000
H	-5.193354000000	5.301306000000	-2.279116000000
H	-5.010463000000	3.808906000000	-0.330228000000
O	-0.488209000000	1.118496000000	2.373845000000
H	-2.834424000000	0.948041000000	0.657699000000
H	-0.448951000000	3.775770000000	2.011077000000
H	-2.509977000000	5.109292000000	1.977110000000
H	-1.920516000000	-0.302461000000	2.619086000000
H	-4.918342000000	1.559779000000	2.300579000000
H	-5.486771000000	7.480909000000	-2.964697000000
H	-4.860389000000	4.372981000000	4.492161000000
H	-6.605831000000	3.038130000000	1.473943000000
H	-6.470250000000	2.945000000000	3.240371000000
H	-7.258180000000	4.337631000000	2.481699000000
C	3.955841000000	8.424864000000	-2.574591000000
C	3.273389000000	7.154418000000	-2.110532000000
O	3.739237000000	6.038536000000	-2.365762000000
H	4.866188000000	8.569821000000	-1.982133000000
H	4.262168000000	8.306708000000	-3.617195000000
N	2.113281000000	7.303088000000	-1.410734000000
C	1.498734000000	6.194541000000	-0.669865000000
C	0.999235000000	5.061900000000	-1.575030000000
O	0.870414000000	3.920892000000	-1.130374000000
C	0.349471000000	6.723731000000	0.197312000000
H	1.838678000000	8.239872000000	-1.143592000000
H	2.250639000000	5.716992000000	-0.030147000000
H	0.725041000000	7.432418000000	0.943294000000

H	-0.416320000000	7.218625000000	-0.411047000000
H	-0.119617000000	5.890651000000	0.725411000000
N	0.691993000000	5.346981000000	-2.865466000000
C	0.319149000000	4.261095000000	-3.755852000000
C	1.440156000000	3.239150000000	-4.019107000000
O	1.137325000000	2.096340000000	-4.372463000000
H	0.806697000000	6.289602000000	-3.209056000000
H	0.007027000000	4.684336000000	-4.715513000000
H	-0.520511000000	3.702648000000	-3.337266000000
N	2.709206000000	3.700416000000	-3.915497000000
C	3.864994000000	2.895310000000	-4.267757000000
C	4.827515000000	2.469664000000	-3.147492000000
O	5.534009000000	1.477834000000	-3.329055000000
H	2.863636000000	4.644586000000	-3.574467000000
H	3.506913000000	1.987037000000	-4.750568000000
N	4.924115000000	3.248351000000	-2.042366000000
C	6.043999000000	3.075072000000	-1.117116000000
C	6.035497000000	1.741290000000	-0.352575000000
O	6.976489000000	0.955209000000	-0.433602000000
H	4.374060000000	4.096828000000	-1.966352000000
H	6.994243000000	3.107316000000	-1.656871000000
N	4.933536000000	1.504847000000	0.413055000000
C	4.808755000000	0.206863000000	1.066155000000
C	4.607261000000	-0.932583000000	0.053835000000
O	5.008475000000	-2.072349000000	0.325811000000
C	3.710671000000	0.264854000000	2.148141000000
O	4.064907000000	1.273757000000	3.113845000000
C	3.530552000000	-1.034525000000	2.918655000000
H	4.150344000000	2.182178000000	0.431093000000
H	5.757786000000	-0.029727000000	1.557878000000
H	2.760870000000	0.550629000000	1.670809000000
H	4.541535000000	1.982130000000	2.647313000000
H	2.806736000000	-0.872485000000	3.723549000000
H	4.478212000000	-1.354321000000	3.361805000000
H	3.157503000000	-1.834146000000	2.275775000000
N	4.028244000000	-0.630355000000	-1.123224000000
C	3.831470000000	-1.618805000000	-2.179302000000
C	5.159885000000	-2.275192000000	-2.622177000000
O	5.211322000000	-3.482046000000	-2.862565000000
C	3.125439000000	-0.937734000000	-3.380906000000
O	2.192680000000	-0.004097000000	-2.827964000000
C	2.446059000000	-1.966671000000	-4.280353000000
H	3.622718000000	0.283772000000	-1.283676000000
H	3.221508000000	-2.446663000000	-1.802822000000
H	3.887853000000	-0.386376000000	-3.947612000000
H	1.848292000000	0.583324000000	-3.532195000000
H	2.016020000000	-1.478663000000	-5.162157000000
H	3.160341000000	-2.725404000000	-4.616974000000
H	1.639012000000	-2.468231000000	-3.735594000000
N	6.228353000000	-1.447636000000	-2.727642000000
C	7.549540000000	-1.950986000000	-3.069391000000
C	8.326201000000	-2.653492000000	-1.935370000000
O	9.432422000000	-3.123135000000	-2.163514000000
H	6.118323000000	-0.451624000000	-2.560136000000
H	7.466919000000	-2.678174000000	-3.881978000000
N	7.679052000000	-2.717624000000	-0.740420000000
C	8.184470000000	-3.406402000000	0.433297000000

C	7.297250000000	-4.620412000000	0.810985000000
O	7.415573000000	-5.179709000000	1.898705000000
C	8.302569000000	-2.413677000000	1.641287000000
O	8.784053000000	-1.144618000000	1.224737000000
C	9.272320000000	-2.909654000000	2.706874000000
H	6.755473000000	-2.311200000000	-0.658615000000
H	9.182649000000	-3.771438000000	0.169705000000
H	7.296513000000	-2.313985000000	2.080491000000
H	8.189769000000	-0.776580000000	0.549238000000
H	9.315387000000	-2.185813000000	3.527787000000
H	10.277910000000	-2.995577000000	2.278259000000
H	8.955970000000	-3.882037000000	3.085929000000
N	6.378874000000	-5.003296000000	-0.121726000000
C	5.410978000000	-6.064069000000	0.111584000000
C	3.987824000000	-5.630633000000	-0.279824000000
O	3.532866000000	-4.535472000000	0.512825000000
C	2.992848000000	-6.769673000000	-0.087107000000
H	6.362162000000	-4.535300000000	-1.019868000000
H	5.457055000000	-6.309621000000	1.175708000000
H	3.999352000000	-5.336400000000	-1.341774000000
H	4.185908000000	-3.814062000000	0.442508000000
H	1.985355000000	-6.438656000000	-0.360017000000
H	3.254553000000	-7.631186000000	-0.711212000000
H	2.976941000000	-7.089240000000	0.961483000000
C	0.993186000000	2.953136000000	6.703284000000
C	1.566647000000	1.655253000000	7.286444000000
C	3.068327000000	1.424264000000	7.019849000000
C	3.952791000000	2.490197000000	7.685160000000
C	3.485836000000	0.018669000000	7.478213000000
H	1.143088000000	3.002564000000	5.617975000000
H	1.392448000000	1.629294000000	8.373118000000
H	1.006414000000	0.805376000000	6.870936000000
H	3.231738000000	1.482597000000	5.932884000000
H	2.891300000000	-0.756903000000	6.979861000000
H	4.541956000000	-0.176271000000	7.257377000000
H	3.345136000000	-0.099955000000	8.561091000000
H	3.724694000000	3.498895000000	7.323731000000
H	3.816078000000	2.486543000000	8.775192000000
H	5.013798000000	2.299443000000	7.484149000000
O	2.904426000000	3.446635000000	0.730744000000
H	2.183188000000	3.415083000000	0.069982000000
H	2.437823000000	3.275967000000	1.595290000000
O	1.685689000000	2.744420000000	3.052741000000
H	0.964861000000	2.099999000000	2.850324000000
H	2.437992000000	2.204875000000	3.377059000000
H	5.685131000000	-6.964140000000	-0.458495000000
H	1.458122000000	3.840622000000	7.148241000000
H	3.330064000000	9.316784000000	-2.478007000000
H	-0.084773000000	3.020615000000	6.894391000000
H	4.473039000000	3.436443000000	-5.005004000000
H	8.158633000000	-1.115720000000	-3.422605000000
H	6.002177000000	3.904433000000	-0.405356000000

^{2}TS7$_{pB}$

C	1.222098000000	-4.473540000000	0.037163000000
C	1.854901000000	-3.227302000000	0.640215000000
O	1.965523000000	-3.070987000000	1.857201000000
C	-0.259351000000	-4.590825000000	0.430540000000
H	1.329121000000	-4.489738000000	-1.050373000000
H	-0.334711000000	-4.663184000000	1.521049000000
H	-0.690760000000	-5.503481000000	0.008778000000
N	2.270142000000	-2.281283000000	-0.252222000000
C	2.882589000000	-1.036562000000	0.176260000000
C	1.968672000000	0.020126000000	0.822411000000
O	2.472172000000	1.040486000000	1.277651000000
H	2.036702000000	-2.403528000000	-1.228105000000
H	3.649922000000	-1.254976000000	0.924492000000
N	0.639250000000	-0.266496000000	0.862415000000
C	-0.289768000000	0.520437000000	1.647274000000
C	-0.732399000000	-0.097226000000	2.985718000000
O	-1.655587000000	0.424163000000	3.606399000000
H	0.297642000000	-1.131855000000	0.452982000000
H	-1.200842000000	0.721771000000	1.075630000000
H	0.192678000000	1.477018000000	1.865574000000
N	-0.044088000000	-1.189746000000	3.403994000000
C	-0.344159000000	-1.848367000000	4.660672000000
H	0.701012000000	-1.572992000000	2.831496000000
H	-1.279213000000	-1.434108000000	5.041486000000
H	-0.453290000000	-2.927775000000	4.509056000000
H	0.447597000000	-1.679128000000	5.401940000000
H	3.380432000000	-0.574602000000	-0.680666000000
H	1.763424000000	-5.339504000000	0.440058000000
S	-1.298707000000	-3.147613000000	-0.021619000000
Fe	-2.101570000000	-3.229966000000	-2.259027000000
C	-2.644571000000	0.126609000000	-1.767382000000
C	0.844515000000	-2.534854000000	-3.809557000000
C	-1.393945000000	-6.603687000000	-2.443362000000
C	-5.156329000000	-3.930705000000	-0.995723000000
N	-1.107214000000	-1.535305000000	-2.702880000000
C	-1.459792000000	-0.245185000000	-2.396639000000
C	-0.447127000000	0.676216000000	-2.852262000000
C	0.518561000000	-0.072116000000	-3.453866000000
C	0.101900000000	-1.450998000000	-3.346357000000
N	-0.589989000000	-4.348154000000	-2.985016000000
C	0.524642000000	-3.878213000000	-3.639982000000
C	1.350893000000	-4.979601000000	-4.079005000000
C	0.737967000000	-6.125283000000	-3.669480000000
C	-0.470607000000	-5.717985000000	-2.992059000000
N	-3.066539000000	-4.936854000000	-1.797878000000
C	-2.621514000000	-6.233322000000	-1.900192000000
C	-3.638371000000	-7.154771000000	-1.445181000000
C	-4.714940000000	-6.396146000000	-1.098298000000
C	-4.344068000000	-5.015658000000	-1.304881000000
N	-3.608139000000	-2.110700000000	-1.539264000000
C	-4.796924000000	-2.586571000000	-1.064044000000
C	-5.627659000000	-1.495905000000	-0.605869000000
C	-4.913116000000	-0.353051000000	-0.796608000000
C	-3.655440000000	-0.748006000000	-1.386757000000
H	-2.801127000000	1.185125000000	-1.582003000000
H	1.769489000000	-2.311551000000	-4.334347000000

H	-1.144066000000	-7.659871000000	-2.491221000000
H	-6.146092000000	-4.155098000000	-0.609237000000
H	1.026880000000	-7.159705000000	-3.815829000000
H	2.277739000000	-4.873442000000	-4.628736000000
H	-3.538171000000	-8.232886000000	-1.397568000000
H	-5.682892000000	-6.730045000000	-0.748860000000
H	1.434756000000	0.262830000000	-3.923630000000
H	-0.485436000000	1.750335000000	-2.724579000000
H	-5.192192000000	0.665571000000	-0.559371000000
H	-6.615566000000	-1.609113000000	-0.177802000000
O	-2.936246000000	-3.287767000000	-3.996346000000
C	-6.360410000000	-2.328334000000	-4.156349000000
C	-5.295995000000	-0.749312000000	-7.783745000000
C	-4.380783000000	-2.655135000000	-5.577859000000
C	-5.180524000000	-1.873238000000	-4.661498000000
C	-6.887891000000	-3.633600000000	-4.525178000000
C	-6.049031000000	-4.446802000000	-5.392383000000
C	-4.877920000000	-3.974390000000	-5.897639000000
H	-2.471614000000	-3.881820000000	-7.233727000000
H	-1.582156000000	-2.417375000000	-7.656567000000
C	-2.829548000000	-0.657928000000	-6.002981000000
C	-2.194369000000	-2.885321000000	-6.878706000000
C	-3.402729000000	-1.980062000000	-6.556570000000
C	-4.263694000000	-1.701992000000	-7.820303000000
C	-4.069113000000	-2.384077000000	-9.033505000000
C	-4.847753000000	-2.121641000000	-10.157650000000
C	-5.862459000000	-1.162366000000	-10.096805000000
C	-6.084866000000	-0.476279000000	-8.898066000000
H	-3.288859000000	-3.130996000000	-9.121741000000
H	-4.677380000000	-2.649657000000	-11.090546000000
O	-6.596475000000	-0.941054000000	-11.226204000000
H	-6.872720000000	0.272581000000	-8.835343000000
H	-5.501479000000	-0.204908000000	-6.867595000000
O	-8.017916000000	-4.023978000000	-4.128708000000
H	-6.952180000000	-1.727267000000	-3.473522000000
H	-6.389869000000	-5.443238000000	-5.644920000000
H	-4.320982000000	-4.606165000000	-6.580650000000
H	-2.890907000000	-4.222266000000	-4.262123000000
H	-4.815004000000	-0.897779000000	-4.363343000000
H	-7.248346000000	-0.244488000000	-11.050404000000
H	-1.590982000000	-3.004925000000	-5.978467000000
H	-3.589969000000	0.111221000000	-5.843210000000
H	-2.316346000000	-0.842115000000	-5.056325000000
H	-2.108977000000	-0.251415000000	-6.720015000000
C	-12.482482000000	-10.795097000000	-3.048123000000
C	-11.151189000000	-10.084170000000	-2.916222000000
O	-10.082179000000	-10.654477000000	-3.160921000000
H	-12.690047000000	-10.954790000000	-4.112131000000
H	-12.411682000000	-11.780118000000	-2.579281000000
N	-11.186999000000	-8.785725000000	-2.499532000000
C	-10.021763000000	-7.900036000000	-2.634466000000
C	-8.851938000000	-8.296540000000	-1.726047000000
O	-7.699314000000	-7.972259000000	-2.019105000000
C	-10.431337000000	-6.445637000000	-2.370918000000
H	-12.092188000000	-8.337798000000	-2.431290000000
H	-9.633671000000	-7.981559000000	-3.653031000000
H	-11.170968000000	-6.122193000000	-3.109968000000

H	-10.846036000000	-6.324637000000	-1.363212000000
H	-9.561161000000	-5.792897000000	-2.472984000000
N	-9.140845000000	-8.973907000000	-0.586324000000
C	-8.086741000000	-9.486151000000	0.271578000000
C	-7.387456000000	-10.765834000000	-0.216429000000
O	-6.381395000000	-11.155899000000	0.380186000000
H	-10.103665000000	-9.226356000000	-0.411403000000
H	-8.498998000000	-9.690553000000	1.263903000000
H	-7.309550000000	-8.727980000000	0.386278000000
N	-7.960614000000	-11.416640000000	-1.253387000000
C	-7.425232000000	-12.664150000000	-1.771278000000
C	-6.715656000000	-12.586940000000	-3.133649000000
O	-5.664595000000	-13.200707000000	-3.312505000000
H	-8.767866000000	-11.010927000000	-1.717454000000
H	-6.699634000000	-13.043661000000	-1.052906000000
N	-7.333547000000	-11.891420000000	-4.123894000000
C	-6.866081000000	-12.010414000000	-5.503759000000
C	-5.444198000000	-11.473960000000	-5.739733000000
O	-4.563152000000	-12.194284000000	-6.207156000000
H	-8.212185000000	-11.417719000000	-3.945399000000
H	-6.846538000000	-13.059345000000	-5.811868000000
N	-5.233862000000	-10.178126000000	-5.390946000000
C	-3.912952000000	-9.600793000000	-5.616962000000
C	-2.876848000000	-10.266428000000	-4.700426000000
O	-1.698752000000	-10.391539000000	-5.071385000000
C	-3.923713000000	-8.053192000000	-5.446403000000
O	-4.951217000000	-7.482264000000	-6.240176000000
C	-2.603169000000	-7.438518000000	-5.898218000000
H	-6.030459000000	-9.574404000000	-5.187742000000
H	-3.590143000000	-9.828568000000	-6.638185000000
H	-4.094434000000	-7.824252000000	-4.381825000000
H	-5.797692000000	-7.623552000000	-5.763995000000
H	-2.620567000000	-6.360737000000	-5.709368000000
H	-2.472276000000	-7.596655000000	-6.974096000000
H	-1.744077000000	-7.871178000000	-5.382611000000
N	-3.304028000000	-10.700629000000	-3.500115000000
C	-2.425492000000	-11.354539000000	-2.535682000000
C	-1.795254000000	-12.644483000000	-3.106766000000
O	-0.623889000000	-12.937157000000	-2.856323000000
C	-3.237896000000	-11.662128000000	-1.255504000000
O	-4.034033000000	-10.504476000000	-0.999777000000
C	-2.328553000000	-11.997446000000	-0.075925000000
H	-4.244748000000	-10.491657000000	-3.189808000000
H	-1.583202000000	-10.695832000000	-2.297826000000
H	-3.899286000000	-12.512639000000	-1.472902000000
H	-4.731028000000	-10.743089000000	-0.355863000000
H	-2.931044000000	-12.269256000000	0.797803000000
H	-1.661071000000	-12.832312000000	-0.312029000000
H	-1.716528000000	-11.127333000000	0.184694000000
N	-2.601028000000	-13.414944000000	-3.877115000000
C	-2.099642000000	-14.604196000000	-4.547294000000
C	-1.195419000000	-14.360537000000	-5.774477000000
O	-0.671092000000	-15.311126000000	-6.338509000000
H	-3.564295000000	-13.138828000000	-4.041383000000
H	-1.511617000000	-15.204545000000	-3.847698000000
N	-1.028829000000	-13.055953000000	-6.120887000000
C	-0.161192000000	-12.590568000000	-7.185468000000

C	0.999464000000	-11.727873000000	-6.627054000000
O	1.662218000000	-10.997522000000	-7.365201000000
C	-0.983491000000	-11.796341000000	-8.258904000000
O	-2.249969000000	-12.396283000000	-8.482703000000
C	-0.280372000000	-11.760761000000	-9.610249000000
H	-1.505988000000	-12.338718000000	-5.588940000000
H	0.262902000000	-13.487419000000	-7.650174000000
H	-1.108887000000	-10.768917000000	-7.882119000000
H	-2.758244000000	-12.392997000000	-7.653778000000
H	-0.880734000000	-11.184566000000	-10.322367000000
H	-0.180011000000	-12.780797000000	-9.999879000000
H	0.708666000000	-11.310895000000	-9.513141000000
N	1.219176000000	-11.825712000000	-5.287564000000
C	2.212740000000	-11.035836000000	-4.576771000000
C	1.631528000000	-10.411858000000	-3.297122000000
O	0.589389000000	-9.475941000000	-3.595063000000
C	2.702627000000	-9.665405000000	-2.510836000000
H	0.653702000000	-12.468216000000	-4.744167000000
H	2.557590000000	-10.253821000000	-5.257854000000
H	1.219892000000	-11.219792000000	-2.674942000000
H	-0.065441000000	-9.911926000000	-4.176650000000
H	2.259368000000	-9.188004000000	-1.630897000000
H	3.487423000000	-10.350582000000	-2.172211000000
H	3.165826000000	-8.886023000000	-3.127562000000
C	4.671262000000	-5.598809000000	-9.284640000000
C	3.314750000000	-5.805143000000	-8.599916000000
C	2.880119000000	-7.275802000000	-8.442319000000
C	3.821707000000	-8.080955000000	-7.533127000000
C	1.435412000000	-7.366171000000	-7.927018000000
H	4.685972000000	-6.067298000000	-10.276817000000
H	3.330473000000	-5.331650000000	-7.605438000000
H	2.543816000000	-5.272308000000	-9.175338000000
H	2.906114000000	-7.737943000000	-9.441781000000
H	0.738280000000	-6.855200000000	-8.603860000000
H	1.122943000000	-8.411158000000	-7.830860000000
H	1.341469000000	-6.895915000000	-6.938031000000
H	4.846300000000	-8.110092000000	-7.921459000000
H	3.860444000000	-7.638051000000	-6.527147000000
H	3.465328000000	-9.111808000000	-7.434847000000
O	-7.245769000000	-8.104895000000	-4.794841000000
H	-7.228177000000	-7.942266000000	-3.826846000000
H	-7.930034000000	-7.472784000000	-5.134329000000
O	-9.087950000000	-6.189081000000	-5.373422000000
H	-8.708583000000	-5.392794000000	-4.906960000000
H	-9.207542000000	-5.910187000000	-6.294075000000
H	3.078123000000	-11.660720000000	-4.311087000000
H	5.493440000000	-6.030518000000	-8.703394000000
H	-13.317446000000	-10.242574000000	-2.607769000000
H	4.888183000000	-4.532189000000	-9.418939000000
H	-8.240067000000	-13.394733000000	-1.858945000000
H	-2.953885000000	-15.202316000000	-4.873061000000
H	-7.573728000000	-11.465658000000	-6.136205000000

^{4}TS7$_{pB}$

C	1.821535000000	-2.984364000000	-0.268735000000
C	1.808263000000	-1.766485000000	0.645068000000
O	1.929483000000	-1.864641000000	1.867163000000
C	0.664217000000	-3.940386000000	0.060158000000
H	1.788737000000	-2.696425000000	-1.323376000000
H	0.758623000000	-4.268929000000	1.100601000000
H	0.711678000000	-4.825008000000	-0.579461000000
N	1.626812000000	-0.561205000000	0.031409000000
C	1.594807000000	0.681578000000	0.781455000000
C	0.345139000000	0.962715000000	1.635648000000
O	0.326960000000	1.961365000000	2.345597000000
H	1.440022000000	-0.536427000000	-0.962133000000
H	2.436406000000	0.703452000000	1.480015000000
N	-0.668848000000	0.058838000000	1.552206000000
C	-1.787395000000	0.075417000000	2.474204000000
C	-1.743986000000	-0.965917000000	3.607029000000
O	-2.741197000000	-1.125142000000	4.306774000000
H	-0.574778000000	-0.745934000000	0.939758000000
H	-2.728289000000	-0.079860000000	1.937669000000
H	-1.818488000000	1.066560000000	2.934557000000
N	-0.574563000000	-1.635814000000	3.768153000000
C	-0.399348000000	-2.632210000000	4.807321000000
H	0.200740000000	-1.466781000000	3.135708000000
H	-0.082955000000	-3.589250000000	4.376791000000
H	0.352759000000	-2.314103000000	5.540065000000
H	-1.358562000000	-2.758806000000	5.311786000000
H	1.722541000000	1.515306000000	0.085621000000
H	2.769583000000	-3.510716000000	-0.095292000000
S	-1.006705000000	-3.177913000000	-0.056097000000
Fe	-1.930494000000	-3.265488000000	-2.286339000000
C	-2.272036000000	0.120742000000	-1.866666000000
C	0.970438000000	-2.766694000000	-4.026673000000
C	-1.455901000000	-6.653581000000	-2.436857000000
C	-4.973072000000	-3.757982000000	-0.797972000000
N	-0.883954000000	-1.642785000000	-2.875619000000
C	-1.161414000000	-0.330592000000	-2.567587000000
C	-0.128761000000	0.524360000000	-3.094628000000
C	0.771778000000	-0.284969000000	-3.728114000000
C	0.296290000000	-1.636459000000	-3.575825000000
N	-0.520182000000	-4.455794000000	-3.036034000000
C	0.587157000000	-4.072770000000	-3.765398000000
C	1.318241000000	-5.236388000000	-4.196985000000
C	0.656560000000	-6.329582000000	-3.722972000000
C	-0.495212000000	-5.833639000000	-3.012561000000
N	-3.027642000000	-4.884341000000	-1.794567000000
C	-2.642658000000	-6.202591000000	-1.877463000000
C	-3.668480000000	-7.057246000000	-1.331246000000
C	-4.675940000000	-6.238307000000	-0.918602000000
C	-4.260243000000	-4.883183000000	-1.180052000000
N	-3.327962000000	-2.059642000000	-1.461514000000
C	-4.522705000000	-2.447207000000	-0.914009000000
C	-5.252459000000	-1.292006000000	-0.455349000000
C	-4.482368000000	-0.202031000000	-0.733839000000
C	-3.287093000000	-0.689520000000	-1.376167000000
H	-2.361848000000	1.190034000000	-1.704526000000
H	1.882855000000	-2.616407000000	-4.594942000000

H	-1.276708000000	-7.723198000000	-2.467404000000
H	-5.938062000000	-3.914193000000	-0.327052000000
H	0.882269000000	-7.382424000000	-3.858689000000
H	2.217478000000	-5.207148000000	-4.799018000000
H	-3.606940000000	-8.138205000000	-1.272054000000
H	-5.624517000000	-6.513694000000	-0.478193000000
H	1.675807000000	-0.004736000000	-4.254038000000
H	-0.113964000000	1.602939000000	-2.999687000000
H	-4.693065000000	0.840993000000	-0.534620000000
H	-6.220790000000	-1.327192000000	0.027113000000
O	-2.961829000000	-3.243444000000	-4.003556000000
C	-6.269999000000	-2.249657000000	-4.262322000000
C	-5.092011000000	-0.819166000000	-7.894542000000
C	-4.249384000000	-2.664765000000	-5.612035000000
C	-5.065749000000	-1.837165000000	-4.743581000000
C	-6.819447000000	-3.551239000000	-4.613561000000
C	-5.980816000000	-4.402536000000	-5.444007000000
C	-4.783001000000	-3.973990000000	-5.927487000000
H	-2.338328000000	-3.972761000000	-7.195780000000
H	-1.397547000000	-2.540702000000	-7.622559000000
C	-2.658145000000	-0.706792000000	-6.065361000000
C	-2.045740000000	-2.976032000000	-6.854643000000
C	-3.241265000000	-2.035766000000	-6.592881000000
C	-4.062924000000	-1.775619000000	-7.885532000000
C	-3.833901000000	-2.475662000000	-9.081967000000
C	-4.578536000000	-2.228656000000	-10.232633000000
C	-5.591916000000	-1.266503000000	-10.216538000000
C	-5.847394000000	-0.561472000000	-9.035638000000
H	-3.054225000000	-3.226488000000	-9.135088000000
H	-4.382845000000	-2.772105000000	-11.151598000000
O	-6.292029000000	-1.060932000000	-11.370582000000
H	-6.634749000000	0.190034000000	-9.007371000000
H	-5.322733000000	-0.259853000000	-6.993302000000
O	-7.960232000000	-3.911769000000	-4.221556000000
H	-6.862971000000	-1.620792000000	-3.605425000000
H	-6.343219000000	-5.393905000000	-5.686499000000
H	-4.221411000000	-4.637762000000	-6.574734000000
H	-3.428868000000	-4.092522000000	-3.904626000000
H	-4.679739000000	-0.867078000000	-4.453051000000
H	-6.949193000000	-0.362711000000	-11.223574000000
H	-1.474274000000	-3.096210000000	-5.934446000000
H	-3.409442000000	0.079571000000	-5.952568000000
H	-2.172289000000	-0.865398000000	-5.099776000000
H	-1.911353000000	-0.336444000000	-6.775129000000
C	-12.401782000000	-10.652294000000	-2.774956000000
C	-11.057696000000	-9.956280000000	-2.710590000000
O	-10.005435000000	-10.548751000000	-2.974564000000
H	-12.646785000000	-10.843971000000	-3.825607000000
H	-12.328110000000	-11.622404000000	-2.276348000000
N	-11.062521000000	-8.645860000000	-2.332020000000
C	-9.889717000000	-7.781928000000	-2.527341000000
C	-8.697422000000	-8.173564000000	-1.646527000000
O	-7.549873000000	-7.874128000000	-1.982394000000
C	-10.269759000000	-6.315156000000	-2.289736000000
H	-11.958814000000	-8.183137000000	-2.247301000000
H	-9.535572000000	-7.895640000000	-3.555384000000
H	-11.030576000000	-6.001019000000	-3.011086000000

H	-10.646916000000	-6.160665000000	-1.271941000000
H	-9.395274000000	-5.677835000000	-2.440386000000
N	-8.957478000000	-8.819948000000	-0.482018000000
C	-7.879338000000	-9.321678000000	0.352230000000
C	-7.185861000000	-10.599414000000	-0.149441000000
O	-6.143162000000	-10.963390000000	0.398469000000
H	-9.917036000000	-9.051468000000	-0.265575000000
H	-8.267712000000	-9.524670000000	1.354497000000
H	-7.102502000000	-8.560467000000	0.446208000000
N	-7.807095000000	-11.281157000000	-1.137953000000
C	-7.286734000000	-12.536197000000	-1.651746000000
C	-6.647790000000	-12.494371000000	-3.049642000000
O	-5.628316000000	-13.146455000000	-3.271581000000
H	-8.643120000000	-10.894810000000	-1.565982000000
H	-6.522806000000	-12.891940000000	-0.961460000000
N	-7.292254000000	-11.791351000000	-4.016299000000
C	-6.900063000000	-11.941652000000	-5.416504000000
C	-5.479084000000	-11.445142000000	-5.734067000000
O	-4.641830000000	-12.196576000000	-6.231731000000
H	-8.146984000000	-11.290310000000	-3.800554000000
H	-6.922303000000	-12.995165000000	-5.708158000000
N	-5.219262000000	-10.150405000000	-5.419077000000
C	-3.895241000000	-9.608315000000	-5.708970000000
C	-2.834698000000	-10.319447000000	-4.856711000000
O	-1.698550000000	-10.532011000000	-5.305697000000
C	-3.854162000000	-8.066340000000	-5.502214000000
O	-4.889331000000	-7.445840000000	-6.247481000000
C	-2.530425000000	-7.476146000000	-5.976603000000
H	-5.990170000000	-9.524722000000	-5.185024000000
H	-3.632006000000	-9.821818000000	-6.749971000000
H	-3.985006000000	-7.861295000000	-4.426670000000
H	-5.725518000000	-7.574874000000	-5.749951000000
H	-2.509747000000	-6.404835000000	-5.755911000000
H	-2.434547000000	-7.610705000000	-7.059440000000
H	-1.669302000000	-7.946396000000	-5.497752000000
N	-3.201307000000	-10.683541000000	-3.612324000000
C	-2.296624000000	-11.343646000000	-2.679142000000
C	-1.749224000000	-12.674865000000	-3.241835000000
O	-0.572365000000	-12.997586000000	-3.066558000000
C	-3.050132000000	-11.590600000000	-1.348257000000
O	-3.832672000000	-10.421791000000	-1.095825000000
C	-2.084591000000	-11.885622000000	-0.203338000000
H	-4.105742000000	-10.411803000000	-3.246617000000
H	-1.416646000000	-10.713991000000	-2.507056000000
H	-3.725211000000	-12.444528000000	-1.498915000000
H	-4.515046000000	-10.640392000000	-0.429051000000
H	-2.641466000000	-12.130557000000	0.707708000000
H	-1.426595000000	-12.726195000000	-0.446554000000
H	-1.463682000000	-11.006205000000	-0.001535000000
N	-2.639230000000	-13.448638000000	-3.908905000000
C	-2.241825000000	-14.697203000000	-4.539206000000
C	-1.442699000000	-14.571462000000	-5.853077000000
O	-1.012282000000	-15.579499000000	-6.397202000000
H	-3.603251000000	-13.145442000000	-4.009966000000
H	-1.614932000000	-15.274647000000	-3.854032000000
N	-1.249268000000	-13.300198000000	-6.294259000000
C	-0.450451000000	-12.946589000000	-7.451998000000

C	0.812182000000	-12.146658000000	-7.043111000000
O	1.475861000000	-11.533084000000	-7.880024000000
C	-1.314205000000	-12.151188000000	-8.491142000000
O	-2.619180000000	-12.697489000000	-8.593449000000
C	-0.718339000000	-12.197791000000	-9.893121000000
H	-1.652504000000	-12.530858000000	-5.773528000000
H	-0.129617000000	-13.891510000000	-7.903773000000
H	-1.362578000000	-11.105697000000	-8.146042000000
H	-3.077570000000	-12.598190000000	-7.741514000000
H	-1.346865000000	-11.618305000000	-10.577827000000
H	-0.697846000000	-13.234861000000	-10.248915000000
H	0.295801000000	-11.796502000000	-9.891434000000
N	1.120868000000	-12.164485000000	-5.717532000000
C	2.230237000000	-11.421295000000	-5.141595000000
C	1.803446000000	-10.643690000000	-3.884952000000
O	0.816760000000	-9.651537000000	-4.182353000000
C	2.990878000000	-9.927199000000	-3.252602000000
H	0.553075000000	-12.722802000000	-5.090172000000
H	2.597672000000	-10.737200000000	-5.909904000000
H	1.385364000000	-11.360979000000	-3.162860000000
H	0.071127000000	-10.080363000000	-4.646980000000
H	2.666602000000	-9.362354000000	-2.372421000000
H	3.757663000000	-10.643813000000	-2.938624000000
H	3.441735000000	-9.225429000000	-3.964375000000
C	4.532108000000	-5.697820000000	-8.584734000000
C	3.272203000000	-6.215863000000	-7.880271000000
C	2.804184000000	-7.615803000000	-8.325148000000
C	3.839240000000	-8.708541000000	-8.013067000000
C	1.447233000000	-7.966724000000	-7.695264000000
H	4.394056000000	-5.677627000000	-9.673303000000
H	3.444018000000	-6.232887000000	-6.792164000000
H	2.452160000000	-5.502569000000	-8.048460000000
H	2.667433000000	-7.584260000000	-9.418032000000
H	0.692154000000	-7.203768000000	-7.926656000000
H	1.087486000000	-8.934533000000	-8.058503000000
H	1.522029000000	-8.044631000000	-6.602516000000
H	4.775060000000	-8.561956000000	-8.564802000000
H	4.083408000000	-8.711871000000	-6.941019000000
H	3.444147000000	-9.698411000000	-8.263855000000
O	-7.166423000000	-8.038397000000	-4.761500000000
H	-7.132677000000	-7.867420000000	-3.795189000000
H	-7.858146000000	-7.412220000000	-5.096834000000
O	-9.028363000000	-6.141435000000	-5.346708000000
H	-8.649661000000	-5.319749000000	-4.925782000000
H	-9.181397000000	-5.903423000000	-6.273856000000
H	3.049456000000	-12.105843000000	-4.876032000000
H	5.404464000000	-6.326079000000	-8.374223000000
H	-13.214249000000	-10.074019000000	-2.325682000000
H	4.775769000000	-4.677835000000	-8.263243000000
H	-8.097165000000	-13.276369000000	-1.679212000000
H	-3.142416000000	-15.279727000000	-4.747156000000
H	-7.626295000000	-11.388353000000	-6.019712000000

^{2}I7$_{pB}$

C	1.613789000000	-4.462142000000	0.001281000000
C	2.372722000000	-3.245254000000	0.513597000000
O	2.574696000000	-3.056984000000	1.714336000000
C	0.168303000000	-4.478418000000	0.520473000000
H	1.626005000000	-4.512607000000	-1.090061000000
H	0.178690000000	-4.532928000000	1.614646000000
H	-0.362795000000	-5.356950000000	0.145396000000
N	2.791538000000	-2.363166000000	-0.439058000000
C	3.534841000000	-1.161747000000	-0.101167000000
C	2.763962000000	-0.021241000000	0.588099000000
O	3.385149000000	0.954354000000	0.992171000000
H	2.501775000000	-2.509868000000	-1.396789000000
H	4.347150000000	-1.420836000000	0.584013000000
N	1.419287000000	-0.185177000000	0.716727000000
C	0.615078000000	0.692720000000	1.542803000000
C	0.218813000000	0.141400000000	2.924402000000
O	-0.615896000000	0.746739000000	3.592066000000
H	0.980350000000	-1.025776000000	0.354325000000
H	-0.313777000000	0.957509000000	1.028836000000
H	1.188357000000	1.609991000000	1.701982000000
N	0.844389000000	-0.994903000000	3.323976000000
C	0.584408000000	-1.594936000000	4.618581000000
H	1.524098000000	-1.443635000000	2.718783000000
H	0.473575000000	-2.679233000000	4.514587000000
H	1.397447000000	-1.394047000000	5.328727000000
H	-0.338270000000	-1.164989000000	5.012324000000
H	3.987561000000	-0.761336000000	-1.012391000000
H	2.131461000000	-5.352057000000	0.382333000000
S	-0.802911000000	-2.961334000000	0.149628000000
Fe	-1.718217000000	-3.067575000000	-1.899715000000
C	-2.248179000000	0.328311000000	-1.748629000000
C	1.093444000000	-2.535306000000	-3.764487000000
C	-1.352248000000	-6.460920000000	-2.341848000000
C	-4.712991000000	-3.591803000000	-0.352862000000
N	-0.793960000000	-1.441591000000	-2.632573000000
C	-1.116040000000	-0.117544000000	-2.412137000000
C	-0.115656000000	0.747868000000	-2.982148000000
C	0.820657000000	-0.058033000000	-3.553158000000
C	0.395212000000	-1.416917000000	-3.326363000000
N	-0.413231000000	-4.248054000000	-2.855209000000
C	0.704109000000	-3.851133000000	-3.558062000000
C	1.423140000000	-4.999410000000	-4.058300000000
C	0.739729000000	-6.104660000000	-3.653994000000
C	-0.401898000000	-5.626353000000	-2.911201000000
N	-2.843680000000	-4.689420000000	-1.518741000000
C	-2.494146000000	-6.019060000000	-1.686138000000
C	-3.473488000000	-6.881438000000	-1.077585000000
C	-4.413323000000	-6.066116000000	-0.519732000000
C	-4.014347000000	-4.709785000000	-0.782125000000
N	-3.203781000000	-1.872795000000	-1.242556000000
C	-4.336195000000	-2.274615000000	-0.571831000000
C	-5.092792000000	-1.129587000000	-0.128336000000
C	-4.402392000000	-0.025142000000	-0.523980000000
C	-3.224984000000	-0.496906000000	-1.209078000000
H	-2.380742000000	1.400674000000	-1.643953000000
H	1.999866000000	-2.366488000000	-4.338910000000

H	-1.179743000000	-7.529956000000	-2.423355000000
H	-5.626278000000	-3.762044000000	0.209200000000
H	0.944705000000	-7.154178000000	-3.832440000000
H	2.327142000000	-4.947759000000	-4.652228000000
H	-3.448106000000	-7.965163000000	-1.081611000000
H	-5.301442000000	-6.347323000000	0.032345000000
H	1.719620000000	0.226094000000	-4.085506000000
H	-0.142319000000	1.829267000000	-2.944369000000
H	-4.646782000000	1.017352000000	-0.365084000000
H	-6.023557000000	-1.181459000000	0.422223000000
O	-3.427859000000	-3.545159000000	-4.379222000000
C	-6.894029000000	-3.035460000000	-4.084409000000
C	-6.266435000000	-0.829834000000	-6.808953000000
C	-4.600785000000	-3.159930000000	-5.122073000000
C	-5.624276000000	-2.602177000000	-4.161630000000
C	-7.382326000000	-4.127692000000	-4.940485000000
C	-6.391325000000	-4.799650000000	-5.782370000000
C	-5.122194000000	-4.370454000000	-5.854330000000
H	-3.013555000000	-3.557043000000	-7.399843000000
H	-2.526092000000	-1.878384000000	-7.657861000000
C	-3.620993000000	-0.791490000000	-5.404534000000
C	-2.846363000000	-2.600080000000	-6.898856000000
C	-4.095155000000	-2.049735000000	-6.169227000000
C	-5.227303000000	-1.707074000000	-7.156800000000
C	-5.278029000000	-2.259375000000	-8.449203000000
C	-6.301999000000	-1.961896000000	-9.344320000000
C	-7.325343000000	-1.088367000000	-8.968011000000
C	-7.301681000000	-0.521463000000	-7.691323000000
H	-4.500403000000	-2.937490000000	-8.781411000000
H	-6.317002000000	-2.394437000000	-10.339878000000
O	-8.309393000000	-0.827874000000	-9.880739000000
H	-8.087409000000	0.166978000000	-7.384214000000
H	-6.285825000000	-0.365604000000	-5.829256000000
O	-8.574731000000	-4.484710000000	-4.912560000000
H	-7.606253000000	-2.611944000000	-3.381771000000
H	-6.713977000000	-5.682528000000	-6.320647000000
H	-4.409959000000	-4.932885000000	-6.448277000000
H	-3.680042000000	-4.130710000000	-3.641999000000
H	-5.269745000000	-1.823056000000	-3.493235000000
H	-8.929848000000	-0.185935000000	-9.501450000000
H	-2.029398000000	-2.741023000000	-6.188986000000
H	-4.427738000000	-0.252785000000	-4.901711000000
H	-2.875815000000	-1.064027000000	-4.652790000000
H	-3.158004000000	-0.095906000000	-6.112794000000
C	-12.462482000000	-11.147410000000	-3.916691000000
C	-11.225147000000	-10.284840000000	-3.776790000000
O	-10.088903000000	-10.743035000000	-3.942107000000
H	-12.602957000000	-11.388632000000	-4.976328000000
H	-12.305131000000	-12.090367000000	-3.386486000000
N	-11.421002000000	-8.976795000000	-3.444751000000
C	-10.353314000000	-7.976513000000	-3.594182000000
C	-9.168656000000	-8.219756000000	-2.650746000000
O	-8.033995000000	-7.852898000000	-2.958974000000
C	-10.922524000000	-6.568013000000	-3.382391000000
H	-12.371573000000	-8.628722000000	-3.441705000000
H	-9.941588000000	-8.045138000000	-4.604909000000
H	-11.702498000000	-6.362361000000	-4.123660000000

H	-11.345254000000	-6.454823000000	-2.377270000000
H	-10.133447000000	-5.824613000000	-3.519997000000
N	-9.431995000000	-8.802804000000	-1.452260000000
C	-8.361384000000	-9.150155000000	-0.533836000000
C	-7.652227000000	-10.488767000000	-0.799032000000
O	-6.711640000000	-10.818108000000	-0.072217000000
H	-10.377683000000	-9.105835000000	-1.263107000000
H	-8.748812000000	-9.178159000000	0.488371000000
H	-7.595741000000	-8.372418000000	-0.581476000000
N	-8.136143000000	-11.240513000000	-1.811458000000
C	-7.567277000000	-12.530491000000	-2.162384000000
C	-6.720330000000	-12.561546000000	-3.446236000000
O	-5.642261000000	-13.154022000000	-3.456069000000
H	-8.891052000000	-10.878342000000	-2.385865000000
H	-6.921229000000	-12.849061000000	-1.345281000000
N	-7.244752000000	-11.976766000000	-4.554030000000
C	-6.614479000000	-12.176078000000	-5.857408000000
C	-5.194248000000	-11.595179000000	-5.967913000000
O	-4.241888000000	-12.305021000000	-6.288152000000
H	-8.148900000000	-11.519593000000	-4.511810000000
H	-6.521372000000	-13.242807000000	-6.078366000000
N	-5.071801000000	-10.274757000000	-5.675366000000
C	-3.757571000000	-9.647405000000	-5.779108000000
C	-2.792458000000	-10.266711000000	-4.758365000000
O	-1.597761000000	-10.437002000000	-5.045367000000
C	-3.856920000000	-8.102619000000	-5.620175000000
O	-4.754417000000	-7.572763000000	-6.582409000000
C	-2.505766000000	-7.427865000000	-5.834722000000
H	-5.910906000000	-9.695973000000	-5.632742000000
H	-3.322051000000	-9.859261000000	-6.761101000000
H	-4.218420000000	-7.885940000000	-4.600333000000
H	-5.669226000000	-7.750939000000	-6.273868000000
H	-2.591934000000	-6.357194000000	-5.627022000000
H	-2.190143000000	-7.557293000000	-6.875291000000
H	-1.728482000000	-7.842878000000	-5.190160000000
N	-3.302751000000	-10.604209000000	-3.558283000000
C	-2.498888000000	-11.183072000000	-2.486412000000
C	-1.828834000000	-12.509587000000	-2.908394000000
O	-0.680795000000	-12.776777000000	-2.546141000000
C	-3.408246000000	-11.398164000000	-1.253653000000
O	-4.196813000000	-10.213187000000	-1.123633000000
C	-2.597912000000	-11.677762000000	0.009488000000
H	-4.250399000000	-10.341425000000	-3.317633000000
H	-1.675513000000	-10.506239000000	-2.233445000000
H	-4.071313000000	-12.247659000000	-1.469521000000
H	-4.981803000000	-10.423227000000	-0.578440000000
H	-3.271526000000	-11.873045000000	0.851073000000
H	-1.941268000000	-12.543847000000	-0.119932000000
H	-1.978580000000	-10.809385000000	0.258442000000
N	-2.575312000000	-13.341696000000	-3.673042000000
C	-2.029086000000	-14.582267000000	-4.199581000000
C	-1.015764000000	-14.440524000000	-5.354800000000
O	-0.434189000000	-15.432160000000	-5.774264000000
H	-3.523012000000	-13.084362000000	-3.931313000000
H	-1.512966000000	-15.129092000000	-3.405725000000
N	-0.826740000000	-13.172714000000	-5.807172000000
C	0.122517000000	-12.801874000000	-6.838667000000

C	1.216047000000	-11.858253000000	-6.276674000000
O	1.907485000000	-11.163130000000	-7.022433000000
C	-0.614889000000	-12.152768000000	-8.060147000000
O	-1.840546000000	-12.815797000000	-8.326302000000
C	0.208962000000	-12.239646000000	-9.339218000000
H	-1.364416000000	-12.416292000000	-5.401547000000
H	0.599720000000	-13.732903000000	-7.164314000000
H	-0.797710000000	-11.095213000000	-7.811803000000
H	-2.438242000000	-12.696839000000	-7.568503000000
H	-0.336267000000	-11.763203000000	-10.160928000000
H	0.367812000000	-13.291948000000	-9.603702000000
H	1.174526000000	-11.749888000000	-9.205153000000
N	1.349075000000	-11.852569000000	-4.921939000000
C	2.275333000000	-10.987003000000	-4.209089000000
C	1.596756000000	-10.291013000000	-3.018012000000
O	0.553610000000	-9.409756000000	-3.449135000000
C	2.595500000000	-9.461041000000	-2.220154000000
H	0.756958000000	-12.464159000000	-4.371067000000
H	2.645827000000	-10.246235000000	-4.922074000000
H	1.164255000000	-11.064448000000	-2.366925000000
H	-0.042536000000	-9.899437000000	-4.051186000000
H	2.084840000000	-8.938746000000	-1.404526000000
H	3.373738000000	-10.098820000000	-1.786923000000
H	3.077343000000	-8.711377000000	-2.858836000000
C	4.495903000000	-5.530326000000	-8.997411000000
C	3.105885000000	-5.872141000000	-8.447709000000
C	2.829115000000	-7.376630000000	-8.256967000000
C	3.759065000000	-8.027348000000	-7.220940000000
C	1.359681000000	-7.616216000000	-7.877400000000
H	4.673829000000	-6.030850000000	-9.957719000000
H	2.957218000000	-5.359722000000	-7.483789000000
H	2.345591000000	-5.460167000000	-9.126886000000
H	3.009336000000	-7.872126000000	-9.224011000000
H	0.680392000000	-7.220956000000	-8.643810000000
H	1.160353000000	-8.685706000000	-7.753549000000
H	1.115693000000	-7.117656000000	-6.928777000000
H	4.814488000000	-7.952159000000	-7.507044000000
H	3.644975000000	-7.542339000000	-6.240297000000
H	3.512544000000	-9.087716000000	-7.101780000000
O	-7.262388000000	-8.313139000000	-5.595416000000
H	-7.416960000000	-8.059268000000	-4.659041000000
H	-7.966738000000	-7.825201000000	-6.086717000000
O	-9.269016000000	-6.649368000000	-6.467448000000
H	-9.048812000000	-5.835478000000	-5.943084000000
H	-9.351539000000	-6.350412000000	-7.385877000000
H	3.135876000000	-11.566738000000	-3.843888000000
H	5.294269000000	-5.837370000000	-8.312749000000
H	-13.372080000000	-10.668326000000	-3.543277000000
H	4.601192000000	-4.451220000000	-9.162703000000
H	-8.378571000000	-13.261715000000	-2.271423000000
H	-2.858483000000	-15.199557000000	-4.553022000000
H	-7.261138000000	-11.710863000000	-6.607572000000

^{4}I7$_{pB}$

C	2.437433000000	-2.879445000000	-0.280098000000
C	2.441944000000	-1.632833000000	0.594130000000
O	2.643744000000	-1.684020000000	1.808114000000
C	1.355352000000	-3.872131000000	0.171213000000
H	2.302351000000	-2.627696000000	-1.336451000000
H	1.562511000000	-4.181534000000	1.200452000000
H	1.380522000000	-4.763418000000	-0.461566000000
N	2.185804000000	-0.455322000000	-0.047422000000
C	2.146029000000	0.811046000000	0.660465000000
C	0.917570000000	1.093532000000	1.544594000000
O	0.878934000000	2.138868000000	2.184016000000
H	1.934438000000	-0.473311000000	-1.026341000000
H	3.008717000000	0.873735000000	1.330414000000
N	-0.049386000000	0.137098000000	1.573399000000
C	-1.122895000000	0.166019000000	2.546808000000
C	-0.981410000000	-0.802796000000	3.734321000000
O	-1.932137000000	-0.948404000000	4.499001000000
H	0.051731000000	-0.703345000000	1.010155000000
H	-2.081369000000	-0.056490000000	2.068006000000
H	-1.172716000000	1.180814000000	2.950414000000
N	0.218398000000	-1.422575000000	3.869067000000
C	0.480999000000	-2.358662000000	4.945124000000
H	0.943251000000	-1.281753000000	3.173173000000
H	0.727267000000	-3.351052000000	4.549140000000
H	1.314257000000	-2.016006000000	5.570620000000
H	-0.421959000000	-2.425985000000	5.553997000000
H	2.232767000000	1.624644000000	-0.064787000000
H	3.420945000000	-3.356996000000	-0.173720000000
S	-0.348912000000	-3.170017000000	0.192732000000
Fe	-1.394020000000	-3.177674000000	-2.018565000000
C	-1.762576000000	0.226452000000	-1.851258000000
C	1.331398000000	-2.772922000000	-4.066839000000
C	-1.183466000000	-6.576529000000	-2.426896000000
C	-4.440293000000	-3.572209000000	-0.478041000000
N	-0.440711000000	-1.598054000000	-2.835760000000
C	-0.691775000000	-0.265597000000	-2.582521000000
C	0.315918000000	0.557003000000	-3.198089000000
C	1.179142000000	-0.286691000000	-3.836585000000
C	0.702351000000	-1.624784000000	-3.603454000000
N	-0.182427000000	-4.405681000000	-3.024929000000
C	0.912482000000	-4.065273000000	-3.794339000000
C	1.573454000000	-5.249732000000	-4.276477000000
C	0.880426000000	-6.320242000000	-3.795975000000
C	-0.215263000000	-5.785089000000	-3.029003000000
N	-2.629235000000	-4.745328000000	-1.655628000000
C	-2.314492000000	-6.085019000000	-1.793036000000
C	-3.325329000000	-6.907531000000	-1.178535000000
C	-4.246238000000	-6.056011000000	-0.645888000000
C	-3.800836000000	-4.716980000000	-0.924495000000
N	-2.824235000000	-1.932187000000	-1.341161000000
C	-3.978331000000	-2.277467000000	-0.671707000000
C	-4.649815000000	-1.099014000000	-0.193487000000
C	-3.891773000000	-0.030349000000	-0.572508000000
C	-2.759090000000	-0.555399000000	-1.286931000000
H	-1.834063000000	1.301614000000	-1.722788000000
H	2.219708000000	-2.650238000000	-4.678318000000

H	-1.040880000000	-7.651370000000	-2.480671000000
H	-5.356578000000	-3.697404000000	0.089877000000
H	1.048199000000	-7.381311000000	-3.943436000000
H	2.451824000000	-5.247430000000	-4.909511000000
H	-3.327493000000	-7.991334000000	-1.156566000000
H	-5.144402000000	-6.299407000000	-0.092480000000
H	2.055443000000	-0.037826000000	-4.421789000000
H	0.339219000000	1.638652000000	-3.154676000000
H	-4.070157000000	1.021487000000	-0.389164000000
H	-5.574598000000	-1.102495000000	0.369049000000
O	-3.243864000000	-3.612410000000	-4.563341000000
C	-6.683127000000	-2.991452000000	-4.146752000000
C	-6.080664000000	-0.807893000000	-6.893542000000
C	-4.433003000000	-3.199235000000	-5.261153000000
C	-5.402558000000	-2.603034000000	-4.269110000000
C	-7.236330000000	-4.072742000000	-4.977428000000
C	-6.298002000000	-4.782803000000	-5.847739000000
C	-5.018190000000	-4.396906000000	-5.965117000000
H	-2.955891000000	-3.654708000000	-7.597705000000
H	-2.423693000000	-1.994454000000	-7.886546000000
C	-3.386694000000	-0.865590000000	-5.590327000000
C	-2.735385000000	-2.701659000000	-7.110544000000
C	-3.934054000000	-2.107743000000	-6.332906000000
C	-5.092040000000	-1.728128000000	-7.275870000000
C	-5.218347000000	-2.287001000000	-8.560433000000
C	-6.265651000000	-1.954794000000	-9.415499000000
C	-7.236823000000	-1.037938000000	-9.005472000000
C	-7.138091000000	-0.463998000000	-7.735594000000
H	-4.483006000000	-2.998068000000	-8.919247000000
H	-6.338820000000	-2.393412000000	-10.405806000000
O	-8.246011000000	-0.743758000000	-9.879525000000
H	-7.882600000000	0.257413000000	-7.402535000000
H	-6.041916000000	-0.336185000000	-5.918014000000
O	-8.437776000000	-4.390277000000	-4.903574000000
H	-7.356556000000	-2.541056000000	-3.422884000000
H	-6.667662000000	-5.659686000000	-6.365429000000
H	-4.343636000000	-4.987980000000	-6.575023000000
H	-3.482218000000	-4.163512000000	-3.795899000000
H	-4.998285000000	-1.832868000000	-3.618832000000
H	-8.824340000000	-0.075184000000	-9.480242000000
H	-1.894489000000	-2.867346000000	-6.434756000000
H	-4.153437000000	-0.300583000000	-5.054891000000
H	-2.620845000000	-1.162897000000	-4.868990000000
H	-2.930704000000	-0.185836000000	-6.318198000000
C	-12.510147000000	-10.884199000000	-3.644954000000
C	-11.237841000000	-10.066322000000	-3.562483000000
O	-10.125964000000	-10.567213000000	-3.765362000000
H	-12.700645000000	-11.130434000000	-4.695596000000
H	-12.366586000000	-11.827058000000	-3.110680000000
N	-11.373053000000	-8.748391000000	-3.239428000000
C	-10.274882000000	-7.790972000000	-3.439381000000
C	-9.068743000000	-8.064300000000	-2.531913000000
O	-7.932833000000	-7.742757000000	-2.883692000000
C	-10.784587000000	-6.359054000000	-3.233090000000
H	-12.309172000000	-8.364788000000	-3.203656000000
H	-9.899817000000	-7.890672000000	-4.461786000000
H	-11.580752000000	-6.136363000000	-3.951870000000

H	-11.169065000000	-6.213921000000	-2.216770000000
H	-9.973870000000	-5.647971000000	-3.409688000000
N	-9.311635000000	-8.622586000000	-1.317649000000
C	-8.224402000000	-9.004765000000	-0.433408000000
C	-7.588790000000	-10.379428000000	-0.699639000000
O	-6.647294000000	-10.746404000000	0.007627000000
H	-10.261029000000	-8.886508000000	-1.091906000000
H	-8.572610000000	-8.996816000000	0.603121000000
H	-7.425933000000	-8.264778000000	-0.524626000000
N	-8.132746000000	-11.117913000000	-1.691190000000
C	-7.630954000000	-12.435475000000	-2.043098000000
C	-6.810925000000	-12.509999000000	-3.342706000000
O	-5.747906000000	-13.128692000000	-3.365244000000
H	-8.883976000000	-10.728153000000	-2.252463000000
H	-6.983555000000	-12.777986000000	-1.236868000000
N	-7.342410000000	-11.927880000000	-4.448819000000
C	-6.741695000000	-12.158529000000	-5.761029000000
C	-5.307437000000	-11.620691000000	-5.904267000000
O	-4.383255000000	-12.362205000000	-6.235350000000
H	-8.233249000000	-11.446487000000	-4.395031000000
H	-6.683552000000	-13.229688000000	-5.972404000000
N	-5.139689000000	-10.301516000000	-5.629184000000
C	-3.810395000000	-9.713519000000	-5.771154000000
C	-2.839609000000	-10.346461000000	-4.764165000000
O	-1.656524000000	-10.551858000000	-5.075224000000
C	-3.864261000000	-8.164721000000	-5.633102000000
O	-4.762422000000	-7.624636000000	-6.589161000000
C	-2.499183000000	-7.529290000000	-5.877406000000
H	-5.960382000000	-9.697476000000	-5.577845000000
H	-3.403809000000	-9.950857000000	-6.759639000000
H	-4.203890000000	-7.924525000000	-4.611001000000
H	-5.676367000000	-7.767654000000	-6.260122000000
H	-2.557189000000	-6.452638000000	-5.691997000000
H	-2.198810000000	-7.688418000000	-6.918433000000
H	-1.724998000000	-7.949733000000	-5.232657000000
N	-3.331530000000	-10.657313000000	-3.549321000000
C	-2.518191000000	-11.248238000000	-2.491306000000
C	-1.903459000000	-12.601076000000	-2.914381000000
O	-0.754781000000	-12.901555000000	-2.581458000000
C	-3.399230000000	-11.416380000000	-1.231345000000
O	-4.144848000000	-10.204177000000	-1.097071000000
C	-2.563671000000	-11.705464000000	0.012998000000
H	-4.266955000000	-10.368874000000	-3.291318000000
H	-1.666231000000	-10.596083000000	-2.270645000000
H	-4.096462000000	-12.245669000000	-1.416304000000
H	-4.919723000000	-10.382745000000	-0.526852000000
H	-3.219516000000	-11.868186000000	0.875252000000
H	-1.938836000000	-12.593891000000	-0.122428000000
H	-1.910491000000	-10.854312000000	0.233092000000
N	-2.697482000000	-13.416754000000	-3.648072000000
C	-2.207286000000	-14.680633000000	-4.173704000000
C	-1.229032000000	-14.586974000000	-5.363348000000
O	-0.696045000000	-15.602778000000	-5.789412000000
H	-3.643100000000	-13.131354000000	-3.883107000000
H	-1.681826000000	-15.231776000000	-3.389005000000
N	-1.010596000000	-13.331504000000	-5.837012000000
C	-0.078907000000	-13.006266000000	-6.899525000000

C	1.069395000000	-12.105564000000	-6.378578000000
O	1.774649000000	-11.456660000000	-7.152679000000
C	-0.826862000000	-12.335320000000	-8.102612000000
O	-2.082687000000	-12.954511000000	-8.330093000000
C	-0.044974000000	-12.456124000000	-9.405053000000
H	-1.508557000000	-12.552394000000	-5.423721000000
H	0.349844000000	-13.957926000000	-7.232686000000
H	-0.963870000000	-11.270836000000	-7.853548000000
H	-2.651921000000	-12.815429000000	-7.554026000000
H	-0.598164000000	-11.964399000000	-10.212342000000
H	0.068941000000	-13.514424000000	-9.668778000000
H	0.940755000000	-12.000238000000	-9.302572000000
N	1.233889000000	-12.081918000000	-5.027838000000
C	2.217561000000	-11.250063000000	-4.352881000000
C	1.605539000000	-10.515238000000	-3.149316000000
O	0.588531000000	-9.593631000000	-3.559089000000
C	2.661621000000	-9.721777000000	-2.389279000000
H	0.629952000000	-12.657735000000	-4.451877000000
H	2.597237000000	-10.532152000000	-5.084307000000
H	1.159193000000	-11.263238000000	-2.478150000000
H	-0.048301000000	-10.063286000000	-4.134752000000
H	2.197636000000	-9.169407000000	-1.565730000000
H	3.423400000000	-10.388426000000	-1.970523000000
H	3.157695000000	-9.000785000000	-3.049800000000
C	4.683872000000	-6.203912000000	-9.536613000000
C	3.339193000000	-6.365149000000	-8.817435000000
C	2.918494000000	-7.821409000000	-8.536266000000
C	3.883517000000	-8.548080000000	-7.586419000000
C	1.485008000000	-7.877882000000	-7.985994000000
H	4.689216000000	-6.751195000000	-10.487831000000
H	3.365639000000	-5.813542000000	-7.864214000000
H	2.555346000000	-5.885187000000	-9.421118000000
H	2.927774000000	-8.359979000000	-9.496854000000
H	0.771000000000	-7.424019000000	-8.685512000000
H	1.180799000000	-8.913337000000	-7.801647000000
H	1.409006000000	-7.331852000000	-7.035138000000
H	4.899030000000	-8.606376000000	-7.994841000000
H	3.943723000000	-8.024647000000	-6.620877000000
H	3.533784000000	-9.568263000000	-7.396341000000
O	-7.266196000000	-8.269111000000	-5.535564000000
H	-7.385433000000	-7.994092000000	-4.599963000000
H	-7.971216000000	-7.771249000000	-6.015562000000
O	-9.250869000000	-6.566304000000	-6.383199000000
H	-8.989279000000	-5.746704000000	-5.887845000000
H	-9.359563000000	-6.287632000000	-7.305229000000
H	3.063775000000	-11.862469000000	-4.007914000000
H	5.518062000000	-6.580610000000	-8.934552000000
H	-13.386450000000	-10.368445000000	-3.241949000000
H	4.888951000000	-5.149933000000	-9.759988000000
H	-8.477433000000	-13.128478000000	-2.131484000000
H	-3.066331000000	-15.276997000000	-4.490279000000
H	-7.388296000000	-11.682502000000	-6.504452000000

^{2}TS8$_{B}$

C	1.914157000000	-4.451470000000	-0.148744000000
C	2.767513000000	-3.271383000000	0.297140000000
O	3.041646000000	-3.070837000000	1.481290000000
C	0.495976000000	-4.371673000000	0.435432000000
H	1.874095000000	-4.528759000000	-1.237825000000
H	0.552312000000	-4.414432000000	1.528709000000
H	-0.104373000000	-5.220107000000	0.097079000000
N	3.187115000000	-2.435913000000	-0.696578000000
C	4.023391000000	-1.278886000000	-0.427943000000
C	3.366150000000	-0.074436000000	0.269976000000
O	4.069328000000	0.866906000000	0.616159000000
H	2.835925000000	-2.583921000000	-1.633368000000
H	4.852157000000	-1.576391000000	0.220948000000
N	2.021786000000	-0.146128000000	0.469292000000
C	1.319260000000	0.798377000000	1.314657000000
C	0.954359000000	0.296812000000	2.723678000000
O	0.191109000000	0.963764000000	3.417432000000
H	1.515148000000	-0.967753000000	0.156064000000
H	0.386655000000	1.118204000000	0.840110000000
H	1.960461000000	1.676681000000	1.427983000000
N	1.524448000000	-0.870529000000	3.115956000000
C	1.288762000000	-1.428531000000	4.434051000000
H	2.151334000000	-1.368584000000	2.492801000000
H	1.183019000000	-2.515431000000	4.362611000000
H	2.111658000000	-1.201969000000	5.125113000000
H	0.370961000000	-0.991829000000	4.832136000000
H	4.453056000000	-0.929847000000	-1.370916000000
H	2.395575000000	-5.362050000000	0.230867000000
S	-0.397895000000	-2.802091000000	0.087677000000
Fe	-1.434450000000	-2.888269000000	-1.894621000000
C	-1.846389000000	0.526658000000	-1.810664000000
C	1.291718000000	-2.498661000000	-3.914089000000
C	-1.273833000000	-6.295576000000	-2.360877000000
C	-4.374892000000	-3.265193000000	-0.195509000000
N	-0.504977000000	-1.315565000000	-2.726754000000
C	-0.773513000000	0.024732000000	-2.529942000000
C	0.221564000000	0.841039000000	-3.175316000000
C	1.105166000000	-0.010145000000	-3.764411000000
C	0.651204000000	-1.347096000000	-3.475652000000
N	-0.247128000000	-4.131353000000	-2.916582000000
C	0.860799000000	-3.793478000000	-3.666163000000
C	1.508014000000	-4.977565000000	-4.179106000000
C	0.788876000000	-6.046124000000	-3.740104000000
C	-0.302479000000	-5.509586000000	-2.963084000000
N	-2.633228000000	-4.447991000000	-1.472147000000
C	-2.364986000000	-5.797182000000	-1.663146000000
C	-3.360846000000	-6.608214000000	-1.015767000000
C	-4.221053000000	-5.749401000000	-0.397638000000
C	-3.761518000000	-4.415435000000	-0.667165000000
N	-2.847153000000	-1.626161000000	-1.198128000000
C	-3.951866000000	-1.968458000000	-0.450437000000
C	-4.629894000000	-0.786734000000	0.021633000000
C	-3.922999000000	0.281349000000	-0.438059000000
C	-2.813584000000	-0.249111000000	-1.189097000000
H	-1.934145000000	1.605298000000	-1.724910000000
H	2.177939000000	-2.375548000000	-4.530161000000

H	-1.156269000000	-7.371958000000	-2.445715000000
H	-5.258131000000	-3.390367000000	0.423564000000
H	0.940526000000	-7.105837000000	-3.909430000000
H	2.390296000000	-4.973378000000	-4.806943000000
H	-3.408108000000	-7.690777000000	-1.044658000000
H	-5.095342000000	-5.989004000000	0.194347000000
H	1.985633000000	0.229651000000	-4.347009000000
H	0.229037000000	1.923364000000	-3.169111000000
H	-4.114955000000	1.335501000000	-0.284555000000
H	-5.525041000000	-0.791703000000	0.630479000000
O	-3.606749000000	-3.883845000000	-4.370792000000
C	-7.271491000000	-3.843373000000	-4.088013000000
C	-7.351914000000	-1.090944000000	-5.600497000000
C	-4.929951000000	-3.833385000000	-4.758711000000
C	-5.965561000000	-3.573047000000	-3.798543000000
C	-7.654581000000	-4.512352000000	-5.322492000000
C	-6.570563000000	-4.899901000000	-6.206906000000
C	-5.271834000000	-4.622413000000	-5.907570000000
H	-3.990256000000	-2.711744000000	-7.555164000000
H	-3.455048000000	-1.074451000000	-7.148015000000
C	-4.533345000000	-0.952500000000	-4.613167000000
C	-3.776579000000	-2.037968000000	-6.724482000000
C	-4.948842000000	-1.800595000000	-5.795094000000
C	-6.286100000000	-1.650735000000	-6.348995000000
C	-6.610406000000	-2.086525000000	-7.661543000000
C	-7.878794000000	-1.943804000000	-8.198640000000
C	-8.903564000000	-1.376096000000	-7.429847000000
C	-8.628129000000	-0.944120000000	-6.127352000000
H	-5.843980000000	-2.529411000000	-8.285241000000
H	-8.099786000000	-2.270428000000	-9.209690000000
O	-10.131266000000	-1.262959000000	-8.002482000000
H	-9.416838000000	-0.500971000000	-5.522353000000
H	-7.178477000000	-0.740622000000	-4.590709000000
O	-8.863544000000	-4.769602000000	-5.585729000000
H	-8.066586000000	-3.606755000000	-3.387162000000
H	-6.823270000000	-5.477233000000	-7.089495000000
H	-4.470568000000	-4.997098000000	-6.534207000000
H	-3.521516000000	-3.970251000000	-3.401557000000
H	-5.691370000000	-3.113568000000	-2.851340000000
H	-10.747909000000	-0.871304000000	-7.363601000000
H	-2.931190000000	-2.446315000000	-6.163598000000
H	-5.311656000000	-0.808246000000	-3.862762000000
H	-3.660203000000	-1.389934000000	-4.121223000000
H	-4.231247000000	0.041386000000	-4.976487000000
C	-12.496948000000	-11.317277000000	-4.686263000000
C	-11.303290000000	-10.405486000000	-4.486135000000
O	-10.144676000000	-10.836378000000	-4.521050000000
H	-12.539903000000	-11.611056000000	-5.741098000000
H	-12.354904000000	-12.228758000000	-4.099816000000
N	-11.567814000000	-9.090405000000	-4.251428000000
C	-10.530550000000	-8.051334000000	-4.361749000000
C	-9.417287000000	-8.210135000000	-3.318781000000
O	-8.266576000000	-7.843730000000	-3.554788000000
C	-11.177607000000	-6.665001000000	-4.248562000000
H	-12.525964000000	-8.779389000000	-4.351122000000
H	-10.039816000000	-8.137070000000	-5.335731000000
H	-11.929510000000	-6.540337000000	-5.036496000000

H	-11.659107000000	-6.528504000000	-3.272847000000
H	-10.420259000000	-5.889213000000	-4.393819000000
N	-9.765062000000	-8.722301000000	-2.107057000000
C	-8.760203000000	-8.978442000000	-1.090652000000
C	-7.963998000000	-10.285228000000	-1.241449000000
O	-7.045476000000	-10.518326000000	-0.451350000000
H	-10.714945000000	-9.040042000000	-1.972411000000
H	-9.233838000000	-8.992981000000	-0.104848000000
H	-8.033175000000	-8.163392000000	-1.101336000000
N	-8.360019000000	-11.125558000000	-2.221797000000
C	-7.714933000000	-12.405137000000	-2.457345000000
C	-6.790938000000	-12.481282000000	-3.684698000000
O	-5.715290000000	-13.073418000000	-3.600179000000
H	-9.095329000000	-10.838082000000	-2.860700000000
H	-7.107955000000	-12.645319000000	-1.585289000000
N	-7.242683000000	-11.941532000000	-4.844332000000
C	-6.527923000000	-12.180067000000	-6.096561000000
C	-5.105263000000	-11.595224000000	-6.129355000000
O	-4.128386000000	-12.313657000000	-6.340093000000
H	-8.147130000000	-11.484101000000	-4.877894000000
H	-6.418642000000	-13.252923000000	-6.276610000000
N	-5.011455000000	-10.262708000000	-5.890266000000
C	-3.696427000000	-9.630711000000	-5.913662000000
C	-2.812264000000	-10.200862000000	-4.796810000000
O	-1.599405000000	-10.385498000000	-4.983761000000
C	-3.823482000000	-8.082468000000	-5.820927000000
O	-4.632335000000	-7.601790000000	-6.879825000000
C	-2.463748000000	-7.398653000000	-5.929307000000
H	-5.851708000000	-9.684795000000	-5.952098000000
H	-3.181622000000	-9.873157000000	-6.849159000000
H	-4.280572000000	-7.835778000000	-4.847499000000
H	-5.573511000000	-7.736263000000	-6.631338000000
H	-2.578469000000	-6.325246000000	-5.754659000000
H	-2.054063000000	-7.549442000000	-6.933695000000
H	-1.744813000000	-7.791287000000	-5.206837000000
N	-3.410575000000	-10.480405000000	-3.621967000000
C	-2.685848000000	-11.000382000000	-2.467246000000
C	-1.995453000000	-12.349500000000	-2.768657000000
O	-0.876760000000	-12.597368000000	-2.310824000000
C	-3.677172000000	-11.135690000000	-1.288269000000
O	-4.429579000000	-9.921006000000	-1.255233000000
C	-2.960306000000	-11.388671000000	0.035824000000
H	-4.368068000000	-10.193085000000	-3.461392000000
H	-1.876535000000	-10.313049000000	-2.197335000000
H	-4.352922000000	-11.972773000000	-1.513562000000
H	-5.275283000000	-10.096944000000	-0.795548000000
H	-3.694150000000	-11.523725000000	0.837848000000
H	-2.328157000000	-12.280738000000	-0.013505000000
H	-2.326906000000	-10.531854000000	0.289727000000
N	-2.686779000000	-13.219483000000	-3.541419000000
C	-2.109201000000	-14.485481000000	-3.963741000000
C	-1.015203000000	-14.402613000000	-5.049354000000
O	-0.408588000000	-15.414281000000	-5.375611000000
H	-3.615029000000	-12.979475000000	-3.876858000000
H	-1.652584000000	-14.991804000000	-3.108808000000
N	-0.788096000000	-13.158870000000	-5.548046000000
C	0.241509000000	-12.837038000000	-6.516716000000

C	1.306009000000	-11.890312000000	-5.906180000000
O	2.078245000000	-11.249210000000	-6.620821000000
C	-0.391990000000	-12.218640000000	-7.810748000000
O	-1.602276000000	-12.875867000000	-8.149236000000
C	0.525519000000	-12.354874000000	-9.020025000000
H	-1.354533000000	-12.384916000000	-5.222490000000
H	0.726322000000	-13.785223000000	-6.774308000000
H	-0.578209000000	-11.152064000000	-7.608019000000
H	-2.258415000000	-12.720261000000	-7.448224000000
H	0.052248000000	-11.894691000000	-9.893963000000
H	0.685792000000	-13.416739000000	-9.242132000000
H	1.486458000000	-11.876353000000	-8.826290000000
N	1.319022000000	-11.816409000000	-4.547159000000
C	2.195190000000	-10.928955000000	-3.799469000000
C	1.424112000000	-10.150441000000	-2.721275000000
O	0.439492000000	-9.286591000000	-3.300700000000
C	2.361119000000	-9.282350000000	-1.889514000000
H	0.667351000000	-12.387517000000	-4.020367000000
H	2.651261000000	-10.240081000000	-4.514941000000
H	0.920852000000	-10.875128000000	-2.065570000000
H	-0.122374000000	-9.809508000000	-3.908727000000
H	1.790592000000	-8.713543000000	-1.147824000000
H	3.095132000000	-9.899182000000	-1.359637000000
H	2.900517000000	-8.571204000000	-2.526131000000
C	5.365532000000	-6.151048000000	-8.792708000000
C	3.904833000000	-6.283369000000	-8.345096000000
C	3.448798000000	-7.720127000000	-8.022518000000
C	4.209295000000	-8.335019000000	-6.837325000000
C	1.934525000000	-7.769030000000	-7.767246000000
H	5.566181000000	-6.779531000000	-9.669615000000
H	3.734542000000	-5.649850000000	-7.459774000000
H	3.254558000000	-5.878721000000	-9.134037000000
H	3.656429000000	-8.339840000000	-8.909062000000
H	1.371582000000	-7.398013000000	-8.633385000000
H	1.610129000000	-8.792910000000	-7.554626000000
H	1.661728000000	-7.145066000000	-6.904263000000
H	5.288066000000	-8.392856000000	-7.022724000000
H	4.060527000000	-7.733543000000	-5.928351000000
H	3.843198000000	-9.347374000000	-6.636798000000
O	-7.213337000000	-8.341663000000	-6.090671000000
H	-7.471446000000	-8.054357000000	-5.188401000000
H	-7.883792000000	-7.903777000000	-6.671750000000
O	-9.224693000000	-6.863701000000	-7.227788000000
H	-9.120205000000	-6.021571000000	-6.695538000000
H	-9.254086000000	-6.586316000000	-8.155598000000
H	3.001733000000	-11.502873000000	-3.319727000000
H	6.064421000000	-6.452045000000	-8.004447000000
H	-13.449573000000	-10.854209000000	-4.413234000000
H	5.603454000000	-5.115885000000	-9.065928000000
H	-8.485736000000	-13.178901000000	-2.566967000000
H	-2.914279000000	-15.119050000000	-4.343510000000
H	-7.125283000000	-11.740396000000	-6.900901000000

^{4}TS8$_{B}$

C	2.039264000000	-4.408774000000	-0.091480000000
C	2.895993000000	-3.211409000000	0.296490000000
O	3.252051000000	-3.007335000000	1.457652000000
C	0.668916000000	-4.362749000000	0.601433000000
H	1.915407000000	-4.477096000000	-1.175872000000
H	0.814965000000	-4.403619000000	1.685459000000
H	0.073333000000	-5.232247000000	0.308499000000
N	3.223899000000	-2.364466000000	-0.722953000000
C	4.030507000000	-1.176358000000	-0.508788000000
C	3.366672000000	0.017886000000	0.200547000000
O	4.043735000000	1.008497000000	0.449794000000
H	2.806151000000	-2.517038000000	-1.630875000000
H	4.895211000000	-1.438874000000	0.107790000000
N	2.053121000000	-0.119556000000	0.526789000000
C	1.382397000000	0.816652000000	1.405889000000
C	1.212897000000	0.367595000000	2.868668000000
O	0.524022000000	1.049421000000	3.623557000000
H	1.559855000000	-0.974297000000	0.284711000000
H	0.381835000000	1.045367000000	1.026295000000
H	1.964103000000	1.742113000000	1.409607000000
N	1.864665000000	-0.765928000000	3.232321000000
C	1.798555000000	-1.288178000000	4.583608000000
H	2.393582000000	-1.291794000000	2.544363000000
H	1.447662000000	-2.326493000000	4.580318000000
H	2.780804000000	-1.253931000000	5.071407000000
H	1.098725000000	-0.668431000000	5.146582000000
H	4.408888000000	-0.826937000000	-1.473268000000
H	2.570333000000	-5.310912000000	0.239860000000
S	-0.295939000000	-2.823588000000	0.292029000000
Fe	-1.436455000000	-2.904087000000	-1.857457000000
C	-1.810939000000	0.509472000000	-1.769796000000
C	1.242990000000	-2.533671000000	-3.969583000000
C	-1.312928000000	-6.309113000000	-2.322691000000
C	-4.438977000000	-3.257160000000	-0.227895000000
N	-0.509914000000	-1.338652000000	-2.731816000000
C	-0.760038000000	0.000183000000	-2.515483000000
C	0.225388000000	0.810158000000	-3.182801000000
C	1.079368000000	-0.044940000000	-3.809837000000
C	0.620174000000	-1.378483000000	-3.519171000000
N	-0.284260000000	-4.148380000000	-2.916235000000
C	0.810916000000	-3.822466000000	-3.692328000000
C	1.449257000000	-5.013720000000	-4.186582000000
C	0.739047000000	-6.075661000000	-3.711081000000
C	-0.341963000000	-5.528489000000	-2.933056000000
N	-2.684835000000	-4.454555000000	-1.469490000000
C	-2.413836000000	-5.802929000000	-1.647595000000
C	-3.427168000000	-6.607312000000	-1.019462000000
C	-4.301550000000	-5.742658000000	-0.428873000000
C	-3.830836000000	-4.412408000000	-0.693280000000
N	-2.855279000000	-1.637465000000	-1.187185000000
C	-3.981050000000	-1.968645000000	-0.461697000000
C	-4.631920000000	-0.780964000000	0.022129000000
C	-3.890654000000	0.278735000000	-0.407655000000
C	-2.788170000000	-0.260830000000	-1.156797000000
H	-1.881319000000	1.587566000000	-1.667501000000
H	2.117033000000	-2.422203000000	-4.604056000000

H	-1.193031000000	-7.385817000000	-2.395351000000
H	-5.334005000000	-3.369876000000	0.375750000000
H	0.892428000000	-7.138673000000	-3.858711000000
H	2.322917000000	-5.022662000000	-4.826132000000
H	-3.474757000000	-7.690162000000	-1.041051000000
H	-5.189754000000	-5.976350000000	0.144255000000
H	1.944852000000	0.190535000000	-4.415957000000
H	0.247886000000	1.892087000000	-3.163522000000
H	-4.059014000000	1.333981000000	-0.235187000000
H	-5.533909000000	-0.773797000000	0.620595000000
O	-3.642751000000	-3.898137000000	-4.395518000000
C	-7.308427000000	-3.878707000000	-4.125540000000
C	-7.398848000000	-1.108028000000	-5.607766000000
C	-4.964204000000	-3.848466000000	-4.787345000000
C	-6.005363000000	-3.603920000000	-3.828511000000
C	-7.682751000000	-4.538835000000	-5.367580000000
C	-6.592701000000	-4.913083000000	-6.250636000000
C	-5.296955000000	-4.630391000000	-5.944092000000
H	-4.025760000000	-2.693987000000	-7.570696000000
H	-3.498753000000	-1.059713000000	-7.142069000000
C	-4.584182000000	-0.970244000000	-4.610408000000
C	-3.817215000000	-2.029656000000	-6.731123000000
C	-4.992892000000	-1.809177000000	-5.801750000000
C	-6.329537000000	-1.659037000000	-6.357725000000
C	-6.649612000000	-2.086408000000	-7.674043000000
C	-7.917365000000	-1.944185000000	-8.212872000000
C	-8.945678000000	-1.385229000000	-7.442462000000
C	-8.674405000000	-0.961591000000	-6.136317000000
H	-5.880445000000	-2.522336000000	-8.299239000000
H	-8.135059000000	-2.264421000000	-9.226670000000
O	-10.172407000000	-1.271987000000	-8.017025000000
H	-9.465781000000	-0.525069000000	-5.529984000000
H	-7.228881000000	-0.764137000000	-4.595150000000
O	-8.888884000000	-4.800262000000	-5.638178000000
H	-8.107649000000	-3.653261000000	-3.425742000000
H	-6.838616000000	-5.485020000000	-7.138685000000
H	-4.490737000000	-4.995649000000	-6.569971000000
H	-3.559005000000	-3.952989000000	-3.424515000000
H	-5.737925000000	-3.151655000000	-2.875920000000
H	-10.791664000000	-0.885980000000	-7.377233000000
H	-2.971575000000	-2.441420000000	-6.173049000000
H	-5.365058000000	-0.838240000000	-3.860368000000
H	-3.709628000000	-1.408294000000	-4.121210000000
H	-4.286603000000	0.029019000000	-4.962366000000
C	-12.506463000000	-11.364834000000	-4.783181000000
C	-11.319311000000	-10.447253000000	-4.571521000000
O	-10.158249000000	-10.872321000000	-4.595395000000
H	-12.537952000000	-11.658577000000	-5.838432000000
H	-12.365476000000	-12.275750000000	-4.195602000000
N	-11.592329000000	-9.133496000000	-4.339174000000
C	-10.558814000000	-8.089657000000	-4.438643000000
C	-9.455395000000	-8.243523000000	-3.384530000000
O	-8.303932000000	-7.872111000000	-3.609109000000
C	-11.213377000000	-6.706342000000	-4.331804000000
H	-12.550973000000	-8.826910000000	-4.447498000000
H	-10.057678000000	-8.172829000000	-5.407539000000
H	-11.956234000000	-6.584082000000	-5.128612000000

H	-11.707218000000	-6.573137000000	-3.361816000000
H	-10.457723000000	-5.927104000000	-4.466841000000
N	-9.812823000000	-8.757152000000	-2.176309000000
C	-8.816709000000	-9.009619000000	-1.150423000000
C	-8.012000000000	-10.311876000000	-1.295556000000
O	-7.098429000000	-10.540195000000	-0.498436000000
H	-10.762605000000	-9.078933000000	-2.050850000000
H	-9.300189000000	-9.028573000000	-0.169484000000
H	-8.094043000000	-8.190661000000	-1.152257000000
N	-8.396107000000	-11.154259000000	-2.278861000000
C	-7.742461000000	-12.430410000000	-2.509306000000
C	-6.805371000000	-12.500130000000	-3.727003000000
O	-5.726659000000	-13.084951000000	-3.631165000000
H	-9.127117000000	-10.870339000000	-2.924246000000
H	-7.143163000000	-12.668799000000	-1.631470000000
N	-7.248323000000	-11.962894000000	-4.891135000000
C	-6.518935000000	-12.196087000000	-6.136003000000
C	-5.098539000000	-11.605041000000	-6.152423000000
O	-4.116344000000	-12.318836000000	-6.353593000000
H	-8.155755000000	-11.512158000000	-4.934077000000
H	-6.403137000000	-13.268111000000	-6.317006000000
N	-5.013043000000	-10.272438000000	-5.910450000000
C	-3.700776000000	-9.634247000000	-5.919567000000
C	-2.825175000000	-10.200876000000	-4.794057000000
O	-1.609513000000	-10.379192000000	-4.968449000000
C	-3.837417000000	-8.086943000000	-5.826536000000
O	-4.635089000000	-7.609302000000	-6.895196000000
C	-2.480472000000	-7.395081000000	-5.915611000000
H	-5.855028000000	-9.698127000000	-5.981483000000
H	-3.175090000000	-9.872894000000	-6.849969000000
H	-4.308754000000	-7.844462000000	-4.858822000000
H	-5.578659000000	-7.747388000000	-6.658037000000
H	-2.604495000000	-6.322405000000	-5.743012000000
H	-2.055002000000	-7.543648000000	-6.913770000000
H	-1.770082000000	-7.783395000000	-5.182440000000
N	-3.434358000000	-10.483594000000	-3.625550000000
C	-2.719537000000	-11.002041000000	-2.463948000000
C	-2.022789000000	-12.349428000000	-2.759458000000
O	-0.907536000000	-12.594401000000	-2.291855000000
C	-3.721526000000	-11.139515000000	-1.294407000000
O	-4.475618000000	-9.925417000000	-1.266808000000
C	-3.016921000000	-11.393269000000	0.036056000000
H	-4.395395000000	-10.202414000000	-3.475922000000
H	-1.914386000000	-10.312987000000	-2.186137000000
H	-4.394565000000	-11.976756000000	-1.527009000000
H	-5.326136000000	-10.104233000000	-0.817251000000
H	-3.758137000000	-11.530027000000	0.830957000000
H	-2.383394000000	-12.284616000000	-0.008237000000
H	-2.386809000000	-10.536140000000	0.296969000000
N	-2.704671000000	-13.220720000000	-3.539010000000
C	-2.120331000000	-14.485141000000	-3.956689000000
C	-1.017836000000	-14.399328000000	-5.033445000000
O	-0.406285000000	-15.409417000000	-5.355201000000
H	-3.630974000000	-12.983610000000	-3.881800000000
H	-1.669269000000	-14.990334000000	-3.098138000000
N	-0.789184000000	-13.154764000000	-5.529636000000
C	0.249095000000	-12.830090000000	-6.488130000000

C	1.305267000000	-11.881456000000	-5.866219000000
O	2.080455000000	-11.234508000000	-6.572363000000
C	-0.372687000000	-12.212928000000	-7.788253000000
O	-1.577481000000	-12.873405000000	-8.139540000000
C	0.557672000000	-12.346030000000	-8.988049000000
H	-1.359895000000	-12.382328000000	-5.208109000000
H	0.738611000000	-13.777140000000	-6.741070000000
H	-0.563972000000	-11.146946000000	-7.586956000000
H	-2.240865000000	-12.720902000000	-7.444685000000
H	0.092238000000	-11.886656000000	-9.866605000000
H	0.723026000000	-13.407343000000	-9.209039000000
H	1.515299000000	-11.865069000000	-8.784248000000
N	1.307856000000	-11.813033000000	-4.506830000000
C	2.176570000000	-10.926828000000	-3.749048000000
C	1.396181000000	-10.154427000000	-2.673332000000
O	0.412564000000	-9.291611000000	-3.256986000000
C	2.324946000000	-9.286470000000	-1.832350000000
H	0.653676000000	-12.387913000000	-3.987316000000
H	2.636376000000	-10.234195000000	-4.458490000000
H	0.889991000000	-10.882308000000	-2.023712000000
H	-0.143813000000	-9.813936000000	-3.870708000000
H	1.748140000000	-8.720993000000	-1.093050000000
H	3.056307000000	-9.903273000000	-1.298846000000
H	2.868058000000	-8.572610000000	-2.462829000000
C	5.325801000000	-6.122000000000	-8.759183000000
C	3.862313000000	-6.267470000000	-8.325044000000
C	3.420291000000	-7.706125000000	-7.991631000000
C	4.176551000000	-8.299553000000	-6.792886000000
C	1.904276000000	-7.770697000000	-7.750624000000
H	5.542946000000	-6.757044000000	-9.627366000000
H	3.675515000000	-5.626452000000	-7.448355000000
H	3.215245000000	-5.879105000000	-9.124733000000
H	3.643817000000	-8.332485000000	-8.869564000000
H	1.345458000000	-7.414718000000	-8.625691000000
H	1.590006000000	-8.796244000000	-7.531015000000
H	1.615627000000	-7.141829000000	-6.896340000000
H	5.257587000000	-8.346669000000	-6.967614000000
H	4.012471000000	-7.690356000000	-5.891660000000
H	3.820319000000	-9.313890000000	-6.584851000000
O	-7.221324000000	-8.359678000000	-6.134712000000
H	-7.491313000000	-8.075819000000	-5.234824000000
H	-7.888194000000	-7.925432000000	-6.722435000000
O	-9.230217000000	-6.891412000000	-7.289807000000
H	-9.133979000000	-6.049821000000	-6.755694000000
H	-9.255661000000	-6.613024000000	-8.217425000000
H	2.980838000000	-11.500932000000	-3.265785000000
H	6.020100000000	-6.406269000000	-7.960740000000
H	-13.463915000000	-10.906573000000	-4.519109000000
H	5.553971000000	-5.086919000000	-9.040826000000
H	-8.508441000000	-13.207614000000	-2.627826000000
H	-2.920647000000	-15.120821000000	-4.342924000000
H	-7.109412000000	-11.757432000000	-6.945963000000

^{2}I8$_{B}$

C	2.018076000000	-4.432450000000	-0.180994000000
C	2.890070000000	-3.259635000000	0.247665000000
O	3.180676000000	-3.055602000000	1.427308000000
C	0.607530000000	-4.331371000000	0.418201000000
H	1.964777000000	-4.516505000000	-1.269052000000
H	0.675392000000	-4.366263000000	1.511053000000
H	-0.006514000000	-5.175035000000	0.092812000000
N	3.306897000000	-2.434055000000	-0.755482000000
C	4.158384000000	-1.284619000000	-0.502397000000
C	3.521885000000	-0.069853000000	0.196968000000
O	4.238955000000	0.865479000000	0.530781000000
H	2.942601000000	-2.582703000000	-1.687144000000
H	4.991280000000	-1.587729000000	0.138546000000
N	2.179060000000	-0.125954000000	0.411265000000
C	1.496160000000	0.830297000000	1.259581000000
C	1.141343000000	0.339547000000	2.675001000000
O	0.393368000000	1.018233000000	3.374002000000
H	1.660188000000	-0.943804000000	0.108165000000
H	0.561949000000	1.158217000000	0.793726000000
H	2.148151000000	1.702023000000	1.361516000000
N	1.702322000000	-0.832447000000	3.066496000000
C	1.474578000000	-1.381576000000	4.389707000000
H	2.317270000000	-1.340149000000	2.439243000000
H	1.360325000000	-2.468001000000	4.324877000000
H	2.305311000000	-1.157428000000	5.072144000000
H	0.563658000000	-0.936131000000	4.793891000000
H	4.580989000000	-0.944891000000	-1.451936000000
H	2.492725000000	-5.346173000000	0.199521000000
S	-0.270509000000	-2.753880000000	0.065623000000
Fe	-1.343710000000	-2.856417000000	-1.895399000000
C	-1.737971000000	0.562267000000	-1.868691000000
C	1.356032000000	-2.517853000000	-3.957596000000
C	-1.234512000000	-6.269891000000	-2.337366000000
C	-4.245647000000	-3.185451000000	-0.122351000000
N	-0.418008000000	-1.303311000000	-2.768564000000
C	-0.678244000000	0.041647000000	-2.594272000000
C	0.312066000000	0.840699000000	-3.267986000000
C	1.185263000000	-0.025858000000	-3.850378000000
C	0.728608000000	-1.354757000000	-3.530694000000
N	-0.187311000000	-4.122714000000	-2.922501000000
C	0.915601000000	-3.805041000000	-3.688319000000
C	1.542093000000	-5.001383000000	-4.198396000000
C	0.813664000000	-6.056926000000	-3.743739000000
C	-0.261341000000	-5.500450000000	-2.957894000000
N	-2.547885000000	-4.399345000000	-1.427806000000
C	-2.304404000000	-5.753043000000	-1.620516000000
C	-3.297919000000	-6.546695000000	-0.948422000000
C	-4.128169000000	-5.673446000000	-0.310099000000
C	-3.655616000000	-4.347178000000	-0.595732000000
N	-2.735802000000	-1.573914000000	-1.194494000000
C	-3.821695000000	-1.896060000000	-0.410801000000
C	-4.481525000000	-0.702380000000	0.055731000000
C	-3.783739000000	0.353325000000	-0.445093000000
C	-2.696002000000	-0.196638000000	-1.213297000000
H	-1.819012000000	1.642802000000	-1.801566000000
H	2.235942000000	-2.411179000000	-4.585660000000

H	-1.133798000000	-7.348212000000	-2.420064000000
H	-5.112986000000	-3.294423000000	0.521752000000
H	0.949510000000	-7.119787000000	-3.906396000000
H	2.417172000000	-5.013504000000	-4.836152000000
H	-3.367258000000	-7.627863000000	-0.980977000000
H	-4.992925000000	-5.898185000000	0.301335000000
H	2.059926000000	0.198494000000	-4.447703000000
H	0.324451000000	1.922930000000	-3.282897000000
H	-3.967531000000	1.411151000000	-0.307456000000
H	-5.359982000000	-0.691262000000	0.688354000000
O	-3.634026000000	-3.940329000000	-4.275274000000
C	-7.314954000000	-4.036305000000	-4.064132000000
C	-7.738193000000	-1.363618000000	-5.327697000000
C	-4.953970000000	-4.101664000000	-4.593450000000
C	-6.000388000000	-3.838049000000	-3.685722000000
C	-7.659576000000	-4.547680000000	-5.366327000000
C	-6.555904000000	-4.861235000000	-6.236564000000
C	-5.249244000000	-4.652664000000	-5.856755000000
H	-4.277668000000	-2.155162000000	-7.552411000000
H	-3.672756000000	-0.608608000000	-6.931150000000
C	-4.926140000000	-0.739375000000	-4.452086000000
C	-4.085972000000	-1.587440000000	-6.642368000000
C	-5.285629000000	-1.388204000000	-5.758699000000
C	-6.608622000000	-1.593687000000	-6.185740000000
C	-6.922749000000	-2.105863000000	-7.492887000000
C	-8.215728000000	-2.310535000000	-7.914232000000
C	-9.288445000000	-2.078100000000	-7.032685000000
C	-9.035139000000	-1.568511000000	-5.747927000000
H	-6.121758000000	-2.307025000000	-8.192463000000
H	-8.431098000000	-2.682339000000	-8.910526000000
O	-10.535273000000	-2.295182000000	-7.503053000000
H	-9.864074000000	-1.377702000000	-5.070034000000
H	-7.581230000000	-0.978848000000	-4.328716000000
O	-8.881338000000	-4.699040000000	-5.732159000000
H	-8.128099000000	-3.840334000000	-3.371340000000
H	-6.779372000000	-5.307927000000	-7.199599000000
H	-4.433218000000	-4.939452000000	-6.510150000000
H	-3.495052000000	-3.952494000000	-3.307827000000
H	-5.762167000000	-3.463307000000	-2.692434000000
H	-11.176490000000	-2.183006000000	-6.782647000000
H	-3.299198000000	-2.103664000000	-6.078714000000
H	-5.746243000000	-0.651254000000	-3.740173000000
H	-4.112865000000	-1.295436000000	-3.970872000000
H	-4.533394000000	0.271352000000	-4.642682000000
C	-12.521815000000	-11.275284000000	-4.883741000000
C	-11.324229000000	-10.372359000000	-4.665985000000
O	-10.170119000000	-10.816443000000	-4.665742000000
H	-12.542275000000	-11.581751000000	-5.935640000000
H	-12.402566000000	-12.180689000000	-4.283002000000
N	-11.581568000000	-9.052104000000	-4.456510000000
C	-10.534024000000	-8.021245000000	-4.552264000000
C	-9.449869000000	-8.174741000000	-3.478159000000
O	-8.290336000000	-7.822017000000	-3.686790000000
C	-11.176500000000	-6.630634000000	-4.473645000000
H	-12.534286000000	-8.734419000000	-4.583030000000
H	-10.018117000000	-8.120596000000	-5.511835000000
H	-11.914590000000	-6.519192000000	-5.276840000000

H	-11.674797000000	-6.476670000000	-3.508788000000
H	-10.413276000000	-5.859836000000	-4.622144000000
N	-9.833044000000	-8.669356000000	-2.268851000000
C	-8.853108000000	-8.919129000000	-1.226959000000
C	-8.042907000000	-10.219317000000	-1.360357000000
O	-7.126728000000	-10.436614000000	-0.563042000000
H	-10.787695000000	-8.979979000000	-2.153601000000
H	-9.353382000000	-8.940074000000	-0.254417000000
H	-8.131866000000	-8.099010000000	-1.215827000000
N	-8.427839000000	-11.074504000000	-2.332286000000
C	-7.769312000000	-12.349685000000	-2.552161000000
C	-6.830305000000	-12.428823000000	-3.767848000000
O	-5.762720000000	-13.033636000000	-3.670181000000
H	-9.159562000000	-10.798718000000	-2.980296000000
H	-7.170953000000	-12.579975000000	-1.671509000000
N	-7.259852000000	-11.879041000000	-4.930736000000
C	-6.532267000000	-12.119707000000	-6.175306000000
C	-5.103495000000	-11.549288000000	-6.187455000000
O	-4.129710000000	-12.279386000000	-6.371865000000
H	-8.159116000000	-11.412461000000	-4.974627000000
H	-6.432302000000	-13.192920000000	-6.358652000000
N	-5.001036000000	-10.215604000000	-5.959282000000
C	-3.679105000000	-9.598039000000	-5.966831000000
C	-2.822753000000	-10.159806000000	-4.824230000000
O	-1.607580000000	-10.356421000000	-4.983148000000
C	-3.792059000000	-8.046634000000	-5.906202000000
O	-4.582734000000	-7.581414000000	-6.984549000000
C	-2.422833000000	-7.381285000000	-6.011788000000
H	-5.832753000000	-9.628689000000	-6.049667000000
H	-3.149248000000	-9.862602000000	-6.887847000000
H	-4.257942000000	-7.774500000000	-4.944202000000
H	-5.528346000000	-7.686308000000	-6.738769000000
H	-2.528754000000	-6.302027000000	-5.871917000000
H	-1.997901000000	-7.566048000000	-7.004100000000
H	-1.720804000000	-7.759781000000	-5.265245000000
N	-3.447252000000	-10.422208000000	-3.658686000000
C	-2.749616000000	-10.939278000000	-2.485993000000
C	-2.067007000000	-12.297608000000	-2.762986000000
O	-0.959107000000	-12.552069000000	-2.282514000000
C	-3.763279000000	-11.054934000000	-1.324431000000
O	-4.492549000000	-9.826338000000	-1.304154000000
C	-3.075607000000	-11.320957000000	0.012733000000
H	-4.404647000000	-10.123120000000	-3.520611000000
H	-1.938843000000	-10.257665000000	-2.206148000000
H	-4.450434000000	-11.879642000000	-1.560919000000
H	-5.352577000000	-9.990502000000	-0.867351000000
H	-3.827146000000	-11.434640000000	0.801560000000
H	-2.464875000000	-12.228453000000	-0.021684000000
H	-2.425804000000	-10.479156000000	0.275416000000
N	-2.752345000000	-13.168101000000	-3.539992000000
C	-2.181210000000	-14.444644000000	-3.938106000000
C	-1.067110000000	-14.385143000000	-5.004449000000
O	-0.465845000000	-15.406673000000	-5.309454000000
H	-3.672778000000	-12.924007000000	-3.893803000000
H	-1.745634000000	-14.946815000000	-3.069811000000
N	-0.817166000000	-13.149159000000	-5.511448000000
C	0.235004000000	-12.848306000000	-6.462378000000

C	1.302209000000	-11.914028000000	-5.837506000000
O	2.101926000000	-11.294254000000	-6.540826000000
C	-0.365508000000	-12.228128000000	-7.771202000000
O	-1.577049000000	-12.871884000000	-8.130416000000
C	0.573402000000	-12.381474000000	-8.961886000000
H	-1.380904000000	-12.365986000000	-5.203632000000
H	0.710444000000	-13.804940000000	-6.705696000000
H	-0.542271000000	-11.158432000000	-7.576663000000
H	-2.245242000000	-12.705300000000	-7.443366000000
H	0.123356000000	-11.918614000000	-9.846616000000
H	0.723547000000	-13.446341000000	-9.176658000000
H	1.536849000000	-11.915345000000	-8.751259000000
N	1.285365000000	-11.825238000000	-4.479579000000
C	2.158484000000	-10.943271000000	-3.722030000000
C	1.377245000000	-10.149530000000	-2.662584000000
O	0.412732000000	-9.278650000000	-3.264728000000
C	2.309096000000	-9.286138000000	-1.820129000000
H	0.613256000000	-12.380430000000	-3.961506000000
H	2.634627000000	-10.263885000000	-4.433478000000
H	0.854285000000	-10.863924000000	-2.011248000000
H	-0.147830000000	-9.799175000000	-3.876293000000
H	1.731511000000	-8.704108000000	-1.094305000000
H	3.024951000000	-9.907486000000	-1.271035000000
H	2.869582000000	-8.587033000000	-2.451877000000
C	5.577143000000	-6.293393000000	-8.651975000000
C	4.103407000000	-6.391213000000	-8.239601000000
C	3.606859000000	-7.816160000000	-7.924414000000
C	4.323074000000	-8.443718000000	-6.718423000000
C	2.085900000000	-7.829800000000	-7.707608000000
H	5.784836000000	-6.929500000000	-9.521717000000
H	3.926612000000	-5.751533000000	-7.359945000000
H	3.481693000000	-5.974128000000	-9.044912000000
H	3.822678000000	-8.443564000000	-8.803564000000
H	1.553372000000	-7.450778000000	-8.589318000000
H	1.732987000000	-8.844838000000	-7.498406000000
H	1.805207000000	-7.195463000000	-6.854752000000
H	5.404496000000	-8.527414000000	-6.876646000000
H	4.165850000000	-7.835110000000	-5.815659000000
H	3.928411000000	-9.446359000000	-6.523426000000
O	-7.187880000000	-8.280460000000	-6.213574000000
H	-7.450301000000	-7.999072000000	-5.311173000000
H	-7.844822000000	-7.819776000000	-6.794182000000
O	-9.179859000000	-6.784349000000	-7.360609000000
H	-9.090679000000	-5.937842000000	-6.823063000000
H	-9.172053000000	-6.507400000000	-8.288837000000
H	2.950225000000	-11.522928000000	-3.224712000000
H	6.249777000000	-6.606889000000	-7.845991000000
H	-13.476248000000	-10.799969000000	-4.639432000000
H	5.844909000000	-5.264853000000	-8.922638000000
H	-8.532293000000	-13.130545000000	-2.665796000000
H	-2.986462000000	-15.073192000000	-4.325748000000
H	-7.115414000000	-11.669642000000	-6.984278000000

^{4}I8$_{B}$

C	2.748515000000	-3.128671000000	-0.512587000000
C	2.991834000000	-1.796001000000	0.183881000000
O	3.278093000000	-1.730398000000	1.380364000000
C	1.613749000000	-3.907367000000	0.166287000000
H	2.531818000000	-2.995963000000	-1.576147000000
H	1.876354000000	-4.087781000000	1.214827000000
H	1.466745000000	-4.877351000000	-0.315859000000
N	2.844808000000	-0.681130000000	-0.587295000000
C	3.059681000000	0.653595000000	-0.056003000000
C	1.992430000000	1.213355000000	0.901942000000
O	2.183126000000	2.303307000000	1.427797000000
H	2.539866000000	-0.777955000000	-1.546542000000
H	3.997941000000	0.674695000000	0.506404000000
N	0.893703000000	0.438224000000	1.115011000000
C	-0.058231000000	0.732520000000	2.168082000000
C	0.069352000000	-0.108304000000	3.451147000000
O	-0.805135000000	-0.018230000000	4.308767000000
H	0.814917000000	-0.456071000000	0.642190000000
H	-1.082452000000	0.603970000000	1.804915000000
H	0.074112000000	1.781530000000	2.445770000000
N	1.170646000000	-0.895673000000	3.553114000000
C	1.422505000000	-1.712125000000	4.725503000000
H	1.847429000000	-0.927099000000	2.797623000000
H	1.597018000000	-2.755234000000	4.438709000000
H	2.297809000000	-1.352276000000	5.280840000000
H	0.543560000000	-1.652692000000	5.369364000000
H	3.159995000000	1.350068000000	-0.892569000000
H	3.673378000000	-3.715222000000	-0.431718000000
S	0.011850000000	-3.006964000000	0.241595000000
Fe	-1.033384000000	-3.003233000000	-1.840648000000
C	-1.356838000000	0.432378000000	-1.860643000000
C	1.479770000000	-2.760616000000	-4.192010000000
C	-1.133545000000	-6.422796000000	-2.383595000000
C	-4.021539000000	-3.229809000000	-0.122228000000
N	-0.136173000000	-1.442491000000	-2.884020000000
C	-0.346076000000	-0.100345000000	-2.669865000000
C	0.632224000000	0.665219000000	-3.403448000000
C	1.427622000000	-0.232743000000	-4.064214000000
C	0.938901000000	-1.550764000000	-3.732376000000
N	-0.001081000000	-4.345855000000	-3.043153000000
C	1.049586000000	-4.050568000000	-3.881563000000
C	1.613296000000	-5.276921000000	-4.410442000000
C	0.890388000000	-6.307791000000	-3.890745000000
C	-0.128208000000	-5.711440000000	-3.047050000000
N	-2.433398000000	-4.533428000000	-1.482139000000
C	-2.200031000000	-5.884099000000	-1.655495000000
C	-3.193785000000	-6.648491000000	-0.937796000000
C	-3.995167000000	-5.747775000000	-0.296053000000
C	-3.506980000000	-4.430141000000	-0.620842000000
N	-2.471089000000	-1.640538000000	-1.162674000000
C	-3.549025000000	-1.935621000000	-0.368615000000
C	-4.125085000000	-0.713098000000	0.150048000000
C	-3.377173000000	0.315850000000	-0.341672000000
C	-2.338140000000	-0.273284000000	-1.160917000000
H	-1.382119000000	1.514853000000	-1.770508000000
H	2.323154000000	-2.683520000000	-4.873193000000

H	-1.067861000000	-7.505786000000	-2.450096000000
H	-4.874108000000	-3.313535000000	0.546393000000
H	0.993739000000	-7.374758000000	-4.051241000000
H	2.444880000000	-5.328722000000	-5.102135000000
H	-3.282685000000	-7.728856000000	-0.937708000000
H	-4.841252000000	-5.953518000000	0.347538000000
H	2.258751000000	-0.024638000000	-4.726771000000
H	0.693155000000	1.746337000000	-3.419130000000
H	-3.506978000000	1.377016000000	-0.169904000000
H	-4.985696000000	-0.659183000000	0.804726000000
O	-3.431107000000	-3.995245000000	-4.310395000000
C	-7.108384000000	-3.986311000000	-4.023123000000
C	-7.485797000000	-1.314052000000	-5.308117000000
C	-4.759971000000	-4.125486000000	-4.597256000000
C	-5.781521000000	-3.820803000000	-3.673240000000
C	-7.493396000000	-4.502913000000	-5.311761000000
C	-6.416377000000	-4.860707000000	-6.198499000000
C	-5.097349000000	-4.685473000000	-5.846586000000
H	-4.080077000000	-2.217699000000	-7.576462000000
H	-3.413103000000	-0.694912000000	-6.960510000000
C	-4.646595000000	-0.763670000000	-4.477096000000
C	-3.859007000000	-1.658159000000	-6.667954000000
C	-5.041371000000	-1.415832000000	-5.772167000000
C	-6.375810000000	-1.587528000000	-6.178858000000
C	-6.723550000000	-2.106864000000	-7.474390000000
C	-8.028024000000	-2.279908000000	-7.874547000000
C	-9.080565000000	-2.006720000000	-6.980749000000
C	-8.794059000000	-1.487853000000	-5.706810000000
H	-5.939003000000	-2.340368000000	-8.182572000000
H	-8.268177000000	-2.658046000000	-8.862785000000
O	-10.340156000000	-2.195730000000	-7.429657000000
H	-9.607221000000	-1.264771000000	-5.019708000000
H	-7.303921000000	-0.920759000000	-4.316718000000
O	-8.725921000000	-4.620580000000	-5.652868000000
H	-7.901608000000	-3.757954000000	-3.317246000000
H	-6.671319000000	-5.313323000000	-7.150842000000
H	-4.303630000000	-5.004529000000	-6.512379000000
H	-3.267192000000	-4.003788000000	-3.345416000000
H	-5.513743000000	-3.440263000000	-2.690041000000
H	-10.966502000000	-2.058677000000	-6.700546000000
H	-3.086497000000	-2.203459000000	-6.111569000000
H	-5.453873000000	-0.652325000000	-3.753916000000
H	-3.838401000000	-1.333655000000	-4.003730000000
H	-4.235807000000	0.236994000000	-4.682636000000
C	-12.587439000000	-11.056909000000	-4.775914000000
C	-11.354285000000	-10.199979000000	-4.570770000000
O	-10.217525000000	-10.686289000000	-4.589073000000
H	-12.633384000000	-11.357249000000	-5.828776000000
H	-12.493910000000	-11.969056000000	-4.180827000000
N	-11.560250000000	-8.872194000000	-4.351042000000
C	-10.477398000000	-7.879694000000	-4.458734000000
C	-9.381323000000	-8.080530000000	-3.404918000000
O	-8.213005000000	-7.770819000000	-3.632028000000
C	-11.066054000000	-6.466880000000	-4.358501000000
H	-12.502393000000	-8.519666000000	-4.463446000000
H	-9.982907000000	-7.990717000000	-5.428224000000
H	-11.814007000000	-6.321704000000	-5.147073000000

H	-11.540386000000	-6.301824000000	-3.383476000000
H	-10.277043000000	-5.724270000000	-4.515568000000
N	-9.762380000000	-8.565808000000	-2.191247000000
C	-8.775247000000	-8.853105000000	-1.165890000000
C	-8.012161000000	-10.179366000000	-1.317904000000
O	-7.086520000000	-10.429303000000	-0.541443000000
H	-10.726210000000	-8.839885000000	-2.060662000000
H	-9.260988000000	-8.861834000000	-0.185802000000
H	-8.026496000000	-8.057961000000	-1.162394000000
N	-8.447739000000	-11.019847000000	-2.281380000000
C	-7.836989000000	-12.315732000000	-2.517455000000
C	-6.920400000000	-12.420674000000	-3.748255000000
O	-5.871342000000	-13.059705000000	-3.670222000000
H	-9.181479000000	-10.716384000000	-2.914461000000
H	-7.233168000000	-12.571160000000	-1.647516000000
N	-7.351345000000	-11.853807000000	-4.902511000000
C	-6.652793000000	-12.114628000000	-6.159505000000
C	-5.206896000000	-11.590088000000	-6.195164000000
O	-4.260311000000	-12.350821000000	-6.396900000000
H	-8.233928000000	-11.355222000000	-4.929625000000
H	-6.590041000000	-13.190120000000	-6.345930000000
N	-5.058039000000	-10.260577000000	-5.968582000000
C	-3.717344000000	-9.685386000000	-6.000614000000
C	-2.859083000000	-10.273963000000	-4.873040000000
O	-1.654674000000	-10.511803000000	-5.055297000000
C	-3.777582000000	-8.131000000000	-5.941589000000
O	-4.572751000000	-7.641267000000	-7.005725000000
C	-2.388578000000	-7.513393000000	-6.076387000000
H	-5.872378000000	-9.647709000000	-6.043828000000
H	-3.213080000000	-9.968360000000	-6.930455000000
H	-4.214833000000	-7.840702000000	-4.971556000000
H	-5.516507000000	-7.719205000000	-6.743119000000
H	-2.454874000000	-6.430364000000	-5.941547000000
H	-1.989033000000	-7.718295000000	-7.075251000000
H	-1.685932000000	-7.911552000000	-5.340727000000
N	-3.469430000000	-10.514038000000	-3.695181000000
C	-2.766446000000	-11.052631000000	-2.535285000000
C	-2.133554000000	-12.432189000000	-2.824112000000
O	-1.024897000000	-12.722049000000	-2.366195000000
C	-3.760524000000	-11.136059000000	-1.354089000000
O	-4.449374000000	-9.884690000000	-1.318407000000
C	-3.056048000000	-11.426235000000	-0.030768000000
H	-4.413281000000	-10.182730000000	-3.538562000000
H	-1.928930000000	-10.397325000000	-2.271895000000
H	-4.479172000000	-11.937443000000	-1.576860000000
H	-5.308199000000	-10.024180000000	-0.870820000000
H	-3.795498000000	-11.512899000000	0.772741000000
H	-2.479071000000	-12.355052000000	-0.076664000000
H	-2.371342000000	-10.608420000000	0.218608000000
N	-2.863105000000	-13.281329000000	-3.584494000000
C	-2.342631000000	-14.576945000000	-3.989825000000
C	-1.250970000000	-14.557151000000	-5.080246000000
O	-0.690270000000	-15.598745000000	-5.394802000000
H	-3.781682000000	-13.007551000000	-3.921005000000
H	-1.904577000000	-15.089921000000	-3.129141000000
N	-0.971471000000	-13.331503000000	-5.596729000000
C	0.067480000000	-13.069611000000	-6.573133000000

C	1.179323000000	-12.169150000000	-5.977581000000
O	1.982908000000	-11.579914000000	-6.702557000000
C	-0.542831000000	-12.435860000000	-7.870815000000
O	-1.781588000000	-13.043085000000	-8.199158000000
C	0.363234000000	-12.622914000000	-9.081962000000
H	-1.502167000000	-12.528968000000	-5.279993000000
H	0.505204000000	-14.042463000000	-6.822860000000
H	-0.681811000000	-11.360354000000	-7.677299000000
H	-2.429599000000	-12.848924000000	-7.500091000000
H	-0.092907000000	-12.150168000000	-9.958319000000
H	0.475645000000	-13.692784000000	-9.294991000000
H	1.345147000000	-12.185826000000	-8.895811000000
N	1.197871000000	-12.074475000000	-4.620167000000
C	2.118916000000	-11.220106000000	-3.888052000000
C	1.392791000000	-10.403966000000	-2.806183000000
O	0.435894000000	-9.506382000000	-3.379786000000
C	2.373729000000	-9.567733000000	-1.992695000000
H	0.519754000000	-12.604188000000	-4.083636000000
H	2.594986000000	-10.555172000000	-4.613183000000
H	0.869228000000	-11.103367000000	-2.138999000000
H	-0.154454000000	-10.010655000000	-3.976600000000
H	1.835354000000	-8.971310000000	-1.248759000000
H	3.089220000000	-10.209584000000	-1.467085000000
H	2.932753000000	-8.883135000000	-2.641349000000
C	5.604749000000	-6.851133000000	-9.093055000000
C	4.167131000000	-6.834052000000	-8.559831000000
C	3.591656000000	-8.215430000000	-8.188764000000
C	4.350477000000	-8.886681000000	-7.033258000000
C	2.095697000000	-8.112151000000	-7.855075000000
H	5.690108000000	-7.501935000000	-9.972509000000
H	4.113624000000	-6.178058000000	-7.676164000000
H	3.514595000000	-6.374762000000	-9.316410000000
H	3.690782000000	-8.863877000000	-9.073435000000
H	1.525493000000	-7.700489000000	-8.697740000000
H	1.684971000000	-9.095744000000	-7.605187000000
H	1.931814000000	-7.451425000000	-6.991791000000
H	5.407680000000	-9.050264000000	-7.272325000000
H	4.307477000000	-8.263172000000	-6.128118000000
H	3.900309000000	-9.856699000000	-6.797971000000
O	-7.181907000000	-8.255775000000	-6.182642000000
H	-7.414846000000	-7.968161000000	-5.274066000000
H	-7.833131000000	-7.769216000000	-6.748654000000
O	-9.133562000000	-6.680315000000	-7.286405000000
H	-9.000764000000	-5.842069000000	-6.744138000000
H	-9.128785000000	-6.396654000000	-8.212630000000
H	2.906844000000	-11.823826000000	-3.413864000000
H	6.315331000000	-7.215136000000	-8.342830000000
H	-13.520168000000	-10.547646000000	-4.516583000000
H	5.927779000000	-5.846555000000	-9.392019000000
H	-8.628305000000	-13.068990000000	-2.622860000000
H	-3.176082000000	-15.180382000000	-4.357297000000
H	-7.235025000000	-11.644760000000	-6.957820000000
